# A framework to model global, regional, and national estimates of intimate partner violence

**DOI:** 10.1186/s12874-022-01634-5

**Published:** 2022-06-01

**Authors:** Mathieu Maheu-Giroux, Lynnmarie Sardinha, Heidi Stöckl, Sarah R. Meyer, Arnaud Godin, Monica Alexander, Claudia García-Moreno

**Affiliations:** 1 Department of Epidemiology and Biostatistics, School of Population and Global Health, McGill University, 2001 Avenue McGill College, Montréal, Québec H3A 1G1 Canada; 2 Department of Sexual and Reproductive Health and Research, World Health Organization, Geneva, Switzerland; 3 Population Health Sciences, Bristol Medical School & School for Policy Studies, University of Bristol, Bristol, UK; 4 Institute for Medical Information Processing, Biometry and Epidemiology (IBE), Ludwig-Maximilians Universität München, Munich, Germany; 5 Departments of Statistical Sciences and Sociology, University of Toronto, Toronto, Canada

**Keywords:** Bayesian inferences, Hierarchical models, Domestic violence, Intimate partner violence, Spousal violence, Sexual assault, Violence against women

## Abstract

**Background:**

Accurate and reliable estimates of violence against women form the backbone of global and regional monitoring efforts to eliminate this human right violation and public health problem. Estimating the prevalence of intimate partner violence (IPV) is challenging due to variations in case definition and recall period, surveyed populations, partner definition, level of age disaggregation, and survey representativeness, among others. In this paper, we aim to develop a sound and flexible statistical modeling framework for global, regional, and national IPV statistics.

**Methods:**

We modeled IPV within a Bayesian multilevel modeling framework, accounting for heterogeneity of age groups using age-standardization, and age patterns and time trends using splines functions. Survey comparability is achieved using adjustment factors which are estimated using exact matching and their uncertainty accounted for. Both in-sample and out-of-sample comparisons are used for model validation, including posterior predictive checks. Post-processing of models’ outputs is performed to aggregate estimates at different geographic levels and age groups.

**Results:**

A total of 307 unique studies conducted between 2000–2018, from 154 countries/areas, and totaling nearly 1.8 million unique women responses informed lifetime IPV. Past year IPV had a similar number of studies (*n* = 332), countries/areas represented (*n* = 159), and individual responses (*n* = 1.8 million). Roughly half of IPV observations required some adjustments. Posterior predictive checks suggest good model fit to data and out-of-sample comparisons provided reassuring results with small median prediction errors and appropriate coverage of predictions’ intervals.

**Conclusions:**

The proposed modeling framework can pool both national and sub-national surveys, account for heterogeneous age groups and age trends, accommodate different surveyed populations, adjust for differences in survey instruments, and efficiently propagate uncertainty to model outputs. Describing this model to reproducible levels of detail enables the accurate interpretation and responsible use of estimates to inform effective violence against women prevention policy and programs, and global monitoring of elimination efforts as part of the Sustainable Development Goals.

**Supplementary Information:**

The online version contains supplementary material available at 10.1186/s12874-022-01634-5.

## Background

Violence against women (VAW) is a gross human rights violation, and a significant global health and development concern. VAW takes many forms, including physical, sexual, and psychological violence perpetrated by an intimate partner —termed intimate partner violence (IPV)— and sexual violence perpetrated by someone other than an intimate partner (e.g., a friend, family member, neighbor, stranger), termed non-partner sexual violence. Other, and often overlapping, types of VAW include trafficking of women and girls, forced or early marriage, and killings in the name of honor [1]. Previous analyses pointed out that VAW has serious short- and long-term impacts on affected individuals, families, and wider societies and that further investments in research and data collection are required to better understand and address VAW epidemic trends [1–3].

In addition to previous international conventions and treaties, countries agreed in 2015 to eliminate all forms of VAW as part of the United Nations *Sustainable Development Goals* (SDGs). Accurate and reliable VAW statistics form the backbone of monitoring efforts, can help guide resources allocation, and, ultimately, enable the deployment of adequate and sustainable intersectoral responses [1, 4–7]. For VAW elimination to be successful, indicators tracking VAW prevalence must be collected, analyzed, and reported.

Estimating prevalence of IPV is challenging for several reasons [8]. First, variations in case definition (definitions based on frequency or severity of acts) and recall periods (lifetime versus past year) are common. Second, lack of disaggregation between different forms of violence (physical, sexual, psychological) can pose comparability issues. Third, differences in surveyed population (all women, ever partnered, or those currently partnered) and whether the perpetrator of violence is the current or most recent partner (versus any previous partners) further compound comparability of estimates. Fourth, reported survey estimates are often not age-disaggregated and, when available, heterogeneous age-group definitions are often encountered, with few observations for women aged 50 years and above. In addition, VAW data are sparse geographically (some countries do not have any survey estimates) and temporally (most countries with data have only one or two estimates at different time points). Considering these issues, comparing and longitudinally tracking IPV statistics requires overcoming several methodological hurdles.

In addition to these issues specific to IPV data, other more general issues need to be considered. First, VAW statistics, like other health indicators, are noisy [9, 10]. This means that the degree of observed heterogeneity can be large; larger than what would be expected from random sampling alone. This heterogeneity can be explained by differences in survey sampling schemes, geographical coverage (national versus only rural or urban), survey instruments and methods, and implementation issues, among others. Taken together, these considerations entail that statistical models are required to adjust, compare, and monitor VAW statistics within and across countries.

The objective of this article is to present a flexible statistical modeling framework for monitoring global, regional, and national IPV statistics which can inform the development of effective policies and programs to address VAW and that are in line with SDG monitoring of target 5.2 that aims to eliminate all forms of violence against all women and girls. Specifically, we focus here on subsection of SDG indicator 5.2.1: the proportion of ever-partnered women and girls aged 15 years and older subjected to physical or sexual violence by a current or former intimate partner in the previous 12 months, by form of violence and by age. In this article, we first present a brief overview of the global VAW database, provide details on the chosen modeling framework, including adjustments and age modeling, present selected results and model validation, including posterior predictive checks, and discuss potential further steps to improve estimates of IPV statistics.

## Methods

### Global violence against women database

The World Health Organization (WHO)’s *Global Database on Prevalence of Violence Against Women* (henceforth referred to as the VAW database) includes prevalence surveys/studies on physical, sexual and psychological IPV, sexual violence by any perpetrator (including a partner), and non-partner sexual violence. This database builds on the earlier database and systematic reviews that WHO curated [2, 3]. The protocol for this systematic review, including the search strategy and a description of all variables can be found elsewhere [11]. Briefly, all population-based studies conducted between 2000 and 2018, representative at either national or sub-national level, and that used specific acts to measure violence were eligible for inclusion. For each study that met the inclusion criteria, we extracted age-specific prevalence estimates by 5-year age groups (if available) and their denominator. If prevalence estimates were only available from a broad age group (e.g., 15–49 years old), the latter was used instead. Estimates were extracted for the different types of IPV reported in the study, such as physical IPV, sexual IPV, and physical and/or sexual IPV. In addition, design-adjusted standard errors and lower and upper limit of the confidence intervals were recorded, whenever available [11]. For each observation, we added their characteristics to the database. These include the country, authors of report/publication, year of publication, the survey’s data collection period (start and end years), the type of violence, the population surveyed (i.e., ever-partnered, currently-partnered, all women), lower and upper age limit of respondents, the recall period (e.g., lifetime, past year), and whether the estimate is representative at the national or sub-national level. For studies with sub-national representativeness, we extracted whether the survey was conducted in a rural, urban, or mixed rural/urban area. We further classified IPV estimates depending on whether the perpetrator referred solely to the spouse –as opposed to all types of intimate partners– and whether the questions on IPV concerned the current or most recent partner –as opposed to any current or previous partners [11].

### Pre-processing

**Fig. 1:**
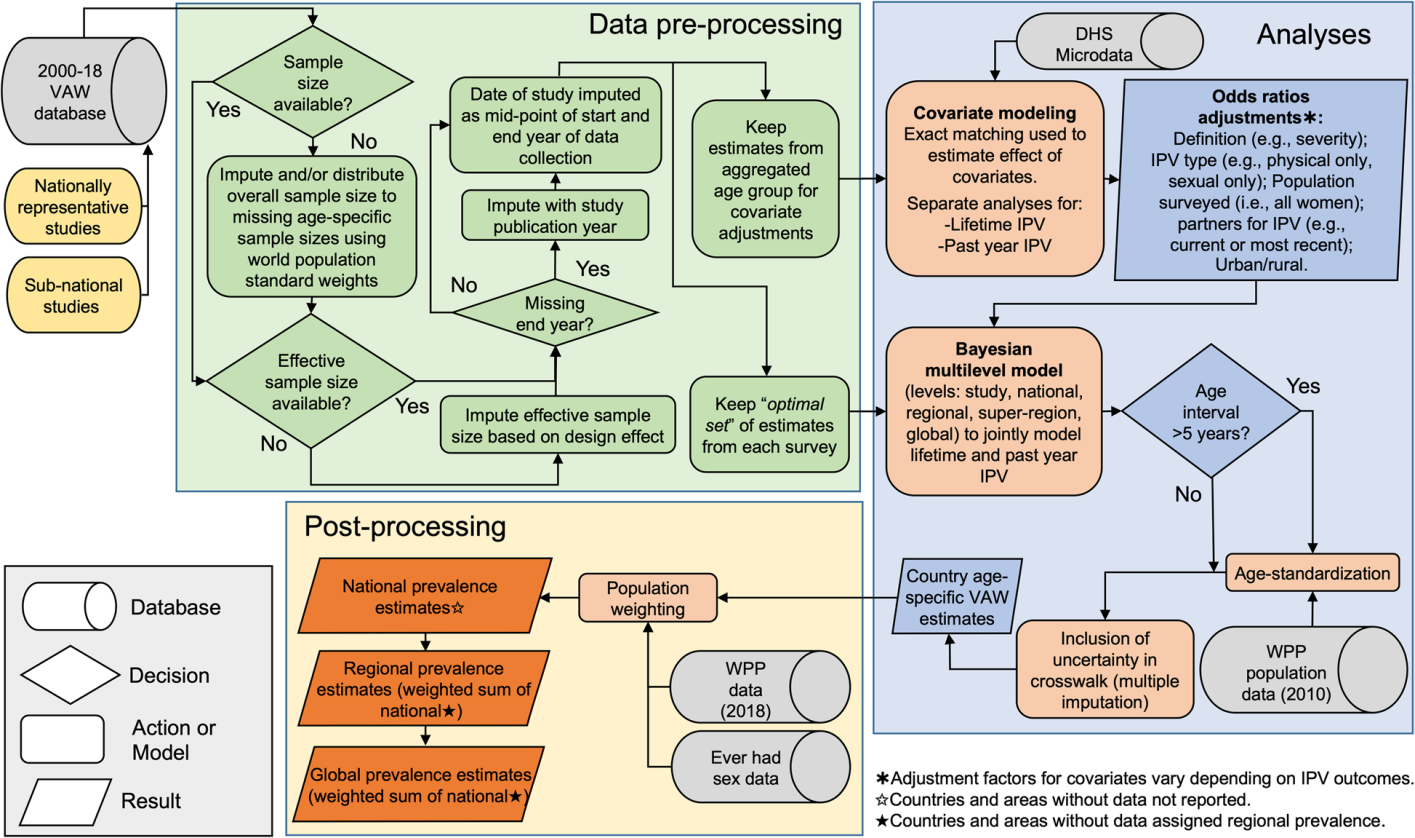
Conceptual overview of data inputs, data pre-processing, data analysis, and post-processing steps required to produce global, regional, and national violence against women statistics. (DHS: Demographic and Health Surveys; IPV: intimate partner violence; VAW: violence against women; WPP: World Population Prospect 2019 revision.)

The conceptual overview of methods used for data analyses describes data inputs, data pre-processing, data analyses, and post-processing to obtain national, regional, and global estimates of IPV statistics (Fig. 1).

Some survey samples sizes are missing and the first step of the data pre-processing involves their imputation. In cases where the overall survey sample size was available but not the age-specific denominators, we imputed them by allocating the overall survey sample size relative to the age-specific number of women in that country in 2010, as reported in the *United Nations World Population Prospect* (WPP) 2019 [12]; where 2010 roughly corresponds to the median year of data collection. In the few instances where the survey sample size was not reported, we conservatively assumed that the sample sizes would be of 3,000 and 1,000 for nationally representative and sub-national surveys, respectively. These denominators are based on the lowest tercile of all sample sizes recorded in the VAW database.

The population-based surveys on VAW often use complex sampling schemes (e.g., stratification and/or clustered sampling) that needs explicit consideration. The extracted estimates of point prevalence adjusted for survey weighting (if required). For stratification and/or clustered sampling, it could impact the precision of estimates. If design-adjusted confidence intervals or standard errors were reported, they were used to obtain an effective sample size. For confidence intervals, Wilson’s formula was used and applied to the upper limit of the interval to approximate standard errors [13]. If it was not possible to derive an effective sample size, a design effect of 2.5 was used. This corresponds to the median design effects obtained from standardized analyses of 89 *Demographic and Health Surveys* (DHS). Surveys for which the end date of data collection was not available were imputed using the date of publication as a proxy. Finally, where the upper age of the survey sample was missing or not reported, we assumed an open-ended age category.

The final pre-processing stage involved the creation of two datasets (Fig. 1). The first one was used to calculate adjustment factors that enabled the combination of different types of estimates. In this case, we only kept the prevalence estimates from the broadest age-group (e.g., 15–49 years). The second dataset was used to model global, regional, and national estimates of IPV statistics. For this, only the most granular age strata were retained as to avoid double-counting women. Similarly, if nationally representative prevalence estimates were available, observations from rural and urban areas of the same study were removed. This process was repeated and, if more than one prevalence estimate remained for each age group, we selected the ones from the “*optimal set*” of observations that used gold-standard methods and survey instruments. Specifically, we applied the following rules for each survey:

- ▪ If a study has estimates for both “*severe physical and/or sexual violence only*” and “*severe and non-severe physical and/or sexual violence*”, we only keep estimates that correspond to this second definition.
- ▪ Retain those from “*physical and/or sexual IPV*”. If it is unavailable we keep “*physical IPV only*” or “*sexual IPV only*”, in that order of preference.
- ▪ Keep estimates that refer to surveyed populations composed of “*ever-partnered/married women*” (if not available, we keep those for “*all women*”; otherwise, we use those for “*currently-partnered women*”).
- ▪ Select estimates that correspond to IPV being perpetrated by “*any current/previous intimate partners/husbands*”. If unavailable, we retain estimates where IPV was experienced from the “*current or most recent intimate partner/husband*”.

### Multilevel modeling framework

Multilevel modeling is a valuable statistical approach used to pool data from different sources. This multilevel approach enables us to “*borrow strength*” across units. For instance, if a country has only one small sub-national survey, the accuracy and precision of that country’s estimate can be improved by countries in the same region. Another appealing characteristic of such multilevel models is that the degree of pooling –in other words, how much information is shared between observations– is determined empirically by the data and not arbitrarily by the user [14]. A Bayesian implementation of these models is straightforward (more so than classical maximum likelihood estimators) and uncertainty is efficiently propagated to model outputs using this approach [10].

The chosen model structure is based on similar meta-regressions of health indicators [2, 3, 9, 10, 15–20] and has five nested levels: 1) individual studies, 2) countries (including territories and areas), 3) regions, 4) super-regions, and 5) the world. Here, regions correspond to the classification used by the *Global Burden of Disease* (GBD) study that groups countries in 21 mutually exclusive regions, themselves grouped into seven “super-regions”, based on the similarities of their epidemiological profiles. The regression model is based on a binomial likelihood for a count outcome, as follow:

$${y}_{it}\sim Binomial\left({p}_{it},{N}_{it}\right)$$

where *y*_*it*_ is the survey-adjusted number of women reporting violence for observation *i* at calendar year *t* and *N*_*it*_ is the effective sample size for that observation. Further, the logit-transformed prevalence estimate *p*_*it*_ is equal to the sum of the study specific intercepts (i.e., the random effects; denoted *α*_*s[i]*_), the country-specific age adjustments (*γ*_*c[i]*_), the country-level time trend (${\delta }_{c\left[i\right],t}$), and the sum of the log-odds ratios of the adjustment factors (i.e., the crosswalk covariate modeling; *X*_*s[i]*_). The model’s equation takes the following form:

$$logit\left({p}_{it}\right)= {\alpha }_{s\left[i\right]}+{\gamma }_{c\left[i\right]}+{\delta }_{c\left[i\right],t}+{X}_{s\left[i\right]}$$

The next sections detail the four terms on the right-hand-side of this equation.

### Random effects to account for study variability

Random effects are useful to account for unobserved heterogeneity and each study is assumed to have its own random intercept. We can further impose a hierarchy on these intercepts. This entails that each study, conducted within a specific country, should yield a prevalence estimate closer to the average of that country as opposed to that of other ones. At the regional level, we posit that the average prevalence of a country should be closer to its regional prevalence than to that of other regions. It is statistically advantageous to nest these effects within clear geographical units because it allows us to borrow strength from other observations to improve prevalence estimates in data sparse settings. To model this hierarchy, we have the following equation for the intercept (*α*_*s[i]*_) of observation *i*:

$${\alpha }_{s[i]}= {u}_{g}+{u}_{z[i]}+ {u}_{r[i]}+{u}_{c[i]}+{u}_{s[i]}$$

where *u*_*g*_ is the overall intercept, *u*_*z*_ is the super-region effect, *u*_*r*_ is the regional effect, *u*_*c*_ is the country effect, and *u*_*s*_ is the study effect. These effects are assumed normally distributed on the logit scale. The following non-informative priors were assigned to these parameters:

$${u}_{g}\sim N(0, 1000)$$

$${u}_{z}\sim N\left(0,{\sigma }_{z}\right) \; and \; {\sigma}_{z}\sim \mathcal{H}\mathcal{C}\left(0, 25\right)$$

$${u}_{r}\sim N\left(0,{\sigma }_{r}\right) \; and \; {\sigma }_{r} \sim \mathcal{H}\mathcal{C}\left(0, 25\right)$$

$${u}_{c}\sim N\left(0,{\sigma }_{c}\right) \; and \; {\sigma }_{c}\sim \mathcal{H}\mathcal{C}\left(0, 25\right)$$

$${u}_{s}\sim N(0,{\sigma }_{s\left[i\right]})$$

The degree of pooling between the different studies depends on the standard deviation of the random effects. A smaller standard deviation means that the degree of pooling of estimates will be greater than for a larger standard deviation. The standard deviations for the super-region (*σ*_*z*_), region (*σ*_*r*_), and country (*σ*_*c*_) random effects are given weakly informative half-Cauchy ($\mathcal{H}\mathcal{C}$) priors with a scale parameter of 25 [21]. Sub-national studies (e.g., a survey conducted in one administrative region) are inherently more variable that those representative at the national level. Because of that variability, they should potentially be given less weight than nationally representative studies. We achieve this by allowing the standard deviation of the study-level random effects to depend on their representativeness [10]. This effectively means that sub-national studies have equal or more variability than those representative at the national level.

$${\sigma }_{s[i]}\left\{\begin{array}{c}{\sigma }_{n}\sim HC\left(0, 25\right) ; {\text{if}} \, {\text{study}} \, {\textit{i}} \, {\text{is}}\, {\text{nationally}}\, {\text{representative}}\\ {\sigma }_{l}={\sigma }_{n}+\tau ; {\text{where}} \tau \sim HC\left(0, 25\right)\text{;} \, \, {\text{if}} \, {\text{study}} \, {\textit{i}} \, {\text{is}} \, {\text{sub-national}}\end{array}\right.$$

### Age modeling

Previous studies suggested that the relationship between age and IPV is not linear [22, 23]. Splines functions are a simple and effective way to model non-linear relationships using piecewise polynomials [24]. We investigated natural cubic splines with either one knot (at age 20, 25, 30, or 35 years) or two knots (at 20 and 35, 20 and 40, 25 and 35, or 25 and 40 years). For each outcome, we used the Deviance Information Criterion [25] and the Widely Applicable Information Criterion [26, 27] to choose the best fitting spline. Age was centered at 30 years old to improve model convergence. There were few observations in the age groups above 65 years old and we hence modified the splines so that prevalence among the ≥ 65 years age group remains constant. This was achieved by recoding all ages above 65 years to that value before calculating the splines and fitting the model. Our model assumes that each country has its own age pattern (${\gamma }_{c}$) but that this pattern is more similar across regions, and super-regions. In practice, this means that we have included country-specific coefficients (random slopes) for the natural cubic spline with *K* degrees of freedom, denoted *λ*_*c[i],k*_.

$${\gamma }_{c\left[i\right]}={\sum }_{k=1}^{K}\left({\lambda }_{c\left[i\right],k}{A}_{a\left[i\right],k}\right)$$

$${\lambda }_{c\left[i\right],k}={\eta }_{g,k}+{\eta }_{z\left[i\right],k}+{\eta }_{r\left[i\right],k}+{\eta }_{c\left[i\right],k}$$

where *η*_*g,k*_ is a vector that contains the coefficients for the global age-prevalence pattern common to all studies, and *η*_*z[i],k*_, *η*_*r[i],k*_, and *η*_*c[i],k*_ contains the super-region, region, and country-specific deviations from this overall pattern, respectively. In all cases, the midpoint of the 5-year age distribution is used to obtain the basis of the natural cubic spline (*A*_*a[i],k*_). The model specification is completed using non-informative normal prior distributions. Hyper-parameters for the standard deviations of the random coefficients (*ν*_*k*_) are given weakly informative half-Cauchy ($\mathcal{H}\mathcal{C}$) prior distributions.

$${\eta }_{gk}\sim N\left(0, 1000\right)$$

$${\eta }_{zk}\sim N\left(0, {\upsilon }_{k}^{z}\right) \; and \; {\upsilon }_{k}^{z}\sim \mathcal{H}\mathcal{C}\left(0, 25\right)$$

$${\eta }_{rk}\sim N\left(0, {\upsilon }_{k}^{r}\right) \; and \; {\upsilon }_{k}^{r}\sim \mathcal{H}\mathcal{C}\left(0, 25\right)$$

$${\eta }_{ck}\sim N\left(0, {\upsilon }_{k}^{c}\right) \; and \; {\upsilon }_{k}^{c}\sim \mathcal{H}\mathcal{C}\left(0, 25\right)$$

A further challenge is that posed by having age groups that are heterogenous. Some prevalence observations refer to 5-year age groups (at best), others to much wider ones (e.g., 15–49 years old). To enable inclusion of all observations and consider these age-heterogeneous categories, an age-standardizing approach was adopted [9]. Age-standardization works by defining the prevalence in a wide age group as a function of both the age-specific prevalence and the underlying age distribution of the sampled population. The sample’s underlying age distribution is estimated from the *UN World Population Prospect* (2019 revision) [12] and we aggregated the 2010 country-level female age distributions for the 21 GBD regions; 2010 being close to the median date of survey data collection. Age-standardization is applied to all age groups for which the width of the age interval was larger than five years. For those observations, the effect of age on prevalence is modeled as a weighted average of the age-specific estimates, over the lower (*l*_*i*_) and upper (*h*_*i*_) bounds of the age interval of that observation *i*, and where *w*_*a[r]*_ is the population weight in region *r* for the relevant age group:

$${p}_{it}=\left\{\begin{array}{cc}{logit}^{-1}\left({\alpha }_{s\left[i\right]}+{\gamma }_{c\left[i\right]}+{\delta }_{c\left[i\right],t}+{X}_{s\left[i\right]}\right),& if {l}_{i}={h}_{i}\\ \frac{{\sum }_{a={l}_{i}}^{{h}_{i}}{logit}^{-1}\left({\alpha }_{s\left[i\right]}+{\sum }_{k=1}^{K}\left({\lambda }_{c\left[i\right],k}{A}_{a,k}\right)+{\delta }_{c\left[i\right],t}+{X}_{s\left[i\right]}\right)\times {w}_{a[r]}}{{\sum }_{a={l}_{i}}^{{u}_{i}}{w}_{a[r]}},& if{ l}_{i}<{h}_{i}\end{array}\right.$$

### Time trends

Prevalence of IPV could exhibit secular changes over the study period. To allow for potential non-linear changes in prevalence, natural cubic splines with one knot placed at the median year of data collection were used (i.e., 2011). We modeled the country-level time trend (${\delta }_{c\left[i\right],t}$) with a multilevel structure:

$${\delta }_{c\left[i\right],t}={\sum }_{k=1}^{K}({\phi }_{gk}+{\phi }_{z\left[i\right],k}+{\phi }_{r\left[i\right],k}+{\phi }_{c\left[i\right],k})\times {T}_{tk}$$

where ${\phi }_{gk}$, ${\phi }_{z\left[i\right],k}$, ${\phi }_{r\left[i\right],k}$, and ${\phi }_{c\left[i\right],k}$ contain the spline’s *K* coefficients for the global, super-region, region, and country-specific time trends. ${T}_{tk}$ contains the basis matrix for the natural cubic spline for calendar year *t*. The following prior distributions complement the model specification.

$${\phi }_{gk}\sim N\left(0, 1000\right)$$

$${\phi }_{zk}\sim N\left(0, {\omega }_{k}^{z}\right) \; and \; {\omega }_{k}^{z}\sim \mathcal{H}\mathcal{C}\left(0, 25\right)$$

$${\phi }_{rk}\sim N\left(0, {\omega }_{k}^{r}\right) \; and \; {\omega }_{k}^{r}\sim \mathcal{H}\mathcal{C}\left(0, 25\right)$$

$${\phi }_{ck}\sim N\left(0, {\omega }_{k}^{c}\right) \; and \; {\omega }_{k}^{c}\sim \mathcal{H}\mathcal{C}\left(0, 25\right)$$

### Covariate modeling

Adjustments are required if surveys using different outcome definitions and/or eligibility criteria are to be compared and combined. Covariate modeling, also termed cross-walk in the field of global descriptive epidemiology, is the process by which these adjustments values are estimated. A common way to conduct covariate modeling is to include indicator variables in the regression model, assuming that these fixed effects are constant across all studies and multiplicatively related [9]. Preliminary models using this approach suggested that the resulting adjustment factors could be affected by compositional bias. This particular bias could occur, for instance, if surveys that required a specific adjustment are more frequent in countries with lower (or higher) IPV prevalence. This could potentially bias adjustment factors. To circumvent this issue, we chose an exact matching identification strategy [28] where the adjustment factors are calculated outside of the main meta-regression models.

Robust estimation can be achieved using matching methods, by ensuring that observations with and without the factor to be adjusted for, have the same distribution of other characteristics (e.g., country, calendar year of data collection, population surveyed, etc.). This is operationalized by matching on the survey’s identifier and this procedure provides us with the ideal comparison group to obtain unbiased adjustment factors. For all adjustment factors except geographical strata, the following procedure was employed:

- ▪ First, exact matching for each adjustment factor separately (Table 1) was performed. If more than one match is available from a specific survey, only the one closest to the “*optimal set*” (as described in the preceding section) was retained. For studies that surveyed ever-partnered/married women, the survey results are not always stratified by the current partnership status of those women. To increase the precision of adjustment factors for this variable, 89 DHS surveys with publicly available microdata were analyzed.
- ▪ Second, the odds ratio comparing prevalence in the observation with the adjustment factor as compared to the reference group within each matched set were calculated.
- ▪ Third, the odds ratios were pooled using meta-analytic approaches. Specifically, random-effect meta-analysis [29] were used and, to account for potential variability of the adjustment between regions, results were stratified by the seven GBD super regions. This level was chosen since there were often too few matched observations to estimate adjustment factors for the 21 GBD regions separately. Region-specific adjustment factors were used if a region had more than three estimates; otherwise, the overall adjustment factor was chosen.

**Table 1:** List of covariates for which adjustments were estimated characteristics used for exact matching

Covariates to adjust*	Exact matching on
IPV definition: “*severe violence*” (ref.: “all severity”)^a^	Survey, population surveyed, violence type, age, geographical strata, reference partners
IPV type: “*physical only*” (ref.: “*physical and/or sexual*”)	Survey, population surveyed, age, geographical strata, severity, reference partners
IPV type: “*sexual only*” (ref.: “*physical and/or sexual*”)	Survey, population surveyed, age, geographical strata, severity, reference partners
Population surveyed: “*all women*” (ref.: “*ever-partnered/married*”)	Survey, violence type, age, geographical strata, severity, reference partners
Population surveyed: “*currently partnered*” (ref.: “*ever-partnered/married*”)	Survey, violence type, age, geographical strata, severity, reference partners
Reference partners: “*current/most recent*” (ref.: “*any current/previous partners*”)	Survey, population surveyed, violence type, age, geographical strata, severity
Geographical strata: “*urban*” or “*rural*” (ref.: “*national*”)	Survey, population surveyed, violence type, age, severity, reference partners

*IPV* intimate partner violence

^*^Separate adjustments estimated for lifetime and past year IPV

^a^The definition of severe IPV includes punching, kicking/dragging, trying to strangle/burn, threatening with a weapon, attacking with weapon, and any type of sexual violence

For geographical strata, a similar exact matching approach was used but, since the adjustment factor was not binary (i.e., “rural”, “urban”, “national”), only surveys that had information on all three categories were used. Matched surveys were then pooled using random effect logistic regressions with one random intercept per survey and random slopes that vary by the seven GBD super regions for the “rural” and “urban” areas (referent was “national”).

Once the adjustment factors are estimated, a vector *X*_*s[i]*_ summarizing adjustments required for each observation was created. If the observation pertains to a study in the “*optimal set*”, all indicators in *C*_*s[i]*_ are zeros, meaning that all covariates belong to the reference group. Otherwise, the adjustment is the sum of the log-odds ratios in vector ${\beta }_{r[i]}$ multiplied by the binary covariates included in *C*_*s[i]*_, as outlined below.

$${X}_{s[i]}=\sum {\beta }_{r[i]}{C}_{s[i]}$$

The approach outlined above did not consider the uncertainty in the meta-analyzed odds ratios. To address this, we independently sampled values of those odds ratios from their distributions. Specifically, we used normal distributions where the mean corresponds to the logit-transformed odds ratios and the standard deviation to the standard error of the adjustment factor. To ensure appropriate coverage of the parameter space, Latin hypercube sampling was used and several ${\beta }_{r[i]}$ vectors were created to represent this uncertainty. Whenever the odds ratios were structurally bound at the null, truncated distributions were used. This was the case, for example, for estimates of severe IPV that cannot be higher than estimates of severe and non-severe IPV combined. The procedure by which the uncertainty of these adjustments was propagated to final results is described in the section titled “Computations”.

Covariates can also be used to improve out-of-sample predictions. For example, if country characteristics like per capita alcohol consumption or gross domestic product can explain between-country variation in IPV. Including them in the modeling could improve prevalence estimates of countries without any survey. However, previous studies on the topic did not find consistent relationships between the above-mentioned country-level covariates and IPV estimates [2, 3, 30]. For this reason, and because data is available for most countries, we do not consider inclusion of covariates to improve out-of-sample predictions.

### Constraints

By definition, estimates of past year IPV should be equal or lower than those of lifetime IPV. Hence, these two IPV outcomes were jointly modelled to ensure that this constraint is respected. This was achieved by jointly performing the meta-regression described above and forcing model predictions for past year IPV (${\widehat{p}}_{{c}^{*},a,t}^{past}$) in a new country ${c}^{*}$ (and country with data on only one type of estimate), for age group *a*, and calendar time *t* to be equal or lower to those of their corresponding prediction for lifetime IPV (${\widehat{p}}_{{c}^{*},a,t}^{life}$), as outlined below:

$$logit\left({\widehat{p}}_{{c}^{*},a,t}^{life}\right)={\widehat{u}}_{g}^{life}+{\widehat{u}}_{z[{c}^{*}]}^{life}+{\widehat{u}}_{r[{c}^{*}]}^{life}+{\widehat{u}}_{{c}^{*}}^{life}+{\widehat{\gamma }}_{{c}^{*},a}^{life}+{\widehat{\delta }}_{{c}^{*},t}^{life}$$

$$logit\left({\widehat{p}}_{{c}^{*},a,t}^{past}\right)={\widehat{u}}_{g}^{past}+{\widehat{u}}_{z[{c}^{*}]}^{past}+{\widehat{u}}_{r[{c}^{*}]}^{past}+{\widehat{u}}_{{c}^{*}}^{past}+{\widehat{\gamma }}_{{c}^{*},a}^{past} +{\widehat{\delta }}_{{c}^{*},t}^{life}$$

$${\widehat{p}}_{{c}^{*},a,t}^{past}\le {\widehat{p}}_{{c}^{*},a,t}^{life}$$

The difference between lifetime and past year IPV should also be relatively small for the youngest age-group of 15–19 years old. These girls and young women have comparable time periods of exposure, more so than those of women in older age groups. Preliminary analyses suggested that including a constraint such that the prevalence ratio of predicted lifetime versus past year IPV among this youngest age group is equal or smaller than 3 improved out-of-sample predictions. This conservative value was chosen based on the empirical observation that prevalence ratio of lifetime to past year IPV among 15–19 years old ($${a}^{*})$$ are always less than 3. This constraint was implemented as follows:

$$RR\le 3\;where\;RR= {\widehat{p}}_{{c}^{*},{a}^{*},t}^{life}/{\widehat{p}}_{{c}^{*},{a}^{*},t}^{past}$$

Models were fitted with and without these constraints to assess the impact of this specification. Adding constraints had either no or minor impact for most country estimates but increased the precision of estimates in countries with data on only one type of recall period for IPV measure.

### Computations

The posterior distributions of the parameters of interest were obtained using Markov chain Monte Carlo simulations implemented through the JAGS software [31]. Inferences were based on 4 chains of 50,000 iterations (with an adaptation phase of 10,000 iterations and an additional 5,000 used as warm up), thinned at every 20^th^ iteration.

Uncertainty in the estimated log-odds ratios of the adjustment factors were considered by sampling a total of 10 vectors from their estimated distributions using Latin hypercube sampling. The Bayesian model, as outlined above, was fitted for each individual set. All draws from the posterior distributions of the sampled vectors were mixed and these mixed draws were used to summarize the overall posterior distributions of parameter of interests [32, 33]. Convergence was examined using traceplots and we ensured that the potential scale reduction factor for all parameters and hyperparameters remained close to one [34]. Moreover, we verified that estimates were based on a minimum of roughly 1,000 independent samples from the posterior distributions [35].

### Model validation

The performance of the models was assessed using posterior predictive checks, and both in-sample and out-of-sample comparisons. Graphical posterior predictive checks enabled the visual assessment of how well simulations from the fitted model compared to the observed data [32]. This procedure was especially useful to understand the ways in which this multilevel model did not fit the observed IPV statistics. Through the iterative process of model building and refinement, we improved the estimates by systematically identifying where model predictions are not congruent with the survey data. In addition to this visual inspection, selected summary statistics for in-sample comparisons were computed, such as the median error, absolute error, and the proportion of empirical observations outside the lower and upper credible intervals. Model performance was also quantified through out-of-sample comparisons by randomly excluding 20% of countries and 20% of studies from the datasets and comparing their model-predicted age-specific prevalence with the known-but-excluded empirical observations.

### Post-processing

The model described above provided us with estimated parameters for the global, regional, and country-level intercepts (${\widehat{u}}_{g}$, ${\widehat{u}}_{z[c]}$,${\widehat{u}}_{r[c]}$, and ${\widehat{u}}_{c}$) that, when combined with the spline’s coefficients (${\widehat{\gamma }}_{c,a}$) and the time trend (${\widehat{\delta }}_{c,t})$, produced estimates of IPV by age and time (${\widehat{p}}_{c,a,t}$) for all countries with available data using the equation below:

$$logit\left({\widehat{p}}_{c,a,t}\right)= {\widehat{u}}_{g}+{\widehat{u}}_{z[c]}+{\widehat{u}}_{r[c]}+{\widehat{u}}_{c}+{\widehat{\gamma }}_{c,a}+{\widehat{\delta }}_{c,t}$$

To estimate the prevalence for broader age groups (e.g., 15–49 years), at higher level of aggregation (i.e., regional and global), and for countries without data the age-specific prevalence estimates were first weighted by the age structure of their respective country (in 2018) considering the proportion of women who ever had sex. This is necessary as the denominator of interest for IPV is composed of ever-partnered/married women. The definition of a partnership varies around the world and the fraction of women who have ever been sexually active is a superior proxy of partnership formation than marriage.

Estimates of age-specific prevalence at the country level were aggregated using the country’s own age distribution of the number of women who have ever had sex. For countries without any empirical observations informing IPV statistics, they were statistically imputed based on their region. The added uncertainty for that country’s estimate is further considered by sampling from the distribution of country-level intercepts (i.e., ${\sim N(0, \sigma }_{c})$). The country-specific prevalence estimates were then aggregated to the regional level by summing the number of women having experienced IPV. Global prevalence estimates were obtained using the same approach.

All analyses are carried in the R statistical software [36] and selected packages [35, 37–39].

## Results

**Fig. 2:**
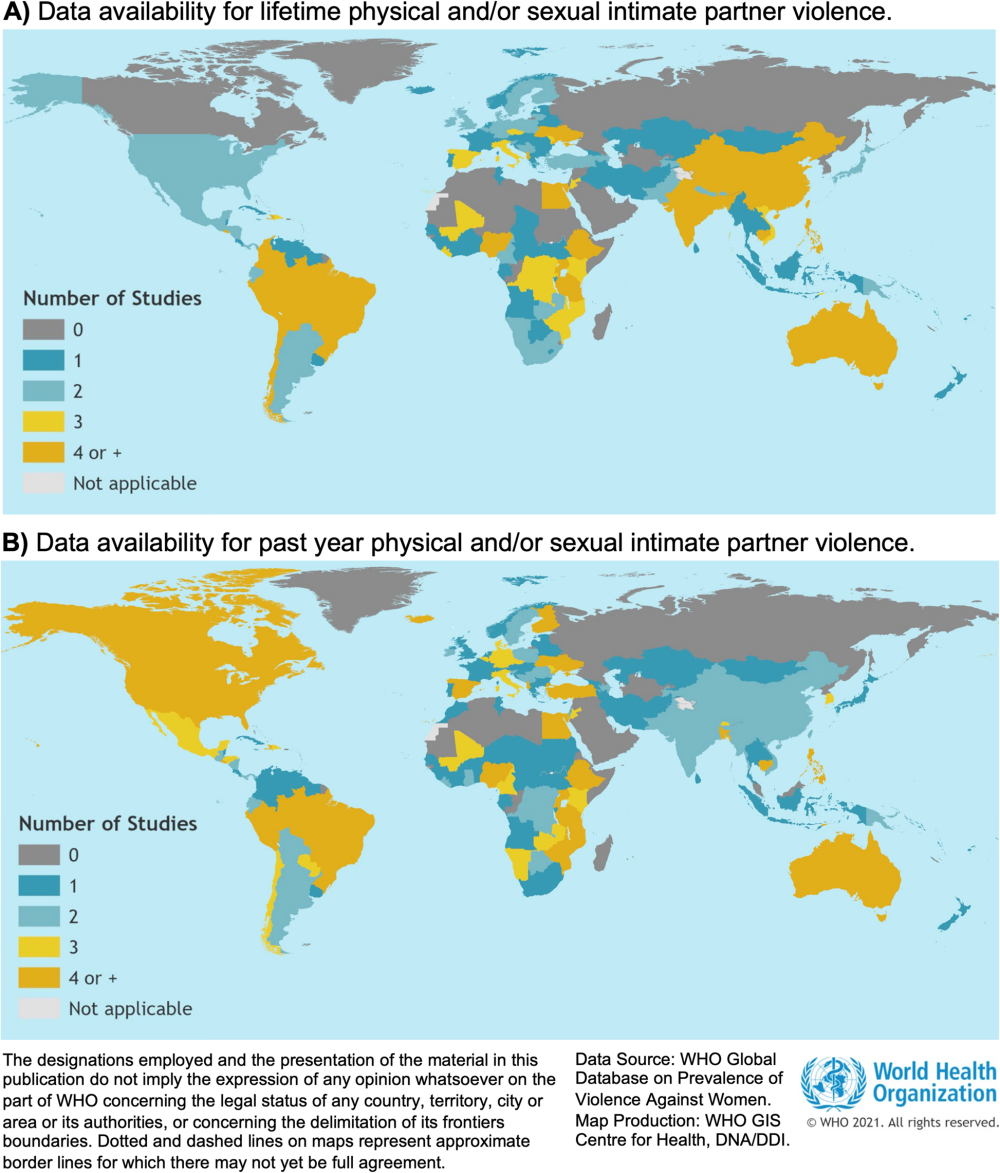
Map of data availability informing estimates of lifetime physical and/or sexual intimate partner violence (IPV; Panel **A**) and past year physical and/or sexual IPV (Panel **B**) for the reference period 2000–2018. (Both nationally and sub-nationally representative studies are included.) Reproduced with permission from the World Health Organization

**Fig. 3:**
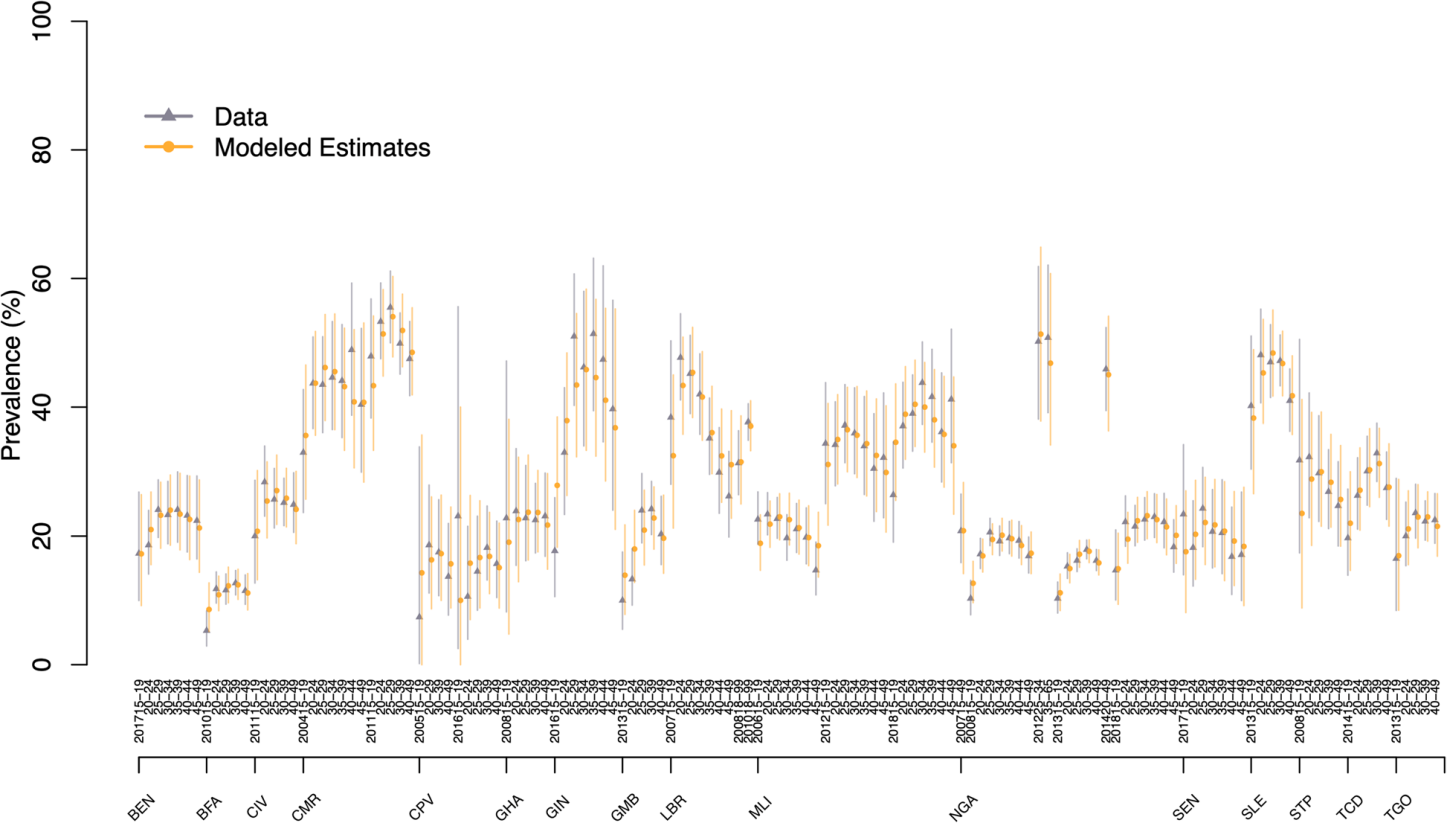
Graphical posterior predictive checks for 16 countries of the Western region of sub-Saharan Africa. Average prevalence for the observed data (triangle) are presented in grey while the model predictions are in yellow (round dots). The vertical lines correspond to the 95% confidence or uncertainty intervals of the data and prediction, respectively. The annotations above the country names described the type of prevalence estimates displayed, the year of data collection, the age group, the surveyed population, and the type of intimate partner violence recorded. (BEN: Benin; BFA: Burkina Faso; CIV: Côte d’Ivoire; CMR: Cameroon; CPV: Cabo Verde; GHA: Ghana; GIN: Guinea; GMB: The Gambia; LBR: Liberia; MLI: Mali; NGA: Nigeria; SEN: Senegal; SLE: Sierra Leone; STP: Sao Tome and Principe; TCD: Chad; TGO: Togo.)

A total of 307 unique studies conducted between 2000–2018, from 154 countries or areas (Fig. 2), covering all 21 regions of the world and totaling 1,767,802 unique women responses informed lifetime IPV (Table 2). Past year IPV had a slightly higher number of studies (*n* = 332), countries or areas represented (*n* = 159; Fig. 3), totaling 1,763,989 individual responses. For both lifetime and past year IPV surveys were conducted between 2000–2018, and the median year of data collection was between 2011 and 2012.

**Table 2:** Characteristics of studies conducted between 2000 to 2018 measuring lifetime and past year intimate partner violence (IPV) informing estimates of global, regional, and national violence against women statistics

Characteristics	Lifetime IPV	Past year IPV
**Sample characteristics and representativeness**		
Number of women interviewed^a^	1,767,802	1,763,989
Number of age-specific observations	1,551	1,598
Number of studies	307	332
Nationally representative studies	260 (85%)	292 (88%)
Number of countries/areas represented	154	159
Countries with 1 study	77 (50%)	81 (51%)
Countries with 2 studies	41 (27%)	33 (21%)
Countries with 3 studies	16 (10%)	19 (12%)
Countries with 4 or more studies	20 (13%)	26 (16%)
Number of GBD regions represented	21 (100%)	21 (100%)
Median date of data collection	2011.5	2011.5
Studies conducted 2000–2004	53 (17%)	65 (20%)
Studies conducted 2005–2009	67 (22%)	67 (20%)
Studies conducted 2010–2014	115 (37%)	119 (36%)
Studies conducted 2015–2018	72 (23%)	81 (24%)
Country-years of observations	302	323
**Study types**		
Studies requiring adjustments		
Violence definition: “*severe violence only*^b^”	4 (1%)	5 (2%)
IPV type: “*sexual violence only*”	5 (2%)	0 (0%)
IPV type: “*physical violence only*”	63 (21%)	84 (25%)
Population surveyed: “*all women*”	19 (6%)	28 (8%)
Population surveyed: “*currently partnered*”	26 (9%)	39 (12%)
Reference partners: “*current/most recent*”	116 (38%)	80 (24%)
Geographical strata: “*rural only*”	14 (5%)	12 (4%)
Geographical strata: “*urban only*”	18 (6%)	13 (4%)
Recall period: “*past two years or more*”	NA	0 (0%)
Observations not requiring any adjustments	635 (41%)	857 (54%)

*IPV* intimate partner violence, *GBD* global burden of disease

^a^Number of women interviewed imputed for surveys with missing denominators

^b^The definition of “severe violence” corresponds to the one reported in the survey description

Country estimates of physical and/or sexual IPV were found to be consistent with the survey data. Data from nationally representative surveys were, as expected, closer to country-level modeled estimates as compared to those from sub-national surveys. This was reflected in the posterior variance estimates at the level of sub-national studies, where the standard deviation of the random effect was twice as large as that of national studies for both lifetime and past year IPV. The latter was similar to that of the across country variance (Table S1).

This proposed framework has been used to produce country-level estimates for both lifetime and past year physical and/or sexual IPV. These estimates can be used to inform evidence-based policy and programming and monitor progress on SDG indicator 5.2 on the elimination of all forms of violence against women and girls. Detailed results have been presented elsewhere [40, 41]. Briefly, an estimated 27% (95%CrI: 23–31%) of ever-married/partnered women aged 15–49 years old have ever experienced physical and/or sexual IPV in their lifetime. Past year IPV was estimated at 13% (95%CrI: 10–16%) with the prevalence of recent IPV being higher among adolescent girls and young women aged 15–24 years old [40, 41].

### Adjustment factors

Roughly half of IPV observations pertained to the optimal set of surveys that collected IPV information using gold-standard definitions and methods (Table 2). The most common adjustments required for lifetime (38%) and past year (24%) IPV studies was that the outcome definition measured violence from a *“current and/or most recent partner only*” as opposed to *“any current/previous partner*”. The second most common adjustment was for “*physical violence only*”, with 21% (lifetime) and 25% (past year) of surveys requiring adjustment. A slightly smaller proportion of studies surveyed “*currently partnered women*” only, whereas the gold-standard VAW questionnaire would have asked IPV questions to ever-partnered women. Table 3 presents these odds ratios for the two IPV outcomes. The detailed forest plots for all meta-analyses are presented as supplementary materials (Figure S1-15).

**Table 3:** Results of random effects meta-analysis for different adjustment factors, stratified by super region, for lifetime intimate partner violence (IPV) and past year IPV

Adjustment factors by super regions (and overall)	Lifetime IPV OR (95%CI)		Past year IPV OR (95%CI)
**Severe violence** (ref. all severity levels)^a^			
Central Europe, Eastern Europe & Central Asia	*NA*		0.39 (0.34–0.45)
High Income	*NA*		*NA*
Latin America & Caribbean	0.51 (0.43–0.60)		0.62 (0.56–0.69)
North Africa & Middle East	*NA*		0.47 (0.34–0.64)
South Asia	*NA*		0.52 (0.44–0.61)
South-East Asia, East Asia & Oceania	0.36 (0.25–0.50)		0.57 (0.48–0.67)
Sub-Saharan Africa	0.37 (0.33–0.42)		0.61 (0.58–0.64)
* Overall*	0.38 (0.34–0.42)		0.57 (0.55–0.60)
**Physical IPV** (ref. physical and/or sexual IPV)			
Central Europe, Eastern Europe & Central Asia	0.93 (0.92–0.95)		0.95 (0.93–0.97)
High Income	0.86 (0.84–0.88)		0.76 (0.70–0.83)
Latin America & Caribbean	0.90 (0.87–0.93)		0.84 (0.81–0.88)
North Africa & Middle East	0.93 (0.90–0.96)		0.83 (0.76–0.91)
South Asia	0.82 (0.73–0.93)		0.78 (0.67–0.90)
South-East Asia, East Asia & Oceania	0.79 (0.73–0.84)		0.78 (0.73–0.83)
Sub-Saharan Africa	0.82 (0.78–0.86)		0.79 (0.76–0.82)
* Overall*	0.86 (0.84–0.87)		0.81 (0.79–0.83)
**Sexual IPV** (ref. physical and/or sexual IPV)			
Central Europe, Eastern Europe & Central Asia	0.22 (0.20–0.25)		0.22 (0.18–0.27)
High Income	0.29 (0.24–0.34)		0.27 (0.22–0.32)
Latin America & Caribbean	0.26 (0.23–0.29)		0.31 (0.28–0.35)
North Africa & Middle East	0.19 (0.15–0.25)		0.28 (0.18–0.43)
South Asia	0.28 (0.21–0.37)		0.36 (0.27–0.47)
South-East Asia, East Asia & Oceania	0.31 (0.26–0.36)		0.35 (0.28–0.44)
Sub-Saharan Africa	0.27 (0.24–0.29)		0.32 (0.29–0.35)
* Overall*	0.26 (0.25–0.28)		0.31 (0.29–0.33)
**All women surveyed** (ref. ever-partnered/married)			
Central Europe, Eastern Europe & Central Asia	*NA*		*NA*
High Income	*NA*		*NA*
Latin America & Caribbean	*NA*		*NA*
North Africa & Middle East	*NA*		*NA*
South Asia	*NA*		*NA*
South-East Asia, East Asia & Oceania	*NA*		*NA*
Sub-Saharan Africa	*NA*		*NA*
* Overall*	0.79 (0.74–0.84)		^b^
**Currently partnered women surveyed** (ref. ever-partnered/married)			
Central Europe, Eastern Europe & Central Asia	0.81 (0.71–0.92)		0.88 (0.80–0.98)
High Income	*NA*		*NA*
Latin America & Caribbean	0.85 (0.80–0.90)		0.89 (0.82–0.96)
North Africa & Middle East	0.94 (0.91–0.99)		1.00 (0.99–1.02)
South Asia	0.98 (0.97–0.98)		1.06 (1.04–1.07)
South-East Asia, East Asia & Oceania	0.92 (0.89–0.96)		1.00 (0.98–1.02)
Sub-Saharan Africa	0.93 (0.92–0.94)		1.02 (1.00–1.04)
* Overall*	0.91 (0.90–0.93)		0.99 (0.97–1.01)
**Partner perpetrating is current or most recent** (ref. any current or previous partners)			
Central Europe, Eastern Europe & Central Asia	*NA*		*NA*
High Income	*NA*		0.68 (0.58–0.81)
Latin America & Caribbean	0.82 (0.71–0.95)		0.99 (0.98–1.00)
North Africa & Middle East	*NA*		*NA*
South Asia	*NA*		*NA*
South-East Asia, East Asia & Oceania	0.88 (0.82–0.95)		0.99 (0.99–1.00)
Sub-Saharan Africa	0.89 (0.86–0.92)		0.98 (0.95–1.01)
* Overall*	0.84 (0.77–0.93)		0.97 (0.95–1.00)
**Geographical urban strata** (ref. nationally representative)			
Central Europe, Eastern Europe & Central Asia	0.98 (0.89–1.08)		0.90 (0.82–0.98)
High Income	*NA*		*NA*
Latin America & Caribbean	1.06 (0.97–1.16)		1.04 (0.96–1.13)
North Africa & Middle East	0.86 (0.79–0.95)		0.88 (0.81–0.96)
South Asia	0.76 (0.70–0.84)		0.77 (0.71–0.83)
South-East Asia, East Asia & Oceania	0.91 (0.83–1.00)		0.93 (0.85–1.01)
Sub-Saharan Africa	0.99 (0.91–1.08)		0.98 (0.91–1.07)
* Overall*	0.92 (0.85–1.01)		0.91 (0.84–0.99)
**Geographical rural strata** (ref. nationally representative)			
Central Europe, Eastern Europe & Central Asia	1.00 (0.92–1.08)		1.05 (0.99–1.12)
High Income	*NA*		*NA*
Latin America & Caribbean	0.88 (0.82–0.95)		0.94 (0.89–0.99)
North Africa & Middle East	1.14 (1.06–1.23)		1.08 (1.03–1.14)
South Asia	1.14 (1.06–1.22)		1.13 (1.07–1.19)
South-East Asia, East Asia & Oceania	1.04 (0.96–1.12)		1.03 (0.97–1.09)
Sub-Saharan Africa	1.00 (0.93–1.08)		1.01 (0.96–1.06)
* Overall*	1.03 (0.96–1.11)		1.04 (0.99–1.09)

*95%CI* 95% confidence interval, *IPV* intimate partner violence, *OR* odds ratio, *VAW* violence against women

^a^The adjustment factors for past year severe IPV is based on the analyses of microdata of *Demographic and Health Surveys* (DHS) where the definition of severe physical and/or sexual violence includes punching, kicking/dragging, trying to strangle/burn, threatening with a weapon, attacking with weapon, and any type of sexual violence

^b^Matching for past year IPV for the population surveyed (all women) did not result in enough matches. The OR for lifetime IPV are used instead as adjustment factors in the regression

The meta-analyses indicate that, overall, the odds of having experienced IPV for women asked about their experience of only severe violence are 62% (lifetime) and 43% (past year) lower than if these women had reported on IPV for all severity levels (Table 3). Examining results by super-regions, the contribution of severe violence to IPV (as indicated by the highest odds ratios for these regions) is relatively more important in *Latin America & the Caribbean*, followed by *Sub-Saharan Africa*, for both lifetime and past year IPV.

When surveys report “*physical IPV only*”, the discrepancy with “*physical and/or sexual IPV*” estimates is greatest in *South East Asia, East Asia & Oceania,* as well as *South Asia* and *Sub-Saharan Africa* for lifetime IPV (Table 3). In addition to those super-regions, the *High Income* region also exhibited high discrepancy for past year IPV. For both lifetime and past year IPV, the odds of reporting IPV are reduced by 14–19% when women were asked about physical IPV only.

The surveys’ denominators impact IPV prevalence (i.e., those eligible to answer the IPV questions). Overall, the odds of reporting IPV –when only currently partnered women were surveyed– were reduced by 9% for lifetime IPV and 1% for past year IPV, respectively, as compared to observations where all ever-partnered women were included (Table 3). When the surveys’ denominator included all women, and not only those that have ever been partnered, the odds of experiencing IPV was lower since a certain proportion of women, namely never-married/partnered women, will not have been exposed to the risk of IPV. Since there were no surveys of past year IPV that could be matched and compared, the odds ratios for lifetime IPV were used to adjust the studies that surveyed all women for past year IPV.

Some surveys measured IPV perpetrated by the current and/or most recent partner, while others referred to the current and/or all previous partners. Overall, the odds of reporting IPV was 16% lower for lifetime IPV if the question refers to the current or most recent partner compared to any current or previous partner. For past year IPV, the difference was much smaller: a 3% reduction in the odds of reporting IPV (Table 3).

Our results also showed marked regional variations in IPV prevalence by urban and rural areas (Table 3). IPV is higher in urban areas of *Latin America & Caribbean* as compared to the national average for both lifetime and past year IPV. The opposite pattern of higher IPV prevalence in rural areas is noted for all other regions. That difference is salient for *North Africa & Middle East* and *South Asia*.

### Model validation

The full graphical posterior predictive checks for lifetime IPV (Figure S16) and past year IPV (Figure S17) are presented as supplementary materials for all regions. As a representative example, the graphical posterior checks for the *Western* region of *Sub-Saharan Africa* with both the observed age-specific prevalence estimates and the model predictions are presented in Fig. 3. Overall, we found that the model fits the data well and that differences between data and model predictions, if any, were usually small and well within the uncertainty intervals of the prevalence estimates and model predictions. Further, in-sample comparisons suggested that prediction errors were very close to the expected null values indicating good fit with the empirical data and excellent coverage of uncertainty interval (Table 4). We also explored how those in-sample comparisons metrics varied by GBD regions and time periods (2000–04, 2005–09, 2010–14, 2015–18). These analyses revealed that median errors were smaller than 1% for all regions and time periods.

**Table 4:** In-sample comparisons of model fits with empirical data

VAW outcomes (Nb. observations)	Median (in % point)		Outside 95% CrI	
	**Error**	**Absolute error**	**Below (%)**	**Above (%)**
Lifetime IPV (1,551)	0.0%	1.5%	1.3%	1.3%
Past year IPV (1,598)	0.0%	1.0%	2.2%	1.9%

*95%CrI* 95% credible interval, *IPV* intimate partner violence

Comparisons defined as “*error* = *observed – predicted*”

Our out-of-sample comparisons, where we excluded 20% of countries and compared the known-but-excluded country-level observations with model predictions, were also in accordance with empirical estimates for both lifetime and past year IPV prevalence: median errors were reasonably close to zero (Table 5). The median absolute error quantifies the typical magnitude of the predictions’ errors, regardless of their direction. It was less than 8% points for lifetime IPV and 4% points for past year IPV. The models’ predictions included the point estimates of the known-but-excluded observations close to 95% of the time (as expected) for the two IPV outcomes, suggesting that the model was appropriately propagating uncertainty.

**Table 5:** Out-of-sample comparisons of age-specific model-predicted prevalence in 20% of randomly excluded countries with the empirical observations from these countries (including territories and areas)

VAW outcomes (Nb. countries excluded)	Median (in % point)		Outside 95% CrI	
	**Error**	**Absolute error**	**Below (%)**	**Above (%)**
Lifetime IPV (30)	1.3%	7.6%	1.4%	1.0%
Past year IPV (31)	0.6%	3.8%	1.9%	1.6%

*95%CrI* 95% credible interval, *IPV* intimate partner violence

Comparisons defined as “*error* = *observed – predicted*”. To improve stability of the metrics used for out-of-sample comparison, the process was repeated 20 times and the median estimates are presented above

Similarly, we tested the model’s ability to predict new surveys by excluding 20% of studies (instead of countries, as above). Here again, the model’s predictions were in accordance with the known-but-excluded survey estimates with small median and absolute errors and appropriate coverage of uncertainty intervals (Table 6).

**Table 6:** Out-of-sample comparisons of age-specific model-predicted prevalence in 20% of randomly excluded studies with the empirical observations from these studies

VAW outcomes (Nb. surveys excluded)	Median (in % point)		Outside 95% CrI	
	**Error**	**Absolute error**	**Below (%)**	**Above (%)**
Lifetime IPV (61)	0.3%	6.6%	2.2%	1.6%
Past year IPV (66)	0.4%	3.1%	2.6%	2.6%

*95%CrI* 95% credible interval, *IPV* intimate partner violence

Comparisons defined as “*error* = *observed – predicted*”. To improve stability of the metrics used for out-of-sample comparison, the process was repeated 20 times and the median estimates are presented above

## Discussion

Elimination of all forms of VAW must remain a global priority. Reliable, accurate, and comparable VAW statistics are essential to monitor progress towards this goal. This paper describes a framework for modeling global, regional, and national estimates of IPV statistics that specifically addresses key data and measurement issues [5, 8]. Globally, it is estimated that 27% of ever-married/partnered women (15–49 years old) have experienced physical and/or sexual IPV at least once in their lifetime and 13% experienced it within the past 12 months [40, 41]. There were regional variations in prevalence of both lifetime and past year IPV, with variations between high-income and low- and middle-income countries being particularly stark in relation to recent, that is past year prevalence. Estimated prevalence of IPV was highest in *Oceania* and *Central Sub-Saharan Africa* [40, 41]. The detailed region-, country-, and age-specific results have been reported and interpreted elsewhere [40, 41]. Drawing on well-established research, several social, economic, and political contextual factors could potentially explain these wide variations in the prevalence of IPV, including inequitable gender norms, economic insecurity, societal stigma, discriminatory laws and policies and political conflict [41].

The current modeling framework extends the previously developed methodology [3, 30] and is characterized by its ability to pool both nationally and sub-nationally representative population surveys, account for heterogeneous age groups and age trends, accommodate different surveyed populations, adjust for differences in survey instruments, and efficiently propagate model uncertainty to model outputs. The proposed framework is especially flexible. For example, both age trends and adjustment factors can vary by regions. For the latter, we used a robust identification strategy to estimate adjustments through exact matching of observations by survey identifier, whenever possible. We find that the relative contribution of physical IPV only to lifetime IPV is highest in the *Central Europe, Eastern Europe & Central Asia* and *North Africa & Middle East* super regions. As for the population surveyed, the inclusion of different groups of women according to partnership status influences prevalence estimates, especially for lifetime IPV, with ever-partnered women consistently providing higher prevalence than when surveying all women. Our estimation of adjustment factors offers interesting insights into the epidemiology of IPV by rural/urban areas. IPV is more common in rural areas in all regions, except in *Latin America & Caribbean*.

Our results should be interpreted considering some limitations. First, and most importantly, IPV statistics are based on women’s self-reports. IPV is a sensitive topic, and it is likely that some violence survivors under-report their experiences for a variety of reasons. However, we only included surveys that used act-specific questions: questions that are recognized for their ability to elicit more disclosure and accurate reports. Studies comparing men and women reports of past-year male-perpetrated IPV concluded that IPV indicators such as the ones included in this study are “reasonably reliable” [22]. As evidence accrues on the sensitivity and specificity of different methods for measuring IPV, prevalence estimates could be adjusted, if warranted, for the imperfect nature of those survey instruments.

A second limitation is that our analyses of IPV do not include psychological IPV, a frequent form of partner violence that can manifest in different ways [42]. Examples of acts included within the measure of psychological IPV include insults, humiliation, intimidation, and threats [43]. However, the diversity of acts measured in surveys, from engendering fear, to verbal abuse, enforcing social isolation, or otherwise the inclusion of economic abuse or controlling behaviors in those measures of psychological IPV has impeded the adoption of standardized and comparable measurements of psychological IPV [43]. This is an active research area and WHO has convened several expert meetings on this specific topic over the last years and is currently doing empirical analyses of existing data. The current lack of consensus on measures and thresholds nevertheless hampered the inclusion of psychological IPV in our current estimates.

A third limitation is that some populous countries have yet to conduct recent VAW surveys. The absence of national level data on the burden of violence is a significant challenge for establishing the magnitude and patterns of IPV. This evidence is crucial to advocating for prioritization of investments in national and local policies and programs aimed at eliminating IPV and other forms of VAW. Having said this, most of the world’s most populous countries have conducted such surveys in the last 18 years (Bangladesh, Brazil, India, Indonesia, Mexico, Nigeria, Pakistan, and the United States) such that more than 90% of the world’s women and girls reside in a country with at least one survey data point for both lifetime and past year IPV.

## Conclusion

We have described to reproducible levels of detail a flexible modeling framework to estimate global, regional, and national prevalence of IPV, with reassuring results from both in-sample and out-of-sample comparisons. Following proposed best reporting practice [44], the information provided in this study will support the accurate interpretation and responsible use of IPV statistics for informing national and international VAW prevention interventions and policies, and the monitoring efforts towards the elimination of VAW as part of the SDG agenda.

## Supplementary Information

**Additional file 1.** 

## Data Availability

The code and data are available from a public repository (https://github.com/pop-health-mod/vawstats-release). The list of studies is presented in the references list in the supplementary materials.

## Abbreviations

DHS: Demographic and Health Survey
GBD: Global Burden of Disease
IPV: Intimate Partner Violence
SDG: Sustainable Development Goals
VAW: Violence Against Women
