# Supplementary material for: A framework to model global, regional, and national estimates of intimate partner violence
Source: BMC Med Res Methodol. 2022 Jun 1;22:159. doi: 10.1186/s12874-022-01634-5 (PMC9158349; doi:10.1186/s12874-022-01634-5)
Supplement: Supplementary file 1 — Additional file 1. [file 12874_2022_1634_MOESM1_ESM.pdf]

Appendix - A framework for modeling global, regional, and national estimates of violence against women statistics

Table of Contents

Table S1: Posterior estimates of the Bayesian model of intimate partner violence main random effect estimates (median and 95% credible intervals). 2
Figure S1. Forest plot of the random effect meta-analysis for severe lifetime intimate partner violence (IPV), as compared to violence from all severity levels. 3
Figure S2. Forest plot of the random effect meta-analysis for severe past year intimate partner violence (IPV), as compared to violence from all severity levels. 4
Figure S3. Forest plot of the random effect meta-analysis for lifetime physical intimate partner violence (IPV) only, as compared to physical and/or sexual violence. 5
Figure S4. Forest plot of the random effect meta-analysis for past year physical intimate partner violence (IPV) only, as compared to physical and/or sexual violence. 6
Figure S5. Forest plot of the random effect meta-analysis for lifetime sexual intimate partner violence (IPV) only, as compared to physical and/or sexual violence. 7
Figure S6. Forest plot of the random effect meta-analysis for past year for sexual intimate partner violence (IPV) only, as compared to physical and/or sexual violence. 8
Figure S7. Forest plot of the random effect meta-analysis for lifetime intimate partner violence (IPV) when all women are surveyed, as compared to ever-partnered women. 9
Figure S8. Forest plot of the random effect meta-analysis for lifetime intimate partner violence (IPV) when currently-partnered women are surveyed, as compared to ever-partnered women. 10
Figure S9. Forest plot of the random effect meta-analysis for past year intimate partner violence (IPV) when currently partnered women are surveyed, as compared to ever-partnered women. 11
Figure S10. Forest plot of the random effect meta-analysis for lifetime intimate partner violence (IPV) when the reference partner is the current or most recent one, as compared to any current or previous ones. 12
Figure S11. Forest plot of the random effect meta-analysis for past year intimate partner violence (IPV) when the reference partner is the current or most recent one, as compared to any current or previous ones. 13
Figure S12. Plot of the data used to estimate, through random-effect logistic regression, the adjustment factor for lifetime intimate partner violence (IPV) in urban regions, as compared to a nationally representative sample. 14
Figure S13. Plot of the data used to estimate, through random-effect logistic regression, the adjustment factor for past year intimate partner violence (IPV) in urban regions, as compared to a nationally representative sample. 15
Figure S14. Plot of the data used to estimate, through random-effect logistic regression, the adjustment factor for lifetime intimate partner violence (IPV) in rural regions, as compared to a nationally representative sample. 16
Figure S15. Plot of the data used to estimate, through random-effect logistic regression, the adjustment factor for past year intimate partner violence (IPV) in rural regions, as compared to a nationally representative sample. 17
Figure S16. Posterior predictive checks for the lifetime intimate partner violence (IPV) model. 18
Figure S17. Posterior predictive checks for the past year intimate partner violence (IPV) model. 19

(Note that the effective sample sizes are used. This can explain the slight inequalities between the denominators.)

Table S1: Posterior estimates of the Bayesian model of intimate partner violence main random effect estimates (median and 95% credible intervals).

Posterior estimates of the standard deviations of the random effects	Lifetime IPV	Past year IPV
Study level (sub-national survey)	0.86 (0.68 – 1.12)	0.83 (0.64 – 1.12)
Study level (nationally representative survey)	0.39 (0.34 – 0.45)	0.42 (0.37 – 0.49)
Country level	0.36 (0.20 – 0.46)	0.40 (0.23 – 0.51)
Region level	0.37 (0.18 – 0.63)	0.41 (0.19 – 0.71)
Super region level	0.36 (0.03 – 1.03)	0.46 (0.06 – 1.25)

IPV: intimate partner violence.

Figure S1. Forest plot of the random effect meta-analysis for severe lifetime intimate partner violence (IPV), as compared to violence from all severity levels.

Source	OR (95% CI)
**SuperRegion = High Income**	
Japan – (Yoshihama et al., 2005)	0.24 [0.16; 0.35]
Total	0.24 [0.16; 0.35]
Heterogeneity: not applicable	
**SuperRegion = Central Europe, Eastern Europe & Central Asia**	
Moldova – (NCPM/Moldova, and ORC Macro, 2006)	0.39 [0.34; 0.46]
Serbia – (Otasevic et al., 2005)	0.30 [0.22; 0.40]
Total	0.35 [0.27; 0.46]
Heterogeneity: $\chi^2_1 = 2.57$ ($P = .11$), $I^2 = 61\%$	
**SuperRegion = South–East Asia, East Asia & Oceania**	
Cambodia – (NIS/Cambodia, DGH/Cambodia, and ORC Macro, 2001)	0.53 [0.42; 0.69]
Cambodia – (NIPH/Cambodia, NIS/Cambodia, and ORC Macro, 2006)	0.49 [0.36; 0.66]
Thailand – (Kanchanachitra et al., 2005)	0.22 [0.17; 0.29]
Thailand – (Kanchanachitra et al., 2005)	0.25 [0.19; 0.32]
Samoa – (Posiulai et al., 2005)	0.40 [0.32; 0.50]
Total	0.36 [0.25; 0.50]
Heterogeneity: $\chi^2_4 = 35.39$ ($P < .001$), $I^2 = 89\%$	
**SuperRegion = South Asia**	
Bangladesh – (Garcia–Moreno et al., 2005)	0.22 [0.17; 0.27]
Bangladesh – (Garcia–Moreno et al., 2005)	0.17 [0.14; 0.21]
India – (IIPS/India, and Macro International, 2007)	0.35 [0.34; 0.37]
Total	0.24 [0.14; 0.39]
Heterogeneity: $\chi^2_2 = 57.49$ ($P < .001$), $I^2 = 97\%$	
**SuperRegion = Latin America & Caribbean**	
Bolivia – (Gutiérrez, 2004)	0.66 [0.61; 0.70]
Brazil – (Schraiber et al., 2005)	0.49 [0.37; 0.65]
Brazil – (Schraiber et al., 2005)	0.48 [0.38; 0.60]
Colombia – (Profamilia/Colombia, 2000)	0.41 [0.37; 0.45]
Dominican Republic – (Achécar et al., 2003)	0.61 [0.53; 0.71]
Dominican Republic – (CESDEM/Republica Dominicana and Macro International, 2008)	0.55 [0.47; 0.65]
Haïti – (Cayemittes et al., 2001)	0.51 [0.39; 0.66]
Haïti – (Cayemittes et al., 2007)	0.39 [0.31; 0.49]
Total	0.51 [0.43; 0.60]
Heterogeneity: $\chi^2_7 = 79.06$ ($P < .001$), $I^2 = 91\%$	
**SuperRegion = North Africa & Middle East**	
Egypt – (MHP/Egypt, and NNPC/Egypt, 2006)	0.33 [0.29; 0.37]
Jordan – (DoS/Jordan, and Macro International, 2008)	0.39 [0.31; 0.49]
Total	0.35 [0.29; 0.42]
Heterogeneity: $\chi^2_1 = 2.01$ ($P = .16$), $I^2 = 50\%$	
**SuperRegion = Sub–Saharan Africa**	
Cameroon – (INS/Cameroun, and ORC Macro, 2005)	0.37 [0.31; 0.44]
Ethiopia – (Berhane et al., 2005)	0.23 [0.20; 0.27]
Liberia – (LISGIS/Liberia, MOHSW/Liberia, NACP/Liberia, and Macro International, 2008)	0.41 [0.34; 0.50]
Malawi – (NSO/Malawi, and ORC Macro, 2005)	0.42 [0.37; 0.47]
Namibia – (January et al., 2005)	0.48 [0.38; 0.59]
Rwanda – (INSR/Rwanda, and ORC Macro, 2006)	0.43 [0.36; 0.51]
Tanzania – (MoHCDGEC/Tanzania Mainland, MoH/Zanzibar, NBS/Tanzania, OCGS/Zanzibar, and ICF, 2016)	0.30 [0.24; 0.37]
Tanzania – (MoHCDGEC/Tanzania Mainland, MoH/Zanzibar, NBS/Tanzania, OCGS/Zanzibar, and ICF, 2016)	0.31 [0.25; 0.39]
Uganda – (UBOS/Uganda, and Macro International, 2007)	0.45 [0.37; 0.55]
Zambia – (CSO/Zambia, MOH/Zambia, TDRC/Zambia, and University of Zambia, 2009)	0.32 [0.28; 0.37]
Zimbabwe – (CSO/Zimbabwe, and Macro International, 2007)	0.45 [0.39; 0.51]
Total	0.37 [0.33; 0.42]
Heterogeneity: $\chi^2_{10} = 64.99$ ($P < .001$), $I^2 = 85\%$	
Total	0.38 [0.34; 0.42]
Heterogeneity: $\chi^2_{31} = 511.19$ ($P < .001$), $I^2 = 94\%$	
Residual heterogeneity: $\chi^2_{25} = 241.52$ ($P < .001$), $I^2 = 90\%$	

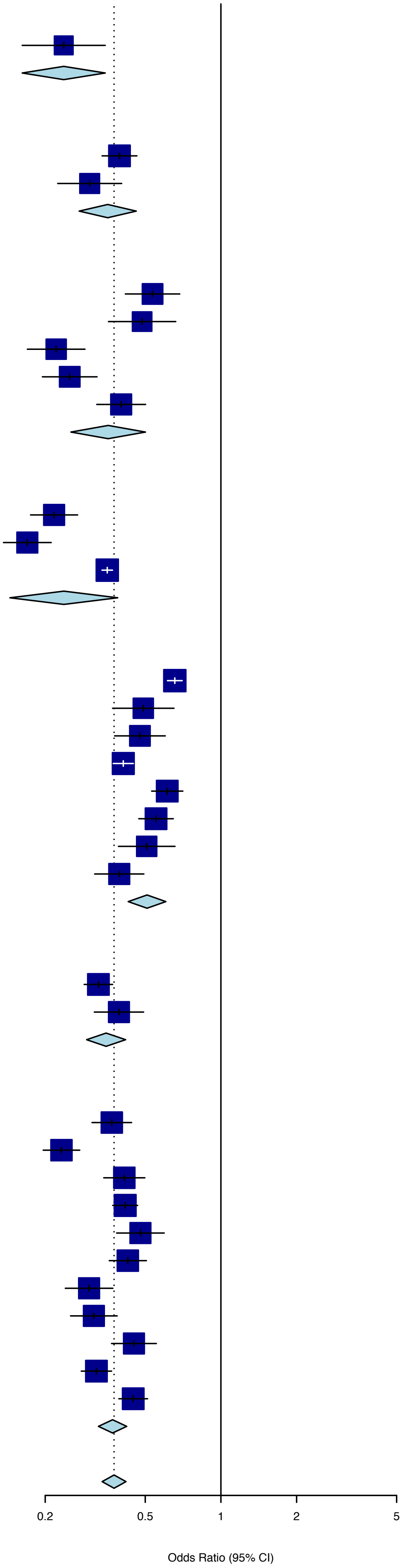

Figure S2. Forest plot of the random effect meta-analysis for severe past year intimate partner violence (IPV), as compared to violence from all severity levels.

Source	OR (95% CI)
**SuperRegion = North Africa & Middle East**	
Afghanistan – (CSO/Afghanistan, MPH/Afghanistan, and ICF, 2017)	0.32 [0.31; 0.34]
Egypt – (MHP/Egypt and ICF International, 2015)	0.37 [0.33; 0.42]
Jordan – (DoS/Jordan, and Macro International, 2008)	0.62 [0.53; 0.72]
Jordan – (DoS/Jordan, and ICF International, 2013)	0.64 [0.58; 0.71]
Jordan – (DoS/Jordan, and ICF, 2019)	0.48 [0.43; 0.54]
Total	0.47 [0.34; 0.64]
Heterogeneity: $\chi^2_4 = 214.06$ ($P < .001$), $I^2 = 98\%$	
**SuperRegion = Sub–Saharan Africa**	
Angola – (INE/Angola, MINSA/Angola, and ICF, 2017)	0.52 [0.48; 0.56]
Burundi – (DHS, 2016)	0.75 [0.69; 0.81]
Benin – (INSAE/Bénin, and ICF, 2019)	0.61 [0.54; 0.71]
Burkina Faso – (DHS, 2010)	0.53 [0.47; 0.59]
Cote d'Ivoire – (DHS, 2012)	0.54 [0.49; 0.61]
Cameroon – (INS/Cameroun, and ORC Macro, 2005)	0.56 [0.50; 0.63]
Cameroon – (INS/Cameroun, and ICF International, 2012)	0.51 [0.46; 0.56]
Democratic Republic of the Congo – (MP/Congo, and Macro International, 2008)	0.60 [0.55; 0.67]
Democratic Republic of the Congo – (MPSMRM/Congo, MSP/Congo, and ICF International, 2014)	0.57 [0.52; 0.62]
Comoros – (DGSP/Comores, and ICF International, 2014)	0.44 [0.31; 0.62]
Ethiopia – (CSA/Ethiopia, and ICF, 2016)	0.61 [0.54; 0.68]
Gabon – (DGS/Gabon, and ICF International, 2013)	0.59 [0.53; 0.65]
Ghana – (GSS, GHS, and Macro International, 2009)	0.58 [0.48; 0.70]
Gambia – (GBOS, and ICF International, 2014)	0.60 [0.49; 0.74]
Kenya – (CBS/Kenya, MOH/Kenya, and ORC Macro, 2004)	0.60 [0.55; 0.65]
Kenya – (KNBS/Kenya, NACC/Kenya, NASCP/Kenya, MPHS/Kenya, and KMRI, 2010)	0.62 [0.57; 0.68]
Kenya – (KNBS/Kenya, MOH/Kenya, NACC/Kenya, KMRI/Kenya, NCPD/Kenya, 2015)	0.62 [0.56; 0.69]
Liberia – (LISGIS/Liberia, MOHSW/Liberia, NACP/Liberia, and Macro International, 2008)	0.56 [0.51; 0.62]
Mali – (Samaké et al., 2007)	0.59 [0.54; 0.64]
Mali – (CPS/SSDSPF/Mali, INSTAT/Mali, INFO–STAT/Mali, and ICF International, 2014)	0.54 [0.47; 0.61]
Mali – (INSTAT/Mali, CPSSSD, and ICF, 2019)	0.55 [0.48; 0.63]
Moçambique – (MISAU/Moçambique, INE/Moçambique, and ICF International, 2013)	0.50 [0.45; 0.55]
Moçambique – (INS, INE, PNCM/Moçambique and ICF, 2019)	0.53 [0.45; 0.62]
Malawi – (NSO/Malawi, and ORC Macro, 2005)	0.69 [0.64; 0.74]
Malawi – (NSO/Malawi, and ICF Macro, 2011)	0.77 [0.70; 0.85]
Malawi – (NSO/Malawi, and ICF, 2017)	0.74 [0.68; 0.82]
Namibia – (MoHSS/Namibia, and ICF International, 2014)	0.71 [0.58; 0.88]
Nigeria – (NPC/Nigeria, and ICF Macro, 2009)	0.48 [0.45; 0.51]
Nigeria – (NPC/Nigeria, and ICF International, 2014)	0.58 [0.54; 0.63]
Nigeria – (NPC/Nigeria, and ICF, 2019)	0.60 [0.54; 0.66]
Rwanda – (INSR/Rwanda, and ORC Macro, 2006)	0.58 [0.51; 0.65]
Rwanda – (NISR/Rwanda, MOH/Rwanda, and ICF International, 2012)	0.95 [0.87; 1.04]
Rwanda – (DHS, 2015)	0.65 [0.55; 0.78]
Senegal – (ANSD/Sénégal, and ICF, 2018)	0.66 [0.54; 0.79]
Sierra Leone – (SSL/Sierra Leone, and ICF International, 2014)	0.50 [0.45; 0.56]
Sao Tome and Principe – (INE/São Tomé, and ICF Macro, 2010)	0.54 [0.45; 0.64]
Chad – (INSEED/Tchad, MSP/Tchad, and ICF International, 2016)	0.64 [0.56; 0.73]
Togo – (MPDAT/Togo, MS/Togo, and ICF International, 2015)	0.67 [0.59; 0.76]
Tanzania – (NBS/Tanzania, and ICF Macro, 2011)	0.59 [0.55; 0.64]
Tanzania – (MoHCDGEC/Tanzania, MoH/Zanzibar, NBS/Tanzania, OCGS/Zanzibar, and ICF, 2016)	0.65 [0.60; 0.70]
Uganda – (UBOS/Uganda, and Macro International, 2007)	0.75 [0.66; 0.86]
Uganda – (DHS, 2011)	0.76 [0.65; 0.88]
Uganda – (UBOS/Uganda, and ICF, 2018)	0.74 [0.69; 0.80]
South Africa – (NDH/South Africa, and ICF, 2019)	0.63 [0.54; 0.75]
Zambia – (CSO/Zambia, MOH/Zambia, TDRC/Zambia, and University of Zambia, 2009)	0.49 [0.45; 0.54]
Zambia – (CSO/Zambia, MOH/Zambia, University of Zambia THVL, DPS, TDRC/Zambia, and ICF International, 2015)	0.65 [0.61; 0.70]
Zimbabwe – (CSO/Zimbabwe, and Macro International, 2007)	0.65 [0.59; 0.71]
Zimbabwe – (ZIMSTAT/Zimbabwe, and ICF International, 2012)	0.65 [0.60; 0.72]
Zimbabwe – (ZNSA/Zimbabwe, and ICF International, 2016)	0.64 [0.57; 0.70]
Total	0.61 [0.58; 0.64]
Heterogeneity: $\chi^2_{48} = 399.83$ ($P < .001$), $I^2 = 88\%$	
**SuperRegion = Central Europe, Eastern Europe & Central Asia**	
Armenia – (DHS, 2016)	0.27 [0.18; 0.40]
Azerbaijan – (SSC/Azerbaijan, and Macro International, 2008)	0.44 [0.37; 0.53]
Kyrgyz Republic – (NSC, MOH/Kyrgyz Republic NSC, and ICF International, 2013)	0.41 [0.36; 0.47]
Moldova – (NCPM/Moldova, and ORC Macro, 2006)	0.47 [0.42; 0.52]
Tajikistan – (SA/Tajikistan, MOH/Tajikistan, and ICF International, 2013)	0.39 [0.33; 0.45]
Tajikistan – (SA, MOH/Tajikistan, and ICF, 2018)	0.31 [0.27; 0.35]
Ukraine – (UCSR, SSC/Ukraine, MOH/Ukraine, and Macro International, 2008)	0.46 [0.37; 0.58]
Total	0.39 [0.34; 0.45]
Heterogeneity: $\chi^2_6 = 29.56$ ($P < .001$), $I^2 = 80\%$	
**SuperRegion = South Asia**	
Bangladesh – (NIPORT/Bangladesh, Mitra, Associates/Bangladesh, and Macro International, 2009)	0.60 [0.54; 0.67]
India – (DHS, 2006)	0.50 [0.49; 0.52]
Nepal – (MOHP/Nepal, New ERA/Nepal, and ICF International, 2012)	0.72 [0.62; 0.83]
Nepal – (MOH/Nepal, New ERA/Nepal, and ICF, 2017)	0.59 [0.50; 0.69]
Pakistan – (NIPS/Pakistan, and ICF International, 2013)	0.32 [0.28; 0.38]
Pakistan – (NIPS/Pakistan, and ICF, 2019)	0.45 [0.38; 0.53]
Total	0.52 [0.44; 0.61]
Heterogeneity: $\chi^2_5 = 69.72$ ($P < .001$), $I^2 = 93\%$	
**SuperRegion = Latin America & Caribbean**	
Colombia – (DHS, 2005)	0.50 [0.48; 0.52]
Colombia – (Profamilia/Colombia, 2005)	0.46 [0.45; 0.48]
Colombia – (MSPSP, 2015)	0.46 [0.44; 0.48]
Dominican Republic – (Achécar et al., 2003)	0.68 [0.62; 0.74]
Dominican Republic – (CESDEM/Republica Dominicana and Macro International, 2008)	0.64 [0.58; 0.72]
Dominican Republic – (CESDEM/Republica Dominicana, and ICF International, 2014)	0.56 [0.50; 0.63]
Guatemala – (DHS, 2015)	0.57 [0.49; 0.66]
Honduras – (SS/Honduras, INE/Honduras, and ICF International, 2013)	0.61 [0.56; 0.67]
Haïti – (Cayemittes et al., 2001)	0.86 [0.76; 0.97]
Haiti – (DHS, 2006)	0.77 [0.66; 0.89]
Haïti – (Cayemittes et al., 2013)	0.80 [0.72; 0.89]
Haïti – (IHE/Haïti, and ICF, 2018)	0.77 [0.67; 0.88]
Total	0.62 [0.56; 0.69]
Heterogeneity: $\chi^2_{11} = 332.1$ ($P < .001$), $I^2 = 97\%$	
**SuperRegion = South–East Asia, East Asia & Oceania**	
Cambodia – (NIS/Cambodia, DGH/Cambodia, and ORC Macro, 2001)	0.60 [0.51; 0.71]
Cambodia – (NIPH/Cambodia, NIS/Cambodia, and ORC Macro, 2006)	0.64 [0.50; 0.81]
Cambodia – (NIS/Cambodia, DGH/Cambodia, and ICF International, 2015)	0.67 [0.56; 0.79]
Maldives – (DHS, 2016)	0.52 [0.40; 0.67]
Myanmar – (DHS, 2016)	0.54 [0.45; 0.65]
Philippines – (NSO/Philippines, and ICF Macro, 2009)	0.66 [0.58; 0.74]
Philippines – (PSA/Philippines, and ICF International, 2014)	0.71 [0.62; 0.82]
Philippines – (PSA/Philippines, and ICF, 2018)	0.64 [0.56; 0.73]
Papua New Guinea – (DHS, 2017)	0.74 [0.68; 0.80]
Timor–leste – (NSD/Timor–Leste, MF/Timor–Leste, and ICF Macro, 2010)	0.30 [0.26; 0.36]
Timor–leste – (GDS, MF/Timor Leste, and ICF, 2018)	0.41 [0.36; 0.45]
Total	0.57 [0.48; 0.67]
Heterogeneity: $\chi^2_{10} = 147.68$ ($P < .001$), $I^2 = 93\%$	
Total	0.57 [0.55; 0.60]

Heterogeneity: $\chi^2_{89} = 2056.92$ ($P < .001$), $I^2 = 96\%$

Residual heterogeneity: $\chi^2_{84} = 1192.96$ ($P < .001$), $I^2 = 93\%$

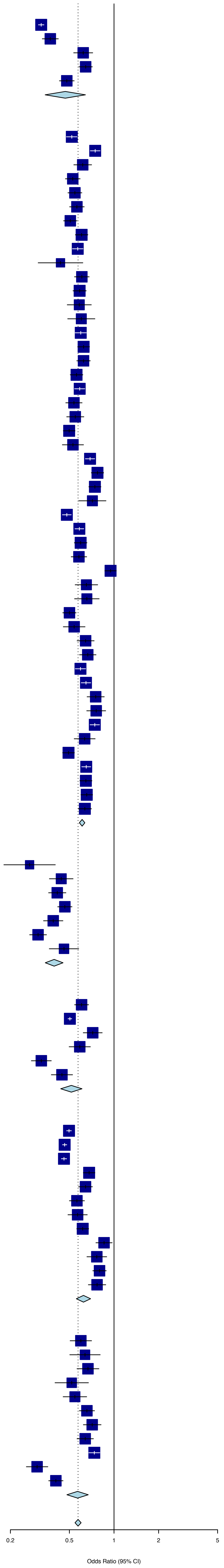

Figure S3. Forest plot of the random effect meta-analysis for lifetime physical intimate partner violence (IPV) only, as compared to physical and/or sexual violence.

Source	OR (95% CI)
**SuperRegion = High Income**	
Argentina – (Ministerio de Justicia y Derechos Humanos Presidencia de la Nacion, 2017)	0.98 [0.74; 1.30]
Argentina – (Ministerio de Justicia y Derechos Humanos Presidencia de la Nacion, 2017)	0.94 [0.71; 1.24]
Australia – (Mouzos and Makai, 2004)	0.87 [0.78; 0.98]
Australia – (Personal Safety Survey, 2017)	0.93 [0.76; 1.14]
Austria – (European Union Agency for Fundamental Rights, 2014)	0.94 [0.66; 1.34]
Belgium – (European Union Agency for Fundamental Rights, 2014)	0.91 [0.68; 1.22]
Cyprus – (European Union Agency for Fundamental Rights, 2014)	0.96 [0.70; 1.33]
Germany – (Schrottle et al., 2004)	0.96 [0.83; 1.11]
Germany – (European Union Agency for Fundamental Rights, 2014)	0.88 [0.66; 1.16]
Denmark – (European Union Agency for Fundamental Rights, 2014)	0.85 [0.68; 1.07]
Spain – (Ministerio de Sanidad, Servicios Sociales e Igualdad, 2015)	0.80 [0.70; 0.92]
Finland – (European Union Agency for Fundamental Rights, 2014)	0.86 [0.68; 1.09]
France – (European Union Agency for Fundamental Rights, 2014)	0.93 [0.74; 1.17]
United Kingdom – (European Union Agency for Fundamental Rights, 2014)	0.93 [0.71; 1.23]
Greece – (European Union Agency for Fundamental Rights, 2014)	0.92 [0.69; 1.24]
Ireland – (European Union Agency for Fundamental Rights, 2014)	0.93 [0.68; 1.27]
Iceland – (Gíslason, 2009)	0.85 [0.70; 1.03]
Italy – (Istat, 2006)	0.82 [0.75; 0.89]
Italy – (European Union Agency for Fundamental Rights, 2014)	0.89 [0.60; 1.33]
Italy – (Istat, 2015)	0.83 [0.77; 0.91]
Japan – (Yoshihama et al., 2005)	0.81 [0.57; 1.15]
Luxembourg – (European Union Agency for Fundamental Rights, 2014)	0.92 [0.68; 1.26]
Malta – (Commission on Domestic Violence, 2011)	0.71 [0.49; 1.03]
Malta – (European Union Agency for Fundamental Rights, 2014)	0.89 [0.63; 1.26]
Netherlands – (European Union Agency for Fundamental Rights, 2014)	0.83 [0.65; 1.07]
New Zealand – (Fanslow et al., 2004)	0.83 [0.69; 0.98]
Portugal – (European Union Agency for Fundamental Rights, 2014)	0.93 [0.70; 1.24]
Singapore – (Bouhours et al., 2013)	0.95 [0.59; 1.50]
Sweden – (European Union Agency for Fundamental Rights, 2014)	0.80 [0.61; 1.05]
Total	0.86 [0.84; 0.88]
Heterogeneity: $\chi^2_{28} = 11.46$ ($P > .99$), $I^2 = 0\%$	
**SuperRegion = Central Europe, Eastern Europe & Central Asia**	
Albania – (Haarr et al., 2013)	0.95 [0.80; 1.13]
Albania – (The Institut of Statistics of Albania ... Instat/Albania, 2018)	0.83 [0.68; 1.00]
Albania – (Organisation for Security and Conflict in Europe, 2019)	1.00 [0.76; 1.32]
Armenia – (National Statistical Service of the Republic of Armenia , 2011)	0.93 [0.68; 1.27]
Armenia – (National Statistical Service, 2017)	1.00 [0.75; 1.33]
Azerbaijan – (SSC/Azerbaijan, and Macro International, 2008)	0.94 [0.76; 1.16]
Bulgaria – (European Union Agency for Fundamental Rights, 2014)	0.92 [0.63; 1.34]
Bosnia and Hezergovina – (Gender Equality Agency of Bosnia and Herzegovina, 2013)	0.86 [0.68; 1.09]
Bosnia and Hezergovina – (Organisation for Security and Conflict in Europe, 2019)	1.00 [0.49; 2.03]
Bosnia and Hezergovina – (Organisation for Security and Conflict in Europe, 2019)	0.89 [0.45; 1.74]
Bosnia and Hezergovina – (Organisation for Security and Conflict in Europe, 2019)	1.00 [0.46; 2.17]
Bosnia and Hezergovina – (Organisation for Security and Conflict in Europe, 2019)	0.91 [0.49; 1.68]
Bosnia and Hezergovina – (Organisation for Security and Conflict in Europe, 2019)	0.89 [0.66; 1.22]
Bosnia and Hezergovina – (Organisation for Security and Conflict in Europe, 2019)	1.00 [0.50; 2.00]
Belarus – (Institute of Sociology of NAS Belarus, 2019)	0.93 [0.69; 1.24]
Czechia – (European Union Agency for Fundamental Rights, 2014)	0.90 [0.66; 1.22]
Czechia – (...Buriánek et al., 2013)	0.93 [0.69; 1.26]
Estonia – (European Union Agency for Fundamental Rights, 2014)	0.92 [0.71; 1.20]
Georgia – (UN Women/GEOSTAT, 2018)	0.91 [0.70; 1.19]
Croatia – (European Union Agency for Fundamental Rights, 2014)	0.90 [0.65; 1.27]
Hungary – (European Union Agency for Fundamental Rights, 2014)	0.87 [0.65; 1.17]
Kazakhstan – (Ministry of National Economy of the Republic of Kazakhstan, 2017)	0.98 [0.82; 1.16]
Kyrgyz Republic – (NSC, MOH/Kyrgyz Republic NSC, and ICF International, 2013)	0.99 [0.85; 1.15]
Lithuania – (Schrottle et al., 2003)	0.81 [0.57; 1.14]
Lithuania – (European Union Agency for Fundamental Rights, 2014)	1.01 [0.75; 1.37]
Latvia – (European Union Agency for Fundamental Rights, 2014)	0.96 [0.71; 1.30]
Moldova – (NCPM/Moldova, and ORC Macro, 2006)	0.98 [0.83; 1.14]
Moldova – (UNW, UNDP, and UNFPA, 2011)	0.79 [0.36; 1.72]
Moldova – (Organisation for Security and Conflict in Europe, 2019)	0.95 [0.76; 1.20]
North Macedonia – (Organisation for Security and Conflict in Europe, 2019)	0.89 [0.63; 1.27]
Montenegro – (Organisation for Security and Conflict in Europe, 2019)	1.00 [0.71; 1.42]
Mongolia – (NSO, UNFPA, SDC, Australian Aid, 2018)	0.93 [0.83; 1.05]
Poland – (European Union Agency for Fundamental Rights, 2014)	0.89 [0.64; 1.23]
Kosovo – (Organisation for Security and Conflict in Europe, 2019)	0.81 [0.57; 1.15]
Romania – (European Union Agency for Fundamental Rights, 2014)	0.96 [0.73; 1.28]
Serbia – (Otasevic et al., 2005)	0.95 [0.71; 1.29]
Serbia – (Organisation for Security and Conflict in Europe, 2019)	0.93 [0.67; 1.29]
Serbia – (Organisation for Security and Conflict in Europe, 2019)	0.92 [0.59; 1.45]
Slovakia – (European Union Agency for Fundamental Rights, 2014)	0.93 [0.66; 1.31]
Slovenia – (Institute of Criminology, Ministry of Interior and Faculty of Social Work, 2010)	0.74 [0.52; 1.07]
Slovenia – (European Union Agency for Fundamental Rights, 2014)	0.89 [0.65; 1.21]
Tajikistan – (SA/Tajikistan, MOH/Tajikistan, and ICF International, 2013)	0.95 [0.80; 1.13]
Tajikistan – (SA, MOH/Tajikistan, and ICF, 2018)	0.97 [0.85; 1.12]
Ukraine – (UCSR, SSC/Ukraine, MOH/Ukraine, and Macro International, 2008)	0.96 [0.74; 1.26]
Ukraine – (GfK Ukraine, 2014)	0.96 [0.68; 1.34]
Ukraine – (Organisation for Security and Conflict in Europe, 2019)	0.85 [0.68; 1.07]
Total	0.93 [0.92; 0.95]
Heterogeneity: $\chi^2_{45} = 8.94$ ($P > .99$), $I^2 = 0\%$	
**SuperRegion = South–East Asia, East Asia & Oceania**	
China – (Guo et al., 2004)	0.49 [0.43; 0.56]
Cook Islands – (Ministry of Health, 2014)	0.85 [0.61; 1.17]
Fiji – (Fiji Women's Crisis Centre, 2013)	0.88 [0.77; 1.01]
Micronesia – (Department of Health and Social Affairs, 2014)	0.85 [0.69; 1.04]
Hong Kong – (Bouhours et al., 2015)	0.61 [0.30; 1.23]
Indonesia – (UNDP, 2016)	0.78 [0.61; 1.01]
Cambodia – (NIS/Cambodia, DGH/Cambodia, and ORC Macro, 2001)	0.96 [0.75; 1.23]
Cambodia – (NIPH/Cambodia, NIS/Cambodia, and ORC Macro, 2006)	0.92 [0.69; 1.22]
Cambodia – (NIS/Cambodia, DGH/Cambodia, and ICF International, 2015)	0.88 [0.72; 1.07]
Cambodia – (Ministry of Women's Affairs, 2015)	0.67 [0.54; 0.83]
Kiribati – (Ministry of Internal and Social Affairs, 2010)	0.72 [0.59; 0.88]
Laos – (National Commission for the Advancement of Women, 2015)	0.72 [0.60; 0.88]
Maldives – (Ministry of Gender and Family, 2011)	0.90 [0.69; 1.18]
Maldives – (Ministry of Health Maldives, 2018)	0.95 [0.76; 1.18]
Marshall Islands – (DHS/Marshall Islands, 2008)	0.77 [0.54; 1.08]
Marshall Islands – (Ministry of Internal Affairs, 2014)	0.90 [0.67; 1.20]
Myanmar – (MoHS/Myanmar, and ICF, 2017)	0.93 [0.75; 1.15]
Nauru – (Ministry of Home Affairs, 2014)	0.93 [0.57; 1.51]
Philippines – (NSO/Philippines, and ICF Macro, 2009)	0.77 [0.67; 0.89]
Philippines – (PSA/Philippines, and ICF International, 2014)	0.85 [0.74; 0.98]
Philippines – (PSA/Philippines, and ICF, 2018)	0.90 [0.80; 1.01]
Palau – (Ministry of Health, 2014)	0.90 [0.67; 1.21]
Papua New Guinea – (NSO/Papua New Guinea, and ICF, 2019)	0.90 [0.78; 1.04]
Solomon Islands – (National Statistics Office, 2009)	0.48 [0.40; 0.57]
Thailand – (Kanchanachitra et al., 2005)	0.43 [0.32; 0.58]
Thailand – (Kanchanachitra et al., 2005)	0.57 [0.43; 0.76]
Timor–leste – (NSD/Timor–Leste, MF/Timor–Leste, and ICF Macro, 2010)	0.96 [0.78; 1.18]
Timor–Leste – (Asia Foundation, 2016)	0.66 [0.56; 0.79]
Timor–Leste – (General Directorate of Statistics, Ministry of Finance/Timor Leste, and ICF, 2018)	0.94 [0.81; 1.10]
Tonga – (Ma'a Fafine mo e Famili, 2012)	0.77 [0.50; 1.18]
Tuvalu – (CSDSPC, 2009)	0.86 [0.57; 1.29]
Vietnam – (Vung et al., 2008)	0.92 [0.70; 1.21]
Vietnam – (General Statistics Office of Vietnam, 2010)	0.88 [0.80; 0.96]
Vanuatu – (Vanuatu Women's Centre, 2011)	0.69 [0.57; 0.84]
Samoa – (Posiulai et al., 2005)	0.80 [0.62; 1.03]
Total	0.79 [0.73; 0.84]
Heterogeneity: $\chi^2_{34} = 155.53$ ($P < .001$), $I^2 = 78\%$	
**SuperRegion = South Asia**	
Bangladesh – (Garcia–Moreno et al., 2005)	0.45 [0.35; 0.57]
Bangladesh – (Garcia–Moreno et al., 2005)	0.58 [0.45; 0.73]
Bangladesh – (NIPORT/Bangladesh, Mitra, Associates/Bangladesh, and Macro International, 2009)	0.83 [0.73; 0.95]
Bangladesh – (Bangladesh Bureau of Statistics, 2016)	0.83 [0.78; 0.89]
Bhutan – (National Commission for Women and Children, 2012)	0.72 [0.42; 1.25]
Bhutan – (National Commission for Women and Children, 2019)	0.91 [0.71; 1.15]
India – (IIPS/India, and Macro International, 2007)	0.91 [0.88; 0.95]
India – (IIPS/India, and ICF, 2017)	0.95 [0.91; 0.99]
Nepal – (MOHP/Nepal, New ERA/Nepal, and ICF International, 2012)	0.76 [0.64; 0.91]
Nepal – (MOH/Nepal, New ERA/Nepal, and ICF, 2017)	0.93 [0.78; 1.10]
Pakistan – (NIPS/Pakistan, and ICF, 2019)	0.95 [0.80; 1.14]
Total	0.82 [0.71; 0.95]
Heterogeneity: $\chi^2_{10} = 64.95$ ($P < .001$), $I^2 = 85\%$	
**SuperRegion = Latin America & Caribbean**	
Belize – (Young Macfarlane Belize Institute for Local Development, 2015)	0.96 [0.60; 1.54]
Bolivia – (Gutiérrez, 2004)	0.96 [0.89; 1.03]
Bolivia – (Instituto Nacional de Estadística, 2017)	0.78 [0.70; 0.87]
Brazil – (Schraiber et al., 2005)	0.91 [0.66; 1.25]
Brazil – (Schraiber et al., 2005)	0.88 [0.67; 1.15]
Brazil – (Instituto de Pesquisa DataSenado, 2017)	0.96 [0.72; 1.27]
Colombia – (Profamilia/Colombia, 2000)	0.94 [0.85; 1.04]
Dominican Republic – (Achécar et al., 2003)	0.95 [0.83; 1.09]
Dominican Republic – (CESDEM/Republica Dominicana and Macro International, 2008)	0.92 [0.81; 1.06]
Dominican Republic – (CESDEM/Republica Dominicana, and ICF International, 2014)	0.94 [0.82; 1.08]
Grenada – (Nicholson and Deshong., 2018)	0.90 [0.70; 1.17]
Guatemala – (MSPAS, 2011)	0.85 [0.78; 0.93]
Guatemala – (MSPAS, INE, and Segeplán, 2017)	0.95 [0.82; 1.11]
Guyana – (Contreras–Urbina et al., 2019)	0.88 [0.69; 1.13]
Honduras – (SS/Honduras, INE/Honduras, and ICF International, 2013)	0.91 [0.83; 1.00]
Haïti – (Cayemittes et al., 2001)	0.58 [0.47; 0.73]
Haïti – (Cayemittes et al., 2007)	0.47 [0.37; 0.59]
Haïti – (Cayemittes et al., 2013)	0.75 [0.65; 0.86]
Haïti – (IHE/Haïti, and ICF, 2018)	0.77 [0.65; 0.91]
Jamaica – (National Family Planning Board, 2010)	0.85 [0.74; 0.97]
Jamaica – (Williams et al., 2018)	0.88 [0.64; 1.21]
Mexico – (Instituto National de Estadística Y Geografía, 2007)	0.86 [0.82; 0.89]
Mexico – (Instituto National de Estadística Y Geografía, 2016)	0.91 [0.87; 0.94]
Nicaragua – (INIDE MINSA, 2008)	0.89 [0.81; 0.98]
Nicaragua – (INIDE MINSA, 2014)	0.86 [0.78; 0.95]
Panama – (Instituto Conmemorativo Gorgas de Estudios de la Salud, 2011)	0.95 [0.80; 1.11]
Panama – (Instituto Conmemorativo Gorgas de Estudios de la Salud, 2018)	0.95 [0.76; 1.18]
Peru – (Guezmes Garcia et al., 2005)	0.70 [0.56; 0.89]
Peru – (Guezmes Garcia et al., 2005)	0.90 [0.69; 1.18]
Peru – (INEI/Perú, and ORC Macro, 2007)	1.00 [0.90; 1.11]
Peru – (INEI/Perú, and ORC Macro, 2009)	0.97 [0.89; 1.06]
Peru – (INEI/Perú, and ORC Macro, 2010)	0.97 [0.89; 1.06]
Peru – (INEI/Perú, and ORC Macro, 2011)	0.97 [0.88; 1.06]
Peru – (INEI/Perú, 2012)	0.96 [0.89; 1.04]
Peru – (INEI/Perú, 2013)	0.97 [0.89; 1.05]
Peru – (INEI/Perú, 2014)	0.97 [0.89; 1.05]
Peru – (INEI/Perú, 2015)	0.96 [0.89; 1.05]
Peru – (INEI/Perú, 2016)	0.97 [0.91; 1.04]
Peru – (INEI/Perú, 2017)	0.98 [0.91; 1.05]
Paraguay – (Centro paraguayo de estudios de población, 2009)	0.85 [0.73; 0.99]
El salvador – (Asociación Demográfica Salvadoreña – ADS/El Salvador, 2008)	0.89 [0.79; 1.01]
El salvador – (Navarros–Mantas, Velásquez, and Megías, 2015)	0.82 [0.61; 1.09]
Suriname – (Joseph, Pemberton and Phillip, 2019)	0.80 [0.61; 1.03]
Trinidad and Tobago – (Pemberton and Joseph, 2018)	0.91 [0.67; 1.23]
Total	0.90 [0.87; 0.93]
Heterogeneity: $\chi^2_{43} = 116.96$ ($P < .001$), $I^2 = 63\%$	
**SuperRegion = North Africa & Middle East**	
Afghanistan – (CSO/Afghanistan, MPH/Afghanistan, and ICF, 2017)	0.99 [0.93; 1.05]
Egypt – (MHP/Egypt, and NNPC/Egypt, 2006)	0.98 [0.86; 1.11]
Egypt – (Seedhom et al., 2010)	0.94 [0.74; 1.20]
Egypt – (MHP/Egypt and ICF International, 2015)	0.98 [0.87; 1.11]
Egypt – (Duvuury et al., 2015)	0.90 [0.84; 0.96]
Jordan – (DoS/Jordan, and Macro International, 2008)	0.87 [0.72; 1.04]
Jordan – (DoS/Jordan, and ICF International, 2013)	0.87 [0.77; 0.98]
Jordan – (DoS/Jordan, and ICF, 2019)	0.91 [0.79; 1.05]
Turkey – (Ministry of Family and Social Policies, Hacettepe University and NEE Institute of Population Studies, 2009)	0.90 [0.83; 0.97]
Turkey – (Ministry of Family and Social Policies, Hacettepe University and NEE Institute of Population Studies, 2015)	0.92 [0.82; 1.03]
Total	0.93 [0.90; 0.97]
Heterogeneity: $\chi^2_{9} = 8.87$ ($P = .45$), $I^2 = 0\%$	
**SuperRegion = Sub–Saharan Africa**	
Angola – (INE/Angola, MINSA/Angola, and ICF, 2017)	0.94 [0.85; 1.03]
Burundi – (MPBGP, MSPLS,– ISTEEBU/Burundi, and ICF, 2017)	0.75 [0.67; 0.84]
Benin – (INSAE/Bénin, and ICF, 2019)	0.84 [0.71; 0.99]
Burkina faso – (Institut National de la Statistique et de la Démographie, 2012)	0.96 [0.83; 1.11]
Central African Republic – (Institut Centrafricain des Statistiques, et des Etudes Economiques et Sociales, 2009)	0.80 [0.73; 0.88]
Côte d'Ivoire – (INS/Côte d'Ivoire, and ICF International, 2013)	0.95 [0.81; 1.11]
Cameroon – (INS/Cameroun, and ORC Macro, 2005)	0.85 [0.70; 1.02]
Cameroon – (INS/Cameroun, and ICF International, 2012)	0.78 [0.67; 0.90]
Democratic Republic of the Congo – (MP/Congo, and Macro International, 2008)	0.76 [0.63; 0.91]
Democratic Republic of the Congo – (Johnson et al., 2010)	0.91 [0.60; 1.36]
Democratic Republic of the Congo – (MPSMRM/Congo, MSP/Congo, and ICF International, 2014)	0.83 [0.73; 0.93]
Comoros – (DGSP/Comores, and ICF International, 2014)	0.87 [0.61; 1.23]
Cabo verde – (Instituto Nacional de Estatística, 2008)	0.98 [0.64; 1.49]
Cabo verde – (ICIEG, INE/Cabo Verde, 2016)	0.96 [0.68; 1.34]
Ethiopia – (Berhane et al., 2005)	0.39 [0.32; 0.47]
Ethiopia – (Deribe et al., 2012)	0.38 [0.31; 0.46]
Ethiopia – (Central Statistical Agency, 2017)	0.85 [0.73; 0.99]
Gabon – (DGS/Gabon, and ICF International, 2013)	0.91 [0.78; 1.05]
Ghana – (GSS, GHS, and Macro International, 2009)	0.88 [0.68; 1.14]
Gambia – (GBOS, and ICF International, 2014)	0.96 [0.80; 1.16]
Equatorial Guinea – (PIP/Guinea Ecuatorial, and ICF International, 2012)	0.91 [0.66; 1.25]
Kenya – (CBS/Kenya, MOH/Kenya, and ORC Macro, 2004)	0.88 [0.76; 1.02]
Kenya – (KNBS/Kenya, NACC/Kenya, NASCP/Kenya, MPHS/Kenya, and KMRI, 2010)	0.91 [0.79; 1.05]
Kenya – (KNBS/Kenya, MOH/Kenya, NACC/Kenya, KMRI/Kenya, NCPD/Kenya, 2015)	0.91 [0.79; 1.05]
Liberia – (LISGIS/Liberia, MOHSW/Liberia, NACP/Liberia, and Macro International, 2008)	0.86 [0.74; 1.00]
Mali – (Samaké et al., 2007)	0.92 [0.82; 1.04]
Mali – (CPS/SSDSPF/Mali, INSTAT/Mali, INFO–STAT/Mali, and ICF International, 2014)	0.81 [0.68; 0.96]
Mali – (INSTAT/Mali, CPSSSD, and ICF, 2019)	0.93 [0.79; 1.09]
Moçambique – (Johnson et al., 2008)	0.86 [0.74; 1.01]
Moçambique – (MISAU/Moçambique, INE/Moçambique, and ICF International, 2013)	0.93 [0.82; 1.05]
Moçambique – (INS, INE, PNCM/Moçambique and ICF, 2019)	0.95 [0.78; 1.17]
Malawi – (NSO/Malawi, and ORC Macro, 2005)	0.69 [0.61; 0.78]
Malawi – (NSO/Malawi, and ICF Macro, 2011)	0.62 [0.54; 0.71]
Malawi – (NSO/Malawi, and ICF, 2017)	0.70 [0.62; 0.80]
Namibia – (January et al., 2005)	0.79 [0.61; 1.01]
Namibia – (MoHSS/Namibia, and ICF International, 2014)	0.91 [0.66; 1.26]
Nigeria – (NPC/Nigeria, and ICF Macro, 2009)	0.95 [0.87; 1.03]
Nigeria – (NPC/Nigeria, and ICF International, 2014)	0.88 [0.82; 0.96]
Nigeria – (NPC/Nigeria, and ICF, 2019)	0.86 [0.77; 0.97]
Rwanda – (INSR/Rwanda, and ORC Macro, 2006)	0.89 [0.73; 1.07]
Rwanda – (NISR/Rwanda, MOH/Rwanda, and ICF International, 2012)	0.97 [0.83; 1.14]
Rwanda – (NISR/Rwanda, MFEP/Rwanda, MOH/Rwanda, and ICF International, 2016)	0.86 [0.68; 1.07]
Senegal – (ANSD/Sénégal, and ICF, 2018)	0.82 [0.65; 1.03]
Sierra Leone – (SSL/Sierra Leone, and ICF International, 2014)	0.96 [0.83; 1.09]
South Sudan – (Ellsberg et al., 2018)	0.61 [0.47; 0.79]
Sao Tome and Principe – (INE/São Tomé, and ICF Macro, 2010)	0.93 [0.73; 1.19]
Chad – (INSEED/Tchad, MSP/Tchad, and ICF International, 2016)	0.89 [0.76; 1.05]
Togo – (MPDAT/Togo, MS/Togo, and ICF International, 2015)	0.90 [0.77; 1.04]
Tanzania – (MoHCDGEC/Tanzania Mainland, MoH/Zanzibar, NBS/Tanzania, OCGS/Zanzibar, and ICF, 2016)	0.69 [0.54; 0.88]
Tanzania – (MoHCDGEC/Tanzania Mainland, MoH/Zanzibar, NBS/Tanzania, OCGS/Zanzibar, and ICF, 2016)	0.70 [0.55; 0.89]
Tanzania – (Mccloskey et al., 2005)	0.69 [0.55; 0.88]
Tanzania – (NBS/Tanzania, and ICF Macro, 2011)	0.83 [0.74; 0.94]
Tanzania – (MoHCDGEC/Tanzania, MoH/Zanzibar, NBS/Tanzania, OCGS/Zanzibar, and ICF, 2016)	0.91 [0.82; 1.01]
Uganda – (UBOS/Uganda, and Macro International, 2007)	0.64 [0.51; 0.80]
Uganda – (Ministry of Health/Uganda, and ICF International, 2012)	0.75 [0.60; 0.93]
Uganda – (Uganda Bureau of Statistics, 2018)	0.78 [0.70; 0.87]
South Africa – (NDH/South Africa, and ICF, 2019)	0.95 [0.83; 1.10]
Zambia – (CSO/Zambia, MOH/Zambia, TDRC/Zambia, and University of Zambia, 2009)	0.89 [0.77; 1.02]
Zambia – (CSO/Zambia, MOH/Zambia, University of Zambia THVL, DPS, TDRC/Zambia, and ICF International, 2015)	0.86 [0.79; 0.95]
Zimbabwe – (CSO/Zimbabwe, and Macro International, 2007)	0.68 [0.59; 0.78]
Zimbabwe – (ZIMSTAT/Zimbabwe, and ICF International, 2012)	0.55 [0.48; 0.63]
Zimbabwe – (ZNSA/Zimbabwe, and ICF International, 2016)	0.81 [0.72; 0.92]
Total	0.82 [0.78; 0.86]
Heterogeneity: $\chi^2_{61} = 284.73$ ($P < .001$), $I^2 = 79\%$	
Total	0.86 [0.84; 0.87]
Heterogeneity: $\chi^2_{236} = 779.89$ ($P < .001$), $I^2 = 70\%$	
Residual heterogeneity: $\chi^2_{230} = 651.44$ ($P < .001$), $I^2 = 65\%$	

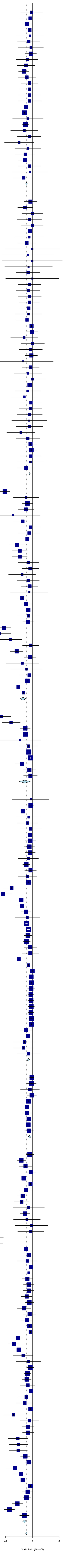

Figure S4. Forest plot of the random effect meta-analysis for past year physical intimate partner violence (IPV) only, as compared to physical and/or sexual violence.

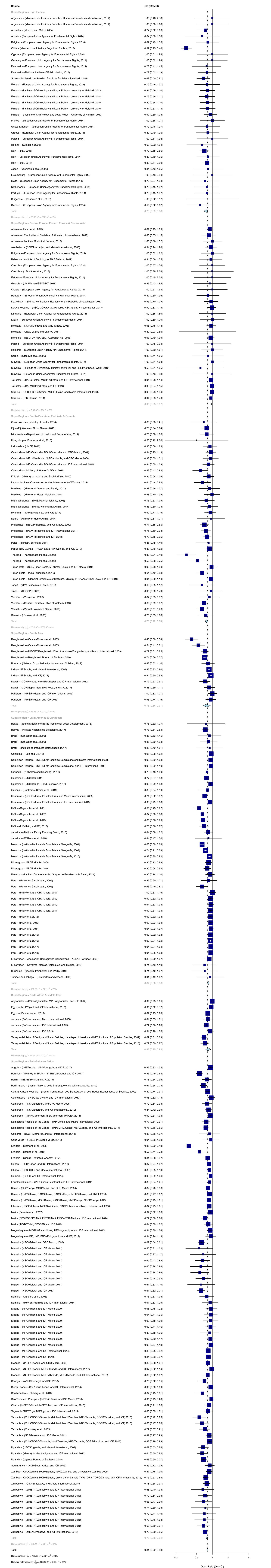
Source OR (95% CI)
SuperRegion = High Income
Argentina – (Ministerio de Justicia y Derechos Humanos Presidencia de la Nacion, 2017) 1.00 [0.46; 2.18]
Argentina – (Ministerio de Justicia y Derechos Humanos Presidencia de la Nacion, 2017) 1.00 [0.50; 1.98]
Australia – (Mouzos and Makai, 2004) 0.74 [0.52; 1.06]
Austria – (European Union Agency for Fundamental Rights, 2014) 0.64 [0.30; 1.38]
Belgium – (European Union Agency for Fundamental Rights, 2014) 0.82 [0.49; 1.36]
Chile – (Ministerio del Interior y Seguridad Pública, 2013) 0.32 [0.25; 0.40]
Cyprus – (European Union Agency for Fundamental Rights, 2014) 1.00 [0.51; 1.98]
Germany – (European Union Agency for Fundamental Rights, 2014) 1.00 [0.52; 1.94]
Denmark – (European Union Agency for Fundamental Rights, 2014) 0.78 [0.41; 1.45]
Denmark – (National Institute of Public Health, 2017) 0.79 [0.52; 1.19]
Spain – (Ministerio de Sanidad, Servicios Sociales e Igualdad, 2015) 0.68 [0.50; 0.91]
Finland – (European Union Agency for Fundamental Rights, 2014) 0.79 [0.46; 1.37]
Finland – (Institute of Criminology and Legal Policy – University of Helsinki, 2013) 0.81 [0.59; 1.10]
Finland – (Institute of Criminology and Legal Policy – University of Helsinki, 2014) 0.79 [0.56; 1.11]
Finland – (Institute of Criminology and Legal Policy – University of Helsinki, 2015) 0.80 [0.58; 1.10]
Finland – (Institute of Criminology and Legal Policy – University of Helsinki, 2016) 0.81 [0.57; 1.14]
Finland – (Institute of Criminology and Legal Policy – University of Helsinki, 2017) 0.92 [0.69; 1.23]
France – (European Union Agency for Fundamental Rights, 2014) 1.00 [0.58; 1.71]
United Kingdom – (European Union Agency for Fundamental Rights, 2014) 0.79 [0.46; 1.37]
Greece – (European Union Agency for Fundamental Rights, 2014) 0.82 [0.49; 1.36]
Ireland – (European Union Agency for Fundamental Rights, 2014) 1.00 [0.51; 1.98]
Iceland – (Gíslason, 2009) 0.63 [0.32; 1.24]
Italy – (Istat, 2006) 0.70 [0.58; 0.86]
Italy – (European Union Agency for Fundamental Rights, 2014) 0.82 [0.50; 1.36]
Italy – (Istat, 2015) 0.80 [0.64; 0.99]
Japan – (Yoshihama et al., 2005) 0.84 [0.43; 1.65]
Luxembourg – (European Union Agency for Fundamental Rights, 2014) 1.00 [0.43; 2.34]
Malta – (European Union Agency for Fundamental Rights, 2014) 0.72 [0.37; 1.38]
Netherlands – (European Union Agency for Fundamental Rights, 2014) 0.78 [0.45; 1.37]
Portugal – (European Union Agency for Fundamental Rights, 2014) 0.78 [0.45; 1.37]
Singapore – (Bouhours et al., 2013) 1.00 [0.32; 3.12]
Sweden – (European Union Agency for Fundamental Rights, 2014) 0.59 [0.32; 1.07]
Total 0.76 [0.69; 0.83]
Heterogeneity: χ^2_{31} = 58.92 (P = .002), I^2 = 47%
SuperRegion = Central Europe, Eastern Europe & Central Asia
Albania – (Haarr et al., 2013) 0.89 [0.73; 1.09]
Albania – (The Institut of Statistics of Albania ... Instat/Albania, 2018) 0.86 [0.65; 1.15]
Armenia – (National Statistical Service, 2017) 1.00 [0.66; 1.52]
Azerbaijan – (SSC/Azerbaijan, and Macro International, 2008) 0.94 [0.74; 1.20]
Bulgaria – (European Union Agency for Fundamental Rights, 2014) 1.00 [0.62; 1.62]
Belarus – (Institute of Sociology of NAS Belarus, 2019) 0.94 [0.56; 1.55]
Czechia – (European Union Agency for Fundamental Rights, 2014) 1.00 [0.57; 1.76]
Czechia – (...Buriánek et al., 2013) 1.00 [0.39; 2.54]
Estonia – (European Union Agency for Fundamental Rights, 2014) 1.00 [0.45; 2.24]
Georgia – (UN Women/GEOSTAT, 2018) 0.89 [0.43; 1.85]
Croatia – (European Union Agency for Fundamental Rights, 2014) 1.00 [0.51; 1.94]
Hungary – (European Union Agency for Fundamental Rights, 2014) 0.82 [0.50; 1.36]
Kazakhstan – (Ministry of National Economy of the Republic of Kazakhstan, 2017) 0.95 [0.70; 1.29]
Kyrgyz Republic – (NSC, MOH/Kyrgyz Republic NSC, and ICF International, 2013) 0.99 [0.83; 1.18]
Lithuania – (European Union Agency for Fundamental Rights, 2014) 1.00 [0.55; 1.80]
Latvia – (European Union Agency for Fundamental Rights, 2014) 1.00 [0.59; 1.70]
Moldova – (NCPM/Moldova, and ORC Macro, 2006) 0.96 [0.78; 1.18]
Moldova – (UNW, UNDP, and UNFPA, 2011) 0.82 [0.23; 2.86]
Mongolia – (NSO, UNFPA, SDC, Australian Aid, 2018) 0.93 [0.79; 1.09]
Poland – (European Union Agency for Fundamental Rights, 2014) 1.00 [0.45; 2.24]
Romania – (European Union Agency for Fundamental Rights, 2014) 1.00 [0.62; 1.61]
Serbia – (Otasevic et al., 2005) 0.83 [0.41; 1.66]
Slovakia – (European Union Agency for Fundamental Rights, 2014) 1.00 [0.61; 1.63]
Slovenia – (Institute of Criminology, Ministry of Interior and Faculty of Social Work, 2010) 0.59 [0.21; 1.65]
Slovenia – (European Union Agency for Fundamental Rights, 2014) 1.00 [0.43; 2.33]
Tajikistan – (SA/Tajikistan, MOH/Tajikistan, and ICF International, 2013) 0.94 [0.78; 1.14]
Tajikistan – (SA, MOH/Tajikistan, and ICF, 2018) 0.98 [0.84; 1.15]
Ukraine – (UCSR, SSC/Ukraine, MOH/Ukraine, and Macro International, 2008) 0.99 [0.73; 1.34]
Ukraine – (GfK Ukraine, 2014) 0.94 [0.63; 1.40]
Total 0.95 [0.93; 0.97]
Heterogeneity: χ^2_{28} = 3.08 (P > .99), I^2 = 0%
SuperRegion = South–East Asia, East Asia & Oceania
Cook Islands – (Ministry of Health, 2014) 0.68 [0.38; 1.21]
Fiji – (Fiji Women's Crisis Centre, 2013) 0.78 [0.64; 0.94]
Micronesia – (Department of Health and Social Affairs, 2014) 0.78 [0.58; 1.06]
Hong Kong – (Bouhours et al., 2015) 0.50 [0.12; 2.00]
Indonesia – (UNDP, 2016) 0.90 [0.66; 1.23]
Cambodia – (NIS/Cambodia, DGH/Cambodia, and ORC Macro, 2001) 0.94 [0.75; 1.19]
Cambodia – (NIPH/Cambodia, NIS/Cambodia, and ORC Macro, 2006) 0.93 [0.65; 1.31]
Cambodia – (NIS/Cambodia, DGH/Cambodia, and ICF International, 2015) 0.84 [0.65; 1.09]
Cambodia – (Ministry of Women's Affairs, 2015) 0.59 [0.42; 0.82]
Kiribati – (Ministry of Internal and Social Affairs, 2010) 0.85 [0.68; 1.06]
Laos – (National Commission for the Advancement of Women, 2015) 0.64 [0.44; 0.92]
Maldives – (Ministry of Gender and Family, 2011) 0.88 [0.56; 1.37]
Maldives – (Ministry of Health Maldives, 2018) 0.98 [0.70; 1.39]
Marshall Islands – (DHS/Marshall Islands, 2008) 0.76 [0.53; 1.09]
Marshall Islands – (Ministry of Internal Affairs, 2014) 0.88 [0.60; 1.28]
Myanmar – (MoHS/Myanmar, and ICF, 2017) 0.92 [0.71; 1.19]
Nauru – (Ministry of Home Affairs, 2014) 0.93 [0.51; 1.71]
Philippines – (NSO/Philippines, and ICF Macro, 2009) 0.71 [0.58; 0.85]
Philippines – (PSA/Philippines, and ICF International, 2014) 0.75 [0.60; 0.93]
Philippines – (PSA/Philippines, and ICF, 2018) 0.79 [0.65; 0.95]
Palau – (Ministry of Health, 2014) 0.85 [0.48; 1.49]
Papua New Guinea – (NSO/Papua New Guinea, and ICF, 2019) 0.88 [0.76; 1.02]
Thailand – (Kanchanachitra et al., 2005) 0.32 [0.21; 0.49]
Thailand – (Kanchanachitra et al., 2005) 0.52 [0.36; 0.75]
Timor–leste – (NSD/Timor–Leste, MF/Timor–Leste, and ICF Macro, 2010) 0.96 [0.78; 1.20]
Timor–Leste – (Asia Foundation, 2016) 0.64 [0.49; 0.83]
Timor–Leste – (General Directorate of Statistics, Ministry of Finance/Timor Leste, and ICF, 2018) 0.94 [0.80; 1.10]
Tonga – (Ma'a Fafine mo e Famili, 2012) 0.63 [0.35; 1.12]
Tuvalu – (CSDSPC, 2009) 0.95 [0.60; 1.49]
Vietnam – (Vung et al., 2008) 0.87 [0.55; 1.37]
Vietnam – (General Statistics Office of Vietnam, 2010) 0.69 [0.59; 0.82]
Vanuatu – (Vanuatu Women's Centre, 2011) 0.63 [0.51; 0.76]
Samoa – (Posiulai et al., 2005) 0.75 [0.55; 1.03]
Total 0.78 [0.72; 0.84]
Heterogeneity: χ^2_{32} = 58.6 (P = .003), I^2 = 45%
SuperRegion = South Asia
Bangladesh – (Garcia–Moreno et al., 2005) 0.40 [0.30; 0.54]
Bangladesh – (Garcia–Moreno et al., 2005) 0.54 [0.41; 0.71]
Bangladesh – (NIPORT/Bangladesh, Mitra, Associates/Bangladesh, and Macro International, 2009) 0.72 [0.61; 0.85]
Bangladesh – (Bangladesh Bureau of Statistics, 2016) 0.71 [0.66; 0.77]
Bhutan – (National Commission for Women and Children, 2019) 0.83 [0.62; 1.10]
India – (IIPS/India, and Macro International, 2007) 0.86 [0.83; 0.90]
India – (IIPS/India, and ICF, 2017) 0.94 [0.90; 0.98]
Nepal – (MOHP/Nepal, New ERA/Nepal, and ICF International, 2012) 0.72 [0.57; 0.91]
Nepal – (MOH/Nepal, New ERA/Nepal, and ICF, 2017) 0.88 [0.69; 1.11]
Pakistan – (NIPS/Pakistan, and ICF International, 2013) 1.00 [0.82; 1.21]
Pakistan – (NIPS/Pakistan, and ICF, 2019) 0.93 [0.74; 1.16]
Total 0.78 [0.66; 0.91]
Heterogeneity: χ^2_{10} = 88.43 (P < .001), I^2 = 89%
SuperRegion = Latin America & Caribbean
Belize – (Young Macfarlane Belize Institute for Local Development, 2015) 0.76 [0.32; 1.77]
Bolivia – (Instituto Nacional de Estadística, 2017) 0.73 [0.64; 0.84]
Brazil – (Schraiber et al., 2005) 0.88 [0.53; 1.45]
Brazil – (Schraiber et al., 2005) 0.85 [0.59; 1.23]
Brazil – (Instituto de Pesquisa DataSenado, 2017) 0.89 [0.49; 1.61]
Colombia – (Bott et al., 2015) 0.95 [0.88; 1.02]
Dominican Republic – (CESDEM/Republica Dominicana and Macro International, 2008) 0.93 [0.79; 1.08]
Dominican Republic – (CESDEM/Republica Dominicana, and ICF International, 2014) 0.93 [0.79; 1.10]
Grenada – (Nicholson and Deshong., 2018) 0.79 [0.48; 1.29]
Guatemala – (MSPAS, 2011) 0.77 [0.67; 0.88]
Guatemala – (MSPAS, INE, and Segeplán, 2017) 0.92 [0.79; 1.08]
Guyana – (Contreras–Urbina et al., 2019) 0.80 [0.54; 1.19]
Honduras – (SS/Honduras, INE/Honduras, and Macro International, 2006) 0.71 [0.62; 0.82]
Honduras – (SS/Honduras, INE/Honduras, and ICF International, 2013) 0.90 [0.79; 1.03]
Haïti – (Cayemittes et al., 2001) 0.56 [0.43; 0.72]
Haïti – (Cayemittes et al., 2007) 0.64 [0.50; 0.83]
Haïti – (Cayemittes et al., 2013) 0.66 [0.56; 0.79]
Haïti – (IHE/Haïti, and ICF, 2018) 0.70 [0.56; 0.87]
Jamaica – (National Family Planning Board, 2010) 0.84 [0.68; 1.02]
Jamaica – (Williams et al., 2018) 0.84 [0.47; 1.50]
Mexico – (Instituto National de Estadística Y Geografía, 2004) 0.63 [0.59; 0.68]
Mexico – (Instituto National de Estadística Y Geografía, 2007) 0.74 [0.71; 0.78]
Mexico – (Instituto National de Estadística Y Geografía, 2016) 0.88 [0.85; 0.92]
Nicaragua – (INIDE MINSA, 2008) 0.85 [0.73; 0.98]
Nicaragua – (INIDE MINSA, 2014) 0.80 [0.68; 0.94]
Panama – (Instituto Conmemorativo Gorgas de Estudios de la Salud, 2011) 0.90 [0.74; 1.10]
Peru – (Guezmes Garcia et al., 2005) 0.86 [0.60; 1.21]
Peru – (Guezmes Garcia et al., 2005) 0.63 [0.49; 0.81]
Peru – (INEI/Perú, and ORC Macro, 2007) 1.00 [0.87; 1.16]
Peru – (INEI/Perú, and ORC Macro, 2009) 0.92 [0.82; 1.04]
Peru – (INEI/Perú, and ORC Macro, 2010) 0.94 [0.83; 1.05]
Peru – (INEI/Perú, and ORC Macro, 2011) 0.92 [0.81; 1.04]
Peru – (INEI/Perú, 2012) 0.92 [0.82; 1.03]
Peru – (INEI/Perú, 2013) 0.93 [0.83; 1.04]
Peru – (INEI/Perú, 2014) 0.94 [0.83; 1.07]
Peru – (INEI/Perú, 2015) 0.92 [0.82; 1.03]
Peru – (INEI/Perú, 2016) 0.92 [0.84; 1.02]
Peru – (INEI/Perú, 2017) 0.94 [0.84; 1.04]
Peru – (INEI/Perú, 2018) 0.94 [0.85; 1.03]
El salvador – (Asociación Demográfica Salvadoreña – ADS/El Salvador, 2008) 0.88 [0.72; 1.07]
El salvador – (Navarros–Mantas, Velásquez, and Megías, 2015) 0.71 [0.43; 1.19]
Suriname – (Joseph, Pemberton and Phillip, 2019) 0.71 [0.40; 1.27]
Trinidad and Tobago – (Pemberton and Joseph, 2018) 0.91 [0.49; 1.67]
Total 0.84 [0.80; 0.88]
Heterogeneity: χ^2_{42} = 180.22 (P < .001), I^2 = 77%
SuperRegion = North Africa & Middle East
Afghanistan – (CSO/Afghanistan, MPH/Afghanistan, and ICF, 2017) 0.99 [0.93; 1.05]
Egypt – (MHP/Egypt and ICF International, 2015) 0.96 [0.82; 1.12]
Egypt – (Duvuury et al., 2015) 0.82 [0.75; 0.90]
Jordan – (DoS/Jordan, and Macro International, 2008) 0.81 [0.65; 1.01]
Jordan – (DoS/Jordan, and ICF International, 2013) 0.77 [0.66; 0.90]
Jordan – (DoS/Jordan, and ICF, 2019) 0.91 [0.78; 1.06]
Turkey – (Ministry of Family and Social Policies, Hacettepe University and NEE Institute of Population Studies, 2009) 0.69 [0.61; 0.79]
Turkey – (Ministry of Family and Social Policies, Hacettepe University and NEE Institute of Population Studies, 2015) 0.72 [0.60; 0.87]
Total 0.83 [0.75; 0.93]
Heterogeneity: χ^2_{7} = 37.58 (P < .001), I^2 = 81%
SuperRegion = Sub–Saharan Africa
Angola – (INE/Angola, MINSA/Angola, and ICF, 2017) 0.92 [0.83; 1.02]
Burundi – (MPBGP, MSPLS,– ISTEEBU/Burundi, and ICF, 2017) 0.56 [0.49; 0.64]
Benin – (INSAE/Bénin, and ICF, 2019) 0.78 [0.64; 0.96]
Burkina faso – (Institut National de la Statistique et de la Démographie, 2012) 0.67 [0.58; 0.78]
Central African Republic – (Institut Centrafricain des Statistiques, et des Etudes Economiques et Sociales, 2009) 0.82 [0.74; 0.91]
Côte d'Ivoire – (INS/Côte d'Ivoire, and ICF International, 2013) 0.96 [0.82; 1.13]
Cameroon – (INS/Cameroun, and ORC Macro, 2005) 0.79 [0.64; 0.98]
Cameroon – (INS/Cameroun, and ICF International, 2012) 0.84 [0.72; 0.99]
Cameroon – (MPH/Cameroon, NIS/Cameroon, UNICEF, 2014) 0.92 [0.81; 1.04]
Democratic Republic of the Congo – (MP/Congo, and Macro International, 2008) 0.77 [0.64; 0.91]
Democratic Republic of the Congo – (MPSMRM/Congo, MSP/Congo, and ICF International, 2014) 0.75 [0.66; 0.85]
Comoros – (DGSP/Comores, and ICF International, 2014) 0.88 [0.56; 1.37]
Cabo verde – (ICIEG, INE/Cabo Verde, 2016) 0.94 [0.66; 1.33]
Ethiopia – (Berhane et al., 2005) 0.35 [0.29; 0.43]
Ethiopia – (Deribe et al., 2012) 0.57 [0.41; 0.78]
Ethiopia – (Central Statistical Agency, 2017) 0.81 [0.68; 0.97]
Gabon – (DGS/Gabon, and ICF International, 2013) 0.87 [0.74; 1.02]
Ghana – (GSS, GHS, and Macro International, 2009) 0.88 [0.65; 1.19]
Gambia – (GBOS, and ICF International, 2014) 0.93 [0.69; 1.26]
Equatorial Guinea – (PIP/Guinea Ecuatorial, and ICF International, 2012) 0.88 [0.64; 1.21]
Kenya – (CBS/Kenya, MOH/Kenya, and ORC Macro, 2004) 0.82 [0.70; 0.96]
Kenya – (KNBS/Kenya, NACC/Kenya, NASCP/Kenya, MPHS/Kenya, and KMRI, 2010) 0.88 [0.77; 1.02]
Kenya – (KNBS/Kenya, MOH/Kenya, NACC/Kenya, KMRI/Kenya, NCPD/Kenya, 2015) 0.86 [0.73; 1.01]
Liberia – (LISGIS/Liberia, MOHSW/Liberia, NACP/Liberia, and Macro International, 2008) 0.87 [0.75; 1.01]
Mali – (Samaké et al., 2007) 0.92 [0.82; 1.03]
Mali – (CPS/SSDSPF/Mali, INSTAT/Mali, INFO–STAT/Mali, and ICF International, 2014) 0.72 [0.60; 0.88]
Mali – (INSTAT/Mali, CPSSSD, and ICF, 2019) 0.84 [0.69; 1.02]
Moçambique – (MISAU/Moçambique, INE/Moçambique, and ICF International, 2013) 0.91 [0.80; 1.04]
Moçambique – (INS, INE, PNCM/Moçambique and ICF, 2019) 0.94 [0.74; 1.19]
Malawi – (NSO/Malawi, and ORC Macro, 2005) 0.62 [0.54; 0.71]
Malawi – (NSO/Malawi, and ICF Macro, 2011) 0.56 [0.31; 1.02]
Malawi – (NSO/Malawi, and ICF Macro, 2011) 0.66 [0.37; 1.17]
Malawi – (NSO/Malawi, and ICF Macro, 2011) 0.65 [0.47; 0.88]
Malawi – (NSO/Malawi, and ICF Macro, 2011) 0.60 [0.38; 0.96]
Malawi – (NSO/Malawi, and ICF Macro, 2011) 0.57 [0.38; 0.86]
Malawi – (NSO/Malawi, and ICF Macro, 2011) 0.67 [0.48; 0.94]
Malawi – (NSO/Malawi, and ICF Macro, 2011) 0.61 [0.33; 1.10]
Malawi – (NSO/Malawi, and ICF, 2017) 0.61 [0.52; 0.71]
Namibia – (January et al., 2005) 0.78 [0.57; 1.06]
Namibia – (MoHSS/Namibia, and ICF International, 2014) 0.91 [0.63; 1.29]
Nigeria – (NPC/Nigeria, and ICF Macro, 2009) 0.95 [0.75; 1.22]
Nigeria – (NPC/Nigeria, and ICF Macro, 2009) 0.94 [0.71; 1.25]
Nigeria – (NPC/Nigeria, and ICF Macro, 2009) 0.93 [0.68; 1.29]
Nigeria – (NPC/Nigeria, and ICF Macro, 2009) 0.92 [0.74; 1.16]
Nigeria – (NPC/Nigeria, and ICF Macro, 2009) 0.89 [0.58; 1.36]
Nigeria – (NPC/Nigeria, and ICF Macro, 2009) 0.92 [0.72; 1.17]
Nigeria – (NPC/Nigeria, and ICF Macro, 2009) 0.93 [0.77; 1.13]
Nigeria – (NPC/Nigeria, and ICF International, 2014) 0.83 [0.75; 0.92]
Nigeria – (NPC/Nigeria, and ICF, 2019) 0.84 [0.73; 0.97]
Rwanda – (INSR/Rwanda, and ORC Macro, 2006) 0.82 [0.66; 1.01]
Rwanda – (NISR/Rwanda, MOH/Rwanda, and ICF International, 2012) 0.97 [0.82; 1.14]
Rwanda – (NISR/Rwanda, MFEP/Rwanda, MOH/Rwanda, and ICF International, 2016) 0.82 [0.62; 1.07]
Senegal – (ANSD/Sénégal, and ICF, 2018) 0.70 [0.52; 0.95]
Sierra Leone – (SSL/Sierra Leone, and ICF International, 2014) 0.93 [0.80; 1.09]
South Sudan – (Ellsberg et al., 2018) 0.64 [0.45; 0.91]
Sao Tome and Principe – (INE/São Tomé, and ICF Macro, 2010) 0.96 [0.75; 1.23]
Chad – (INSEED/Tchad, MSP/Tchad, and ICF International, 2016) 0.87 [0.71; 1.06]
Togo – (MPDAT/Togo, MS/Togo, and ICF International, 2015) 0.83 [0.69; 1.01]
Tanzania – (MoHCDGEC/Tanzania Mainland, MoH/Zanzibar, NBS/Tanzania, OCGS/Zanzibar, and ICF, 2016) 0.56 [0.42; 0.75]
Tanzania – (MoHCDGEC/Tanzania Mainland, MoH/Zanzibar, NBS/Tanzania, OCGS/Zanzibar, and ICF, 2016) 0.63 [0.47; 0.86]
Tanzania – (Mccloskey et al., 2005) 0.72 [0.57; 0.91]
Tanzania – (NBS/Tanzania, and ICF Macro, 2011) 0.87 [0.77; 0.99]
Tanzania – (MoHCDGEC/Tanzania, MoH/Zanzibar, NBS/Tanzania, OCGS/Zanzibar, and ICF, 2016) 0.88 [0.79; 0.99]
Uganda – (UBOS/Uganda, and Macro International, 2007) 0.67 [0.53; 0.84]
Uganda – (Ministry of Health/Uganda, and ICF International, 2012) 0.64 [0.50; 0.82]
Uganda – (Uganda Bureau of Statistics, 2018) 0.68 [0.60; 0.77]
South Africa – (NDH/South Africa, and ICF, 2019) 0.89 [0.72; 1.09]
Zambia – (CSO/Zambia, MOH/Zambia, TDRC/Zambia, and University of Zambia, 2009) 0.87 [0.75; 1.00]
Zambia – (CSO/Zambia, MOH/Zambia, University of Zambia THVL, DPS, TDRC/Zambia, and ICF International, 2015) 0.75 [0.67; 0.84]
Zimbabwe – (CSO/Zimbabwe, and Macro International, 2007) 0.78 [0.68; 0.91]
Zimbabwe – (ZIMSTAT/Zimbabwe, and ICF International, 2012) 0.66 [0.40; 1.08]
Zimbabwe – (ZIMSTAT/Zimbabwe, and ICF International, 2012) 0.72 [0.54; 0.98]
Zimbabwe – (ZIMSTAT/Zimbabwe, and ICF International, 2012) 0.68 [0.47; 0.99]
Zimbabwe – (ZIMSTAT/Zimbabwe, and ICF International, 2012) 0.74 [0.39; 1.38]
Zimbabwe – (ZIMSTAT/Zimbabwe, and ICF International, 2012) 0.70 [0.41; 1.19]
Zimbabwe – (ZIMSTAT/Zimbabwe, and ICF International, 2012) 0.70 [0.46; 1.08]
Zimbabwe – (ZIMSTAT/Zimbabwe, and ICF International, 2012) 0.68 [0.50; 0.91]
Zimbabwe – (ZNSA/Zimbabwe, and ICF International, 2016) 0.73 [0.62; 0.85]
Total 0.79 [0.76; 0.82]
Heterogeneity: χ^2_{77} = 236.41 (P < .001), I^2 = 67%
Total 0.81 [0.79; 0.83]
Heterogeneity: χ^2_{233} = 732.30 (P < .001), I^2 = 68%
Residual heterogeneity: χ^2_{227} = 663.26 (P < .001), I^2 = 66%
0.2 0.5 1 2 5
Odds Ratio (95% CI)

Figure S5. Forest plot of the random effect meta-analysis for lifetime sexual intimate partner violence (IPV) only, as compared to physical and/or sexual violence.

Source	OR (95% CI)
SuperRegion = High Income	
Argentina – (Ministerio de Justicia y Derechos Humanos Presidencia de la Nacion, 2017)	0.11 [0.07; 0.18]
Argentina – (Ministerio de Justicia y Derechos Humanos Presidencia de la Nacion, 2017)	0.14 [0.09; 0.22]
Australia – (Mouzos and Makai, 2004)	0.26 [0.23; 0.30]
Australia – (Personal Safety Survey, 2017)	0.50 [0.40; 0.62]
Austria – (European Union Agency for Fundamental Rights, 2014)	0.43 [0.28; 0.66]
Belgium – (European Union Agency for Fundamental Rights, 2014)	0.32 [0.22; 0.46]
Cyprus – (European Union Agency for Fundamental Rights, 2014)	0.24 [0.15; 0.39]
Germany – (Schrottle et al., 2004)	0.06 [0.04; 0.09]
Germany – (European Union Agency for Fundamental Rights, 2014)	0.31 [0.22; 0.44]
Denmark – (European Union Agency for Fundamental Rights, 2014)	0.26 [0.19; 0.35]
Spain – (Ministerio de Sanidad, Servicios Sociales e Igualdad, 2015)	0.61 [0.53; 0.71]
Finland – (European Union Agency for Fundamental Rights, 2014)	0.29 [0.21; 0.39]
France – (European Union Agency for Fundamental Rights, 2014)	0.27 [0.20; 0.38]
United Kingdom – (European Union Agency for Fundamental Rights, 2014)	0.27 [0.19; 0.37]
Greece – (European Union Agency for Fundamental Rights, 2014)	0.22 [0.15; 0.34]
Ireland – (European Union Agency for Fundamental Rights, 2014)	0.37 [0.25; 0.55]
Iceland – (Gíslason, 2009)	0.22 [0.17; 0.29]
Italy – (Istat, 2006)	0.39 [0.35; 0.43]
Italy – (European Union Agency for Fundamental Rights, 2014)	0.33 [0.21; 0.52]
Italy – (Istat, 2015)	0.39 [0.35; 0.43]
Japan – (Yoshihama et al., 2005)	0.37 [0.24; 0.56]
Luxembourg – (European Union Agency for Fundamental Rights, 2014)	0.34 [0.23; 0.51]
Malta – (Commission on Domestic Violence, 2011)	0.52 [0.35; 0.78]
Malta – (European Union Agency for Fundamental Rights, 2014)	0.38 [0.25; 0.58]
Netherlands – (European Union Agency for Fundamental Rights, 2014)	0.36 [0.27; 0.49]
New Zealand – (Fanslow et al., 2004)	0.39 [0.33; 0.47]
Portugal – (European Union Agency for Fundamental Rights, 2014)	0.13 [0.08; 0.22]
Singapore – (Bouhours et al., 2013)	0.20 [0.09; 0.42]
Sweden – (European Union Agency for Fundamental Rights, 2014)	0.28 [0.20; 0.39]
Total	0.29 [0.24; 0.35]
Heterogeneity: $\chi^2_{28} = 266.11$ ($P < .001$), $I^2 = 89\%$	
SuperRegion = Central Europe, Eastern Europe & Central Asia	
Albania – (Haarr et al., 2013)	0.26 [0.21; 0.33]
Albania – (The Institut of Statistics of Albania ... Instat/Albania, 2018)	0.35 [0.28; 0.45]
Albania – (Organisation for Security and Conflict in Europe, 2019)	0.18 [0.12; 0.28]
Armenia – (National Statistical Service of the Republic of Armenia , 2011)	0.32 [0.21; 0.49]
Armenia – (National Statistical Service, 2017)	0.12 [0.07; 0.22]
Azerbaijan – (SSC/Azerbaijan, and Macro International, 2008)	0.19 [0.14; 0.27]
Bulgaria – (European Union Agency for Fundamental Rights, 2014)	0.32 [0.21; 0.50]
Bosnia and Hezergovina – (Gender Equality Agency of Bosnia and Herzegovina, 2013)	0.35 [0.26; 0.48]
Bosnia and Hezergovina – (Organisation for Security and Conflict in Europe, 2019)	0.23 [0.08; 0.62]
Bosnia and Hezergovina – (Organisation for Security and Conflict in Europe, 2019)	0.34 [0.12; 0.96]
Bosnia and Hezergovina – (Organisation for Security and Conflict in Europe, 2019)	0.34 [0.15; 0.76]
Bosnia and Hezergovina – (Organisation for Security and Conflict in Europe, 2019)	0.34 [0.23; 0.51]
Bosnia and Hezergovina – (Organisation for Security and Conflict in Europe, 2019)	0.26 [0.09; 0.71]
Bosnia and Hezergovina – (Organisation for Security and Conflict in Europe, 2019)	0.39 [0.16; 0.96]
Belarus – (Institute of Sociology of NAS Belarus, 2019)	0.27 [0.19; 0.40]
Czechia – (European Union Agency for Fundamental Rights, 2014)	0.29 [0.20; 0.42]
Czechia – (...Buriánek et al., 2013)	0.23 [0.15; 0.35]
Estonia – (European Union Agency for Fundamental Rights, 2014)	0.30 [0.21; 0.42]
Georgia – (UN Women/GEOSTAT, 2018)	0.37 [0.27; 0.53]
Croatia – (European Union Agency for Fundamental Rights, 2014)	0.21 [0.13; 0.36]
Hungary – (European Union Agency for Fundamental Rights, 2014)	0.28 [0.19; 0.41]
Kazakhstan – (Ministry of National Economy of the Republic of Kazakhstan, 2017)	0.19 [0.15; 0.24]
Kyrgyz Republic – (NSC, MOH/Kyrgyz Republic NSC, and ICF International, 2013)	0.13 [0.10; 0.17]
Lithuania – (Schrottle et al., 2003)	0.13 [0.08; 0.22]
Lithuania – (European Union Agency for Fundamental Rights, 2014)	0.13 [0.08; 0.21]
Latvia – (European Union Agency for Fundamental Rights, 2014)	0.21 [0.14; 0.31]
Moldova – (NCPM/Moldova, and ORC Macro, 2006)	0.13 [0.10; 0.17]
Moldova – (UNW, UNDP, and UNFPA, 2011)	0.18 [0.07; 0.48]
Moldova – (Organisation for Security and Conflict in Europe, 2019)	0.19 [0.14; 0.26]
North Macedonia – (Organisation for Security and Conflict in Europe, 2019)	0.27 [0.17; 0.45]
Montenegro – (Organisation for Security and Conflict in Europe, 2019)	0.15 [0.08; 0.27]
Mongolia – (NSO, UNFPA, SDC, Australian Aid, 2018)	0.19 [0.16; 0.22]
Poland – (European Union Agency for Fundamental Rights, 2014)	0.27 [0.17; 0.43]
Kosovo – (Organisation for Security and Conflict in Europe, 2019)	0.33 [0.21; 0.53]
Romania – (European Union Agency for Fundamental Rights, 2014)	0.17 [0.11; 0.26]
Serbia – (Otasevic et al., 2005)	0.22 [0.14; 0.33]
Serbia – (Organisation for Security and Conflict in Europe, 2019)	0.27 [0.18; 0.43]
Serbia – (Organisation for Security and Conflict in Europe, 2019)	0.24 [0.12; 0.46]
Slovakia – (European Union Agency for Fundamental Rights, 2014)	0.29 [0.19; 0.43]
Slovenia – (Institute of Criminology, Ministry of Interior and Faculty of Social Work, 2010)	0.18 [0.11; 0.30]
Slovenia – (European Union Agency for Fundamental Rights, 2014)	0.27 [0.17; 0.43]
Tajikistan – (SA/Tajikistan, MOH/Tajikistan, and ICF International, 2013)	0.19 [0.15; 0.24]
Tajikistan – (SA, MOH/Tajikistan, and ICF, 2018)	0.06 [0.04; 0.08]
Ukraine – (UCSR, SSC/Ukraine, MOH/Ukraine, and Macro International, 2008)	0.22 [0.15; 0.34]
Ukraine – (GfK Ukraine, 2014)	0.19 [0.11; 0.32]
Ukraine – (Organisation for Security and Conflict in Europe, 2019)	0.22 [0.16; 0.30]
Total	0.22 [0.20; 0.25]
Heterogeneity: $\chi^2_{45} = 189.92$ ($P < .001$), $I^2 = 76\%$	
SuperRegion = South–East Asia, East Asia & Oceania	
Cook Islands – (Ministry of Health, 2014)	0.32 [0.22; 0.48]
Fiji – (Fiji Women's Crisis Centre, 2013)	0.29 [0.25; 0.33]
Micronesia – (Department of Health and Social Affairs, 2014)	0.40 [0.32; 0.50]
Hong Kong – (Bouhours et al., 2015)	0.51 [0.24; 1.07]
Indonesia – (UNDP, 2016)	0.39 [0.30; 0.52]
Cambodia – (NIS/Cambodia, DGH/Cambodia, and ORC Macro, 2001)	0.18 [0.12; 0.27]
Cambodia – (NIPH/Cambodia, NIS/Cambodia, and ORC Macro, 2006)	0.17 [0.11; 0.28]
Cambodia – (NIS/Cambodia, DGH/Cambodia, and ICF International, 2015)	0.26 [0.20; 0.34]
Cambodia – (Ministry of Women's Affairs, 2015)	0.43 [0.34; 0.54]
Kiribati – (Ministry of Internal and Social Affairs, 2010)	0.41 [0.36; 0.48]
Laos – (National Commission for the Advancement of Women, 2015)	0.40 [0.31; 0.53]
Maldives – (Ministry of Gender and Family, 2011)	0.29 [0.21; 0.42]
Maldives – (Ministry of Health Maldives, 2018)	0.24 [0.17; 0.32]
Marshall Islands – (DHS/Marshall Islands, 2008)	0.32 [0.21; 0.48]
Marshall Islands – (Ministry of Internal Affairs, 2014)	0.25 [0.18; 0.35]
Myanmar – (MoHS/Myanmar, and ICF, 2017)	0.17 [0.12; 0.24]
Nauru – (Ministry of Home Affairs, 2014)	0.28 [0.16; 0.48]
Philippines – (NSO/Philippines, and ICF Macro, 2009)	0.40 [0.34; 0.47]
Philippines – (PSA/Philippines, and ICF International, 2014)	0.34 [0.29; 0.41]
Philippines – (PSA/Philippines, and ICF, 2018)	0.32 [0.27; 0.37]
Palau – (Ministry of Health, 2014)	0.34 [0.23; 0.50]
Papua New Guinea – (NSO/Papua New Guinea, and ICF, 2019)	0.32 [0.27; 0.37]
Solomon Islands – (National Statistics Office, 2009)	0.69 [0.59; 0.81]
Thailand – (Kanchanachitra et al., 2005)	0.61 [0.46; 0.81]
Thailand – (Kanchanachitra et al., 2005)	0.45 [0.34; 0.60]
Timor–leste – (NSD/Timor–Leste, MF/Timor–Leste, and ICF Macro, 2010)	0.06 [0.04; 0.09]
Timor–Leste – (Asia Foundation, 2016)	0.48 [0.38; 0.60]
Timor–Leste – (General Directorate of Statistics, Ministry of Finance/Timor Leste, and ICF, 2018)	0.09 [0.07; 0.11]
Tonga – (Ma'a Fafine mo e Famili, 2012)	0.30 [0.18; 0.49]
Tuvalu – (CSDSPC, 2009)	0.19 [0.11; 0.33]
Vanuatu – (Vanuatu Women's Centre, 2011)	0.53 [0.43; 0.64]
Samoa – (Posiulai et al., 2005)	0.28 [0.21; 0.38]
Total	0.31 [0.25; 0.37]
Heterogeneity: $\chi^2_{31} = 368.98$ ($P < .001$), $I^2 = 92\%$	
SuperRegion = South Asia	
Bangladesh – (Garcia–Moreno et al., 2005)	0.52 [0.41; 0.66]
Bangladesh – (Garcia–Moreno et al., 2005)	0.61 [0.48; 0.78]
Bangladesh – (NIPORT/Bangladesh, Mitra, Associates/Bangladesh, and Macro International, 2009)	0.19 [0.16; 0.22]
Bangladesh – (Bangladesh Bureau of Statistics, 2016)	0.32 [0.30; 0.34]
Bhutan – (National Commission for Women and Children, 2012)	0.33 [0.18; 0.62]
Bhutan – (National Commission for Women and Children, 2019)	0.27 [0.20; 0.35]
India – (IIPS/India, and Macro International, 2007)	0.19 [0.18; 0.20]
India – (IIPS/India, and ICF, 2017)	0.16 [0.15; 0.17]
Nepal – (MOHP/Nepal, New ERA/Nepal, and ICF International, 2012)	0.42 [0.35; 0.52]
Nepal – (MOH/Nepal, New ERA/Nepal, and ICF, 2017)	0.25 [0.20; 0.32]
Pakistan – (NIPS/Pakistan, and ICF, 2019)	0.17 [0.13; 0.23]
Total	0.28 [0.20; 0.38]
Heterogeneity: $\chi^2_{10} = 453.83$ ($P < .001$), $I^2 = 98\%$	
SuperRegion = Latin America & Caribbean	
Belize – (Young Macfarlane Belize Institute for Local Development, 2015)	0.27 [0.14; 0.50]
Bolivia – (Gutiérrez, 2004)	0.16 [0.14; 0.17]
Bolivia – (Instituto Nacional de Estadística, 2017)	0.38 [0.34; 0.42]
Brazil – (Schraiber et al., 2005)	0.28 [0.18; 0.41]
Brazil – (Schraiber et al., 2005)	0.29 [0.21; 0.39]
Brazil – (Instituto de Pesquisa DataSenado, 2017)	0.12 [0.07; 0.20]
Dominican Republic – (Achécar et al., 2003)	0.29 [0.24; 0.35]
Dominican Republic – (CESDEM/Republica Dominicana and Macro International, 2008)	0.28 [0.23; 0.34]
Dominican Republic – (CESDEM/Republica Dominicana, and ICF International, 2014)	0.26 [0.22; 0.31]
Grenada – (Nicholson and Deshong., 2018)	0.26 [0.18; 0.37]
Guatemala – (MSPAS, 2011)	0.37 [0.33; 0.41]
Guatemala – (MSPAS, INE, and Segeplán, 2017)	0.25 [0.20; 0.31]
Guyana – (Contreras–Urbina et al., 2019)	0.27 [0.20; 0.36]
Honduras – (SS/Honduras, INE/Honduras, and ICF International, 2013)	0.32 [0.28; 0.36]
Haïti – (Cayemittes et al., 2001)	0.55 [0.44; 0.70]
Haïti – (Cayemittes et al., 2007)	0.62 [0.50; 0.77]
Haïti – (Cayemittes et al., 2013)	0.46 [0.40; 0.54]
Haïti – (IHE/Haïti, and ICF, 2018)	0.46 [0.39; 0.56]
Jamaica – (National Family Planning Board, 2010)	0.34 [0.29; 0.41]
Jamaica – (Williams et al., 2018)	0.22 [0.14; 0.34]
Mexico – (Instituto National de Estadística Y Geografía, 2007)	0.36 [0.34; 0.37]
Mexico – (Instituto National de Estadística Y Geografía, 2016)	0.29 [0.28; 0.30]
Nicaragua – (INIDE MINSA, 2008)	0.36 [0.33; 0.40]
Nicaragua – (INIDE MINSA, 2014)	0.39 [0.34; 0.43]
Panama – (Instituto Conmemorativo Gorgas de Estudios de la Salud, 2011)	0.20 [0.15; 0.25]
Panama – (Instituto Conmemorativo Gorgas de Estudios de la Salud, 2018)	0.33 [0.25; 0.44]
Peru – (Guezmes Garcia et al., 2005)	0.39 [0.31; 0.50]
Peru – (Guezmes Garcia et al., 2005)	0.28 [0.21; 0.37]
Peru – (INEI/Perú, and ORC Macro, 2007)	0.14 [0.12; 0.16]
Peru – (INEI/Perú, and ORC Macro, 2009)	0.14 [0.12; 0.16]
Peru – (INEI/Perú, and ORC Macro, 2010)	0.13 [0.12; 0.15]
Peru – (INEI/Perú, and ORC Macro, 2011)	0.14 [0.12; 0.16]
Peru – (INEI/Perú, 2012)	0.16 [0.14; 0.18]
Peru – (INEI/Perú, 2013)	0.16 [0.14; 0.18]
Peru – (INEI/Perú, 2014)	0.16 [0.14; 0.18]
Peru – (INEI/Perú, 2015)	0.17 [0.15; 0.19]
Peru – (INEI/Perú, 2016)	0.18 [0.16; 0.19]
Peru – (INEI/Perú, 2017)	0.15 [0.13; 0.17]
Paraguay – (Centro paraguayo de estudios de población, 2009)	0.20 [0.16; 0.26]
El salvador – (Asociación Demográfica Salvadoreña – ADS/El Salvador, 2008)	0.36 [0.32; 0.42]
El salvador – (Navarros–Mantas, Velásquez, and Megías, 2015)	0.43 [0.31; 0.60]
Suriname – (Joseph, Pemberton and Phillip, 2019)	0.33 [0.24; 0.44]
Trinidad and Tobago – (Pemberton and Joseph, 2018)	0.27 [0.19; 0.40]
Total	0.26 [0.23; 0.30]
Heterogeneity: $\chi^2_{42} = 1553.85$ ($P < .001$), $I^2 = 97\%$	
SuperRegion = North Africa & Middle East	
Afghanistan – (CSO/Afghanistan, MPH/Afghanistan, and ICF, 2017)	0.08 [0.07; 0.09]
Egypt – (MHP/Egypt, and NNPC/Egypt, 2006)	0.14 [0.11; 0.17]
Egypt – (Seedhom et al., 2010)	0.17 [0.12; 0.24]
Egypt – (MHP/Egypt and ICF International, 2015)	0.13 [0.11; 0.16]
Egypt – (Duvuury et al., 2015)	0.27 [0.25; 0.30]
Jordan – (DoS/Jordan, and Macro International, 2008)	0.28 [0.22; 0.35]
Jordan – (DoS/Jordan, and ICF International, 2013)	0.29 [0.25; 0.34]
Jordan – (DoS/Jordan, and ICF, 2019)	0.23 [0.19; 0.28]
Turkey – (Ministry of Family and Social Policies, Hacettepe University and NEE Institute of Population Studies, 2009)	0.25 [0.23; 0.27]
Turkey – (Ministry of Family and Social Policies, Hacettepe University and NEE Institute of Population Studies, 2015)	0.23 [0.20; 0.26]
Total	0.19 [0.14; 0.26]
Heterogeneity: $\chi^2_{9} = 539.03$ ($P < .001$), $I^2 = 98\%$	
SuperRegion = Sub–Saharan Africa	
Angola – (INE/Angola, MINSA/Angola, and ICF, 2017)	0.17 [0.15; 0.19]
Burundi – (MPBGP, MSPLS,– ISTEEBU/Burundi, and ICF, 2017)	0.39 [0.35; 0.44]
Benin – (INSAE/Bénin, and ICF, 2019)	0.34 [0.28; 0.41]
Burkina faso – (Institut National de la Statistique et de la Démographie, 2012)	0.12 [0.09; 0.15]
Central African Republic – (Institut Centrafricain des Statistiques, et des Etudes Economiques et Sociales, 2009)	0.31 [0.27; 0.35]
Côte d'Ivoire – (INS/Côte d'Ivoire, and ICF International, 2013)	0.16 [0.13; 0.21]
Cameroon – (INS/Cameroun, and ORC Macro, 2005)	0.23 [0.18; 0.28]
Cameroon – (INS/Cameroun, and ICF International, 2012)	0.24 [0.21; 0.29]
Democratic Republic of the Congo – (MP/Congo, and Macro International, 2008)	0.30 [0.25; 0.37]
Democratic Republic of the Congo – (Johnson et al., 2010)	0.08 [0.04; 0.18]
Democratic Republic of the Congo – (MPSMRM/Congo, MSP/Congo, and ICF International, 2014)	0.33 [0.29; 0.38]
Comoros – (DGSP/Comores, and ICF International, 2014)	0.37 [0.24; 0.58]
Cabo verde – (Instituto Nacional de Estatística, 2008)	0.19 [0.10; 0.37]
Cabo verde – (ICIEG, INE/Cabo Verde, 2016)	0.22 [0.14; 0.37]
Ethiopia – (Berhane et al., 2005)	0.58 [0.48; 0.71]
Ethiopia – (Deribe et al., 2012)	0.55 [0.42; 0.71]
Ethiopia – (Central Statistical Agency, 2017)	0.32 [0.27; 0.38]
Gabon – (DGS/Gabon, and ICF International, 2013)	0.22 [0.19; 0.27]
Ghana – (GSS, GHS, and Macro International, 2009)	0.30 [0.22; 0.42]
Gambia – (GBOS, and ICF International, 2014)	0.11 [0.08; 0.16]
Equatorial Guinea – (PIP/Guinea Ecuatorial, and ICF International, 2012)	0.16 [0.11; 0.23]
Kenya – (CBS/Kenya, MOH/Kenya, and ORC Macro, 2004)	0.25 [0.21; 0.29]
Kenya – (KNBS/Kenya, NACC/Kenya, NASCP/Kenya, MPHS/Kenya, and KMRI, 2010)	0.26 [0.22; 0.31]
Kenya – (KNBS/Kenya, MOH/Kenya, NACC/Kenya, KMRI/Kenya, NCPD/Kenya, 2015)	0.24 [0.20; 0.28]
Liberia – (LISGIS/Liberia, MOHSW/Liberia, NACP/Liberia, and Macro International, 2008)	0.19 [0.16; 0.23]
Mali – (Samaké et al., 2007)	0.17 [0.14; 0.21]
Mali – (CPS/SSDSPF/Mali, INSTAT/Mali, INFO–STAT/Mali, and ICF International, 2014)	0.30 [0.24; 0.36]
Mali – (INSTAT/Mali, CPSSSD, and ICF, 2019)	0.21 [0.17; 0.26]
Moçambique – (Johnson et al., 2008)	0.25 [0.20; 0.30]
Moçambique – (MISAU/Moçambique, INE/Moçambique, and ICF International, 2013)	0.17 [0.14; 0.21]
Moçambique – (INS, INE, PNCM/Moçambique and ICF, 2019)	0.19 [0.14; 0.25]
Malawi – (NSO/Malawi, and ORC Macro, 2005)	0.42 [0.37; 0.48]
Malawi – (NSO/Malawi, and ICF Macro, 2011)	0.52 [0.45; 0.60]
Malawi – (NSO/Malawi, and ICF, 2017)	0.44 [0.38; 0.51]
Namibia – (January et al., 2005)	0.35 [0.27; 0.47]
Namibia – (MoHSS/Namibia, and ICF International, 2014)	0.26 [0.17; 0.39]
Nigeria – (NPC/Nigeria, and ICF Macro, 2009)	0.18 [0.16; 0.21]
Nigeria – (NPC/Nigeria, and ICF International, 2014)	0.28 [0.25; 0.31]
Nigeria – (NPC/Nigeria, and ICF, 2019)	0.29 [0.25; 0.34]
Rwanda – (INSR/Rwanda, and ORC Macro, 2006)	0.28 [0.22; 0.35]
Rwanda – (NISR/Rwanda, MOH/Rwanda, and ICF International, 2012)	0.16 [0.14; 0.20]
Rwanda – (NISR/Rwanda, MFEP/Rwanda, MOH/Rwanda, and ICF International, 2016)	0.26 [0.20; 0.34]
Senegal – (ANSD/Sénégal, and ICF, 2018)	0.32 [0.24; 0.42]
Sierra Leone – (SSL/Sierra Leone, and ICF International, 2014)	0.11 [0.09; 0.13]
South Sudan – (Ellsberg et al., 2018)	0.46 [0.36; 0.60]
Sao Tome and Principe – (INE/São Tomé, and ICF Macro, 2010)	0.23 [0.17; 0.32]
Chad – (INSEED/Tchad, MSP/Tchad, and ICF International, 2016)	0.28 [0.23; 0.35]
Togo – (MPDAT/Togo, MS/Togo, and ICF International, 2015)	0.30 [0.25; 0.36]
Tanzania – (MoHCDGEC/Tanzania Mainland, MoH/Zanzibar, NBS/Tanzania, OCGS/Zanzibar, and ICF, 2016)	0.35 [0.27; 0.45]
Tanzania – (MoHCDGEC/Tanzania Mainland, MoH/Zanzibar, NBS/Tanzania, OCGS/Zanzibar, and ICF, 2016)	0.43 [0.33; 0.55]
Tanzania – (NBS/Tanzania, and ICF Macro, 2011)	0.27 [0.23; 0.31]
Tanzania – (MoHCDGEC/Tanzania, MoH/Zanzibar, NBS/Tanzania, OCGS/Zanzibar, and ICF, 2016)	0.22 [0.19; 0.25]
Uganda – (UBOS/Uganda, and Macro International, 2007)	0.38 [0.30; 0.48]
Uganda – (Ministry of Health/Uganda, and ICF International, 2012)	0.36 [0.28; 0.45]
Uganda – (Uganda Bureau of Statistics, 2018)	0.33 [0.29; 0.37]
South Africa – (NDH/South Africa, and ICF, 2019)	0.24 [0.20; 0.30]
Zambia – (CSO/Zambia, MOH/Zambia, TDRC/Zambia, and University of Zambia, 2009)	0.20 [0.17; 0.24]
Zambia – (CSO/Zambia, MOH/Zambia, University of Zambia THVL, DPS, TDRC/Zambia, and ICF International, 2015)	0.27 [0.24; 0.30]
Zimbabwe – (CSO/Zimbabwe, and Macro International, 2007)	0.38 [0.32; 0.44]
Zimbabwe – (ZIMSTAT/Zimbabwe, and ICF International, 2012)	0.48 [0.42; 0.55]
Zimbabwe – (ZNSA/Zimbabwe, and ICF International, 2016)	0.26 [0.23; 0.30]
Total	0.27 [0.24; 0.29]
Heterogeneity: $\chi^2_{60} = 872.24$ ($P < .001$), $I^2 = 93\%$	
Total	0.26 [0.25; 0.28]
Heterogeneity: $\chi^2_{231} = 5084.25$ ($P < .001$), $I^2 = 95\%$	
Residual heterogeneity: $\chi^2_{225} = 4243.96$ ($P < .001$), $I^2 = 95\%$	

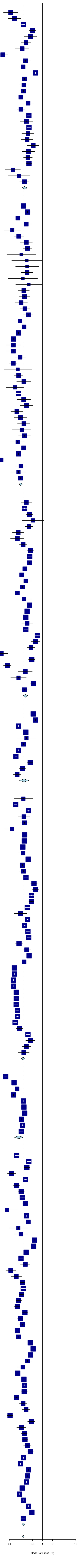

Figure S6. Forest plot of the random effect meta-analysis for past year for sexual intimate partner violence (IPV) only, as compared to physical and/or sexual violence.

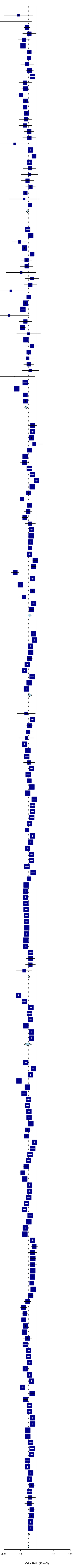

Source	OR (95% CI)
**SuperRegion = High Income**	
Argentina – (Ministerio de Justicia y Derechos Humanos Presidencia de la Nacion, 2017)	0.08 [0.01; 0.58]
Argentina – (Ministerio de Justicia y Derechos Humanos Presidencia de la Nacion, 2017)	0.03 [0.00; 0.46]
Australia – (Mouzos and Makai, 2004)	0.24 [0.16; 0.38]
Austria – (European Union Agency for Fundamental Rights, 2014)	0.35 [0.14; 0.88]
Belgium – (European Union Agency for Fundamental Rights, 2014)	0.16 [0.07; 0.39]
Chile – (Ministerio del Interior y Seguridad Pública, 2013)	0.14 [0.10; 0.20]
Cyprus – (European Union Agency for Fundamental Rights, 2014)	0.35 [0.14; 0.88]
Germany – (European Union Agency for Fundamental Rights, 2014)	0.33 [0.13; 0.83]
Denmark – (European Union Agency for Fundamental Rights, 2014)	0.25 [0.10; 0.63]
Denmark – (National Institute of Public Health, 2017)	0.35 [0.21; 0.60]
Spain – (Ministerio de Sanidad, Servicios Sociales e Igualdad, 2015)	0.53 [0.38; 0.73]
Finland – (European Union Agency for Fundamental Rights, 2014)	0.19 [0.08; 0.46]
Finland – (Institute of Criminology and Legal Policy – University of Helsinki, 2013)	0.19 [0.12; 0.32]
Finland – (Institute of Criminology and Legal Policy – University of Helsinki, 2014)	0.11 [0.05; 0.22]
Finland – (Institute of Criminology and Legal Policy – University of Helsinki, 2015)	0.20 [0.12; 0.33]
Finland – (Institute of Criminology and Legal Policy – University of Helsinki, 2016)	0.19 [0.11; 0.33]
Finland – (Institute of Criminology and Legal Policy – University of Helsinki, 2017)	0.23 [0.14; 0.36]
France – (European Union Agency for Fundamental Rights, 2014)	0.21 [0.08; 0.50]
United Kingdom – (European Union Agency for Fundamental Rights, 2014)	0.19 [0.08; 0.46]
Greece – (European Union Agency for Fundamental Rights, 2014)	0.33 [0.17; 0.64]
Ireland – (European Union Agency for Fundamental Rights, 2014)	0.35 [0.14; 0.88]
Iceland – (Gíslason, 2009)	0.04 [0.01; 0.33]
Italy – (Istat, 2006)	0.41 [0.32; 0.52]
Italy – (European Union Agency for Fundamental Rights, 2014)	0.65 [0.38; 1.11]
Italy – (Istat, 2015)	0.34 [0.26; 0.46]
Japan – (Yoshihama et al., 2005)	0.36 [0.15; 0.86]
Luxembourg – (European Union Agency for Fundamental Rights, 2014)	0.36 [0.11; 1.13]
Malta – (European Union Agency for Fundamental Rights, 2014)	0.26 [0.11; 0.66]
Netherlands – (European Union Agency for Fundamental Rights, 2014)	0.40 [0.20; 0.79]
Portugal – (European Union Agency for Fundamental Rights, 2014)	0.20 [0.08; 0.48]
Singapore – (Bouhours et al., 2013)	0.17 [0.02; 1.38]
Sweden – (European Union Agency for Fundamental Rights, 2014)	0.39 [0.20; 0.76]
Total	0.27 [0.22; 0.32]
Heterogeneity: $\chi^2_{31} = 84.2$ ($P < .001$), $I^2 = 63\%$	
**SuperRegion = Central Europe, Eastern Europe & Central Asia**	
Albania – (Haarr et al., 2013)	0.27 [0.21; 0.36]
Albania – (The Institut of Statistics of Albania ... Instat/Albania, 2018)	0.42 [0.30; 0.59]
Armenia – (National Statistical Service, 2017)	0.09 [0.03; 0.24]
Azerbaijan – (SSC/Azerbaijan, and Macro International, 2008)	0.18 [0.12; 0.26]
Bulgaria – (European Union Agency for Fundamental Rights, 2014)	0.50 [0.28; 0.89]
Belarus – (Institute of Sociology of NAS Belarus, 2019)	0.23 [0.11; 0.51]
Czechia – (European Union Agency for Fundamental Rights, 2014)	0.23 [0.09; 0.57]
Czechia – (...Buriánek et al., 2013)	0.11 [0.01; 0.87]
Estonia – (European Union Agency for Fundamental Rights, 2014)	0.49 [0.18; 1.33]
Georgia – (UN Women/GEOSTAT, 2018)	0.39 [0.16; 0.96]
Croatia – (European Union Agency for Fundamental Rights, 2014)	0.03 [0.00; 0.44]
Hungary – (European Union Agency for Fundamental Rights, 2014)	0.32 [0.16; 0.62]
Kazakhstan – (Ministry of National Economy of the Republic of Kazakhstan, 2017)	0.20 [0.13; 0.30]
Kyrgyz Republic – (NSC, MOH/Kyrgyz Republic NSC, and ICF International, 2013)	0.14 [0.10; 0.19]
Lithuania – (European Union Agency for Fundamental Rights, 2014)	0.02 [0.00; 0.34]
Latvia – (European Union Agency for Fundamental Rights, 2014)	0.20 [0.08; 0.48]
Moldova – (NCPM/Moldova, and ORC Macro, 2006)	0.14 [0.09; 0.20]
Moldova – (UNW, UNDP, and UNFPA, 2011)	0.31 [0.06; 1.60]
Mongolia – (NSO, UNFPA, SDC, Australian Aid, 2018)	0.21 [0.17; 0.27]
Poland – (European Union Agency for Fundamental Rights, 2014)	0.49 [0.18; 1.33]
Romania – (European Union Agency for Fundamental Rights, 2014)	0.32 [0.16; 0.62]
Serbia – (Otasevic et al., 2005)	0.27 [0.10; 0.73]
Slovakia – (European Union Agency for Fundamental Rights, 2014)	0.31 [0.16; 0.62]
Slovenia – (Institute of Criminology, Ministry of Interior and Faculty of Social Work, 2010)	0.39 [0.12; 1.26]
Slovenia – (European Union Agency for Fundamental Rights, 2014)	0.04 [0.00; 0.72]
Tajikistan – (SA/Tajikistan, MOH/Tajikistan, and ICF International, 2013)	0.19 [0.14; 0.26]
Tajikistan – (SA, MOH/Tajikistan, and ICF, 2018)	0.06 [0.04; 0.09]
Ukraine – (UCSR, SSC/Ukraine, MOH/Ukraine, and Macro International, 2008)	0.19 [0.12; 0.31]
Ukraine – (GfK Ukraine, 2014)	0.20 [0.11; 0.38]
Total	0.22 [0.17; 0.27]
Heterogeneity: $\chi^2_{28} = 105.06$ ($P < .001$), $I^2 = 73\%$	
**SuperRegion = South–East Asia, East Asia & Oceania**	
Cook Islands – (Ministry of Health, 2014)	0.54 [0.29; 1.01]
Fiji – (Fiji Women's Crisis Centre, 2013)	0.53 [0.43; 0.65]
Micronesia – (Department of Health and Social Affairs, 2014)	0.46 [0.31; 0.69]
Hong Kong – (Bouhours et al., 2015)	0.66 [0.19; 2.37]
Indonesia – (UNDP, 2016)	0.36 [0.20; 0.62]
Cambodia – (NIS/Cambodia, DGH/Cambodia, and ORC Macro, 2001)	0.19 [0.13; 0.28]
Cambodia – (NIPH/Cambodia, NIS/Cambodia, and ORC Macro, 2006)	0.18 [0.10; 0.32]
Cambodia – (NIS/Cambodia, DGH/Cambodia, and ICF International, 2015)	0.34 [0.24; 0.47]
Cambodia – (Ministry of Women's Affairs, 2015)	0.51 [0.39; 0.68]
Kiribati – (Ministry of Internal and Social Affairs, 2010)	0.90 [0.72; 1.12]
Laos – (National Commission for the Advancement of Women, 2015)	0.50 [0.34; 0.75]
Maldives – (Ministry of Gender and Family, 2011)	0.30 [0.17; 0.56]
Maldives – (Ministry of Health Maldives, 2018)	0.12 [0.06; 0.25]
Marshall Islands – (DHS/Marshall Islands, 2008)	0.35 [0.23; 0.54]
Marshall Islands – (Ministry of Internal Affairs, 2014)	0.29 [0.17; 0.47]
Myanmar – (MoHS/Myanmar, and ICF, 2017)	0.18 [0.12; 0.28]
Nauru – (Ministry of Home Affairs, 2014)	0.38 [0.18; 0.79]
Philippines – (NSO/Philippines, and ICF Macro, 2009)	0.45 [0.36; 0.56]
Philippines – (PSA/Philippines, and ICF International, 2014)	0.43 [0.34; 0.56]
Philippines – (PSA/Philippines, and ICF, 2018)	0.39 [0.31; 0.49]
Palau – (Ministry of Health, 2014)	0.37 [0.18; 0.76]
Papua New Guinea – (NSO/Papua New Guinea, and ICF, 2019)	0.35 [0.30; 0.41]
Thailand – (Kanchanachitra et al., 2005)	0.77 [0.54; 1.09]
Thailand – (Kanchanachitra et al., 2005)	0.62 [0.44; 0.88]
Timor–leste – (NSD/Timor–Leste, MF/Timor–Leste, and ICF Macro, 2010)	0.05 [0.03; 0.08]
Timor–Leste – (Asia Foundation, 2016)	0.52 [0.42; 0.66]
Timor–Leste – (General Directorate of Statistics, Ministry of Finance/Timor Leste, and ICF, 2018)	0.10 [0.07; 0.13]
Tonga – (Ma'a Fafine mo e Famili, 2012)	0.54 [0.30; 0.97]
Tuvalu – (CSDSPC, 2009)	0.16 [0.08; 0.32]
Vanuatu – (Vanuatu Women's Centre, 2011)	0.63 [0.51; 0.76]
Samoa – (Posiulai et al., 2005)	0.45 [0.31; 0.63]
Total	0.35 [0.28; 0.45]
Heterogeneity: $\chi^2_{30} = 344.17$ ($P < .001$), $I^2 = 91\%$	
**SuperRegion = South Asia**	
Bangladesh – (Garcia–Moreno et al., 2005)	0.58 [0.44; 0.77]
Bangladesh – (Garcia–Moreno et al., 2005)	0.68 [0.52; 0.89]
Bangladesh – (NIPORT/Bangladesh, Mitra, Associates/Bangladesh, and Macro International, 2009)	0.39 [0.33; 0.48]
Bangladesh – (Bangladesh Bureau of Statistics, 2016)	0.42 [0.38; 0.45]
Bhutan – (National Commission for Women and Children, 2019)	0.36 [0.25; 0.52]
India – (IIPS/India, and Macro International, 2007)	0.25 [0.24; 0.27]
India – (IIPS/India, and ICF, 2017)	0.18 [0.17; 0.19]
Nepal – (MOHP/Nepal, New ERA/Nepal, and ICF International, 2012)	0.50 [0.39; 0.65]
Nepal – (MOH/Nepal, New ERA/Nepal, and ICF, 2017)	0.33 [0.24; 0.45]
Pakistan – (NIPS/Pakistan, and ICF, 2019)	0.22 [0.16; 0.31]
Total	0.36 [0.26; 0.49]
Heterogeneity: $\chi^2_{9} = 405.58$ ($P < .001$), $I^2 = 98\%$	
**SuperRegion = Latin America & Caribbean**	
Belize – (Young Macfarlane Belize Institute for Local Development, 2015)	0.22 [0.06; 0.78]
Bolivia – (Instituto Nacional de Estadística, 2017)	0.52 [0.46; 0.60]
Brazil – (Schraiber et al., 2005)	0.35 [0.22; 0.55]
Brazil – (Schraiber et al., 2005)	0.29 [0.15; 0.59]
Brazil – (Instituto de Pesquisa DataSenado, 2017)	0.23 [0.08; 0.66]
Colombia – (Bott et al., 2015)	0.18 [0.16; 0.20]
Dominican Republic – (CESDEM/Republica Dominicana and Macro International, 2008)	0.28 [0.23; 0.35]
Dominican Republic – (CESDEM/Republica Dominicana, and ICF International, 2014)	0.24 [0.19; 0.30]
Grenada – (Nicholson and Deshong., 2018)	0.33 [0.15; 0.72]
Guatemala – (MSPAS, 2011)	0.46 [0.39; 0.54]
Guatemala – (MSPAS, INE, and Segeplán, 2017)	0.29 [0.23; 0.36]
Guyana – (Contreras–Urbina et al., 2019)	0.33 [0.20; 0.55]
Honduras – (SS/Honduras, INE/Honduras, and Macro International, 2006)	0.49 [0.42; 0.57]
Honduras – (SS/Honduras, INE/Honduras, and ICF International, 2013)	0.28 [0.23; 0.33]
Haïti – (Cayemittes et al., 2001)	0.66 [0.52; 0.85]
Haïti – (Cayemittes et al., 2007)	0.53 [0.40; 0.69]
Haïti – (Cayemittes et al., 2013)	0.54 [0.45; 0.64]
Haïti – (IHE/Haïti, and ICF, 2018)	0.48 [0.38; 0.61]
Jamaica – (National Family Planning Board, 2010)	0.35 [0.27; 0.45]
Jamaica – (Williams et al., 2018)	0.25 [0.11; 0.57]
Mexico – (Instituto National de Estadística Y Geografía, 2004)	0.53 [0.49; 0.57]
Mexico – (Instituto National de Estadística Y Geografía, 2007)	0.41 [0.39; 0.44]
Mexico – (Instituto National de Estadística Y Geografía, 2016)	0.27 [0.24; 0.30]
Nicaragua – (INIDE MINSA, 2008)	0.45 [0.38; 0.53]
Nicaragua – (INIDE MINSA, 2014)	0.45 [0.37; 0.54]
Panama – (Instituto Conmemorativo Gorgas de Estudios de la Salud, 2011)	0.25 [0.19; 0.33]
Peru – (Guezmes Garcia et al., 2005)	0.57 [0.45; 0.74]
Peru – (Guezmes Garcia et al., 2005)	0.33 [0.21; 0.50]
Peru – (INEI/Perú, and ORC Macro, 2007)	0.22 [0.17; 0.27]
Peru – (INEI/Perú, and ORC Macro, 2009)	0.21 [0.17; 0.25]
Peru – (INEI/Perú, and ORC Macro, 2010)	0.20 [0.17; 0.24]
Peru – (INEI/Perú, and ORC Macro, 2011)	0.21 [0.18; 0.26]
Peru – (INEI/Perú, 2012)	0.22 [0.18; 0.26]
Peru – (INEI/Perú, 2013)	0.22 [0.19; 0.27]
Peru – (INEI/Perú, 2014)	0.22 [0.19; 0.27]
Peru – (INEI/Perú, 2015)	0.24 [0.20; 0.28]
Peru – (INEI/Perú, 2016)	0.22 [0.19; 0.26]
Peru – (INEI/Perú, 2017)	0.21 [0.18; 0.25]
Peru – (INEI/Perú, 2018)	0.21 [0.18; 0.24]
El salvador – (Asociación Demográfica Salvadoreña – ADS/El Salvador, 2008)	0.41 [0.32; 0.52]
El salvador – (Navarros–Mantas, Velásquez, and Megías, 2015)	0.41 [0.23; 0.75]
Suriname – (Joseph, Pemberton and Phillip, 2019)	0.40 [0.20; 0.79]
Trinidad and Tobago – (Pemberton and Joseph, 2018)	0.17 [0.06; 0.48]
Total	0.32 [0.28; 0.36]
Heterogeneity: $\chi^2_{42} = 819.03$ ($P < .001$), $I^2 = 95\%$	
**SuperRegion = North Africa & Middle East**	
Afghanistan – (CSO/Afghanistan, MPH/Afghanistan, and ICF, 2017)	0.08 [0.07; 0.08]
Egypt – (MHP/Egypt and ICF International, 2015)	0.17 [0.13; 0.22]
Egypt – (Duvuury et al., 2015)	0.43 [0.38; 0.48]
Jordan – (DoS/Jordan, and Macro International, 2008)	0.35 [0.26; 0.46]
Jordan – (DoS/Jordan, and ICF International, 2013)	0.39 [0.32; 0.47]
Jordan – (DoS/Jordan, and ICF, 2019)	0.21 [0.17; 0.27]
Turkey – (Ministry of Family and Social Policies, Hacettepe University and NEE Institute of Population Studies, 2009)	0.47 [0.41; 0.55]
Turkey – (Ministry of Family and Social Policies, Hacettepe University and NEE Institute of Population Studies, 2015)	0.45 [0.36; 0.56]
Total	0.28 [0.16; 0.47]
Heterogeneity: $\chi^2_{7} = 785.5$ ($P < .001$), $I^2 = 99\%$	
**SuperRegion = Sub–Saharan Africa**	
Angola – (INE/Angola, MINSA/Angola, and ICF, 2017)	0.21 [0.18; 0.24]
Burundi – (MPBGP, MSPLS,– ISTEEBU/Burundi, and ICF, 2017)	0.59 [0.51; 0.67]
Benin – (INSAE/Bénin, and ICF, 2019)	0.40 [0.31; 0.51]
Burkina faso – (Institut National de la Statistique et de la Démographie, 2012)	0.08 [0.06; 0.11]
Central African Republic – (Institut Centrafricain des Statistiques, et des Etudes Economiques et Sociales, 2009)	0.25 [0.22; 0.29]
Côte d'Ivoire – (INS/Côte d'Ivoire, and ICF International, 2013)	0.16 [0.13; 0.21]
Cameroon – (INS/Cameroun, and ORC Macro, 2005)	0.30 [0.23; 0.39]
Cameroon – (INS/Cameroun, and ICF International, 2012)	0.26 [0.21; 0.32]
Cameroon – (MPH/Cameroon, NIS/Cameroon, UNICEF, 2014)	0.32 [0.28; 0.37]
Democratic Republic of the Congo – (MP/Congo, and Macro International, 2008)	0.33 [0.27; 0.39]
Democratic Republic of the Congo – (MPSMRM/Congo, MSP/Congo, and ICF International, 2014)	0.43 [0.37; 0.49]
Comoros – (DGSP/Comores, and ICF International, 2014)	0.27 [0.14; 0.51]
Cabo verde – (ICIEG, INE/Cabo Verde, 2016)	0.22 [0.13; 0.38]
Ethiopia – (Berhane et al., 2005)	0.69 [0.57; 0.83]
Ethiopia – (Deribe et al., 2012)	0.55 [0.40; 0.76]
Ethiopia – (Central Statistical Agency, 2017)	0.38 [0.30; 0.47]
Gabon – (DGS/Gabon, and ICF International, 2013)	0.30 [0.24; 0.36]
Ghana – (GSS, GHS, and Macro International, 2009)	0.22 [0.15; 0.33]
Gambia – (GBOS, and ICF International, 2014)	0.14 [0.08; 0.24]
Equatorial Guinea – (PIP/Guinea Ecuatorial, and ICF International, 2012)	0.18 [0.12; 0.27]
Kenya – (CBS/Kenya, MOH/Kenya, and ORC Macro, 2004)	0.34 [0.29; 0.42]
Kenya – (KNBS/Kenya, NACC/Kenya, NASCP/Kenya, MPHS/Kenya, and KMRI, 2010)	0.35 [0.30; 0.42]
Kenya – (KNBS/Kenya, MOH/Kenya, NACC/Kenya, KMRI/Kenya, NCPD/Kenya, 2015)	0.32 [0.26; 0.39]
Liberia – (LISGIS/Liberia, MOHSW/Liberia, NACP/Liberia, and Macro International, 2008)	0.23 [0.19; 0.28]
Mali – (Samaké et al., 2007)	0.17 [0.14; 0.21]
Mali – (CPS/SSDSPF/Mali, INSTAT/Mali, INFO–STAT/Mali, and ICF International, 2014)	0.38 [0.30; 0.47]
Mali – (INSTAT/Mali, CPSSSD, and ICF, 2019)	0.32 [0.25; 0.41]
Moçambique – (MISAU/Moçambique, INE/Moçambique, and ICF International, 2013)	0.19 [0.16; 0.23]
Moçambique – (INS, INE, PNCM/Moçambique and ICF, 2019)	0.18 [0.13; 0.27]
Malawi – (NSO/Malawi, and ORC Macro, 2005)	0.53 [0.46; 0.61]
Malawi – (NSO/Malawi, and ICF Macro, 2011)	0.66 [0.42; 1.05]
Malawi – (NSO/Malawi, and ICF Macro, 2011)	0.53 [0.29; 0.97]
Malawi – (NSO/Malawi, and ICF Macro, 2011)	0.56 [0.37; 0.84]
Malawi – (NSO/Malawi, and ICF Macro, 2011)	0.54 [0.29; 1.00]
Malawi – (NSO/Malawi, and ICF Macro, 2011)	0.55 [0.40; 0.77]
Malawi – (NSO/Malawi, and ICF Macro, 2011)	0.51 [0.36; 0.73]
Malawi – (NSO/Malawi, and ICF Macro, 2011)	0.48 [0.26; 0.88]
Malawi – (NSO/Malawi, and ICF, 2017)	0.57 [0.49; 0.67]
Namibia – (January et al., 2005)	0.41 [0.29; 0.59]
Namibia – (MoHSS/Namibia, and ICF International, 2014)	0.27 [0.17; 0.44]
Nigeria – (NPC/Nigeria, and ICF Macro, 2009)	0.15 [0.10; 0.23]
Nigeria – (NPC/Nigeria, and ICF Macro, 2009)	0.19 [0.12; 0.29]
Nigeria – (NPC/Nigeria, and ICF Macro, 2009)	0.16 [0.09; 0.27]
Nigeria – (NPC/Nigeria, and ICF Macro, 2009)	0.20 [0.14; 0.29]
Nigeria – (NPC/Nigeria, and ICF Macro, 2009)	0.17 [0.12; 0.24]
Nigeria – (NPC/Nigeria, and ICF Macro, 2009)	0.27 [0.15; 0.49]
Nigeria – (NPC/Nigeria, and ICF Macro, 2009)	0.19 [0.14; 0.26]
Nigeria – (NPC/Nigeria, and ICF International, 2014)	0.31 [0.27; 0.35]
Nigeria – (NPC/Nigeria, and ICF, 2019)	0.31 [0.26; 0.37]
Rwanda – (INSR/Rwanda, and ORC Macro, 2006)	0.36 [0.28; 0.46]
Rwanda – (NISR/Rwanda, MOH/Rwanda, and ICF International, 2012)	0.19 [0.16; 0.24]
Rwanda – (NISR/Rwanda, MFEP/Rwanda, MOH/Rwanda, and ICF International, 2016)	0.35 [0.25; 0.49]
Senegal – (ANSD/Sénégal, and ICF, 2018)	0.46 [0.33; 0.65]
Sierra Leone – (SSL/Sierra Leone, and ICF International, 2014)	0.14 [0.11; 0.17]
South Sudan – (Ellsberg et al., 2018)	0.50 [0.35; 0.72]
Sao Tome and Principe – (INE/São Tomé, and ICF Macro, 2010)	0.20 [0.14; 0.28]
Chad – (INSEED/Tchad, MSP/Tchad, and ICF International, 2016)	0.35 [0.27; 0.44]
Togo – (MPDAT/Togo, MS/Togo, and ICF International, 2015)	0.35 [0.27; 0.44]
Tanzania – (MoHCDGEC/Tanzania Mainland, MoH/Zanzibar, NBS/Tanzania, OCGS/Zanzibar, and ICF, 2016)	0.54 [0.39; 0.74]
Tanzania – (MoHCDGEC/Tanzania Mainland, MoH/Zanzibar, NBS/Tanzania, OCGS/Zanzibar, and ICF, 2016)	0.55 [0.41; 0.74]
Tanzania – (NBS/Tanzania, and ICF Macro, 2011)	0.28 [0.24; 0.32]
Tanzania – (MoHCDGEC/Tanzania, MoH/Zanzibar, NBS/Tanzania, OCGS/Zanzibar, and ICF, 2016)	0.28 [0.24; 0.32]
Uganda – (UBOS/Uganda, and Macro International, 2007)	0.41 [0.32; 0.52]
Uganda – (Ministry of Health/Uganda, and ICF International, 2012)	0.50 [0.39; 0.64]
Uganda – (Uganda Bureau of Statistics, 2018)	0.47 [0.41; 0.53]
South Africa – (NDH/South Africa, and ICF, 2019)	0.25 [0.19; 0.35]
Zambia – (CSO/Zambia, MOH/Zambia, TDRC/Zambia, and University of Zambia, 2009)	0.27 [0.23; 0.32]
Zambia – (CSO/Zambia, MOH/Zambia, University of Zambia THVL, DPS, TDRC/Zambia, and ICF International, 2015)	0.41 [0.37; 0.47]
Zimbabwe – (CSO/Zimbabwe, and Macro International, 2007)	0.34 [0.29; 0.41]
Zimbabwe – (ZIMSTAT/Zimbabwe, and ICF International, 2012)	0.46 [0.27; 0.79]
Zimbabwe – (ZIMSTAT/Zimbabwe, and ICF International, 2012)	0.34 [0.25; 0.48]
Zimbabwe – (ZIMSTAT/Zimbabwe, and ICF International, 2012)	0.38 [0.18; 0.80]
Zimbabwe – (ZIMSTAT/Zimbabwe, and ICF International, 2012)	0.34 [0.18; 0.63]
Zimbabwe – (ZIMSTAT/Zimbabwe, and ICF International, 2012)	0.49 [0.31; 0.78]
Zimbabwe – (ZIMSTAT/Zimbabwe, and ICF International, 2012)	0.46 [0.31; 0.68]
Zimbabwe – (ZIMSTAT/Zimbabwe, and ICF International, 2012)	0.41 [0.30; 0.56]
Zimbabwe – (ZNSA/Zimbabwe, and ICF International, 2016)	0.41 [0.35; 0.49]
Total	0.32 [0.29; 0.35]
Heterogeneity: $\chi^2_{76} = 851.86$ ($P < .001$), $I^2 = 91\%$	
Total	0.31 [0.29; 0.33]
Heterogeneity: $\chi^2_{229} = 3830.92$ ($P < .001$), $I^2 = 94\%$	
Residual heterogeneity: $\chi^2_{223} = 3395.41$ ($P < .001$), $I^2 = 93\%$	

Figure S7. Forest plot of the random effect meta-analysis for lifetime intimate partner violence (IPV) when all women are surveyed, as compared to ever-partnered women.

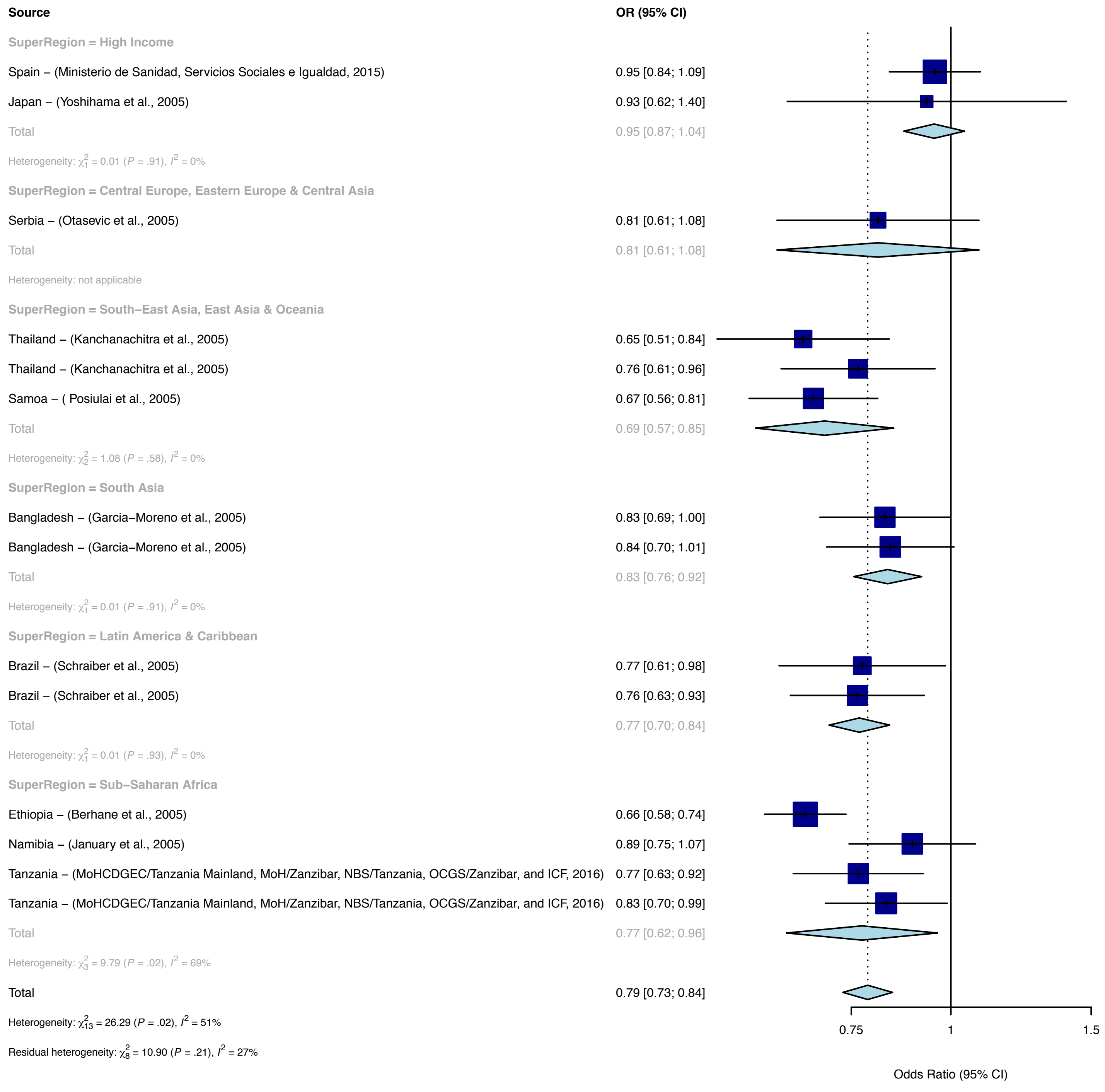
Source
OR (95% CI)
SuperRegion = High Income
Spain – (Ministerio de Sanidad, Servicios Sociales e Igualdad, 2015) 0.95 [0.84; 1.09]
Japan – (Yoshihama et al., 2005) 0.93 [0.62; 1.40]
Total 0.95 [0.87; 1.04]
Heterogeneity: χ^2_1 = 0.01 (P = .91), I^2 = 0%
SuperRegion = Central Europe, Eastern Europe & Central Asia
Serbia – (Otasevic et al., 2005) 0.81 [0.61; 1.08]
Total 0.81 [0.61; 1.08]
Heterogeneity: not applicable
SuperRegion = South–East Asia, East Asia & Oceania
Thailand – (Kanchanachitra et al., 2005) 0.65 [0.51; 0.84]
Thailand – (Kanchanachitra et al., 2005) 0.76 [0.61; 0.96]
Samoa – (Posiulai et al., 2005) 0.67 [0.56; 0.81]
Total 0.69 [0.57; 0.85]
Heterogeneity: χ^2_2 = 1.08 (P = .58), I^2 = 0%
SuperRegion = South Asia
Bangladesh – (Garcia–Moreno et al., 2005) 0.83 [0.69; 1.00]
Bangladesh – (Garcia–Moreno et al., 2005) 0.84 [0.70; 1.01]
Total 0.83 [0.76; 0.92]
Heterogeneity: χ^2_1 = 0.01 (P = .91), I^2 = 0%
SuperRegion = Latin America & Caribbean
Brazil – (Schraiber et al., 2005) 0.77 [0.61; 0.98]
Brazil – (Schraiber et al., 2005) 0.76 [0.63; 0.93]
Total 0.77 [0.70; 0.84]
Heterogeneity: χ^2_1 = 0.01 (P = .93), I^2 = 0%
SuperRegion = Sub–Saharan Africa
Ethiopia – (Berhane et al., 2005) 0.66 [0.58; 0.74]
Namibia – (January et al., 2005) 0.89 [0.75; 1.07]
Tanzania – (MoHCDGEC/Tanzania Mainland, MoH/Zanzibar, NBS/Tanzania, OCGS/Zanzibar, and ICF, 2016) 0.77 [0.63; 0.92]
Tanzania – (MoHCDGEC/Tanzania Mainland, MoH/Zanzibar, NBS/Tanzania, OCGS/Zanzibar, and ICF, 2016) 0.83 [0.70; 0.99]
Total 0.77 [0.62; 0.96]
Heterogeneity: χ^2_3 = 9.79 (P = .02), I^2 = 69%
Total 0.79 [0.73; 0.84]
Heterogeneity: χ^2_{13} = 26.29 (P = .02), I^2 = 51%
Residual heterogeneity: χ^2_8 = 10.90 (P = .21), I^2 = 27%
0.75
1
1.5
Odds Ratio (95% CI)

Figure S8. Forest plot of the random effect meta-analysis for lifetime intimate partner violence (IPV) when currently-partnered women are surveyed, as compared to ever-partnered women.

Source	OR (95% CI)
**SuperRegion = North Africa & Middle East**	
Afghanistan – (CSO/Afghanistan, MPH/Afghanistan, and ICF, 2017)	1.00 [0.96; 1.04]
Egypt – (MHP/Egypt and ICF International, 2015)	0.94 [0.87; 1.02]
Jordan – (DoS/Jordan, and Macro International, 2008)	0.91 [0.81; 1.02]
Jordan – (DoS/Jordan, and ICF International, 2013)	0.94 [0.87; 1.02]
Jordan – (DoS/Jordan, and ICF, 2019)	0.88 [0.81; 0.96]
Total	0.94 [0.89; 1.00]
Heterogeneity: $\chi^2_4 = 8.33$ ($P = .08$), $I^2 = 52\%$	
**SuperRegion = Sub–Saharan Africa**	
Angola – (INE/Angola, MINSA/Angola, and ICF, 2017)	0.97 [0.90; 1.04]
Burundi – (DHS, 2016)	0.93 [0.87; 0.99]
Benin – (INSAE/Bénin, and ICF, 2019)	0.97 [0.88; 1.07]
Burkina Faso – (DHS, 2010)	0.93 [0.85; 1.02]
Cote d'Ivoire – (DHS, 2012)	0.98 [0.89; 1.07]
Cameroon – (INS/Cameroun, and ORC Macro, 2005)	0.98 [0.88; 1.10]
Cameroon – (INS/Cameroun, and ICF International, 2012)	0.95 [0.86; 1.03]
Democratic Republic of the Congo – (MP/Congo, and Macro International, 2008)	0.99 [0.89; 1.11]
Democratic Republic of the Congo – (MPSMRM/Congo, MSP/Congo, and ICF International, 2014)	0.94 [0.88; 1.02]
Comoros – (DGSP/Comores, and ICF International, 2014)	0.95 [0.77; 1.17]
Ethiopia – (CSA/Ethiopia, and ICF, 2016)	0.93 [0.85; 1.02]
Gabon – (DGS/Gabon, and ICF International, 2013)	0.92 [0.84; 1.01]
Ghana – (GSS, GHS, and Macro International, 2009)	0.88 [0.75; 1.03]
Gambia – (GBOS, and ICF International, 2014)	0.94 [0.83; 1.05]
Kenya – (CBS/Kenya, MOH/Kenya, and ORC Macro, 2004)	0.94 [0.86; 1.02]
Kenya – (KNBS/Kenya, NACC/Kenya, NASCP/Kenya, MPHS/Kenya, and KMRI, 2010)	0.89 [0.82; 0.97]
Kenya – (KNBS/Kenya, MOH/Kenya, NACC/Kenya, KMRI/Kenya, NCPD/Kenya, 2015)	0.88 [0.80; 0.96]
Liberia – (LISGIS/Liberia, MOHSW/Liberia, NACP/Liberia, and Macro International, 2008)	0.93 [0.84; 1.02]
Mali – (Samaké et al., 2007)	0.98 [0.92; 1.06]
Mali – (CPS/SSDSPF/Mali, INSTAT/Mali, INFO–STAT/Mali, and ICF International, 2014)	1.00 [0.90; 1.11]
Mali – (INSTAT/Mali, CPSSSD, and ICF, 2019)	1.01 [0.91; 1.11]
Moçambique – (MISAU/Moçambique, INE/Moçambique, and ICF International, 2013)	0.93 [0.86; 1.01]
Moçambique – (INS, INE, PNCM/Moçambique and ICF, 2019)	0.95 [0.83; 1.09]
Malawi – (NSO/Malawi, and ORC Macro, 2005)	0.93 [0.86; 0.99]
Malawi – (NSO/Malawi, and ICF Macro, 2011)	0.88 [0.80; 0.96]
Malawi – (NSO/Malawi, and ICF, 2017)	0.91 [0.84; 0.99]
Namibia – (MoHSS/Namibia, and ICF International, 2014)	0.93 [0.78; 1.11]
Nigeria – (NPC/Nigeria, and ICF Macro, 2009)	0.94 [0.89; 0.99]
Nigeria – (NPC/Nigeria, and ICF International, 2014)	0.93 [0.89; 0.98]
Nigeria – (NPC/Nigeria, and ICF, 2019)	0.94 [0.87; 1.01]
Rwanda – (INSR/Rwanda, and ORC Macro, 2006)	0.89 [0.79; 1.00]
Rwanda – (NISR/Rwanda, MOH/Rwanda, and ICF International, 2012)	0.91 [0.82; 1.00]
Rwanda – (DHS, 2015)	0.90 [0.79; 1.04]
Senegal – (ANSD/Sénégal, and ICF, 2018)	0.95 [0.83; 1.08]
Sierra Leone – (SSL/Sierra Leone, and ICF International, 2014)	0.99 [0.91; 1.08]
Sao Tome and Principe – (INE/São Tomé, and ICF Macro, 2010)	0.99 [0.84; 1.15]
Chad – (INSEED/Tchad, MSP/Tchad, and ICF International, 2016)	0.92 [0.83; 1.02]
Togo – (MPDAT/Togo, MS/Togo, and ICF International, 2015)	0.94 [0.86; 1.03]
Tanzania – (NBS/Tanzania, and ICF Macro, 2011)	0.87 [0.81; 0.95]
Tanzania – (MoHCDGEC/Tanzania, MoH/Zanzibar, NBS/Tanzania, OCGS/Zanzibar, and ICF, 2016)	0.87 [0.81; 0.93]
Uganda – (UBOS/Uganda, and Macro International, 2007)	0.96 [0.83; 1.10]
Uganda – (DHS, 2011)	0.95 [0.83; 1.10]
Uganda – (UBOS/Uganda, and ICF, 2018)	0.91 [0.85; 0.97]
South Africa – (NDH/South Africa, and ICF, 2019)	0.87 [0.75; 1.00]
Zambia – (CSO/Zambia, MOH/Zambia, TDRC/Zambia, and University of Zambia, 2009)	0.92 [0.84; 1.01]
Zambia – (CSO/Zambia, MOH/Zambia, University of Zambia THVL, DPS, TDRC/Zambia, and ICF International, 2015)	0.93 [0.88; 0.99]
Zimbabwe – (CSO/Zimbabwe, and Macro International, 2007)	0.93 [0.85; 1.01]
Zimbabwe – (ZIMSTAT/Zimbabwe, and ICF International, 2012)	0.93 [0.86; 1.01]
Zimbabwe – (ZNSA/Zimbabwe, and ICF International, 2016)	0.94 [0.87; 1.02]
Total	0.93 [0.92; 0.94]
Heterogeneity: $\chi^2_{48} = 29.47$ ($P = .98$), $I^2 = 0\%$	
**SuperRegion = Central Europe, Eastern Europe & Central Asia**	
Armenia – (DHS, 2016)	0.67 [0.55; 0.81]
Azerbaijan – (SSC/Azerbaijan, and Macro International, 2008)	0.80 [0.70; 0.91]
Kyrgyz Republic – (NSC, MOH/Kyrgyz Republic NSC, and ICF International, 2013)	0.86 [0.78; 0.95]
Moldova – (NCPM/Moldova, and ORC Macro, 2006)	0.82 [0.74; 0.90]
Tajikistan – (SA/Tajikistan, MOH/Tajikistan, and ICF International, 2013)	0.95 [0.85; 1.05]
Tajikistan – (SA, MOH/Tajikistan, and ICF, 2018)	0.94 [0.86; 1.03]
Ukraine – (UCSR, SSC/Ukraine, MOH/Ukraine, and Macro International, 2008)	0.57 [0.47; 0.70]
Total	0.81 [0.69; 0.95]
Heterogeneity: $\chi^2_6 = 31.55$ ($P < .001$), $I^2 = 81\%$	
**SuperRegion = Latin America & Caribbean**	
Colombia – (DHS, 2005)	0.79 [0.76; 0.83]
Colombia – (Profamilia/Colombia, 2005)	0.77 [0.74; 0.80]
Colombia – (MSPSP, 2015)	0.74 [0.71; 0.77]
Dominican Republic – (Achécar et al., 2003)	0.78 [0.71; 0.85]
Dominican Republic – (CESDEM/Republica Dominicana and Macro International, 2008)	0.82 [0.75; 0.90]
Dominican Republic – (CESDEM/Republica Dominicana, and ICF International, 2014)	0.82 [0.75; 0.90]
Guatemala – (DHS, 2015)	0.79 [0.73; 0.87]
Honduras – (SS/Honduras, INE/Honduras, and Macro International, 2006)	1.08 [1.01; 1.16]
Honduras – (SS/Honduras, INE/Honduras, and ICF International, 2013)	0.84 [0.79; 0.89]
Haïti – (Cayemittes et al., 2001)	0.84 [0.74; 0.96]
Haiti – (DHS, 2006)	0.96 [0.83; 1.10]
Haïti – (Cayemittes et al., 2013)	0.98 [0.90; 1.06]
Haïti – (IHE/Haïti, and ICF, 2018)	0.93 [0.84; 1.02]
Total	0.85 [0.79; 0.91]
Heterogeneity: $\chi^2_{12} = 136.79$ ($P < .001$), $I^2 = 91\%$	
**SuperRegion = South Asia**	
India – (DHS, 2006)	0.98 [0.96; 1.00]
Nepal – (MOHP/Nepal, New ERA/Nepal, and ICF International, 2012)	0.99 [0.89; 1.10]
Nepal – (MOH/Nepal, New ERA/Nepal, and ICF, 2017)	0.96 [0.87; 1.07]
Pakistan – (NIPS/Pakistan, and ICF, 2019)	0.97 [0.87; 1.09]
Total	0.98 [0.97; 0.99]
Heterogeneity: $\chi^2_3 = 0.17$ ($P = .98$), $I^2 = 0\%$	
**SuperRegion = South–East Asia, East Asia & Oceania**	
Cambodia – (NIS/Cambodia, DGH/Cambodia, and ORC Macro, 2001)	0.93 [0.80; 1.09]
Cambodia – (NIPH/Cambodia, NIS/Cambodia, and ORC Macro, 2006)	0.93 [0.78; 1.11]
Cambodia – (NIS/Cambodia, DGH/Cambodia, and ICF International, 2015)	0.92 [0.82; 1.04]
Maldives – (DHS, 2016)	0.78 [0.68; 0.89]
Myanmar – (DHS, 2016)	0.88 [0.77; 1.01]
Philippines – (NSO/Philippines, and ICF Macro, 2009)	0.93 [0.85; 1.02]
Philippines – (PSA/Philippines, and ICF International, 2014)	0.89 [0.82; 0.97]
Philippines – (PSA/Philippines, and ICF, 2018)	0.91 [0.85; 0.98]
Papua New Guinea – (DHS, 2017)	0.97 [0.88; 1.06]
Timor–leste – (NSD/Timor–Leste, MF/Timor–Leste, and ICF Macro, 2010)	0.98 [0.86; 1.11]
Timor–leste – (GDS, MF/Timor Leste, and ICF, 2018)	1.00 [0.91; 1.10]
Total	0.92 [0.89; 0.96]
Heterogeneity: $\chi^2_{10} = 12.07$ ($P = .28$), $I^2 = 17\%$	
Total	0.91 [0.90; 0.93]

Heterogeneity: $\chi^2_{88} = 460.83$ ($P < .001$), $I^2 = 81\%$

Residual heterogeneity: $\chi^2_{83} = 218.38$ ($P < .001$), $I^2 = 62\%$

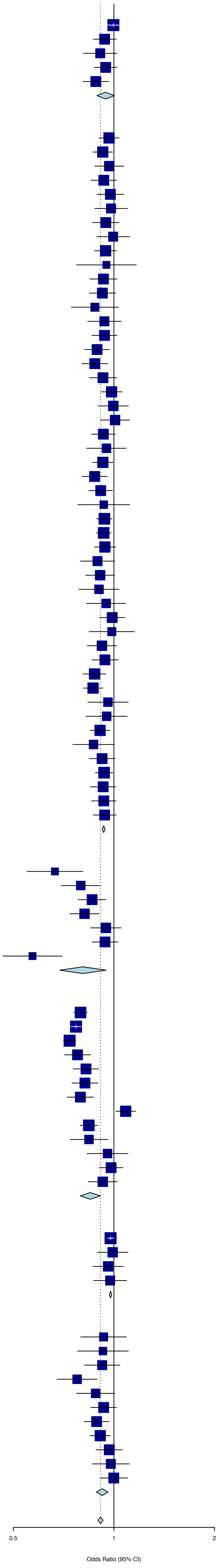

Figure S9. Forest plot of the random effect meta-analysis for past year intimate partner violence (IPV) when currently partnered women are surveyed, as compared to ever-partnered women.

Source	OR (95% CI)
**SuperRegion = North Africa & Middle East**	
Afghanistan – (CSO/Afghanistan, MPH/Afghanistan, and ICF, 2017)	1.01 [0.97; 1.04]
Egypt – (MHP/Egypt and ICF International, 2015)	0.99 [0.90; 1.09]
Jordan – (DoS/Jordan, and Macro International, 2008)	0.98 [0.86; 1.12]
Jordan – (DoS/Jordan, and ICF International, 2013)	1.04 [0.94; 1.14]
Jordan – (DoS/Jordan, and ICF, 2019)	0.97 [0.88; 1.07]
Total	1.00 [0.98; 1.02]
Heterogeneity: $\chi^2_4 = 1.04$ ($P = .90$), $I^2 = 0\%$	
**SuperRegion = Sub–Saharan Africa**	
Angola – (INE/Angola, MINSA/Angola, and ICF, 2017)	0.99 [0.92; 1.07]
Burundi – (DHS, 2016)	1.14 [1.06; 1.23]
Benin – (INSAE/Bénin, and ICF, 2019)	0.98 [0.87; 1.11]
Burkina Faso – (DHS, 2010)	0.99 [0.90; 1.09]
Cote d'Ivoire – (DHS, 2012)	1.05 [0.96; 1.16]
Cameroon – (INS/Cameroun, and ORC Macro, 2005)	1.06 [0.95; 1.18]
Cameroon – (INS/Cameroun, and ICF International, 2012)	1.05 [0.96; 1.16]
Democratic Republic of the Congo – (MP/Congo, and Macro International, 2008)	1.17 [1.05; 1.31]
Democratic Republic of the Congo – (MPSMRM/Congo, MSP/Congo, and ICF International, 2014)	0.99 [0.92; 1.07]
Comoros – (DGSP/Comores, and ICF International, 2014)	0.92 [0.70; 1.21]
Ethiopia – (CSA/Ethiopia, and ICF, 2016)	1.01 [0.91; 1.12]
Gabon – (DGS/Gabon, and ICF International, 2013)	1.02 [0.93; 1.12]
Ghana – (GSS, GHS, and Macro International, 2009)	0.97 [0.81; 1.15]
Gambia – (GBOS, and ICF International, 2014)	0.97 [0.81; 1.16]
Kenya – (CBS/Kenya, MOH/Kenya, and ORC Macro, 2004)	1.06 [0.97; 1.15]
Kenya – (KNBS/Kenya, NACC/Kenya, NASCP/Kenya, MPHS/Kenya, and KMRI, 2010)	0.93 [0.85; 1.02]
Kenya – (KNBS/Kenya, MOH/Kenya, NACC/Kenya, KMRI/Kenya, NCPD/Kenya, 2015)	1.01 [0.91; 1.11]
Liberia – (LISGIS/Liberia, MOHSW/Liberia, NACP/Liberia, and Macro International, 2008)	0.97 [0.88; 1.07]
Mali – (Samaké et al., 2007)	1.01 [0.94; 1.08]
Mali – (CPS/SSDSPF/Mali, INSTAT/Mali, INFO–STAT/Mali, and ICF International, 2014)	1.00 [0.89; 1.12]
Mali – (INSTAT/Mali, CPSSSD, and ICF, 2019)	1.01 [0.90; 1.14]
Moçambique – (MISAU/Moçambique, INE/Moçambique, and ICF International, 2013)	0.98 [0.90; 1.06]
Moçambique – (INS, INE, PNCM/Moçambique and ICF, 2019)	0.98 [0.84; 1.15]
Malawi – (NSO/Malawi, and ORC Macro, 2005)	0.99 [0.92; 1.06]
Malawi – (NSO/Malawi, and ICF Macro, 2011)	0.96 [0.88; 1.06]
Malawi – (NSO/Malawi, and ICF, 2017)	1.00 [0.91; 1.09]
Namibia – (MoHSS/Namibia, and ICF International, 2014)	0.98 [0.80; 1.19]
Nigeria – (NPC/Nigeria, and ICF Macro, 2009)	0.99 [0.94; 1.05]
Nigeria – (NPC/Nigeria, and ICF International, 2014)	0.97 [0.91; 1.03]
Nigeria – (NPC/Nigeria, and ICF, 2019)	0.98 [0.90; 1.07]
Rwanda – (INSR/Rwanda, and ORC Macro, 2006)	1.11 [0.99; 1.25]
Rwanda – (NISR/Rwanda, MOH/Rwanda, and ICF International, 2012)	1.15 [1.04; 1.27]
Rwanda – (DHS, 2015)	1.04 [0.89; 1.23]
Senegal – (ANSD/Sénégal, and ICF, 2018)	0.94 [0.79; 1.11]
Sierra Leone – (SSL/Sierra Leone, and ICF International, 2014)	1.03 [0.93; 1.13]
Sao Tome and Principe – (INE/São Tomé, and ICF Macro, 2010)	0.98 [0.84; 1.15]
Chad – (INSEED/Tchad, MSP/Tchad, and ICF International, 2016)	0.96 [0.85; 1.08]
Togo – (MPDAT/Togo, MS/Togo, and ICF International, 2015)	1.00 [0.89; 1.12]
Tanzania – (NBS/Tanzania, and ICF Macro, 2011)	0.95 [0.88; 1.03]
Tanzania – (MoHCDGEC/Tanzania, MoH/Zanzibar, NBS/Tanzania, OCGS/Zanzibar, and ICF, 2016)	0.99 [0.92; 1.06]
Uganda – (UBOS/Uganda, and Macro International, 2007)	1.18 [1.02; 1.35]
Uganda – (DHS, 2011)	1.15 [1.00; 1.34]
Uganda – (UBOS/Uganda, and ICF, 2018)	1.05 [0.98; 1.13]
South Africa – (NDH/South Africa, and ICF, 2019)	0.99 [0.82; 1.20]
Zambia – (CSO/Zambia, MOH/Zambia, TDRC/Zambia, and University of Zambia, 2009)	0.99 [0.91; 1.09]
Zambia – (CSO/Zambia, MOH/Zambia, University of Zambia THVL, DPS, TDRC/Zambia, and ICF International, 2015)	1.07 [1.00; 1.14]
Zimbabwe – (CSO/Zimbabwe, and Macro International, 2007)	1.11 [1.01; 1.22]
Zimbabwe – (ZIMSTAT/Zimbabwe, and ICF International, 2012)	1.07 [0.98; 1.17]
Zimbabwe – (ZNSA/Zimbabwe, and ICF International, 2016)	1.06 [0.96; 1.17]
Total	1.02 [1.00; 1.04]
Heterogeneity: $\chi^2_{48} = 61.2$ ($P = .10$), $I^2 = 22\%$	
**SuperRegion = Central Europe, Eastern Europe & Central Asia**	
Armenia – (DHS, 2016)	0.73 [0.55; 0.96]
Azerbaijan – (SSC/Azerbaijan, and Macro International, 2008)	0.90 [0.77; 1.04]
Kyrgyz Republic – (NSC, MOH/Kyrgyz Republic NSC, and ICF International, 2013)	0.99 [0.88; 1.10]
Moldova – (NCPM/Moldova, and ORC Macro, 2006)	0.82 [0.74; 0.90]
Tajikistan – (SA/Tajikistan, MOH/Tajikistan, and ICF International, 2013)	0.97 [0.86; 1.09]
Tajikistan – (SA, MOH/Tajikistan, and ICF, 2018)	0.97 [0.88; 1.07]
Ukraine – (UCSR, SSC/Ukraine, MOH/Ukraine, and Macro International, 2008)	0.68 [0.54; 0.84]
Total	0.88 [0.78; 1.00]
Heterogeneity: $\chi^2_6 = 18.91$ ($P = .004$), $I^2 = 68\%$	
**SuperRegion = Latin America & Caribbean**	
Colombia – (DHS, 2005)	0.79 [0.76; 0.83]
Colombia – (Profamilia/Colombia, 2005)	0.77 [0.74; 0.80]
Colombia – (MSPSP, 2015)	0.74 [0.71; 0.77]
Dominican Republic – (Achécar et al., 2003)	0.78 [0.71; 0.85]
Dominican Republic – (CESDEM/Republica Dominicana and Macro International, 2008)	0.93 [0.84; 1.03]
Dominican Republic – (CESDEM/Republica Dominicana, and ICF International, 2014)	0.84 [0.75; 0.94]
Guatemala – (DHS, 2015)	1.00 [0.88; 1.13]
Honduras – (SS/Honduras, INE/Honduras, and Macro International, 2006)	
Honduras – (SS/Honduras, INE/Honduras, and ICF International, 2013)	1.02 [0.94; 1.11]
Haïti – (Cayemittes et al., 2001)	0.84 [0.74; 0.96]
Haiti – (DHS, 2006)	1.00 [0.86; 1.16]
Haïti – (Cayemittes et al., 2013)	1.07 [0.97; 1.18]
Haïti – (IHE/Haïti, and ICF, 2018)	1.06 [0.94; 1.21]
Total	0.89 [0.81; 0.97]
Heterogeneity: $\chi^2_{11} = 136.39$ ($P < .001$), $I^2 = 92\%$	
**SuperRegion = South Asia**	
India – (DHS, 2006)	1.06 [1.03; 1.09]
Nepal – (MOHP/Nepal, New ERA/Nepal, and ICF International, 2012)	1.03 [0.90; 1.18]
Nepal – (MOH/Nepal, New ERA/Nepal, and ICF, 2017)	1.01 [0.87; 1.16]
Pakistan – (NIPS/Pakistan, and ICF, 2019)	1.03 [0.90; 1.18]
Total	1.06 [1.04; 1.08]
Heterogeneity: $\chi^2_3 = 0.66$ ($P = .88$), $I^2 = 0\%$	
**SuperRegion = South–East Asia, East Asia & Oceania**	
Cambodia – (NIS/Cambodia, DGH/Cambodia, and ORC Macro, 2001)	0.93 [0.80; 1.09]
Cambodia – (NIPH/Cambodia, NIS/Cambodia, and ORC Macro, 2006)	1.06 [0.86; 1.30]
Cambodia – (NIS/Cambodia, DGH/Cambodia, and ICF International, 2015)	0.99 [0.85; 1.15]
Maldives – (DHS, 2016)	0.90 [0.72; 1.12]
Myanmar – (DHS, 2016)	0.96 [0.82; 1.13]
Philippines – (NSO/Philippines, and ICF Macro, 2009)	1.00 [0.90; 1.12]
Philippines – (PSA/Philippines, and ICF International, 2014)	0.98 [0.87; 1.11]
Philippines – (PSA/Philippines, and ICF, 2018)	1.01 [0.90; 1.12]
Papua New Guinea – (DHS, 2017)	1.05 [0.96; 1.14]
Timor–leste – (NSD/Timor–Leste, MF/Timor–Leste, and ICF Macro, 2010)	1.02 [0.90; 1.17]
Timor–leste – (GDS, MF/Timor Leste, and ICF, 2018)	1.00 [0.91; 1.11]
Total	1.00 [0.98; 1.03]
Heterogeneity: $\chi^2_{10} = 3.47$ ($P = .97$), $I^2 = 0\%$	
**Total**	0.99 [0.97; 1.01]
Heterogeneity: $\chi^2_{87} = 689.14$ ($P < .001$), $I^2 = 87\%$	
Residual heterogeneity: $\chi^2_{82} = 221.67$ ($P < .001$), $I^2 = 63\%$	

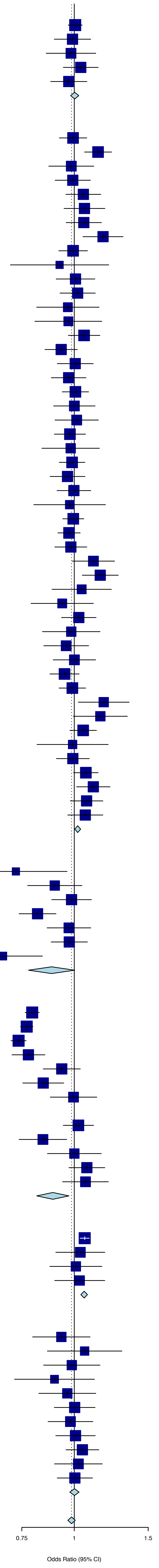

Figure S10. Forest plot of the random effect meta-analysis for lifetime intimate partner violence (IPV) when the reference partner is the current or most recent one, as compared to any current or previous ones.

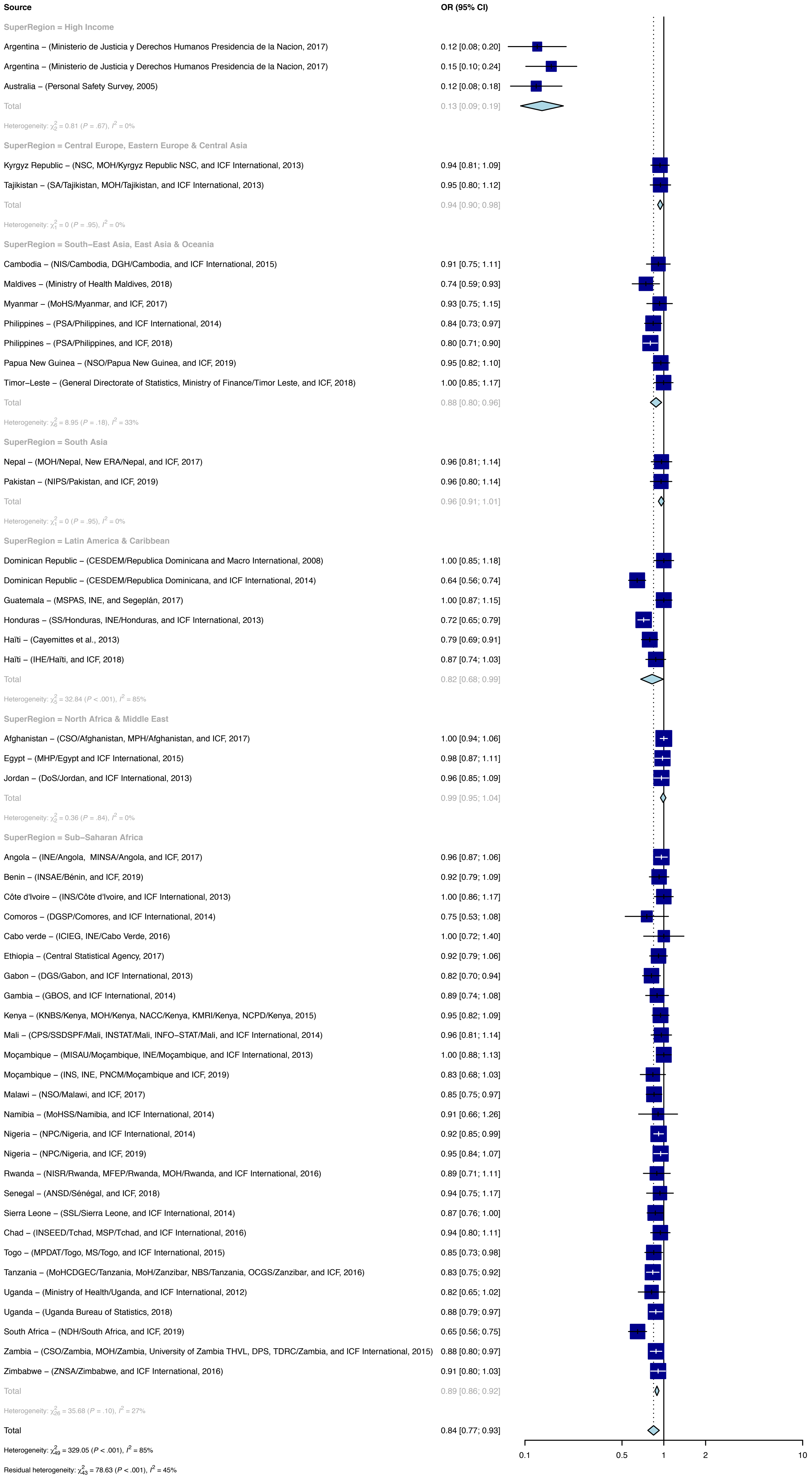
Source
OR (95% CI)
SuperRegion = High Income
Argentina – (Ministerio de Justicia y Derechos Humanos Presidencia de la Nacion, 2017) 0.12 [0.08; 0.20]
Argentina – (Ministerio de Justicia y Derechos Humanos Presidencia de la Nacion, 2017) 0.15 [0.10; 0.24]
Australia – (Personal Safety Survey, 2005) 0.12 [0.08; 0.18]
Total 0.13 [0.09; 0.19]
Heterogeneity: $\chi^2_2 = 0.81$ (P = .67), $I^2 = 0\%$
SuperRegion = Central Europe, Eastern Europe & Central Asia
Kyrgyz Republic – (NSC, MOH/Kyrgyz Republic NSC, and ICF International, 2013) 0.94 [0.81; 1.09]
Tajikistan – (SA/Tajikistan, MOH/Tajikistan, and ICF International, 2013) 0.95 [0.80; 1.12]
Total 0.94 [0.90; 0.98]
Heterogeneity: $\chi^2_1 = 0$ (P = .95), $I^2 = 0\%$
SuperRegion = South–East Asia, East Asia & Oceania
Cambodia – (NIS/Cambodia, DGH/Cambodia, and ICF International, 2015) 0.91 [0.75; 1.11]
Maldives – (Ministry of Health Maldives, 2018) 0.74 [0.59; 0.93]
Myanmar – (MoHS/Myanmar, and ICF, 2017) 0.93 [0.75; 1.15]
Philippines – (PSA/Philippines, and ICF International, 2014) 0.84 [0.73; 0.97]
Philippines – (PSA/Philippines, and ICF, 2018) 0.80 [0.71; 0.90]
Papua New Guinea – (NSO/Papua New Guinea, and ICF, 2019) 0.95 [0.82; 1.10]
Timor–Leste – (General Directorate of Statistics, Ministry of Finance/Timor Leste, and ICF, 2018) 1.00 [0.85; 1.17]
Total 0.88 [0.80; 0.96]
Heterogeneity: $\chi^2_6 = 8.95$ (P = .18), $I^2 = 33\%$
SuperRegion = South Asia
Nepal – (MOH/Nepal, New ERA/Nepal, and ICF, 2017) 0.96 [0.81; 1.14]
Pakistan – (NIPS/Pakistan, and ICF, 2019) 0.96 [0.80; 1.14]
Total 0.96 [0.91; 1.01]
Heterogeneity: $\chi^2_1 = 0$ (P = .95), $I^2 = 0\%$
SuperRegion = Latin America & Caribbean
Dominican Republic – (CESDEM/Republica Dominicana and Macro International, 2008) 1.00 [0.85; 1.18]
Dominican Republic – (CESDEM/Republica Dominicana, and ICF International, 2014) 0.64 [0.56; 0.74]
Guatemala – (MSPAS, INE, and Segeplán, 2017) 1.00 [0.87; 1.15]
Honduras – (SS/Honduras, INE/Honduras, and ICF International, 2013) 0.72 [0.65; 0.79]
Haïti – (Cayemittes et al., 2013) 0.79 [0.69; 0.91]
Haïti – (IHE/Haïti, and ICF, 2018) 0.87 [0.74; 1.03]
Total 0.82 [0.68; 0.99]
Heterogeneity: $\chi^2_5 = 32.84$ (P < .001), $I^2 = 85\%$
SuperRegion = North Africa & Middle East
Afghanistan – (CSO/Afghanistan, MPH/Afghanistan, and ICF, 2017) 1.00 [0.94; 1.06]
Egypt – (MHP/Egypt and ICF International, 2015) 0.98 [0.87; 1.11]
Jordan – (DoS/Jordan, and ICF International, 2013) 0.96 [0.85; 1.09]
Total 0.99 [0.95; 1.04]
Heterogeneity: $\chi^2_2 = 0.36$ (P = .84), $I^2 = 0\%$
SuperRegion = Sub–Saharan Africa
Angola – (INE/Angola, MINSA/Angola, and ICF, 2017) 0.96 [0.87; 1.06]
Benin – (INSAE/Bénin, and ICF, 2019) 0.92 [0.79; 1.09]
Côte d'Ivoire – (INS/Côte d'Ivoire, and ICF International, 2013) 1.00 [0.86; 1.17]
Comoros – (DGSP/Comores, and ICF International, 2014) 0.75 [0.53; 1.08]
Cabo verde – (ICIEG, INE/Cabo Verde, 2016) 1.00 [0.72; 1.40]
Ethiopia – (Central Statistical Agency, 2017) 0.92 [0.79; 1.06]
Gabon – (DGS/Gabon, and ICF International, 2013) 0.82 [0.70; 0.94]
Gambia – (GBOS, and ICF International, 2014) 0.89 [0.74; 1.08]
Kenya – (KNBS/Kenya, MOH/Kenya, NACC/Kenya, KMRI/Kenya, NCPD/Kenya, 2015) 0.95 [0.82; 1.09]
Mali – (CPS/SSDSPF/Mali, INSTAT/Mali, INFO–STAT/Mali, and ICF International, 2014) 0.96 [0.81; 1.14]
Moçambique – (MISAU/Moçambique, INE/Moçambique, and ICF International, 2013) 1.00 [0.88; 1.13]
Moçambique – (INS, INE, PNCM/Moçambique and ICF, 2019) 0.83 [0.68; 1.03]
Malawi – (NSO/Malawi, and ICF, 2017) 0.85 [0.75; 0.97]
Namibia – (MoHSS/Namibia, and ICF International, 2014) 0.91 [0.66; 1.26]
Nigeria – (NPC/Nigeria, and ICF International, 2014) 0.92 [0.85; 0.99]
Nigeria – (NPC/Nigeria, and ICF, 2019) 0.95 [0.84; 1.07]
Rwanda – (NISR/Rwanda, MFEP/Rwanda, MOH/Rwanda, and ICF International, 2016) 0.89 [0.71; 1.11]
Senegal – (ANSD/Sénégal, and ICF, 2018) 0.94 [0.75; 1.17]
Sierra Leone – (SSL/Sierra Leone, and ICF International, 2014) 0.87 [0.76; 1.00]
Chad – (INSEED/Tchad, MSP/Tchad, and ICF International, 2016) 0.94 [0.80; 1.11]
Togo – (MPDAT/Togo, MS/Togo, and ICF International, 2015) 0.85 [0.73; 0.98]
Tanzania – (MoHCDGEC/Tanzania, MoH/Zanzibar, NBS/Tanzania, OCGS/Zanzibar, and ICF, 2016) 0.83 [0.75; 0.92]
Uganda – (Ministry of Health/Uganda, and ICF International, 2012) 0.82 [0.65; 1.02]
Uganda – (Uganda Bureau of Statistics, 2018) 0.88 [0.79; 0.97]
South Africa – (NDH/South Africa, and ICF, 2019) 0.65 [0.56; 0.75]
Zambia – (CSO/Zambia, MOH/Zambia, University of Zambia THVL, DPS, TDRC/Zambia, and ICF International, 2015) 0.88 [0.80; 0.97]
Zimbabwe – (ZNSA/Zimbabwe, and ICF International, 2016) 0.91 [0.80; 1.03]
Total 0.89 [0.86; 0.92]
Heterogeneity: $\chi^2_{26} = 35.68$ (P = .10), $I^2 = 27\%$
Total 0.84 [0.77; 0.93]
Heterogeneity: $\chi^2_{49} = 329.05$ (P < .001), $I^2 = 85\%$
Residual heterogeneity: $\chi^2_{43} = 78.63$ (P < .001), $I^2 = 45\%$
0.1
0.5
1
2
10

Figure S11. Forest plot of the random effect meta-analysis for past year intimate partner violence (IPV) when the reference partner is the current or most recent one, as compared to any current or previous ones.

Source	OR (95% CI)
**SuperRegion = High Income**	
Argentina – (Ministerio de Justicia y Derechos Humanos Presidencia de la Nacion, 2017)	1.24 [0.59; 2.60]
Argentina – (Ministerio de Justicia y Derechos Humanos Presidencia de la Nacion, 2017)	0.88 [0.43; 1.78]
Australia – (Personal Safety Survey, 2013)	0.55 [0.25; 1.20]
Canada – (Juristat, 2006)	0.59 [0.43; 0.80]
Canada – (Juristat, 2011)	0.67 [0.47; 0.96]
Canada – (Juristat, 2016)	0.72 [0.51; 1.03]
Total	0.68 [0.55; 0.85]
Heterogeneity: $\chi^2_5 = 4.27$ (P = .51), I^2 = 0%	
**SuperRegion = Central Europe, Eastern Europe & Central Asia**	
Kyrgyz Republic – (NSC, MOH/Kyrgyz Republic NSC, and ICF International, 2013)	1.00 [0.84; 1.19]
Tajikistan – (SA, MOH/Tajikistan, and ICF, 2018)	0.88 [0.75; 1.03]
Total	0.93 [0.42; 2.10]
Heterogeneity: $\chi^2_1 = 1.1$ (P = .29), I^2 = 9%	
**SuperRegion = South–East Asia, East Asia & Oceania**	
Myanmar – (MoHS/Myanmar, and ICF, 2017)	1.00 [0.78; 1.29]
Philippines – (PSA/Philippines, and ICF International, 2014)	0.98 [0.80; 1.20]
Philippines – (PSA/Philippines, and ICF, 2018)	0.98 [0.82; 1.18]
Papua New Guinea – (NSO/Papua New Guinea, and ICF, 2019)	1.00 [0.87; 1.16]
Timor–Leste – (General Directorate of Statistics, Ministry of Finance/Timor Leste, and ICF, 2018)	1.00 [0.85; 1.17]
Total	0.99 [0.98; 1.01]
Heterogeneity: $\chi^2_4 = 0.04$ (P > .99), I^2 = 0%	
**SuperRegion = Latin America & Caribbean**	
Dominican Republic – (CESDEM/Republica Dominicana and Macro International, 2008)	1.00 [0.83; 1.20]
Dominican Republic – (CESDEM/Republica Dominicana, and ICF International, 2014)	0.97 [0.82; 1.14]
Honduras – (SS/Honduras, INE/Honduras, and ICF International, 2013)	0.99 [0.87; 1.13]
Haïti – (Cayemittes et al., 2013)	0.99 [0.85; 1.17]
Haïti – (IHE/Haïti, and ICF, 2018)	0.99 [0.81; 1.22]
Total	0.99 [0.97; 1.00]
Heterogeneity: $\chi^2_4 = 0.08$ (P > .99), I^2 = 0%	
**SuperRegion = North Africa & Middle East**	
Jordan – (DoS/Jordan, and ICF International, 2013)	1.00 [0.86; 1.16]
Total	1.00 [0.86; 1.16]
Heterogeneity: not applicable	
**SuperRegion = Sub–Saharan Africa**	
Angola – (INE/Angola, MINSA/Angola, and ICF, 2017)	1.00 [0.89; 1.11]
Burundi – (MPBGP, MSPLS,– ISTEEBU/Burundi, and ICF, 2017)	0.99 [0.88; 1.12]
Benin – (INSAE/Bénin, and ICF, 2019)	0.60 [0.48; 0.74]
Côte d'Ivoire – (INS/Côte d'Ivoire, and ICF International, 2013)	0.95 [0.81; 1.11]
Comoros – (DGSP/Comores, and ICF International, 2014)	1.00 [0.65; 1.54]
Cabo verde – (ICIEG, INE/Cabo Verde, 2016)	1.00 [0.71; 1.41]
Gabon – (DGS/Gabon, and ICF International, 2013)	0.99 [0.84; 1.16]
Gambia – (GBOS, and ICF International, 2014)	1.00 [0.74; 1.34]
Kenya – (CBS/Kenya, MOH/Kenya, and ORC Macro, 2004)	0.98 [0.84; 1.15]
Kenya – (KNBS/Kenya, NACC/Kenya, NASCP/Kenya, MPHS/Kenya, and KMRI, 2010)	0.90 [0.78; 1.04]
Kenya – (KNBS/Kenya, MOH/Kenya, NACC/Kenya, KMRI/Kenya, NCPD/Kenya, 2015)	1.00 [0.85; 1.17]
Mali – (CPS/SSDSPF/Mali, INSTAT/Mali, INFO–STAT/Mali, and ICF International, 2014)	0.99 [0.82; 1.18]
Malawi – (NSO/Malawi, and ICF, 2017)	0.99 [0.86; 1.14]
Namibia – (MoHSS/Namibia, and ICF International, 2014)	1.00 [0.70; 1.42]
Nigeria – (NPC/Nigeria, and ICF International, 2014)	0.99 [0.90; 1.09]
Nigeria – (NPC/Nigeria, and ICF, 2019)	0.99 [0.86; 1.14]
Rwanda – (NISR/Rwanda, MFEP/Rwanda, MOH/Rwanda, and ICF International, 2016)	0.99 [0.76; 1.29]
Senegal – (ANSD/Sénégal, and ICF, 2018)	1.00 [0.76; 1.32]
Sierra Leone – (SSL/Sierra Leone, and ICF International, 2014)	0.99 [0.86; 1.16]
Chad – (INSEED/Tchad, MSP/Tchad, and ICF International, 2016)	0.99 [0.82; 1.20]
Togo – (MPDAT/Togo, MS/Togo, and ICF International, 2015)	1.00 [0.83; 1.21]
Tanzania – (MoHCDGEC/Tanzania, MoH/Zanzibar, NBS/Tanzania, OCGS/Zanzibar, and ICF, 2016)	0.99 [0.89; 1.12]
Uganda – (UBOS/Uganda, and Macro International, 2007)	0.91 [0.73; 1.14]
Uganda – (Ministry of Health/Uganda, and ICF International, 2012)	1.07 [0.84; 1.34]
Uganda – (Uganda Bureau of Statistics, 2018)	0.99 [0.88; 1.11]
Zambia – (CSO/Zambia, MOH/Zambia, TDRC/Zambia, and University of Zambia, 2009)	1.04 [0.90; 1.20]
Zambia – (CSO/Zambia, MOH/Zambia, University of Zambia THVL, DPS, TDRC/Zambia, and ICF International, 2015)	0.99 [0.89; 1.10]
Zimbabwe – (ZNSA/Zimbabwe, and ICF International, 2016)	0.99 [0.86; 1.15]
Total	0.98 [0.95; 1.01]
Heterogeneity: $\chi^2_{27} = 23.21$ (P = .67), I^2 = 0%	
Total	0.97 [0.95; 1.00]
Heterogeneity: $\chi^2_{46} = 44.84$ (P = .52), I^2 = 0%	
Residual heterogeneity: $\chi^2_{41} = 28.70$ (P = .93), I^2 = 0%	

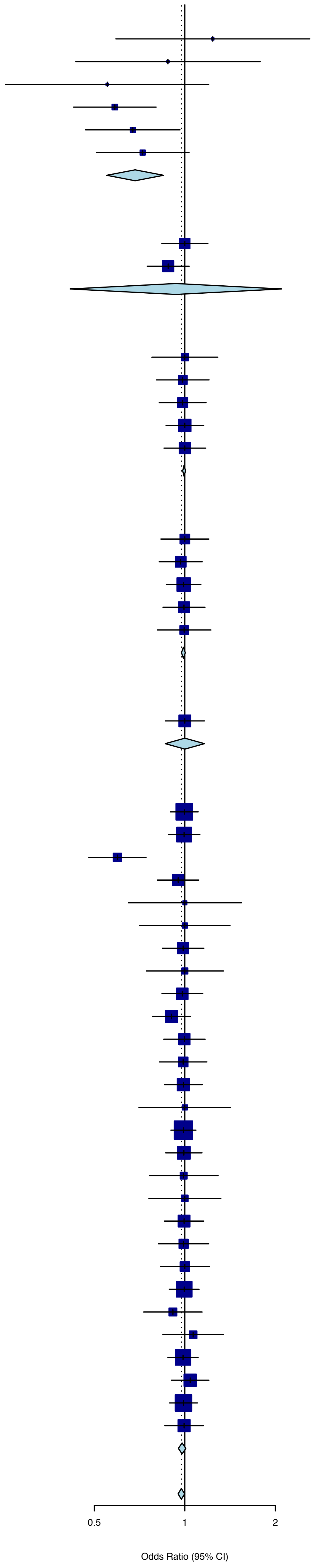

Figure S12. Plot of the data used to estimate, through random-effect logistic regression, the adjustment factor for lifetime intimate partner violence (IPV) in urban regions, as compared to a nationally representative sample.

Source	OR (95% CI)
**SuperRegion = Central Europe, Eastern Europe & Central Asia**	
Albania – (Organisation for Security and Conflict in Europe, 2019)	1.13 [0.92; 1.39]
Azerbaijan – (SSC/Azerbaijan, and Macro International, 2008)	0.96 [0.82; 1.12]
Bosnia and Hezergovina – (Organisation for Security and Conflict in Europe, 2019)	1.00 [0.78; 1.27]
Czechia – (...Buriánek et al., 2013)	0.99 [0.80; 1.21]
Georgia – (UN Women/GEOSTAT, 2018)	1.18 [0.90; 1.55]
Kazakhstan – (Ministry of National Economy of the Republic of Kazakhstan, 2017)	0.99 [0.92; 1.07]
Kyrgyz Republic – (NSC, MOH/Kyrgyz Republic NSC, and ICF International, 2013)	1.06 [0.93; 1.22]
Moldova – (UNW, UNDP, and UNFPA, 2011)	0.72 [0.48; 1.07]
North Macedonia – (Organisation for Security and Conflict in Europe, 2019)	1.11 [0.87; 1.42]
Montenegro – (Organisation for Security and Conflict in Europe, 2019)	1.00 [0.79; 1.28]
Mongolia – (NSO, UNFPA, SDC, Australian Aid, 2018)	1.01 [0.92; 1.10]
Kosovo – (Organisation for Security and Conflict in Europe, 2019)	1.00 [0.76; 1.32]
Tajikistan – (SA/Tajikistan, MOH/Tajikistan, and ICF International, 2013)	1.11 [0.94; 1.31]
Tajikistan – (SA, MOH/Tajikistan, and ICF, 2018)	0.84 [0.72; 0.98]
Ukraine – (UCSR, SSC/Ukraine, MOH/Ukraine, and Macro International, 2008)	0.94 [0.78; 1.13]
Ukraine – (GfK Ukraine, 2014)	0.93 [0.73; 1.19]
**SuperRegion = South–East Asia, East Asia & Oceania**	
Fiji – (Fiji Women's Crisis Centre, 2013)	0.77 [0.67; 0.87]
Cambodia – (NIS/Cambodia, DGH/Cambodia, and ORC Macro, 2001)	0.94 [0.69; 1.27]
Cambodia – (NIPH/Cambodia, NIS/Cambodia, and ORC Macro, 2006)	1.07 [0.76; 1.52]
Cambodia – (NIS/Cambodia, DGH/Cambodia, and ICF International, 2015)	0.70 [0.61; 0.80]
Cambodia – (Ministry of Women's Affairs, 2015)	0.69 [0.61; 0.79]
Maldives – (Ministry of Health Maldives, 2018)	0.90 [0.73; 1.11]
Marshall Islands – (DHS/Marshall Islands, 2008)	1.00 [0.79; 1.25]
Marshall Islands – (Ministry of Internal Affairs, 2014)	0.99 [0.81; 1.22]
Myanmar – (MoHS/Myanmar, and ICF, 2017)	0.86 [0.69; 1.07]
Philippines – (NSO/Philippines, and ICF Macro, 2009)	0.97 [0.87; 1.07]
Philippines – (PSA/Philippines, and ICF International, 2014)	1.02 [0.91; 1.14]
Philippines – (PSA/Philippines, and ICF, 2018)	0.99 [0.90; 1.09]
Papua New Guinea – (NSO/Papua New Guinea, and ICF, 2019)	1.35 [1.10; 1.67]
Timor–leste – (NSD/Timor–Leste, MF/Timor–Leste, and ICF Macro, 2010)	1.58 [1.29; 1.94]
Timor–Leste – (Asia Foundation, 2016)	1.15 [0.97; 1.36]
Timor–Leste – (General Directorate of Statistics, Ministry of Finance/Timor Leste, and ICF, 2018)	0.65 [0.55; 0.77]
Tonga – (Ma'a Fafine mo e Famili, 2012)	1.09 [0.72; 1.65]
**SuperRegion = South Asia**	
Bangladesh – (NIPORT/Bangladesh, Mitra, Associates/Bangladesh, and Macro International, 2009)	0.80 [0.70; 0.92]
Bhutan – (National Commission for Women and Children, 2019)	0.99 [0.77; 1.27]
India – (IIPS/India, and Macro International, 2007)	0.74 [0.71; 0.76]
India – (IIPS/India, and ICF, 2017)	0.76 [0.73; 0.78]
Nepal – (MOHP/Nepal, New ERA/Nepal, and ICF International, 2012)	0.87 [0.73; 1.04]
Nepal – (MOH/Nepal, New ERA/Nepal, and ICF, 2017)	0.95 [0.84; 1.08]
Pakistan – (NIPS/Pakistan, and ICF, 2019)	0.83 [0.71; 0.97]
**SuperRegion = Latin America & Caribbean**	
Colombia – (Profamilia/Colombia, 2000)	1.07 [1.00; 1.15]
Colombia – (Profamilia/Colombia, 2005)	1.03 [0.99; 1.07]
Colombia – (Profamilia/Colombia, 2005)	1.05 [1.01; 1.08]
Dominican Republic – (Achécar et al., 2003)	1.07 [0.98; 1.18]
Dominican Republic – (CESDEM/Republica Dominicana and Macro International, 2008)	0.98 [0.89; 1.07]
Dominican Republic – (CESDEM/Republica Dominicana, and ICF International, 2014)	1.04 [0.94; 1.15]
Guatemala – (MSPAS, 2011)	1.12 [1.04; 1.20]
Guatemala – (MSPAS, INE, and Segeplán, 2017)	1.17 [1.04; 1.32]
Guyana – (Contreras–Urbina et al., 2019)	0.87 [0.65; 1.16]
Honduras – (SS/Honduras, INE/Honduras, and ICF International, 2013)	1.13 [1.05; 1.22]
Haïti – (Cayemittes et al., 2001)	1.02 [0.86; 1.22]
Haïti – (Cayemittes et al., 2007)	0.94 [0.71; 1.25]
Haïti – (Cayemittes et al., 2013)	1.14 [1.02; 1.27]
Haïti – (IHE/Haïti, and ICF, 2018)	1.23 [1.08; 1.41]
Jamaica – (National Family Planning Board, 2010)	1.03 [0.92; 1.14]
Jamaica – (Williams et al., 2018)	1.01 [0.80; 1.28]
Nicaragua – (INIDE MINSA, 2008)	1.17 [1.09; 1.25]
Nicaragua – (INIDE MINSA, 2014)	1.24 [1.15; 1.33]
Panama – (Instituto Conmemorativo Gorgas de Estudios de la Salud, 2011)	1.06 [0.88; 1.28]
Panama – (Instituto Conmemorativo Gorgas de Estudios de la Salud, 2018)	0.91 [0.75; 1.11]
Peru – (INEI/Perú, 2012)	1.04 [0.98; 1.10]
Peru – (INEI/Perú, 2013)	1.03 [0.98; 1.09]
Peru – (INEI/Perú, 2014)	1.03 [0.97; 1.09]
Peru – (INEI/Perú, 2015)	1.02 [0.97; 1.08]
Peru – (INEI/Perú, 2016)	1.03 [0.98; 1.07]
Peru – (INEI/Perú, 2017)	1.04 [0.99; 1.09]
Paraguay – (Centro paraguayo de estudios de población, 2009)	1.17 [1.04; 1.32]
El salvador – (Asociación Demográfica Salvadoreña – ADS/El Salvador, 2008)	1.17 [1.07; 1.28]
Trinidad and Tobago – (Pemberton and Joseph, 2018)	1.00 [0.81; 1.23]
**SuperRegion = North Africa & Middle East**	
Afghanistan – (CSO/Afghanistan, MPH/Afghanistan, and ICF, 2017)	0.66 [0.62; 0.71]
Egypt – (MHP/Egypt and ICF International, 2015)	0.90 [0.80; 1.00]
Egypt – (Duvvury et al., 2015)	0.75 [0.71; 0.80]
Jordan – (DoS/Jordan, and Macro International, 2008)	1.05 [0.93; 1.17]
Jordan – (DoS/Jordan, and ICF International, 2013)	1.04 [0.96; 1.13]
Jordan – (DoS/Jordan, and ICF, 2019)	1.02 [0.93; 1.11]
Turkey – (Ministry of Family and Social Policies, Hacettepe University and NEE Institute of Population Studies, 2009)	0.94 [0.88; 0.99]
Turkey – (Ministry of Family and Social Policies, Hacettepe University and NEE Institute of Population Studies, 2015)	0.98 [0.90; 1.06]
**SuperRegion = Sub–Saharan Africa**	
Angola – (INE/Angola, MINSA/Angola, and ICF, 2017)	1.06 [0.99; 1.14]
Burundi – (MPBGP, MSPLS, ISTEEBU/Burundi, and ICF, 2017)	0.54 [0.46; 0.64]
Benin – (INSAE/Bénin, and ICF, 2019)	0.90 [0.78; 1.03]
Burkina faso – (Institut National de la Statistique et de la Démographie, 2012)	1.36 [1.19; 1.56]
Central African Republic – (Institut Centrafricain des Statistiques, et des Etudes Economiques et Sociales, 2009)	1.40 [1.30; 1.51]
Côte d'Ivoire – (INS/Côte d'Ivoire, and ICF International, 2013)	1.19 [1.05; 1.34]
Cameroon – (INS/Cameroun, and ORC Macro, 2005)	1.11 [0.96; 1.28]
Cameroon – (INS/Cameroun, and ICF International, 2012)	0.96 [0.89; 1.03]
Cabo verde – (Instituto Nacional de Estatística, 2008)	1.18 [0.87; 1.60]
Cabo verde – (ICIEG, INE/Cabo Verde, 2016)	1.11 [0.89; 1.39]
Ethiopia – (Central Statistical Agency, 2017)	0.69 [0.57; 0.83]
Gabon – (DGS/Gabon, and ICF International, 2013)	0.97 [0.88; 1.07]
Ghana – (GSS, GHS, and Macro International, 2009)	1.05 [0.85; 1.29]
Gambia – (GBOS, and ICF International, 2014)	0.84 [0.72; 0.98]
Equatorial Guinea – (PIP/Guinea Ecuatorial, and ICF International, 2012)	0.91 [0.70; 1.17]
Kenya – (CBS/Kenya, MOH/Kenya, and ORC Macro, 2004)	0.75 [0.65; 0.88]
Kenya – (KNBS/Kenya, NACC/Kenya, NASCP/Kenya, MPHS/Kenya, and KMRI, 2010)	0.77 [0.67; 0.89]
Kenya – (KNBS/Kenya, MOH/Kenya, NACC/Kenya, KMRI/Kenya, NCPD/Kenya, 2015)	0.89 [0.79; 1.00]
Liberia – (LISGIS/Liberia, MOHSW/Liberia, NACP/Liberia, and Macro International, 2008)	1.16 [1.02; 1.32]
Mali – (Samaké et al., 2007)	0.87 [0.78; 0.97]
Mali – (CPS/SSDSPF/Mali, INSTAT/Mali, INFO–STAT/Mali, and ICF International, 2014)	0.84 [0.69; 1.01]
Mali – (INSTAT/Mali, CPSSSD, and ICF, 2019)	1.05 [0.89; 1.24]
Moçambique – (MISAU/Moçambique, INE/Moçambique, and ICF International, 2013)	1.23 [1.10; 1.37]
Moçambique – (INS, INE, PNCM/Moçambique and ICF, 2019)	1.43 [1.19; 1.72]
Malawi – (NSO/Malawi, and ORC Macro, 2005)	0.98 [0.85; 1.12]
Malawi – (NSO/Malawi, and ICF Macro, 2011)	1.34 [1.16; 1.55]
Malawi – (NSO/Malawi, and ICF, 2017)	0.92 [0.79; 1.08]
Namibia – (MoHSS/Namibia, and ICF International, 2014)	0.98 [0.77; 1.25]
Nigeria – (NPC/Nigeria, and ICF Macro, 2009)	0.96 [0.89; 1.04]
Nigeria – (NPC/Nigeria, and ICF International, 2014)	1.11 [1.04; 1.19]
Nigeria – (NPC/Nigeria, and ICF, 2019)	0.95 [0.87; 1.05]
Rwanda – (INSR/Rwanda, and ORC Macro, 2006)	0.90 [0.70; 1.15]
Rwanda – (NISR/Rwanda, MFEP/Rwanda, MOH/Rwanda, and ICF International, 2016)	0.78 [0.60; 1.02]
Senegal – (ANSD/Sénégal, and ICF, 2018)	1.05 [0.88; 1.27]
Sierra Leone – (SSL/Sierra Leone, and ICF International, 2014)	1.05 [0.92; 1.19]
Sao Tome and Principe – (INE/São Tomé, and ICF Macro, 2010)	1.10 [0.92; 1.31]
Chad – (INSEED/Tchad, MSP/Tchad, and ICF International, 2016)	0.99 [0.83; 1.18]
Togo – (MPDAT/Togo, MS/Togo, and ICF International, 2015)	0.88 [0.78; 1.00]
Tanzania – (NBS/Tanzania, and ICF Macro, 2011)	0.93 [0.83; 1.05]
Tanzania – (MoHCDGEC/Tanzania, MoH/Zanzibar, NBS/Tanzania, OCGS/Zanzibar, and ICF, 2016)	0.87 [0.79; 0.96]
Uganda – (UBOS/Uganda, and Macro International, 2007)	0.56 [0.42; 0.73]
Uganda – (Ministry of Health/Uganda, and ICF International, 2012)	0.77 [0.59; 0.99]
Uganda – (Uganda Bureau of Statistics, 2018)	0.69 [0.62; 0.77]
South Africa – (NDH/South Africa, and ICF, 2019)	0.98 [0.88; 1.08]
Zambia – (CSO/Zambia, MOH/Zambia, TDRC/Zambia, and University of Zambia, 2009)	1.26 [1.12; 1.42]
Zambia – (CSO/Zambia, MOH/Zambia, University of Zambia THVL, DPS, TDRC/Zambia, and ICF International, 2015)	1.00 [0.92; 1.08]
Zimbabwe – (CSO/Zimbabwe, and Macro International, 2007)	0.79 [0.70; 0.89]
Zimbabwe – (ZIMSTAT/Zimbabwe, and ICF International, 2012)	0.93 [0.83; 1.04]
Zimbabwe – (ZNSA/Zimbabwe, and ICF International, 2016)	0.96 [0.87; 1.07]

Heterogeneity: $\chi^2_{125} = 1467.34$ ($P < .001$), $I^2 = 91\%$

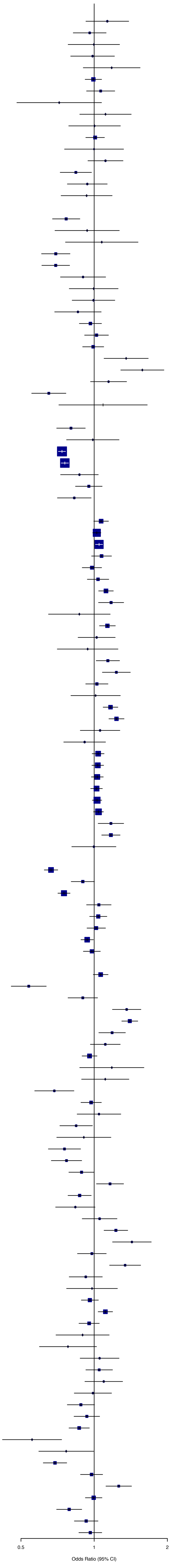

Figure S13. Plot of the data used to estimate, through random-effect logistic regression, the adjustment factor for past year intimate partner violence (IPV) in urban regions, as compared to a nationally representative sample.

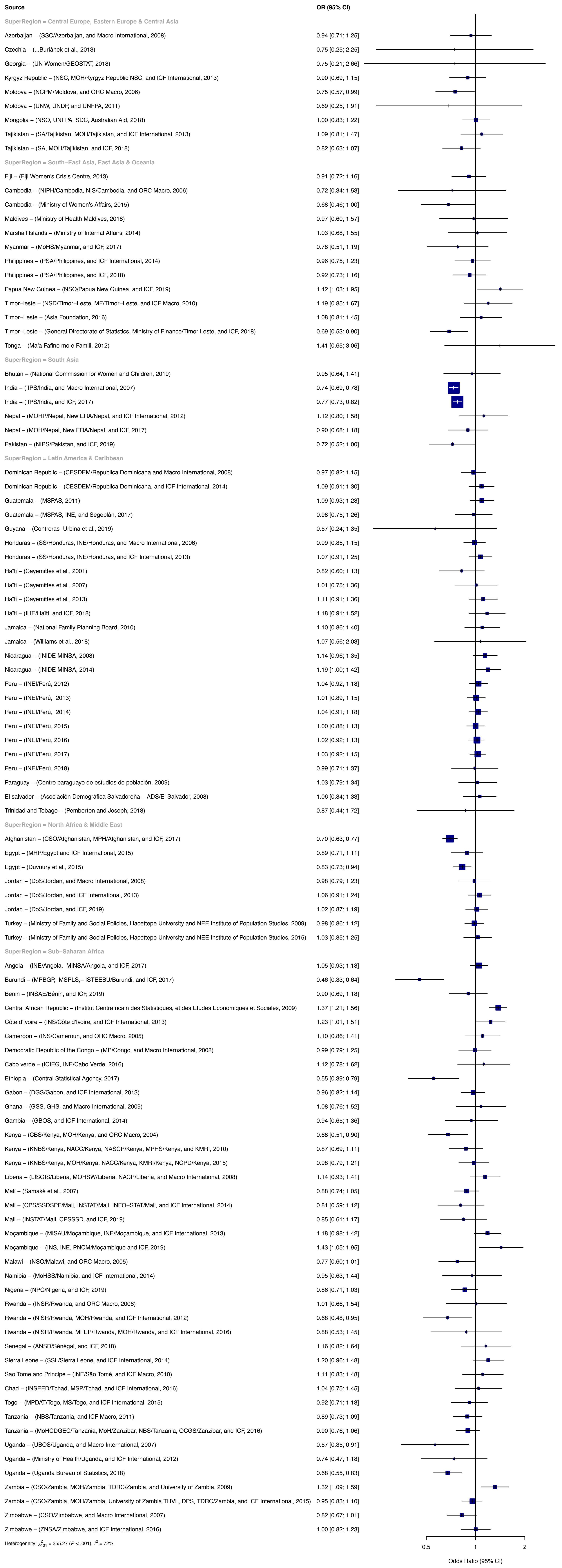
Source
OR (95% CI)
SuperRegion = Central Europe, Eastern Europe & Central Asia
Azerbaijan – (SSC/Azerbaijan, and Macro International, 2008) 0.94 [0.71; 1.25]
Czechia – (...Buriánek et al., 2013) 0.75 [0.25; 2.25]
Georgia – (UN Women/GEOSTAT, 2018) 0.75 [0.21; 2.66]
Kyrgyz Republic – (NSC, MOH/Kyrgyz Republic NSC, and ICF International, 2013) 0.90 [0.69; 1.15]
Moldova – (NCPM/Moldova, and ORC Macro, 2006) 0.75 [0.57; 0.99]
Moldova – (UNW, UNDP, and UNFPA, 2011) 0.69 [0.25; 1.91]
Mongolia – (NSO, UNFPA, SDC, Australian Aid, 2018) 1.00 [0.83; 1.22]
Tajikistan – (SA/Tajikistan, MOH/Tajikistan, and ICF International, 2013) 1.09 [0.81; 1.47]
Tajikistan – (SA, MOH/Tajikistan, and ICF, 2018) 0.82 [0.63; 1.07]
SuperRegion = South–East Asia, East Asia & Oceania
Fiji – (Fiji Women's Crisis Centre, 2013) 0.91 [0.72; 1.16]
Cambodia – (NIPH/Cambodia, NIS/Cambodia, and ORC Macro, 2006) 0.72 [0.34; 1.53]
Cambodia – (Ministry of Women's Affairs, 2015) 0.68 [0.46; 1.00]
Maldives – (Ministry of Health Maldives, 2018) 0.97 [0.60; 1.57]
Marshall Islands – (Ministry of Internal Affairs, 2014) 1.03 [0.68; 1.55]
Myanmar – (MoHS/Myanmar, and ICF, 2017) 0.78 [0.51; 1.19]
Philippines – (PSA/Philippines, and ICF International, 2014) 0.96 [0.75; 1.23]
Philippines – (PSA/Philippines, and ICF, 2018) 0.92 [0.73; 1.16]
Papua New Guinea – (NSO/Papua New Guinea, and ICF, 2019) 1.42 [1.03; 1.95]
Timor–leste – (NSD/Timor–Leste, MF/Timor–Leste, and ICF Macro, 2010) 1.19 [0.85; 1.67]
Timor–Leste – (Asia Foundation, 2016) 1.08 [0.81; 1.45]
Timor–Leste – (General Directorate of Statistics, Ministry of Finance/Timor Leste, and ICF, 2018) 0.69 [0.53; 0.90]
Tonga – (Ma'a Fafine mo e Famili, 2012) 1.41 [0.65; 3.06]
SuperRegion = South Asia
Bhutan – (National Commission for Women and Children, 2019) 0.95 [0.64; 1.41]
India – (IIPS/India, and Macro International, 2007) 0.74 [0.69; 0.78]
India – (IIPS/India, and ICF, 2017) 0.77 [0.73; 0.82]
Nepal – (MOHP/Nepal, New ERA/Nepal, and ICF International, 2012) 1.12 [0.80; 1.58]
Nepal – (MOH/Nepal, New ERA/Nepal, and ICF, 2017) 0.90 [0.68; 1.18]
Pakistan – (NIPS/Pakistan, and ICF, 2019) 0.72 [0.52; 1.00]
SuperRegion = Latin America & Caribbean
Dominican Republic – (CESDEM/Republica Dominicana and Macro International, 2008) 0.97 [0.82; 1.15]
Dominican Republic – (CESDEM/Republica Dominicana, and ICF International, 2014) 1.09 [0.91; 1.30]
Guatemala – (MSPAS, 2011) 1.09 [0.93; 1.28]
Guatemala – (MSPAS, INE, and Segeplán, 2017) 0.98 [0.75; 1.26]
Guyana – (Contreras–Urbina et al., 2019) 0.57 [0.24; 1.35]
Honduras – (SS/Honduras, INE/Honduras, and Macro International, 2006) 0.99 [0.85; 1.15]
Honduras – (SS/Honduras, INE/Honduras, and ICF International, 2013) 1.07 [0.91; 1.25]
Haïti – (Cayemittes et al., 2001) 0.82 [0.60; 1.13]
Haïti – (Cayemittes et al., 2007) 1.01 [0.75; 1.36]
Haïti – (Cayemittes et al., 2013) 1.11 [0.91; 1.36]
Haïti – (IHE/Haïti, and ICF, 2018) 1.18 [0.91; 1.52]
Jamaica – (National Family Planning Board, 2010) 1.10 [0.86; 1.40]
Jamaica – (Williams et al., 2018) 1.07 [0.56; 2.03]
Nicaragua – (INIDE MINSA, 2008) 1.14 [0.96; 1.35]
Nicaragua – (INIDE MINSA, 2014) 1.19 [1.00; 1.42]
Peru – (INEI/Perú, 2012) 1.04 [0.92; 1.18]
Peru – (INEI/Perú, 2013) 1.01 [0.89; 1.15]
Peru – (INEI/Perú, 2014) 1.04 [0.91; 1.18]
Peru – (INEI/Perú, 2015) 1.00 [0.88; 1.13]
Peru – (INEI/Perú, 2016) 1.02 [0.92; 1.13]
Peru – (INEI/Perú, 2017) 1.03 [0.92; 1.15]
Peru – (INEI/Perú, 2018) 0.99 [0.71; 1.37]
Paraguay – (Centro paraguayo de estudios de población, 2009) 1.03 [0.79; 1.34]
El salvador – (Asociación Demográfica Salvadoreña – ADS/El Salvador, 2008) 1.06 [0.84; 1.33]
Trinidad and Tobago – (Pemberton and Joseph, 2018) 0.87 [0.44; 1.72]
SuperRegion = North Africa & Middle East
Afghanistan – (CSO/Afghanistan, MPH/Afghanistan, and ICF, 2017) 0.70 [0.63; 0.77]
Egypt – (MHP/Egypt and ICF International, 2015) 0.89 [0.71; 1.11]
Egypt – (Duvuury et al., 2015) 0.83 [0.73; 0.94]
Jordan – (DoS/Jordan, and Macro International, 2008) 0.98 [0.79; 1.23]
Jordan – (DoS/Jordan, and ICF International, 2013) 1.06 [0.91; 1.24]
Jordan – (DoS/Jordan, and ICF, 2019) 1.02 [0.87; 1.19]
Turkey – (Ministry of Family and Social Policies, Hacettepe University and NEE Institute of Population Studies, 2009) 0.98 [0.86; 1.12]
Turkey – (Ministry of Family and Social Policies, Hacettepe University and NEE Institute of Population Studies, 2015) 1.03 [0.85; 1.25]
SuperRegion = Sub–Saharan Africa
Angola – (INE/Angola, MINSA/Angola, and ICF, 2017) 1.05 [0.93; 1.18]
Burundi – (MPBGP, MSPLS,– ISTEEBU/Burundi, and ICF, 2017) 0.46 [0.33; 0.64]
Benin – (INSAE/Bénin, and ICF, 2019) 0.90 [0.69; 1.18]
Central African Republic – (Institut Centrafricain des Statistiques, et des Etudes Economiques et Sociales, 2009) 1.37 [1.21; 1.56]
Côte d'Ivoire – (INS/Côte d'Ivoire, and ICF International, 2013) 1.23 [1.01; 1.51]
Cameroon – (INS/Cameroun, and ORC Macro, 2005) 1.10 [0.86; 1.41]
Democratic Republic of the Congo – (MP/Congo, and Macro International, 2008) 0.99 [0.79; 1.25]
Cabo verde – (ICIEG, INE/Cabo Verde, 2016) 1.12 [0.78; 1.62]
Ethiopia – (Central Statistical Agency, 2017) 0.55 [0.39; 0.79]
Gabon – (DGS/Gabon, and ICF International, 2013) 0.96 [0.82; 1.14]
Ghana – (GSS, GHS, and Macro International, 2009) 1.08 [0.76; 1.52]
Gambia – (GBOS, and ICF International, 2014) 0.94 [0.65; 1.36]
Kenya – (CBS/Kenya, MOH/Kenya, and ORC Macro, 2004) 0.68 [0.51; 0.90]
Kenya – (KNBS/Kenya, NACC/Kenya, NASCP/Kenya, MPHS/Kenya, and KMRI, 2010) 0.87 [0.69; 1.11]
Kenya – (KNBS/Kenya, MOH/Kenya, NACC/Kenya, KMRI/Kenya, NCPD/Kenya, 2015) 0.98 [0.79; 1.21]
Liberia – (LISGIS/Liberia, MOHSW/Liberia, NACP/Liberia, and Macro International, 2008) 1.14 [0.93; 1.41]
Mali – (Samaké et al., 2007) 0.88 [0.74; 1.05]
Mali – (CPS/SSDSPF/Mali, INSTAT/Mali, INFO–STAT/Mali, and ICF International, 2014) 0.81 [0.59; 1.12]
Mali – (INSTAT/Mali, CPSSSD, and ICF, 2019) 0.85 [0.61; 1.17]
Moçambique – (MISAU/Moçambique, INE/Moçambique, and ICF International, 2013) 1.18 [0.98; 1.42]
Moçambique – (INS, INE, PNCM/Moçambique and ICF, 2019) 1.43 [1.05; 1.95]
Malawi – (NSO/Malawi, and ORC Macro, 2005) 0.77 [0.60; 1.01]
Namibia – (MoHSS/Namibia, and ICF International, 2014) 0.95 [0.63; 1.44]
Nigeria – (NPC/Nigeria, and ICF, 2019) 0.86 [0.71; 1.03]
Rwanda – (INSR/Rwanda, and ORC Macro, 2006) 1.01 [0.66; 1.54]
Rwanda – (NISR/Rwanda, MOH/Rwanda, and ICF International, 2012) 0.68 [0.48; 0.95]
Rwanda – (NISR/Rwanda, MFEP/Rwanda, MOH/Rwanda, and ICF International, 2016) 0.88 [0.53; 1.45]
Senegal – (ANSD/Sénégal, and ICF, 2018) 1.16 [0.82; 1.64]
Sierra Leone – (SSL/Sierra Leone, and ICF International, 2014) 1.20 [0.96; 1.48]
Sao Tome and Principe – (INE/São Tomé, and ICF Macro, 2010) 1.11 [0.83; 1.48]
Chad – (INSEED/Tchad, MSP/Tchad, and ICF International, 2016) 1.04 [0.75; 1.45]
Togo – (MPDAT/Togo, MS/Togo, and ICF International, 2015) 0.92 [0.71; 1.18]
Tanzania – (NBS/Tanzania, and ICF Macro, 2011) 0.89 [0.73; 1.09]
Tanzania – (MoHCDGEC/Tanzania, MoH/Zanzibar, NBS/Tanzania, OCGS/Zanzibar, and ICF, 2016) 0.90 [0.76; 1.06]
Uganda – (UBOS/Uganda, and Macro International, 2007) 0.57 [0.35; 0.91]
Uganda – (Ministry of Health/Uganda, and ICF International, 2012) 0.74 [0.47; 1.18]
Uganda – (Uganda Bureau of Statistics, 2018) 0.68 [0.55; 0.83]
Zambia – (CSO/Zambia, MOH/Zambia, TDRC/Zambia, and University of Zambia, 2009) 1.32 [1.09; 1.59]
Zambia – (CSO/Zambia, MOH/Zambia, University of Zambia THVL, DPS, TDRC/Zambia, and ICF International, 2015) 0.95 [0.83; 1.10]
Zimbabwe – (CSO/Zimbabwe, and Macro International, 2007) 0.82 [0.67; 1.01]
Zimbabwe – (ZNSA/Zimbabwe, and ICF International, 2016) 1.00 [0.82; 1.23]
Heterogeneity: χ^2_{101} = 355.27 (P < .001), I^2 = 72%
0.5
1
2
Odds Ratio (95% CI)

Figure S14. Plot of the data used to estimate, through random-effect logistic regression, the adjustment factor for lifetime intimate partner violence (IPV) in rural regions, as compared to a nationally representative sample.

Source	OR (95% CI)
**SuperRegion = Central Europe, Eastern Europe & Central Asia**	
Albania – (Organisation for Security and Conflict in Europe, 2019)	0.94 [0.75; 1.18]
Azerbaijan – (SSC/Azerbaijan, and Macro International, 2008)	1.05 [0.89; 1.24]
Bosnia and Hezergovina – (Organisation for Security and Conflict in Europe, 2019)	1.00 [0.80; 1.26]
Czechia – (...Buriánek et al., 2013)	1.04 [0.78; 1.40]
Georgia – (UN Women/GEOSTAT, 2018)	0.81 [0.59; 1.10]
Kazakhstan – (Ministry of National Economy of the Republic of Kazakhstan, 2017)	1.01 [0.93; 1.11]
Kyrgyz Republic – (NSC, MOH/Kyrgyz Republic NSC, and ICF International, 2013)	0.97 [0.87; 1.08]
Moldova – (UNW, UNDP, and UNFPA, 2011)	1.30 [0.89; 1.89]
North Macedonia – (Organisation for Security and Conflict in Europe, 2019)	0.68 [0.48; 0.95]
Montenegro – (Organisation for Security and Conflict in Europe, 2019)	1.07 [0.78; 1.47]
Mongolia – (NSO, UNFPA, SDC, Australian Aid, 2018)	0.98 [0.90; 1.07]
Kosovo – (Organisation for Security and Conflict in Europe, 2019)	0.90 [0.69; 1.17]
Tajikistan – (SA/Tajikistan, MOH/Tajikistan, and ICF International, 2013)	0.96 [0.86; 1.08]
Tajikistan – (SA, MOH/Tajikistan, and ICF, 2018)	1.05 [0.96; 1.16]
Ukraine – (UCSR, SSC/Ukraine, MOH/Ukraine, and Macro International, 2008)	1.14 [0.90; 1.44]
Ukraine – (GfK Ukraine, 2014)	1.15 [0.86; 1.55]
**SuperRegion = South–East Asia, East Asia & Oceania**	
Fiji – (Fiji Women's Crisis Centre, 2013)	1.27 [1.12; 1.45]
Cambodia – (NIS/Cambodia, DGH/Cambodia, and ORC Macro, 2001)	1.01 [0.86; 1.19]
Cambodia – (NIPH/Cambodia, NIS/Cambodia, and ORC Macro, 2006)	0.98 [0.81; 1.18]
Cambodia – (NIS/Cambodia, DGH/Cambodia, and ICF International, 2015)	1.06 [0.93; 1.21]
Cambodia – (Ministry of Women's Affairs, 2015)	1.10 [0.97; 1.24]
Maldives – (Ministry of Health Maldives, 2018)	1.06 [0.89; 1.25]
Marshall Islands – (DHS/Marshall Islands, 2008)	1.01 [0.73; 1.41]
Marshall Islands – (Ministry of Internal Affairs, 2014)	1.03 [0.79; 1.34]
Myanmar – (MoHS/Myanmar, and ICF, 2017)	1.05 [0.91; 1.22]
Philippines – (NSO/Philippines, and ICF Macro, 2009)	1.04 [0.93; 1.16]
Philippines – (PSA/Philippines, and ICF International, 2014)	0.98 [0.87; 1.09]
Philippines – (PSA/Philippines, and ICF, 2018)	1.01 [0.92; 1.11]
Papua New Guinea – (NSO/Papua New Guinea, and ICF, 2019)	0.96 [0.87; 1.06]
Timor–leste – (NSD/Timor–Leste, MF/Timor–Leste, and ICF Macro, 2010)	0.85 [0.74; 0.99]
Timor–Leste – (Asia Foundation, 2016)	0.95 [0.80; 1.12]
Timor–Leste – (General Directorate of Statistics, Ministry of Finance/Timor Leste, and ICF, 2018)	1.15 [1.04; 1.28]
Tonga – (Ma'a Fafine mo e Famili, 2012)	0.97 [0.73; 1.30]
**SuperRegion = South Asia**	
Bangladesh – (NIPORT/Bangladesh, Mitra, Associates/Bangladesh, and Macro International, 2009)	1.07 [0.98; 1.17]
Bhutan – (National Commission for Women and Children, 2019)	1.00 [0.82; 1.22]
India – (IIPS/India, and Macro International, 2007)	1.13 [1.11; 1.16]
India – (IIPS/India, and ICF, 2017)	1.15 [1.12; 1.18]
Nepal – (MOHP/Nepal, New ERA/Nepal, and ICF International, 2012)	1.05 [0.93; 1.17]
Nepal – (MOH/Nepal, New ERA/Nepal, and ICF, 2017)	1.07 [0.93; 1.23]
Pakistan – (NIPS/Pakistan, and ICF, 2019)	1.11 [0.97; 1.26]
**SuperRegion = Latin America & Caribbean**	
Colombia – (Profamilia/Colombia, 2000)	0.82 [0.74; 0.91]
Colombia – (Profamilia/Colombia, 2005)	0.93 [0.88; 0.99]
Colombia – (Profamilia/Colombia, 2005)	0.85 [0.81; 0.90]
Dominican Republic – (Achécar et al., 2003)	0.86 [0.76; 0.98]
Dominican Republic – (CESDEM/Republica Dominicana and Macro International, 2008)	1.06 [0.93; 1.20]
Dominican Republic – (CESDEM/Republica Dominicana, and ICF International, 2014)	0.90 [0.77; 1.05]
Guatemala – (MSPAS, 2011)	0.91 [0.85; 0.97]
Guatemala – (MSPAS, INE, and Segeplán, 2017)	0.88 [0.79; 0.99]
Guyana – (Contreras–Urbina et al., 2019)	1.00 [0.84; 1.19]
Honduras – (SS/Honduras, INE/Honduras, and ICF International, 2013)	0.87 [0.80; 0.94]
Haïti – (Cayemittes et al., 2001)	0.98 [0.84; 1.14]
Haïti – (Cayemittes et al., 2007)	1.04 [0.92; 1.19]
Haïti – (Cayemittes et al., 2013)	0.89 [0.80; 1.00]
Haïti – (IHE/Haïti, and ICF, 2018)	0.84 [0.74; 0.96]
Jamaica – (National Family Planning Board, 2010)	0.97 [0.88; 1.07]
Jamaica – (Williams et al., 2018)	0.98 [0.76; 1.27]
Nicaragua – (INIDE MINSA, 2008)	0.81 [0.75; 0.87]
Nicaragua – (INIDE MINSA, 2014)	0.71 [0.66; 0.77]
Panama – (Instituto Conmemorativo Gorgas de Estudios de la Salud, 2011)	0.82 [0.67; 1.00]
Panama – (Instituto Conmemorativo Gorgas de Estudios de la Salud, 2018)	1.37 [1.15; 1.64]
Peru – (INEI/Perú, 2012)	0.91 [0.85; 0.99]
Peru – (INEI/Perú, 2013)	0.92 [0.85; 0.99]
Peru – (INEI/Perú, 2014)	0.92 [0.85; 1.00]
Peru – (INEI/Perú, 2015)	0.94 [0.87; 1.02]
Peru – (INEI/Perú, 2016)	0.92 [0.86; 0.98]
Peru – (INEI/Perú, 2017)	0.88 [0.82; 0.94]
Paraguay – (Centro paraguayo de estudios de población, 2009)	0.75 [0.65; 0.86]
El salvador – (Asociación Demográfica Salvadoreña – ADS/El Salvador, 2008)	0.80 [0.73; 0.88]
Trinidad and Tobago – (Pemberton and Joseph, 2018)	1.01 [0.76; 1.33]
**SuperRegion = North Africa & Middle East**	
Afghanistan – (CSO/Afghanistan, MPH/Afghanistan, and ICF, 2017)	1.12 [1.07; 1.17]
Egypt – (MHP/Egypt and ICF International, 2015)	1.05 [0.97; 1.15]
Egypt – (Duvuury et al., 2015)	1.24 [1.17; 1.30]
Jordan – (DoS/Jordan, and Macro International, 2008)	0.98 [0.79; 1.23]
Jordan – (DoS/Jordan, and ICF International, 2013)	0.82 [0.71; 0.95]
Jordan – (DoS/Jordan, and ICF, 2019)	0.81 [0.66; 1.01]
Turkey – (Ministry of Family and Social Policies, Hacettepe University and NEE Institute of Population Studies, 2009)	1.21 [1.11; 1.32]
Turkey – (Ministry of Family and Social Policies, Hacettepe University and NEE Institute of Population Studies, 2015)	1.07 [0.96; 1.18]
**SuperRegion = Sub–Saharan Africa**	
Angola – (INE/Angola, MINSA/Angola, and ICF, 2017)	0.90 [0.83; 0.98]
Burundi – (MPBGP, MSPLS,– ISTEEBU/Burundi, and ICF, 2017)	1.07 [1.00; 1.15]
Benin – (INSAE/Bénin, and ICF, 2019)	1.07 [0.96; 1.21]
Burkina faso – (Institut National de la Statistique et de la Démographie, 2012)	0.90 [0.82; 1.00]
Central African Republic – (Institut Centrafricain des Statistiques, et des Etudes Economiques et Sociales, 2009)	0.77 [0.72; 0.83]
Côte d'Ivoire – (INS/Côte d'Ivoire, and ICF International, 2013)	0.88 [0.78; 0.99]
Cameroon – (INS/Cameroun, and ORC Macro, 2005)	0.90 [0.78; 1.04]
Cameroon – (INS/Cameroun, and ICF International, 2012)	1.05 [0.94; 1.17]
Cabo verde – (Instituto Nacional de Estatística, 2008)	0.78 [0.54; 1.12]
Cabo verde – (ICIEG, INE/Cabo Verde, 2016)	0.71 [0.49; 1.02]
Ethiopia – (Central Statistical Agency, 2017)	1.08 [0.98; 1.19]
Gabon – (DGS/Gabon, and ICF International, 2013)	1.21 [1.00; 1.47]
Ghana – (GSS, GHS, and Macro International, 2009)	0.96 [0.79; 1.16]
Gambia – (GBOS, and ICF International, 2014)	1.17 [1.01; 1.35]
Equatorial Guinea – (PIP/Guinea Ecuatorial, and ICF International, 2012)	1.08 [0.85; 1.38]
Kenya – (CBS/Kenya, MOH/Kenya, and ORC Macro, 2004)	1.09 [0.99; 1.20]
Kenya – (KNBS/Kenya, NACC/Kenya, NASCP/Kenya, MPHS/Kenya, and KMRI, 2010)	1.08 [0.99; 1.19]
Kenya – (KNBS/Kenya, MOH/Kenya, NACC/Kenya, KMRI/Kenya, NCPD/Kenya, 2015)	1.08 [0.98; 1.20]
Liberia – (LISGIS/Liberia, MOHSW/Liberia, NACP/Liberia, and Macro International, 2008)	0.92 [0.83; 1.03]
Mali – (Samaké et al., 2007)	1.06 [0.98; 1.15]
Mali – (CPS/SSDSPF/Mali, INSTAT/Mali, INFO–STAT/Mali, and ICF International, 2014)	1.05 [0.94; 1.17]
Mali – (INSTAT/Mali, CPSSSD, and ICF, 2019)	0.99 [0.89; 1.10]
Moçambique – (MISAU/Moçambique, INE/Moçambique, and ICF International, 2013)	0.91 [0.83; 0.99]
Moçambique – (INS, INE, PNCM/Moçambique and ICF, 2019)	0.83 [0.71; 0.97]
Malawi – (NSO/Malawi, and ORC Macro, 2005)	1.01 [0.93; 1.08]
Malawi – (NSO/Malawi, and ICF Macro, 2011)	0.94 [0.86; 1.02]
Malawi – (NSO/Malawi, and ICF, 2017)	1.01 [0.93; 1.10]
Namibia – (MoHSS/Namibia, and ICF International, 2014)	1.03 [0.79; 1.35]
Nigeria – (NPC/Nigeria, and ICF Macro, 2009)	1.02 [0.96; 1.08]
Nigeria – (NPC/Nigeria, and ICF International, 2014)	0.93 [0.87; 0.98]
Nigeria – (NPC/Nigeria, and ICF, 2019)	1.04 [0.96; 1.14]
Rwanda – (INSR/Rwanda, and ORC Macro, 2006)	1.01 [0.89; 1.15]
Rwanda – (NISR/Rwanda, MFEP/Rwanda, MOH/Rwanda, and ICF International, 2016)	1.05 [0.90; 1.22]
Senegal – (ANSD/Sénégal, and ICF, 2018)	0.97 [0.82; 1.14]
Sierra Leone – (SSL/Sierra Leone, and ICF International, 2014)	0.97 [0.89; 1.07]
Sao Tome and Principe – (INE/São Tomé, and ICF Macro, 2010)	0.89 [0.73; 1.08]
Chad – (INSEED/Tchad, MSP/Tchad, and ICF International, 2016)	1.01 [0.90; 1.12]
Togo – (MPDAT/Togo, MS/Togo, and ICF International, 2015)	1.09 [0.98; 1.21]
Tanzania – (NBS/Tanzania, and ICF Macro, 2011)	1.02 [0.94; 1.11]
Tanzania – (MoHCDGEC/Tanzania, MoH/Zanzibar, NBS/Tanzania, OCGS/Zanzibar, and ICF, 2016)	1.07 [1.00; 1.15]
Uganda – (UBOS/Uganda, and Macro International, 2007)	1.11 [0.95; 1.28]
Uganda – (Ministry of Health/Uganda, and ICF International, 2012)	1.05 [0.91; 1.22]
Uganda – (Uganda Bureau of Statistics, 2018)	1.12 [1.04; 1.20]
South Africa – (NDH/South Africa, and ICF, 2019)	1.04 [0.92; 1.18]
Zambia – (CSO/Zambia, MOH/Zambia, TDRC/Zambia, and University of Zambia, 2009)	0.87 [0.79; 0.96]
Zambia – (CSO/Zambia, MOH/Zambia, University of Zambia THVL, DPS, TDRC/Zambia, and ICF International, 2015)	1.00 [0.94; 1.08]
Zimbabwe – (CSO/Zimbabwe, and Macro International, 2007)	1.13 [1.03; 1.24]
Zimbabwe – (ZIMSTAT/Zimbabwe, and ICF International, 2012)	1.04 [0.95; 1.14]
Zimbabwe – (ZNSA/Zimbabwe, and ICF International, 2016)	1.02 [0.94; 1.12]

Heterogeneity: $\chi^2_{125} = 826.19$ ($P < .001$), $I^2 = 85\%$

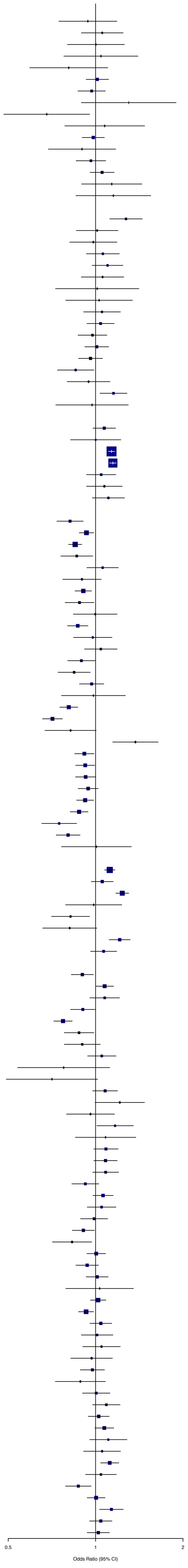

Figure S15. Plot of the data used to estimate, through random-effect logistic regression, the adjustment factor for past year intimate partner violence (IPV) in rural regions, as compared to a nationally representative sample.

Source	OR (95% CI)
**SuperRegion = Central Europe, Eastern Europe & Central Asia**	
Azerbaijan – (SSC/Azerbaijan, and Macro International, 2008)	1.08 [0.80; 1.46]
Czechia – (...Buriánek et al., 2013)	1.29 [0.34; 4.83]
Georgia – (UN Women/GEOSTAT, 2018)	1.51 [0.56; 4.04]
Kyrgyz Republic – (NSC, MOH/Kyrgyz Republic NSC, and ICF International, 2013)	1.07 [0.88; 1.30]
Moldova – (NCPM/Moldova, and ORC Macro, 2006)	1.18 [0.95; 1.46]
Moldova – (UNW, UNDP, and UNFPA, 2011)	1.27 [0.51; 3.17]
Mongolia – (NSO, UNFPA, SDC, Australian Aid, 2018)	0.99 [0.82; 1.20]
Tajikistan – (SA/Tajikistan, MOH/Tajikistan, and ICF International, 2013)	0.97 [0.79; 1.19]
Tajikistan – (SA, MOH/Tajikistan, and ICF, 2018)	1.07 [0.90; 1.26]
**SuperRegion = South−East Asia, East Asia & Oceania**	
Fiji – (Fiji Women's Crisis Centre, 2013)	1.09 [0.87; 1.35]
Cambodia – (NIPH/Cambodia, NIS/Cambodia, and ORC Macro, 2006)	1.07 [0.75; 1.52]
Cambodia – (Ministry of Women's Affairs, 2015)	1.10 [0.85; 1.42]
Maldives – (Ministry of Health Maldives, 2018)	1.02 [0.69; 1.50]
Marshall Islands – (Ministry of Internal Affairs, 2014)	1.00 [0.58; 1.72]
Myanmar – (MoHS/Myanmar, and ICF, 2017)	1.08 [0.83; 1.42]
Philippines – (PSA/Philippines, and ICF International, 2014)	1.03 [0.81; 1.32]
Philippines – (PSA/Philippines, and ICF, 2018)	1.06 [0.86; 1.31]
Papua New Guinea – (NSO/Papua New Guinea, and ICF, 2019)	0.96 [0.83; 1.11]
Timor−leste – (NSD/Timor−Leste, MF/Timor−Leste, and ICF Macro, 2010)	0.95 [0.75; 1.19]
Timor−Leste – (Asia Foundation, 2016)	0.97 [0.77; 1.23]
Timor−Leste – (General Directorate of Statistics, Ministry of Finance/Timor Leste, and ICF, 2018)	1.13 [0.95; 1.35]
Tonga – (Ma'a Fafine mo e Famili, 2012)	0.88 [0.49; 1.57]
**SuperRegion = South Asia**	
Bhutan – (National Commission for Women and Children, 2019)	1.05 [0.70; 1.58]
India – (IIPS/India, and Macro International, 2007)	1.13 [1.08; 1.18]
India – (IIPS/India, and ICF, 2017)	1.13 [1.08; 1.18]
Nepal – (MOHP/Nepal, New ERA/Nepal, and ICF International, 2012)	0.97 [0.76; 1.23]
Nepal – (MOH/Nepal, New ERA/Nepal, and ICF, 2017)	1.16 [0.86; 1.55]
Pakistan – (NIPS/Pakistan, and ICF, 2019)	1.17 [0.92; 1.48]
**SuperRegion = Latin America & Caribbean**	
Dominican Republic – (CESDEM/Republica Dominicana and Macro International, 2008)	1.06 [0.84; 1.33]
Dominican Republic – (CESDEM/Republica Dominicana, and ICF International, 2014)	0.88 [0.67; 1.15]
Guatemala – (MSPAS, 2011)	0.92 [0.79; 1.08]
Guatemala – (MSPAS, INE, and Segeplán, 2017)	1.03 [0.82; 1.29]
Guyana – (Contreras−Urbina et al., 2019)	1.11 [0.73; 1.68]
Honduras – (SS/Honduras, INE/Honduras, and Macro International, 2006)	1.00 [0.85; 1.17]
Honduras – (SS/Honduras, INE/Honduras, and ICF International, 2013)	0.95 [0.80; 1.12]
Haïti – (Cayemittes et al., 2001)	1.13 [0.88; 1.46]
Haïti – (Cayemittes et al., 2007)	0.99 [0.75; 1.32]
Haïti – (Cayemittes et al., 2013)	0.92 [0.75; 1.11]
Haïti – (IHE/Haïti, and ICF, 2018)	0.87 [0.68; 1.11]
Jamaica – (National Family Planning Board, 2010)	0.85 [0.67; 1.07]
Jamaica – (Williams et al., 2018)	0.91 [0.44; 1.87]
Nicaragua – (INIDE MINSA, 2008)	0.83 [0.69; 0.99]
Nicaragua – (INIDE MINSA, 2014)	0.75 [0.61; 0.91]
Peru – (INEI/Perú, 2012)	0.90 [0.76; 1.07]
Peru – (INEI/Perú, 2013)	0.96 [0.81; 1.14]
Peru – (INEI/Perú, 2014)	0.91 [0.75; 1.10]
Peru – (INEI/Perú, 2015)	1.01 [0.85; 1.20]
Peru – (INEI/Perú, 2016)	0.92 [0.79; 1.08]
Peru – (INEI/Perú, 2017)	0.92 [0.77; 1.09]
Peru – (INEI/Perú, 2018)	1.02 [0.73; 1.40]
Paraguay – (Centro paraguayo de estudios de población, 2009)	0.94 [0.71; 1.26]
El salvador – (Asociación Demográfica Salvadoreña – ADS/El Salvador, 2008)	0.94 [0.74; 1.19]
Trinidad and Tobago – (Pemberton and Joseph, 2018)	1.46 [0.68; 3.17]
**SuperRegion = North Africa & Middle East**	
Afghanistan – (CSO/Afghanistan, MPH/Afghanistan, and ICF, 2017)	1.10 [1.03; 1.17]
Egypt – (MHP/Egypt and ICF International, 2015)	1.07 [0.90; 1.27]
Egypt – (Duvuury et al., 2015)	1.14 [1.03; 1.27]
Jordan – (DoS/Jordan, and Macro International, 2008)	1.10 [0.73; 1.65]
Jordan – (DoS/Jordan, and ICF International, 2013)	0.75 [0.55; 1.01]
Jordan – (DoS/Jordan, and ICF, 2019)	0.84 [0.57; 1.23]
Turkey – (Ministry of Family and Social Policies, Hacettepe University and NEE Institute of Population Studies, 2009)	1.03 [0.86; 1.25]
Turkey – (Ministry of Family and Social Policies, Hacettepe University and NEE Institute of Population Studies, 2015)	0.91 [0.70; 1.18]
**SuperRegion = Sub−Saharan Africa**	
Angola – (INE/Angola, MINSA/Angola, and ICF, 2017)	0.92 [0.79; 1.06]
Burundi – (MPBGP, MSPLS,– ISTEEBU/Burundi, and ICF, 2017)	1.09 [0.96; 1.23]
Benin – (INSAE/Bénin, and ICF, 2019)	1.07 [0.85; 1.33]
Central African Republic – (Institut Centrafricain des Statistiques, et des Etudes Economiques et Sociales, 2009)	0.78 [0.69; 0.88]
Côte d'Ivoire – (INS/Côte d'Ivoire, and ICF International, 2013)	0.85 [0.70; 1.03]
Cameroon – (INS/Cameroun, and ORC Macro, 2005)	0.90 [0.70; 1.17]
Democratic Republic of the Congo – (MP/Congo, and Macro International, 2008)	1.01 [0.82; 1.23]
Cabo verde – (ICIEG, INE/Cabo Verde, 2016)	0.68 [0.37; 1.24]
Ethiopia – (Central Statistical Agency, 2017)	1.11 [0.94; 1.32]
Gabon – (DGS/Gabon, and ICF International, 2013)	1.24 [0.90; 1.71]
Ghana – (GSS, GHS, and Macro International, 2009)	0.96 [0.69; 1.32]
Gambia – (GBOS, and ICF International, 2014)	1.06 [0.74; 1.51]
Kenya – (CBS/Kenya, MOH/Kenya, and ORC Macro, 2004)	1.11 [0.94; 1.30]
Kenya – (KNBS/Kenya, NACC/Kenya, NASCP/Kenya, MPHS/Kenya, and KMRI, 2010)	1.04 [0.89; 1.21]
Kenya – (KNBS/Kenya, MOH/Kenya, NACC/Kenya, KMRI/Kenya, NCPD/Kenya, 2015)	1.02 [0.85; 1.22]
Liberia – (LISGIS/Liberia, MOHSW/Liberia, NACP/Liberia, and Macro International, 2008)	0.93 [0.78; 1.11]
Mali – (Samaké et al., 2007)	1.06 [0.93; 1.20]
Mali – (CPS/SSDSPF/Mali, INSTAT/Mali, INFO−STAT/Mali, and ICF International, 2014)	1.06 [0.87; 1.28]
Mali – (INSTAT/Mali, CPSSSD, and ICF, 2019)	1.05 [0.86; 1.28]
Moçambique – (MISAU/Moçambique, INE/Moçambique, and ICF International, 2013)	0.92 [0.80; 1.07]
Moçambique – (INS, INE, PNCM/Moçambique and ICF, 2019)	0.83 [0.63; 1.08]
Malawi – (NSO/Malawi, and ORC Macro, 2005)	1.04 [0.91; 1.18]
Namibia – (MoHSS/Namibia, and ICF International, 2014)	1.07 [0.68; 1.68]
Nigeria – (NPC/Nigeria, and ICF, 2019)	1.10 [0.94; 1.29]
Rwanda – (INSR/Rwanda, and ORC Macro, 2006)	1.00 [0.81; 1.24]
Rwanda – (NISR/Rwanda, MOH/Rwanda, and ICF International, 2012)	1.06 [0.90; 1.25]
Rwanda – (NISR/Rwanda, MFEP/Rwanda, MOH/Rwanda, and ICF International, 2016)	1.03 [0.78; 1.35]
Senegal – (ANSD/Sénégal, and ICF, 2018)	0.90 [0.65; 1.24]
Sierra Leone – (SSL/Sierra Leone, and ICF International, 2014)	0.92 [0.78; 1.09]
Sao Tome and Principe – (INE/São Tomé, and ICF Macro, 2010)	0.87 [0.63; 1.20]
Chad – (INSEED/Tchad, MSP/Tchad, and ICF International, 2016)	0.98 [0.80; 1.21]
Togo – (MPDAT/Togo, MS/Togo, and ICF International, 2015)	1.07 [0.86; 1.32]
Tanzania – (NBS/Tanzania, and ICF Macro, 2011)	1.04 [0.91; 1.19]
Tanzania – (MoHCDGEC/Tanzania, MoH/Zanzibar, NBS/Tanzania, OCGS/Zanzibar, and ICF, 2016)	1.05 [0.93; 1.20]
Uganda – (UBOS/Uganda, and Macro International, 2007)	1.09 [0.87; 1.37]
Uganda – (Ministry of Health/Uganda, and ICF International, 2012)	1.16 [0.91; 1.49]
Uganda – (Uganda Bureau of Statistics, 2018)	1.11 [0.98; 1.26]
Zambia – (CSO/Zambia, MOH/Zambia, TDRC/Zambia, and University of Zambia, 2009)	0.85 [0.72; 1.00]
Zambia – (CSO/Zambia, MOH/Zambia, University of Zambia THVL, DPS, TDRC/Zambia, and ICF International, 2015)	1.03 [0.91; 1.17]
Zimbabwe – (CSO/Zimbabwe, and Macro International, 2007)	1.11 [0.94; 1.30]
Zimbabwe – (ZNSA/Zimbabwe, and ICF International, 2016)	1.01 [0.85; 1.19]

Heterogeneity: $\chi^2_{101} = 145.79$ ($P = .002$), $I^2 = 31\%$

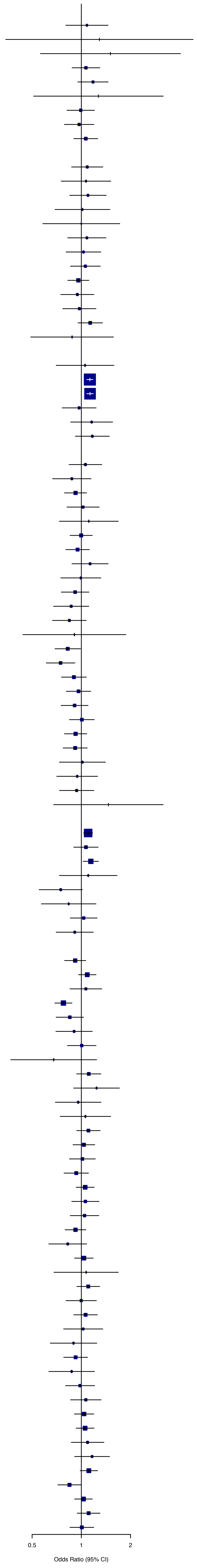

Figure S16. Posterior predictive checks for the lifetime intimate partner violence (IPV) model.

Ever IPV – Asia Pacific, High Income

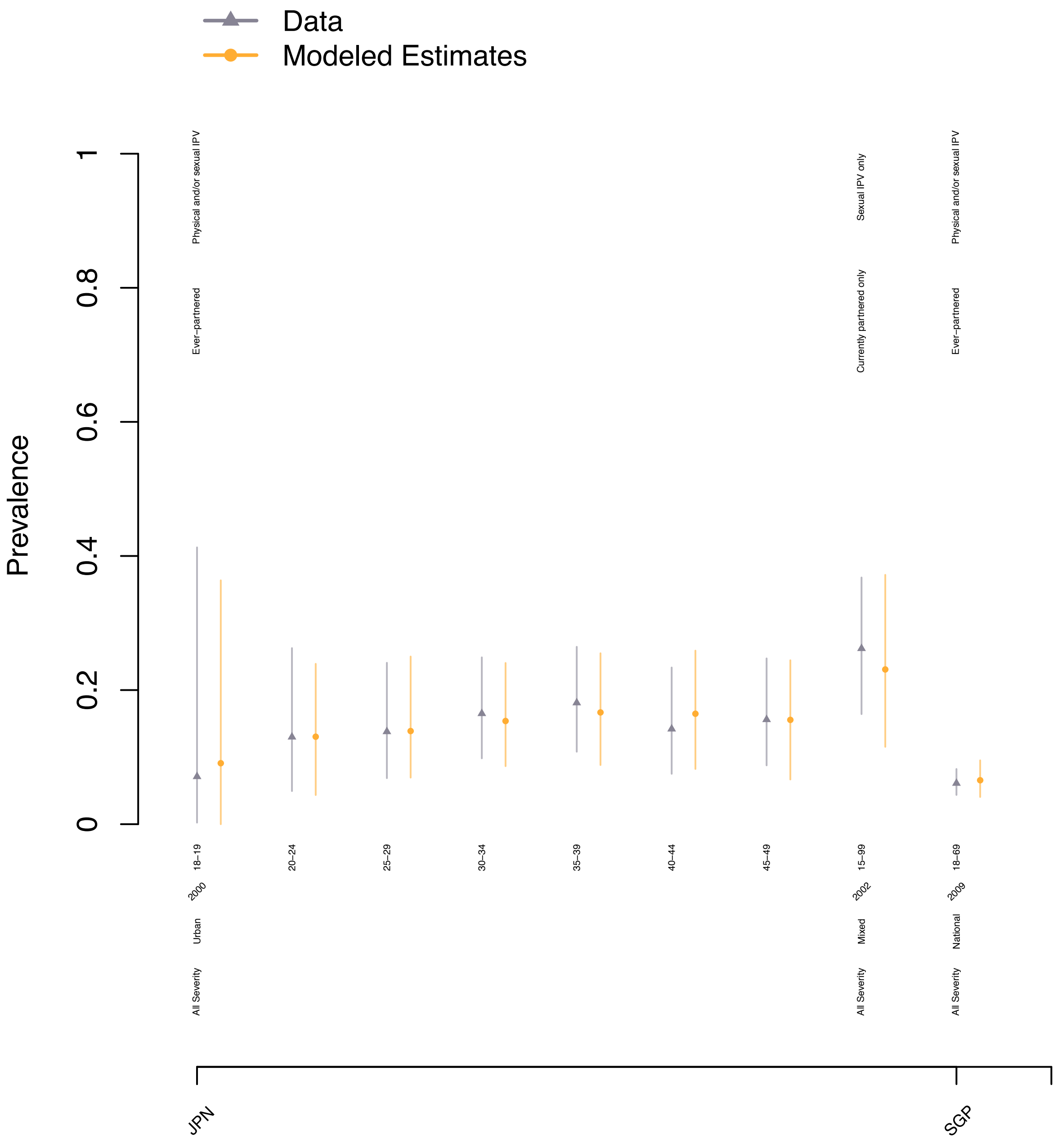

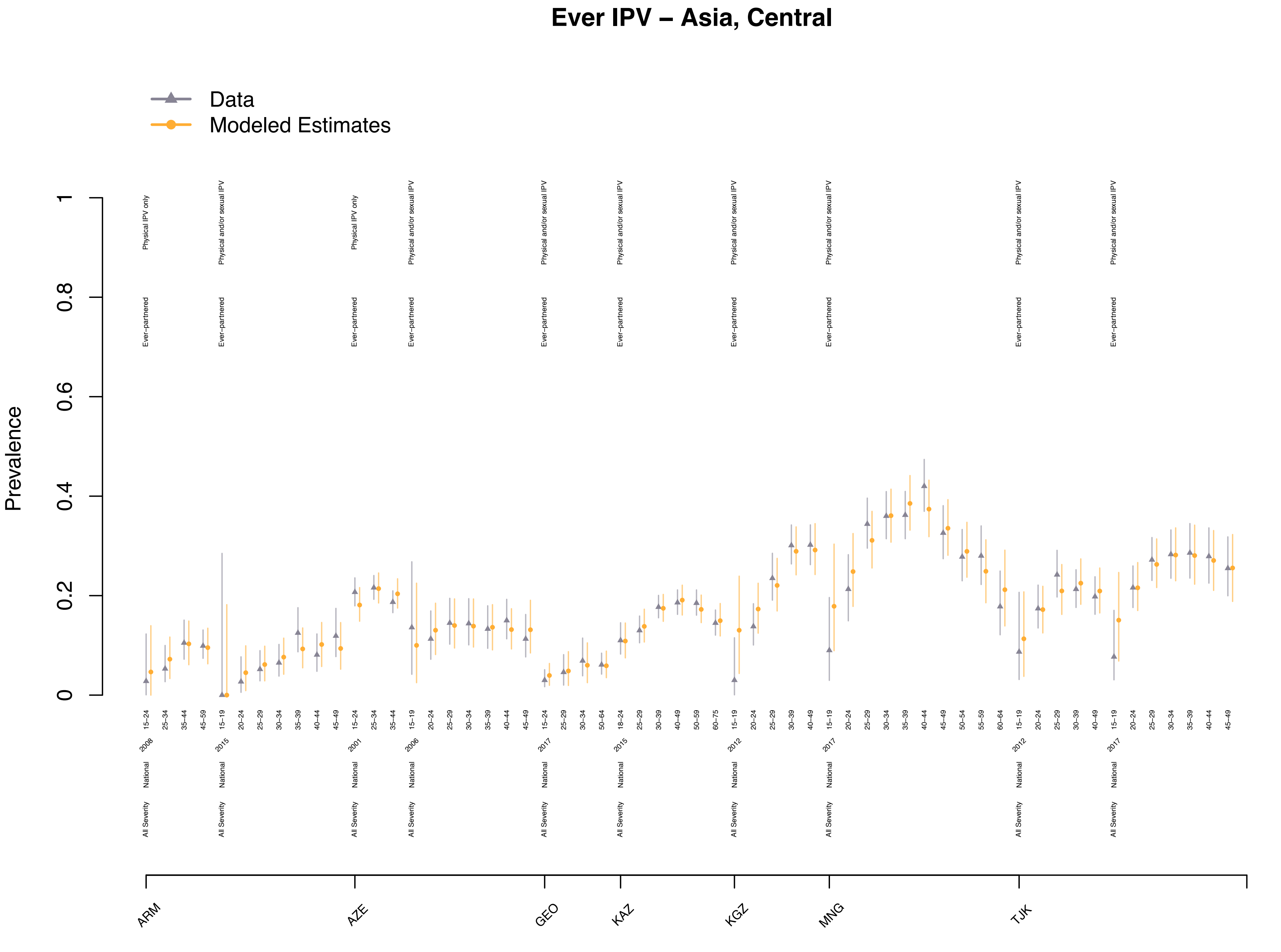
Ever IPV – Asia, Central
Data
Modeled Estimates
Prevalence
0
0.2
0.4
0.6
0.8
1
Physical IPV only
Physical and/or sexual IPV
Ever−partnered
National
All Severity
2008
2015
2001
2006
2017
2012
ARM
AZE
GEO
KAZ
KGZ
MNG
TJK

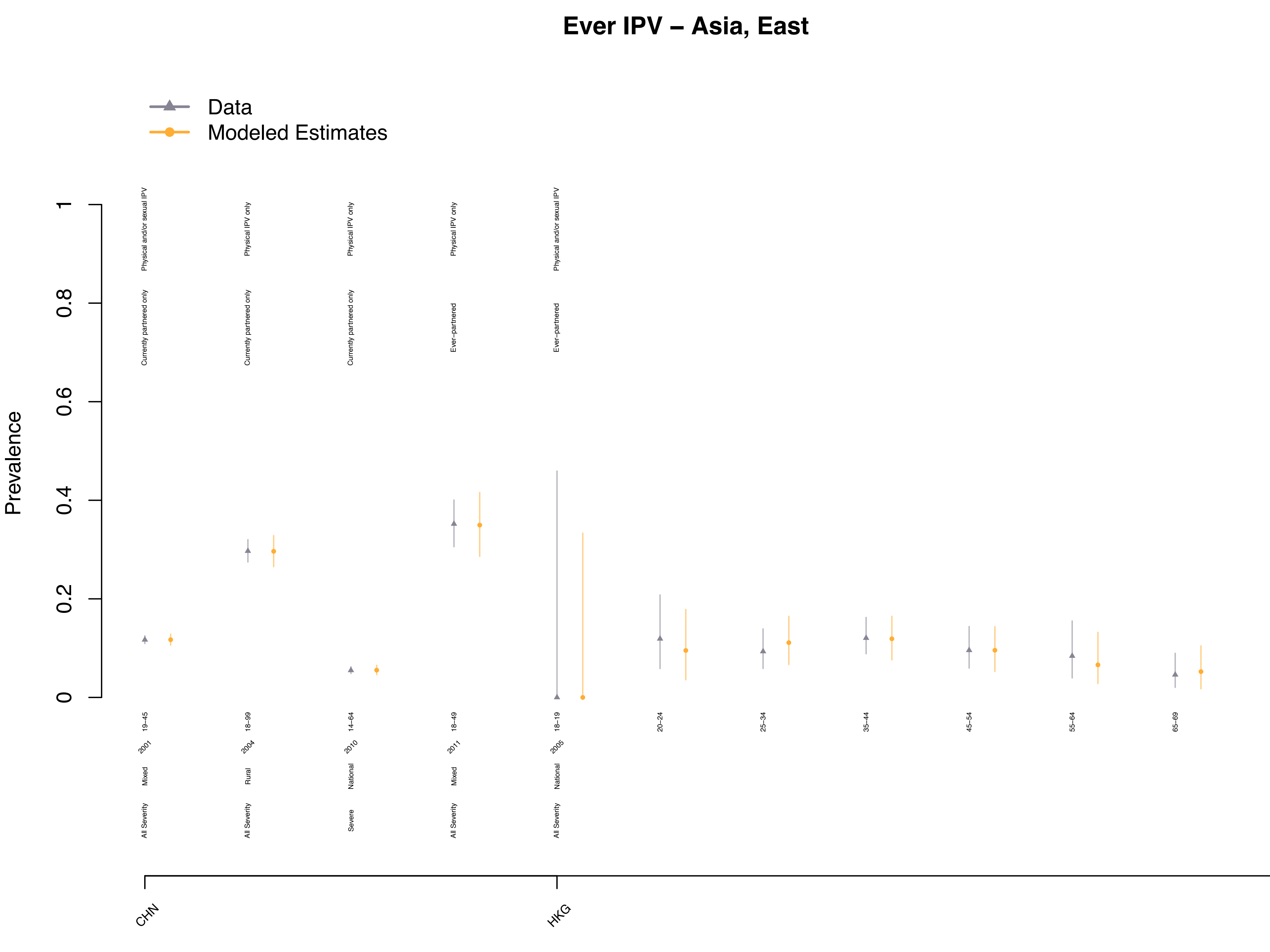

Ever IPV – Asia, East
Data
Modeled Estimates
Prevalence
0
0.2
0.4
0.6
0.8
1
Physical and/or sexual IPV
Currently partnered only
Physical IPV only
Currently partnered only
Physical IPV only
Currently partnered only
Physical IPV only
Ever−partnered
Physical and/or sexual IPV
Ever−partnered
19−45
2001
Mixed
All Severity
18−99
2004
Rural
All Severity
14−64
2010
National
Severe
18−49
2011
Mixed
All Severity
18−19
2005
National
All Severity
20−24
25−34
35−44
45−54
55−64
65−69
CHN
HKG

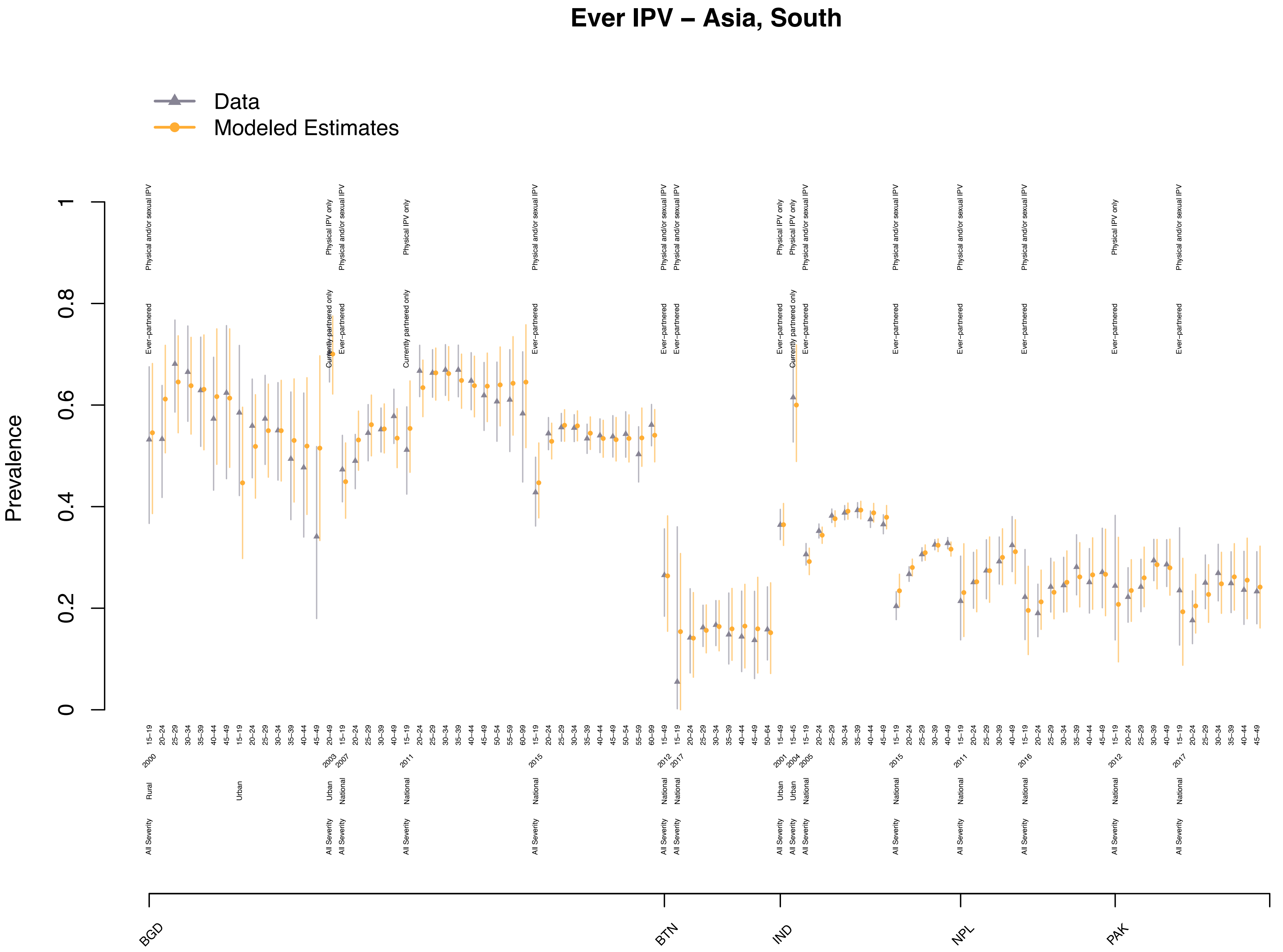

Ever IPV – Asia, South
Data
Modeled Estimates
Prevalence
BGD
BTN
IND
NPL
PAK

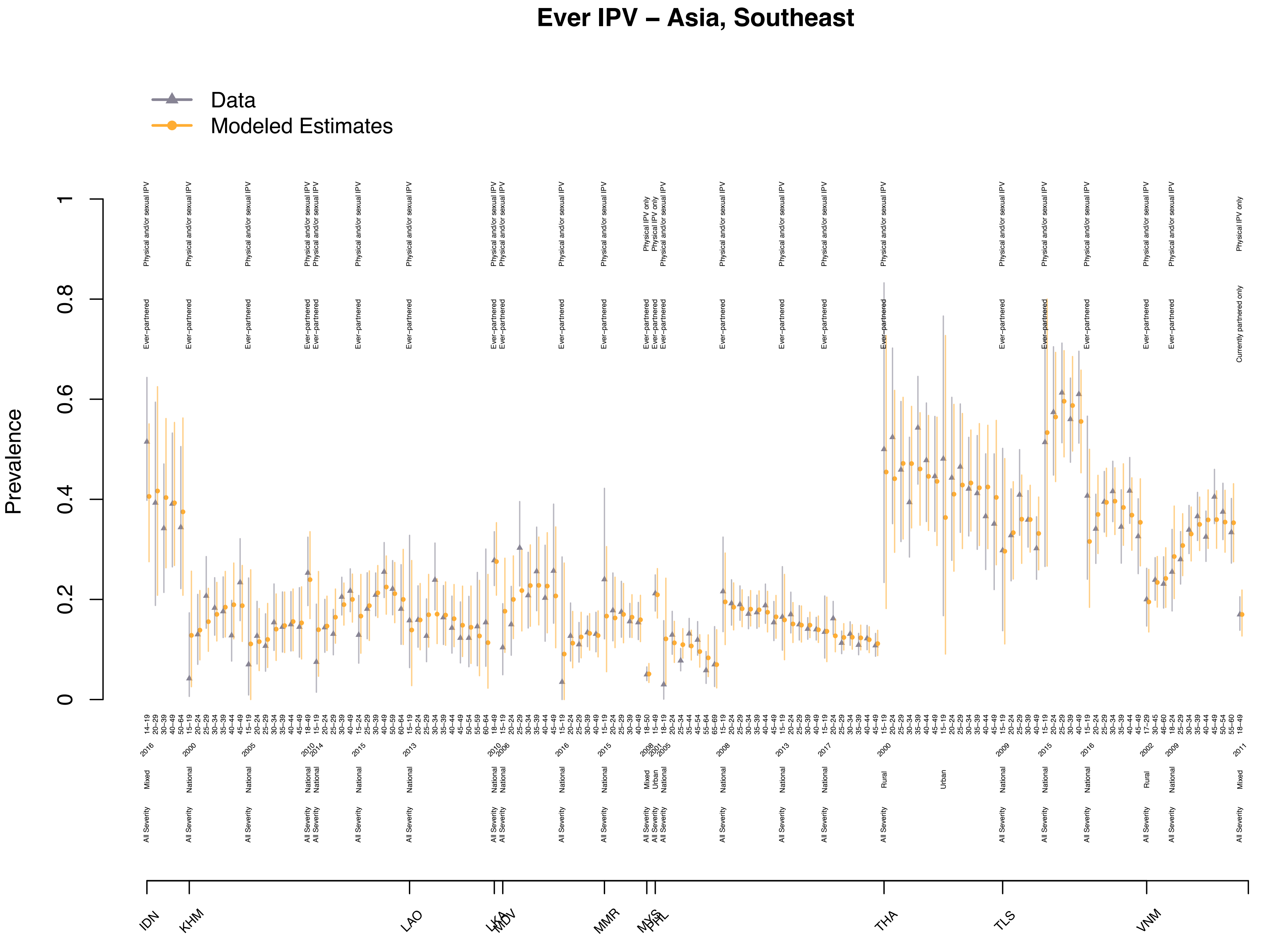

Ever IPV – Asia, Southeast
Data
Modeled Estimates
Prevalence
0
0.2
0.4
0.6
0.8
1
IDN
KHM
LAO
LKA
MDV
MMR
MYS
PHL
THA
TLS
VNM

Ever IPV – Australasia

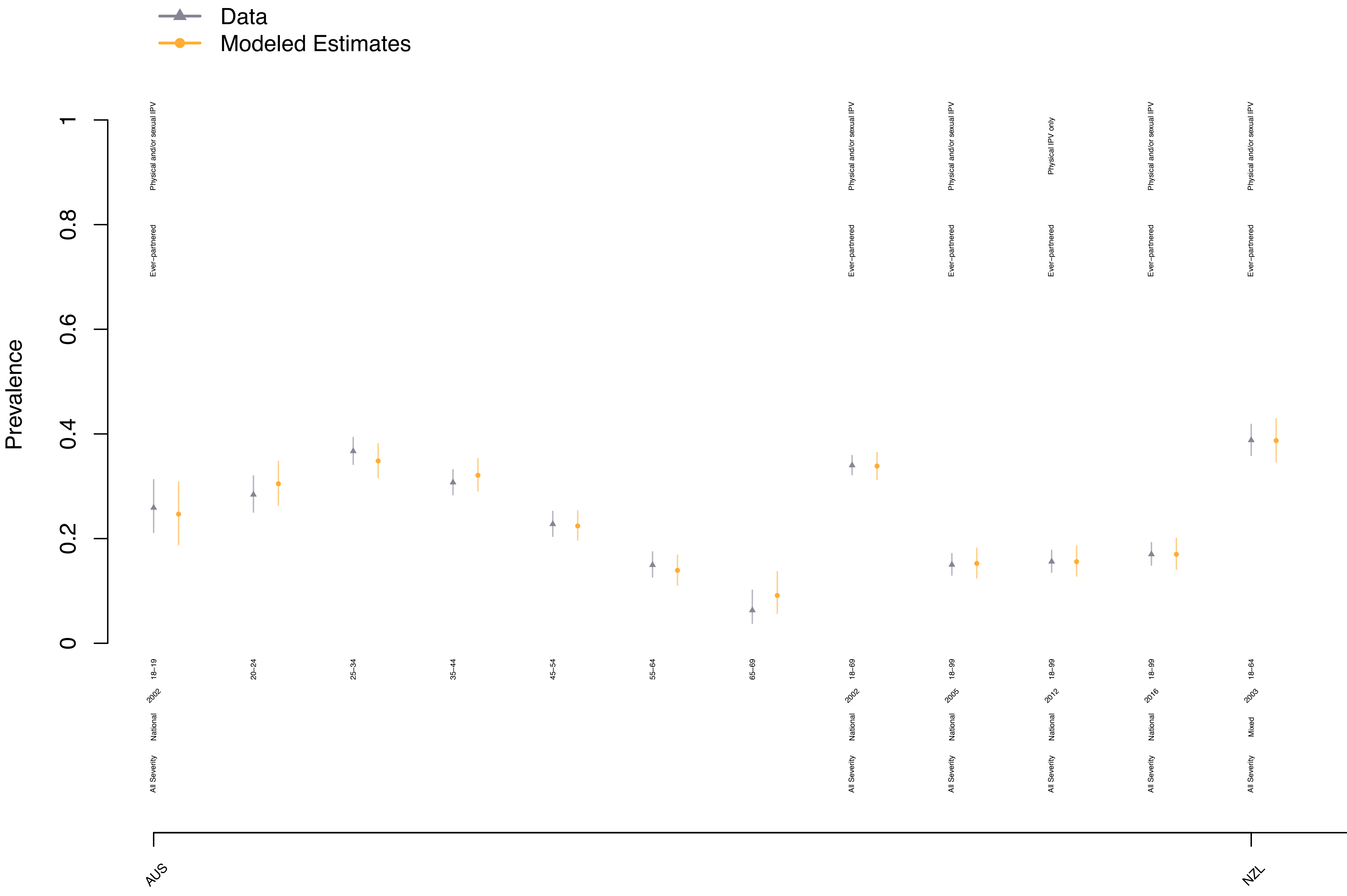

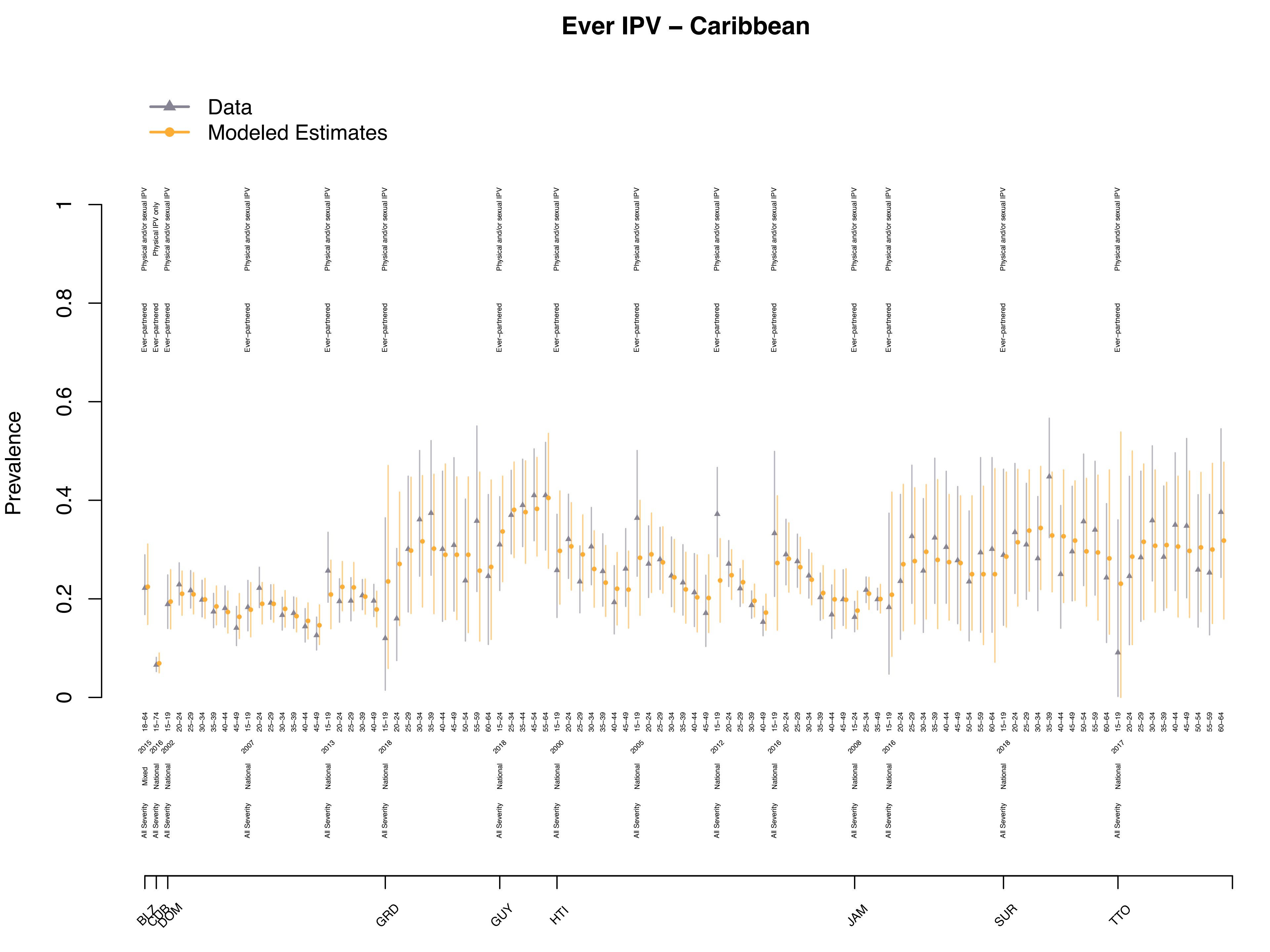

Ever IPV – Caribbean
Data
Modeled Estimates
Prevalence
BLZ
CUB
DOM
GRD
GUY
HTI
JAM
SUR
TTO

Ever IPV – Europe, Central

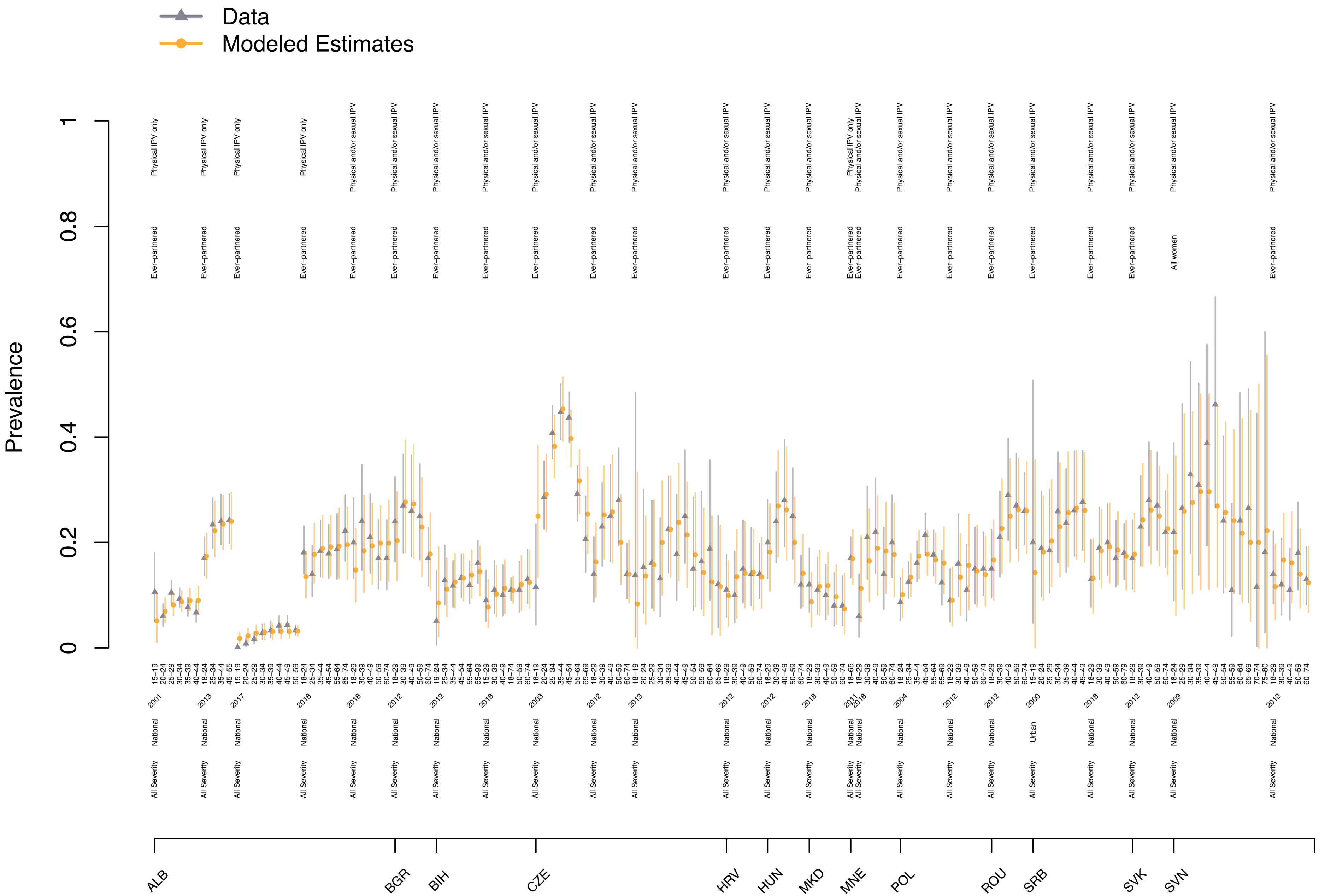

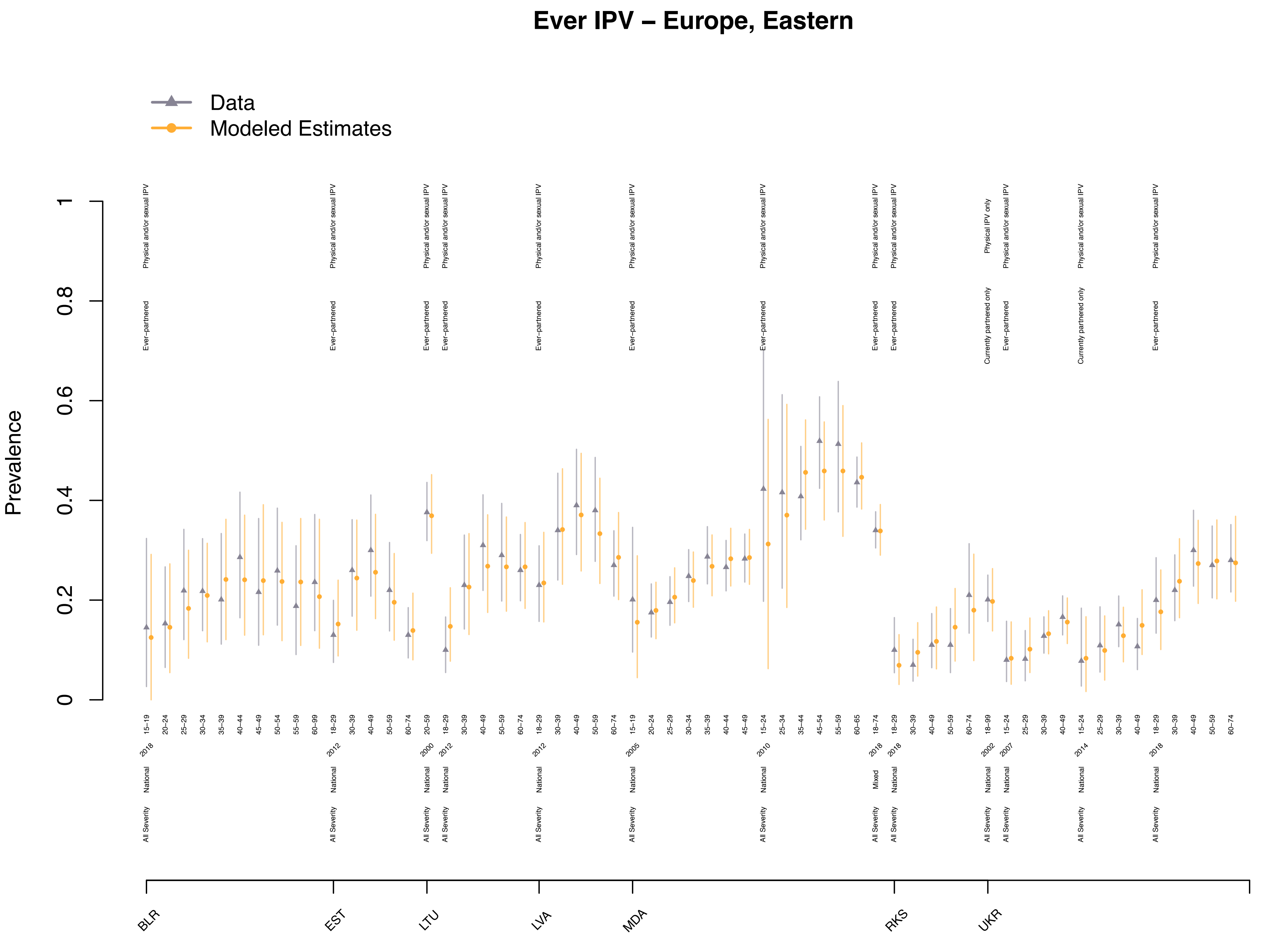

Ever IPV – Europe, Eastern
Data
Modeled Estimates
Prevalence
BLR
EST
LTU
LVA
MDA
RKS
UKR

Ever IPV – Europe, Western

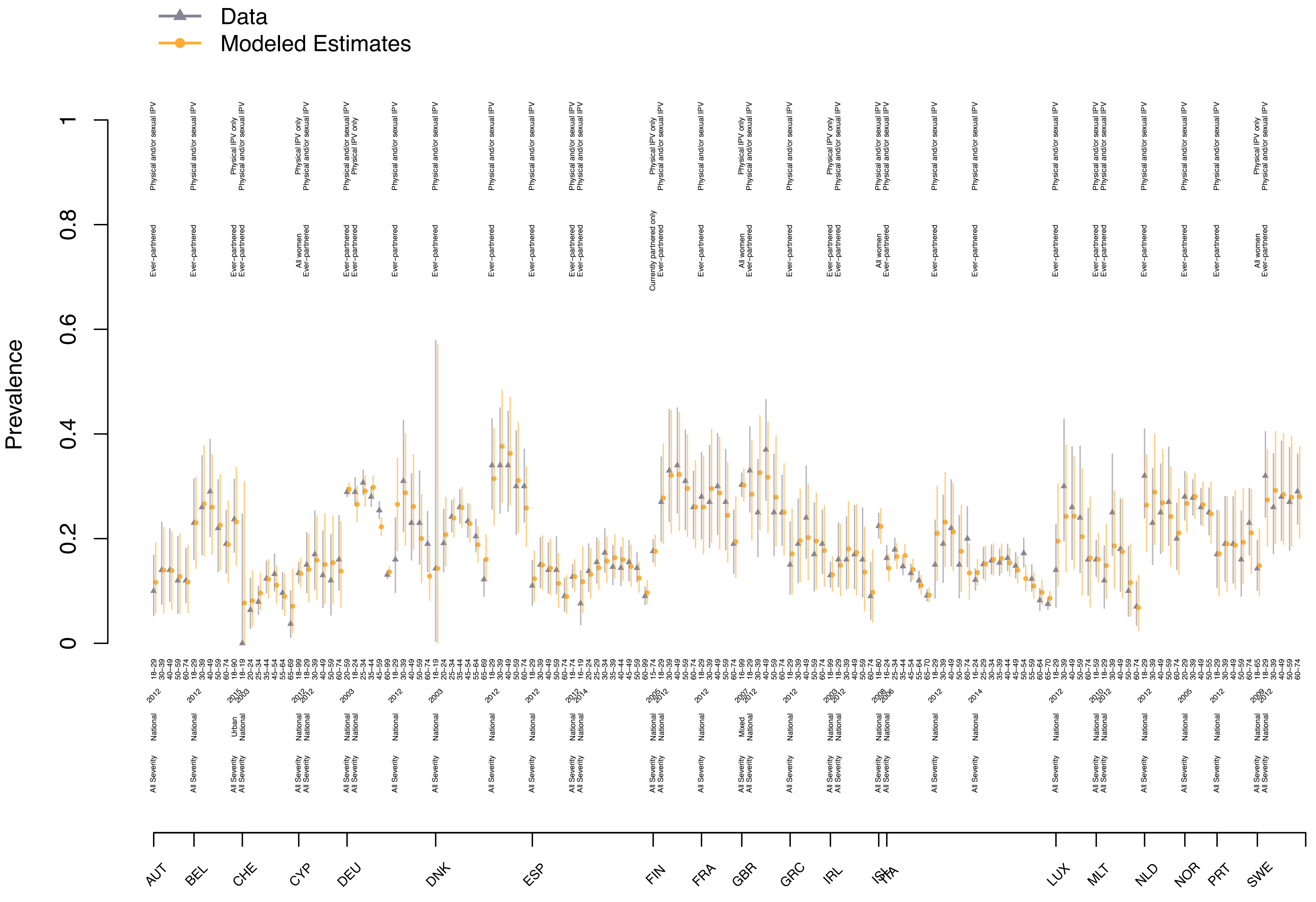

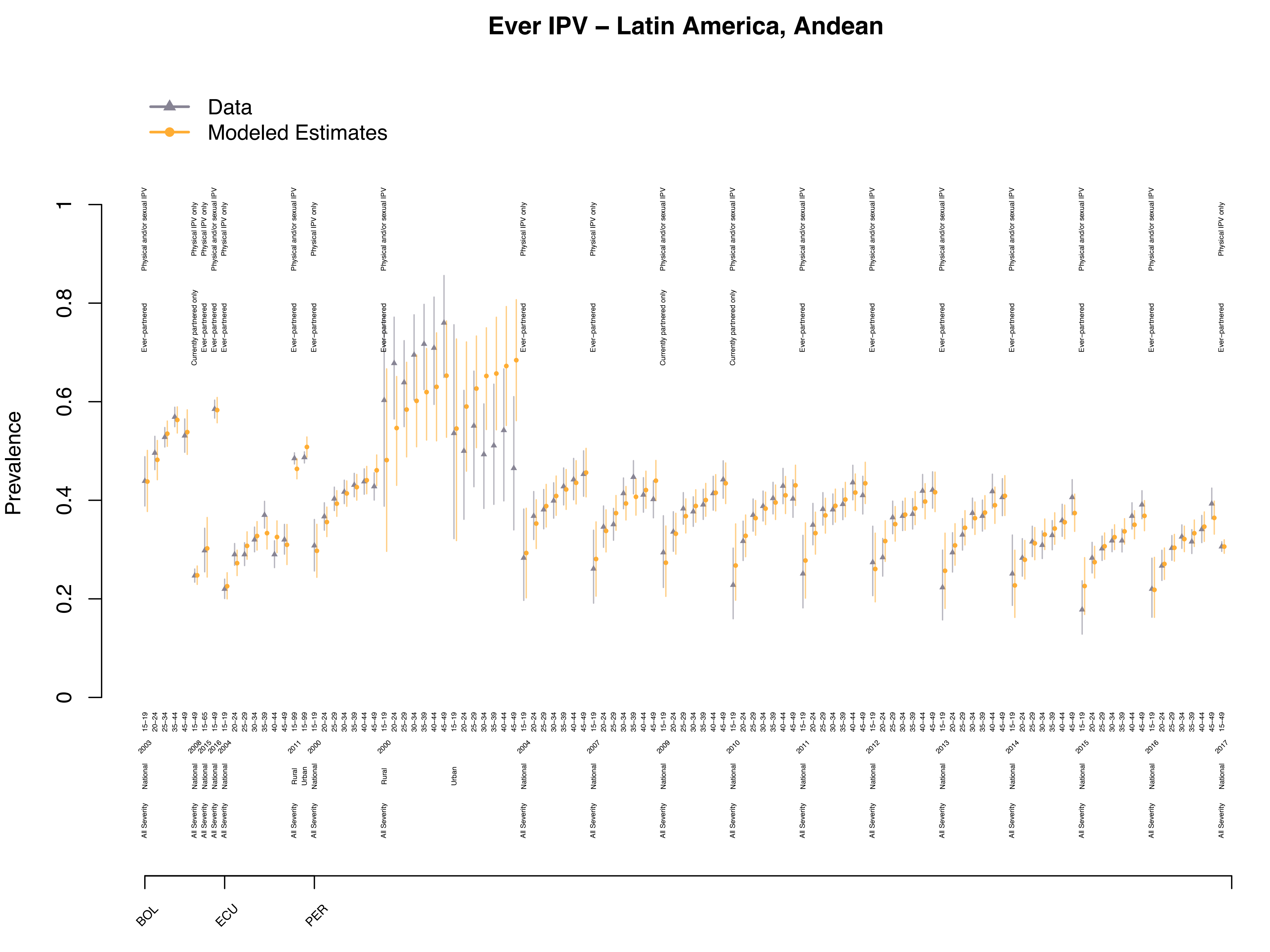

Ever IPV – Latin America, Andean
Data
Modeled Estimates
Prevalence
BOL
ECU
PER

Ever IPV – Latin America, Central

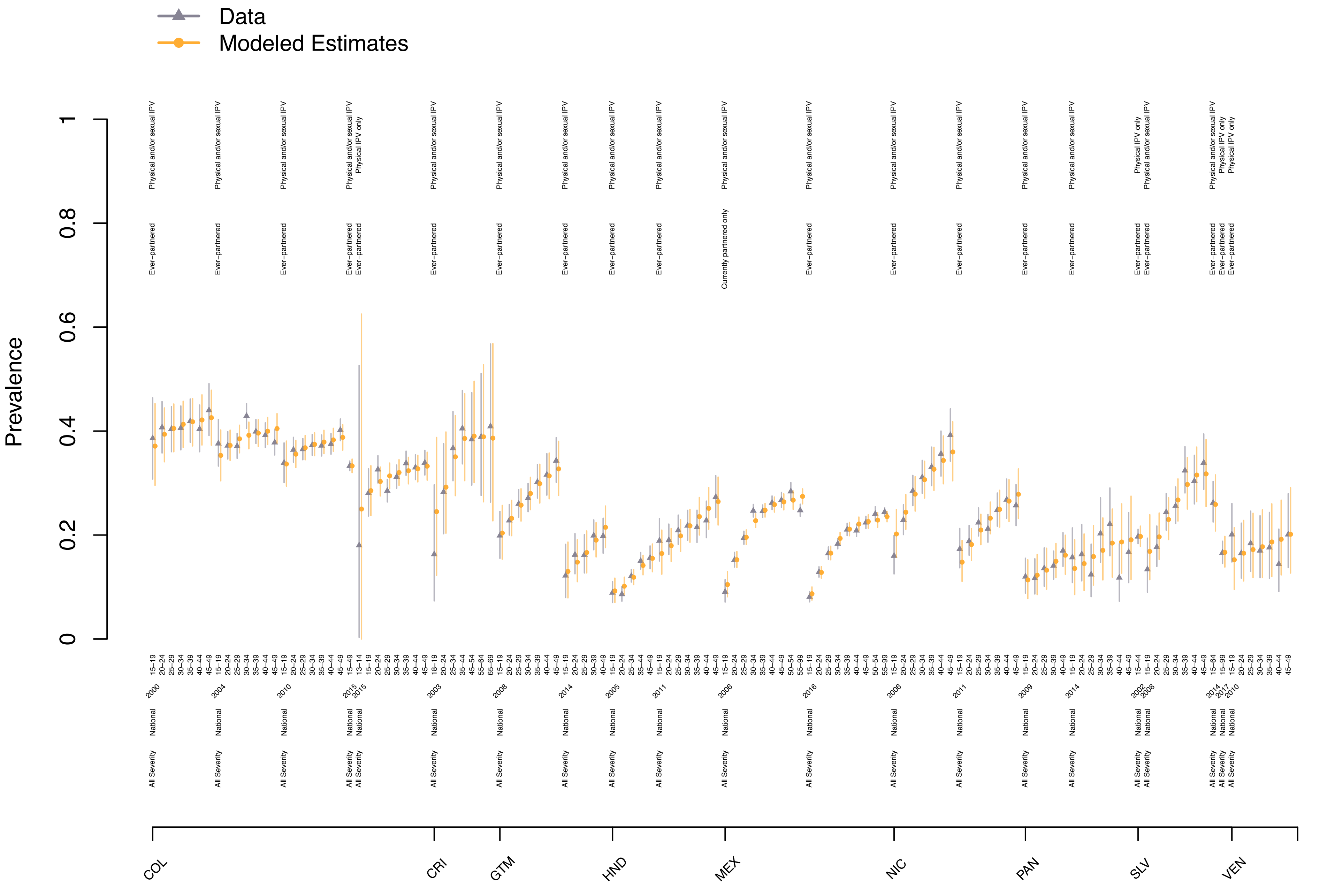

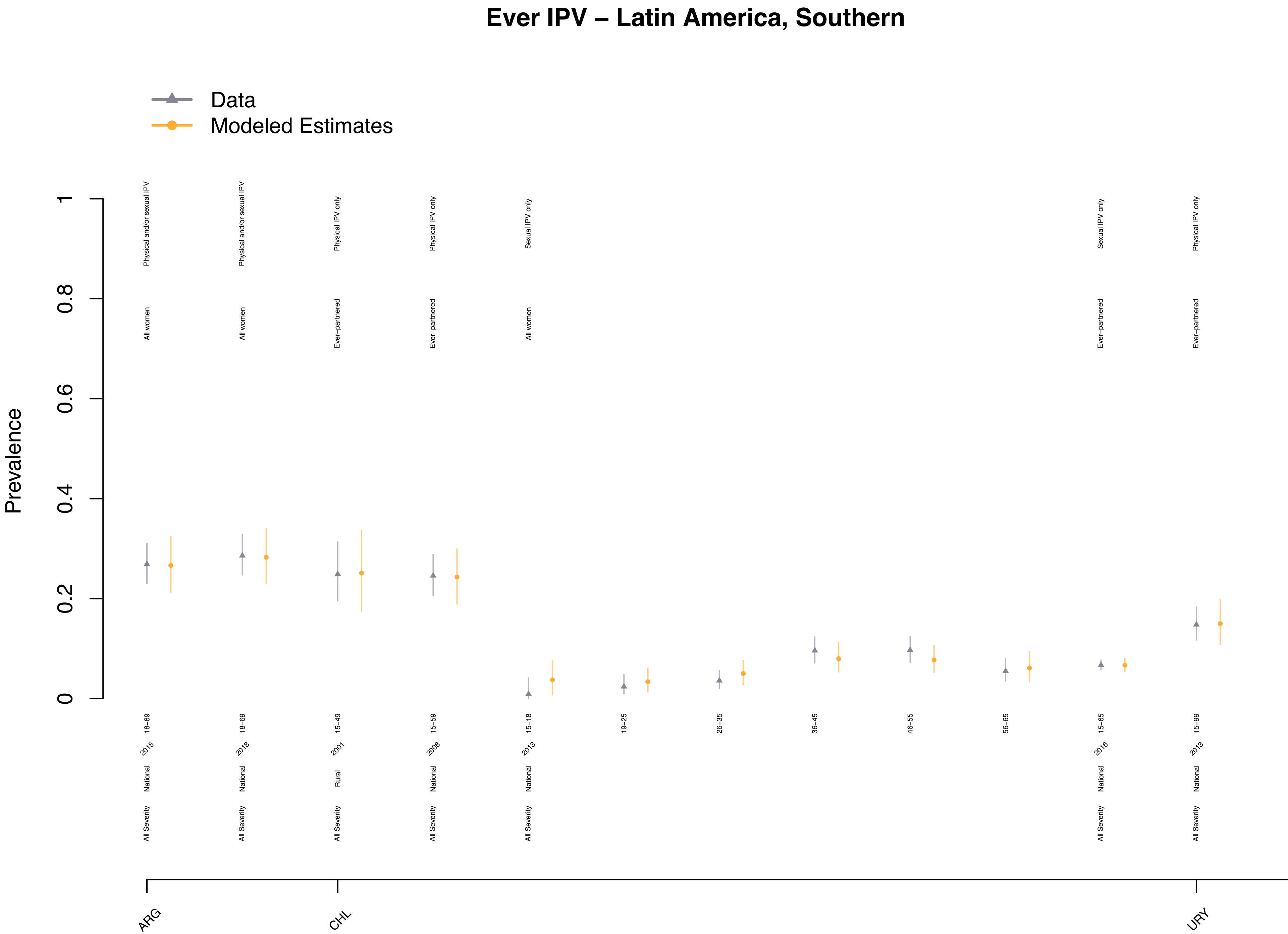

Ever IPV – Latin America, Southern
Data
Modeled Estimates
Prevalence
0
0.2
0.4
0.6
0.8
1
Physical and/or sexual IPV
Physical and/or sexual IPV
Physical IPV only
Physical IPV only
Sexual IPV only
Sexual IPV only
Physical IPV only
All women
All women
Ever−partnered
Ever−partnered
All women
Ever−partnered
Ever−partnered
18−69
18−69
15−49
15−59
15−18
19−25
26−35
36−45
46−55
56−65
15−65
15−99
2015
2018
2001
2008
2013
2016
2013
National
National
Rural
National
National
National
National
All Severity
All Severity
All Severity
All Severity
All Severity
All Severity
All Severity
ARG
CHL
URY

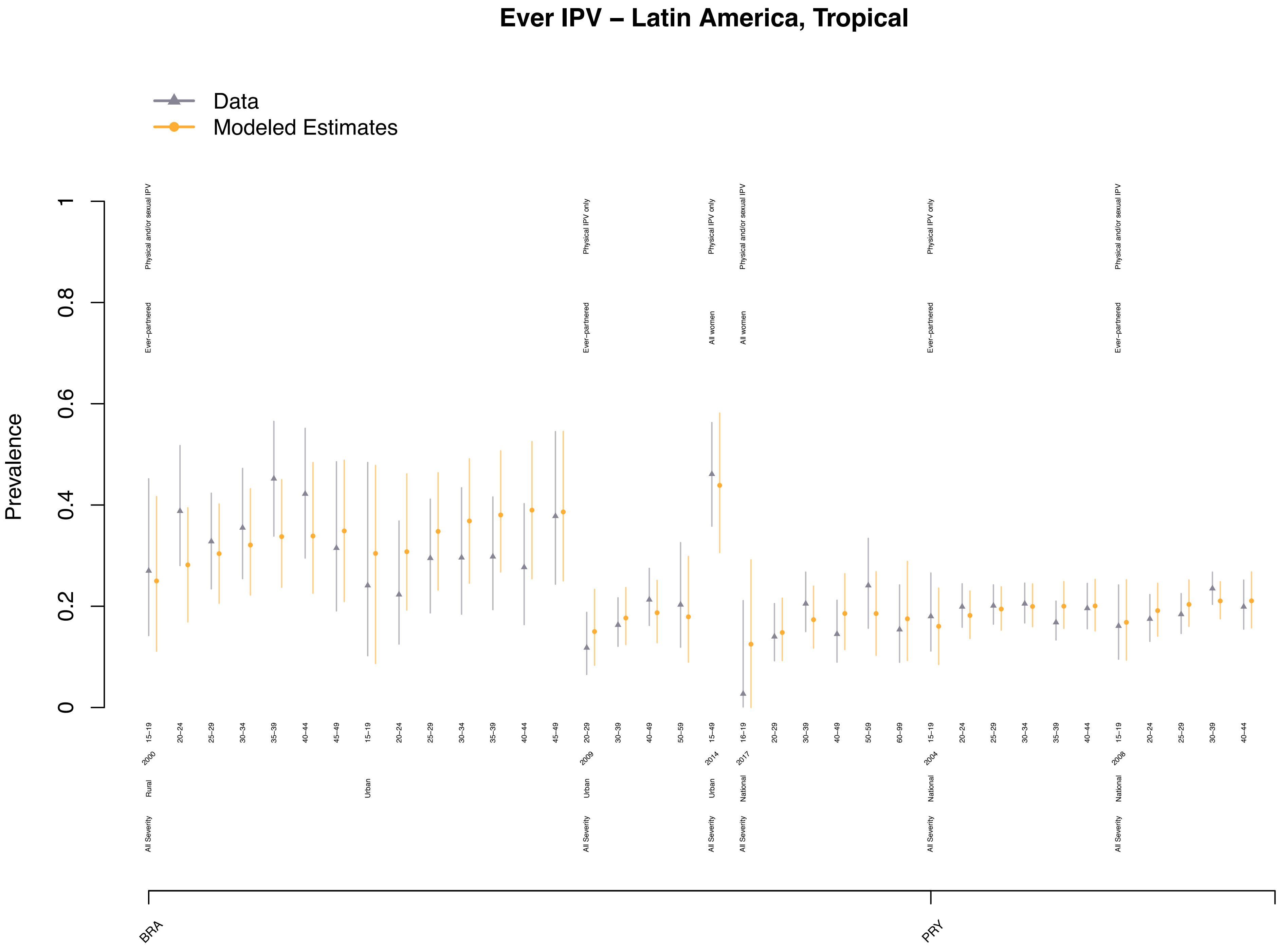

Ever IPV – Latin America, Tropical
Data
Modeled Estimates
Prevalence
BRA
PRY

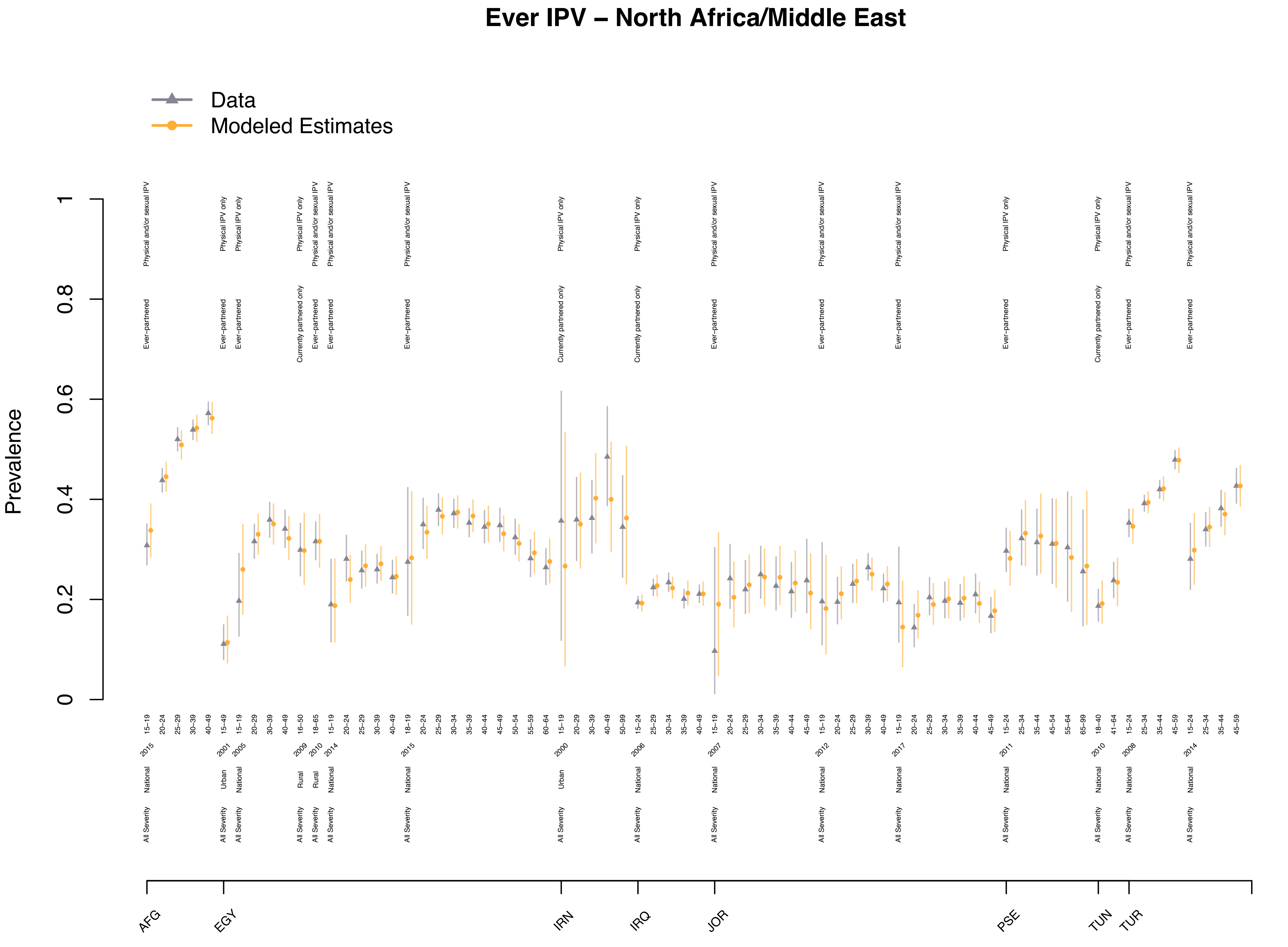

Ever IPV – North Africa/Middle East
Data
Modeled Estimates
Prevalence
0
0.2
0.4
0.6
0.8
1
AFG
EGY
IRN
IRQ
JOR
PSE
TUN
TUR

Ever IPV – North America, High Income

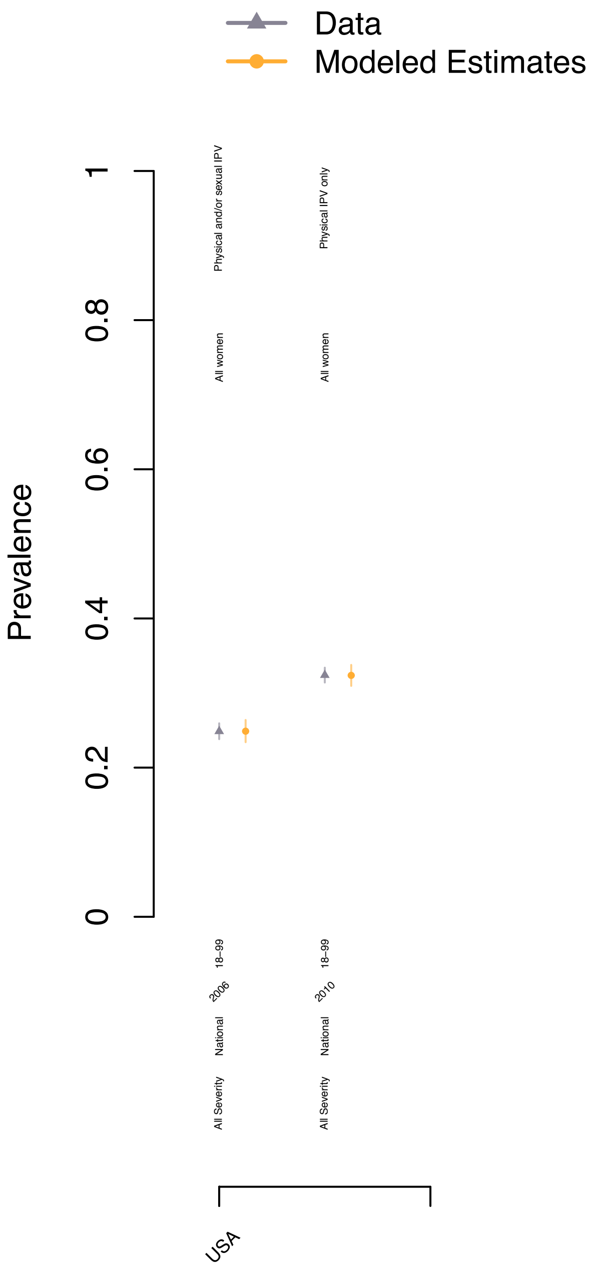

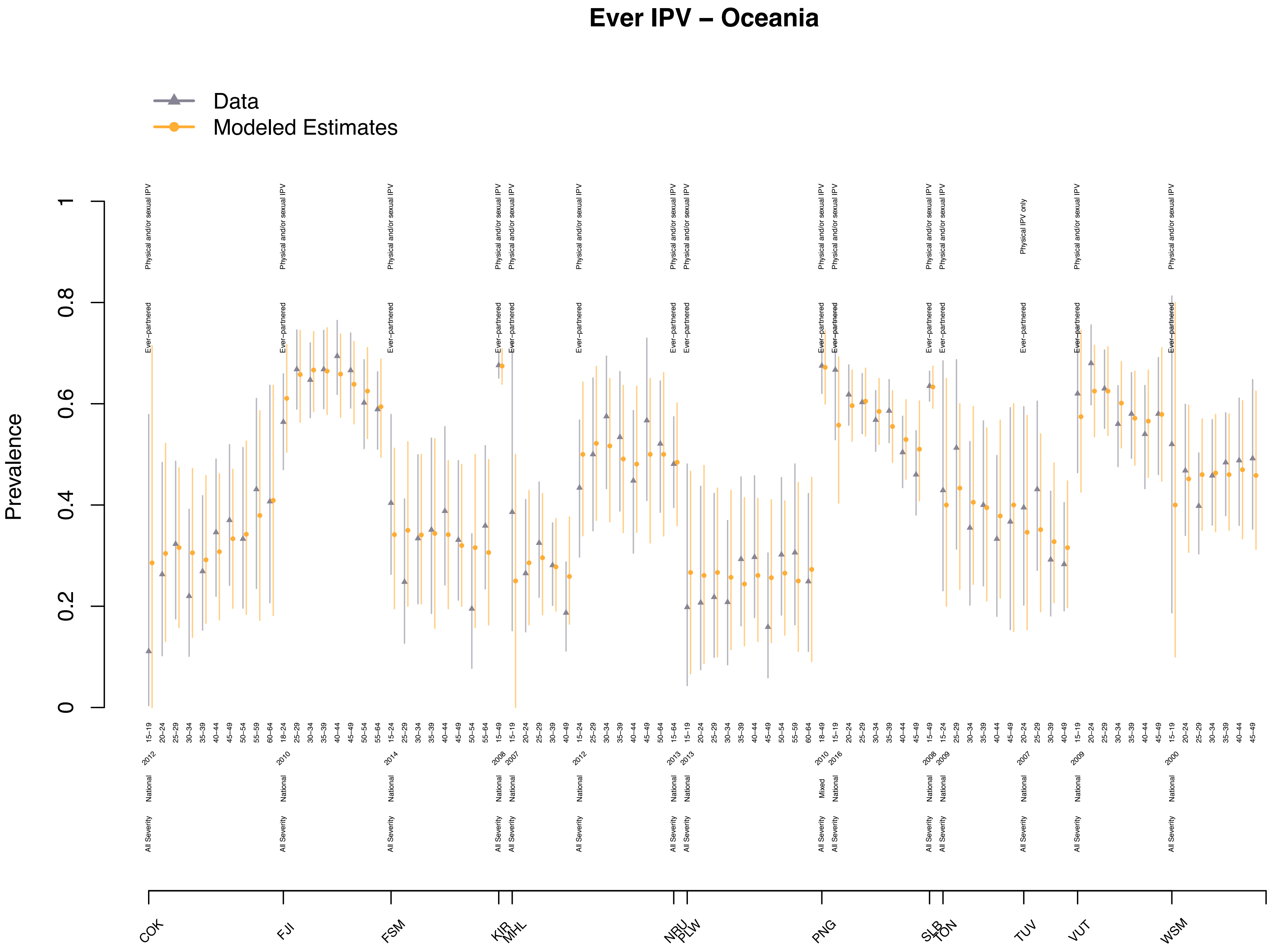

Ever IPV – Oceania
Data
Modeled Estimates
Prevalence
0
0.2
0.4
0.6
0.8
1
COK
FJI
FSM
KIR
MHL
NRU
PLW
PNG
SLB
TON
TUV
VUT
WSM
Physical and/or sexual IPV
Physical IPV only
Ever−partnered
National
Mixed
All Severity

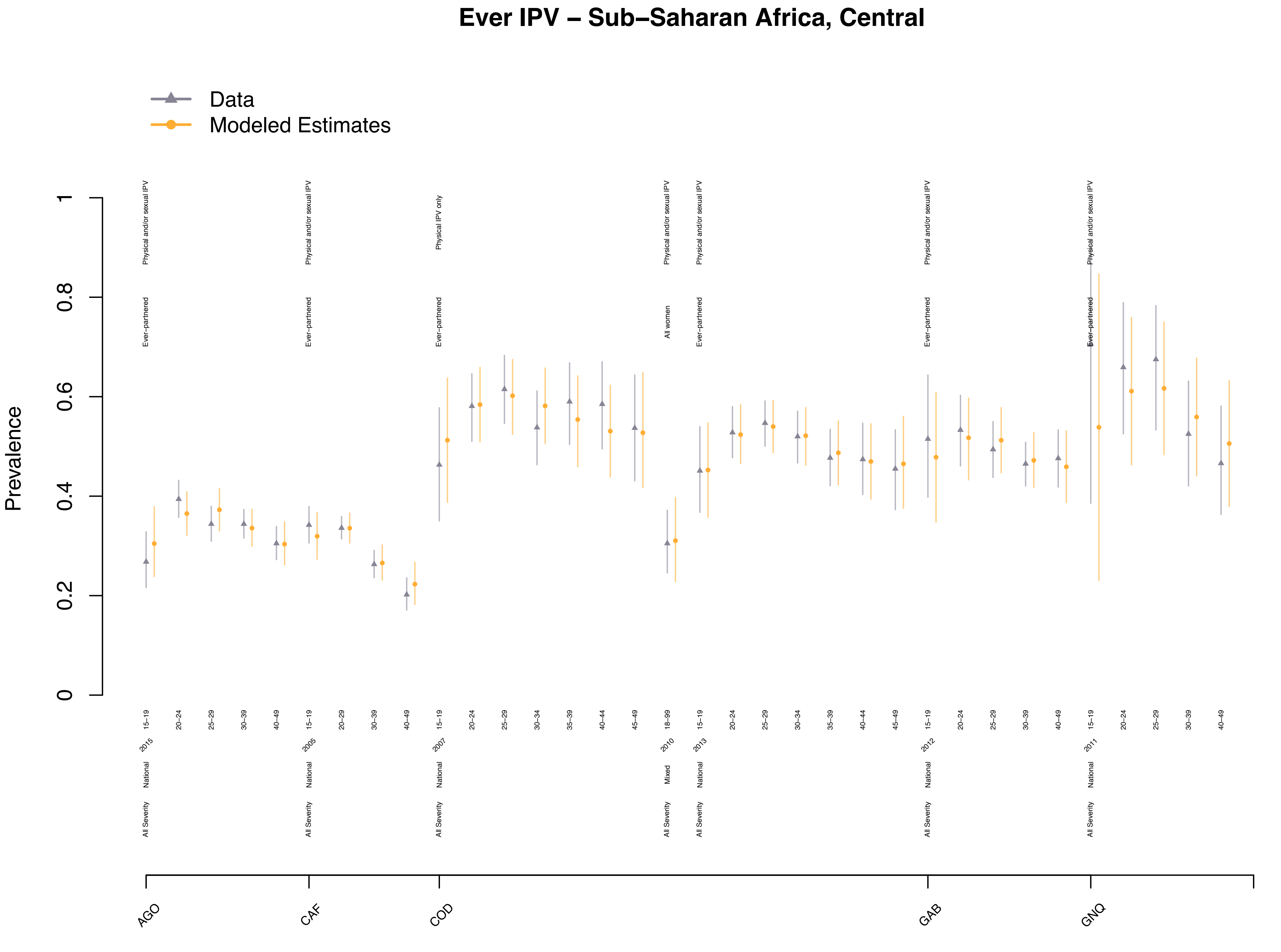

Ever IPV – Sub–Saharan Africa, Central
Data
Modeled Estimates
Prevalence
0
0.2
0.4
0.6
0.8
1
Physical and/or sexual IPV
Physical IPV only
Ever–partnered
All women
15–19
20–24
25–29
30–39
40–49
20–29
30–34
35–39
40–44
45–49
18–99
2015
2005
2007
2010
2013
2012
2011
National
Mixed
All Severity
AGO
CAF
COD
GAB
GNQ

Ever IPV – Sub–Saharan Africa, East

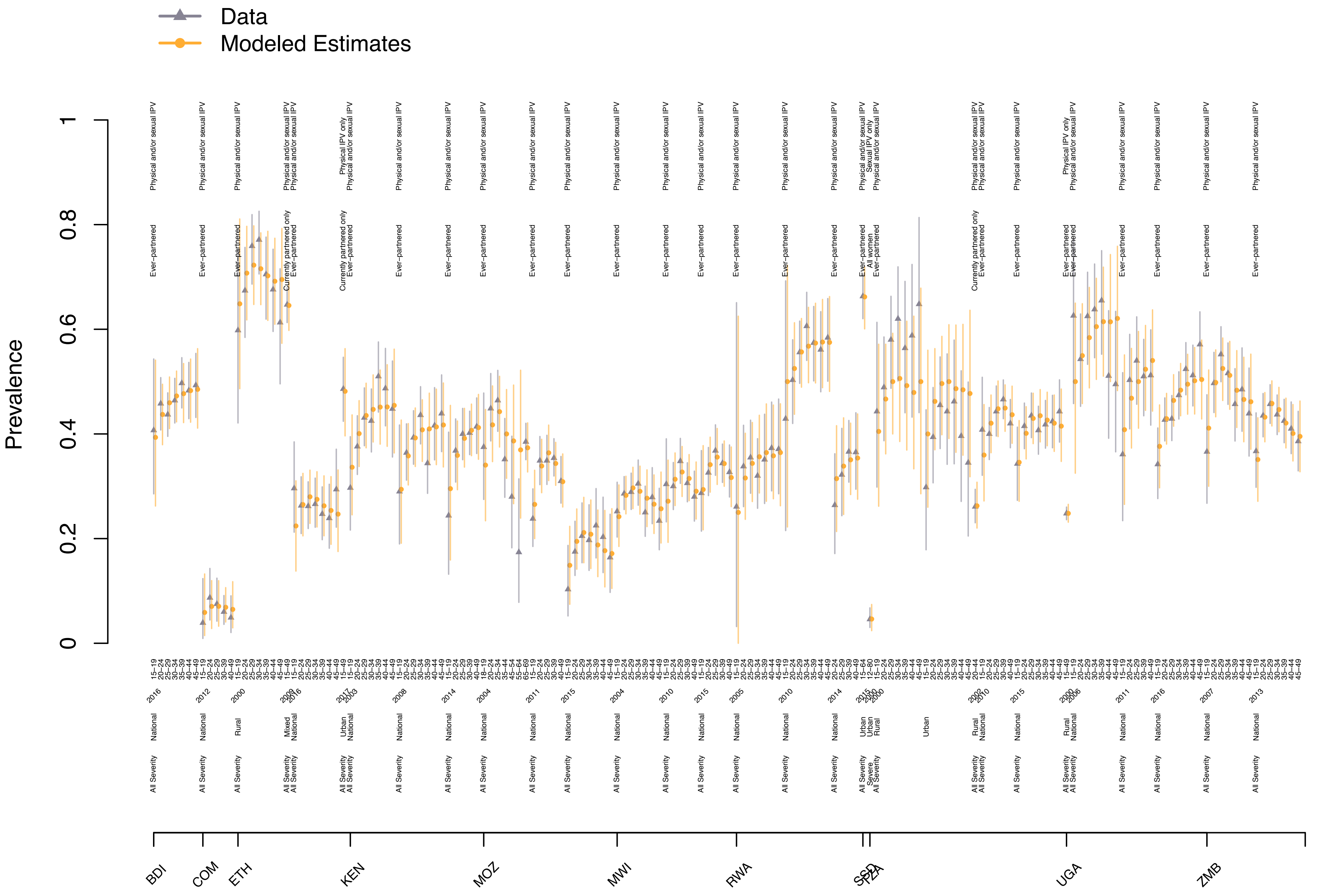

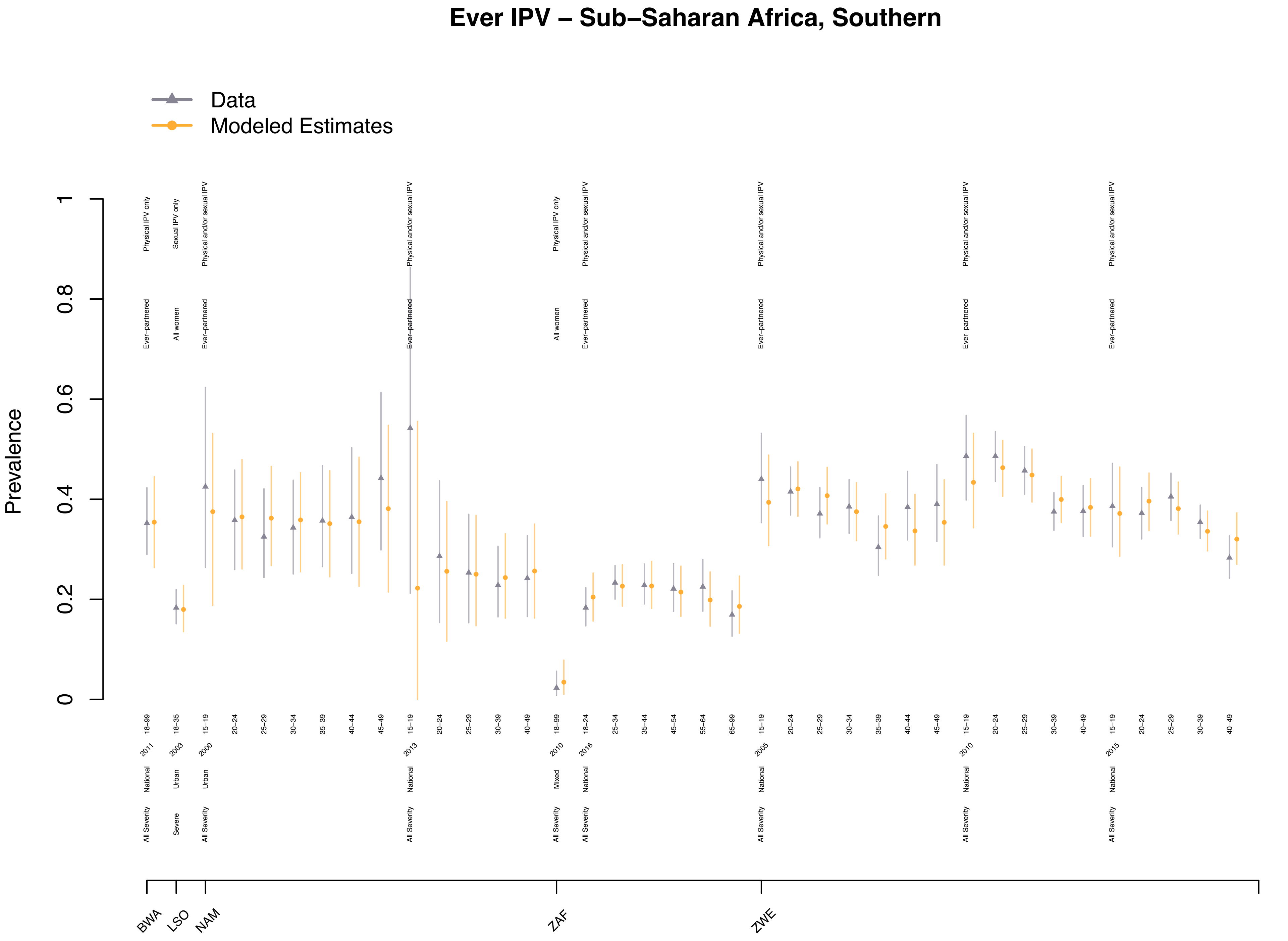
Ever IPV – Sub–Saharan Africa, Southern
Data
Modeled Estimates
Prevalence
0
0.2
0.4
0.6
0.8
1
BWA
LSO
NAM
ZAF
ZWE

Ever IPV – Sub–Saharan Africa, West

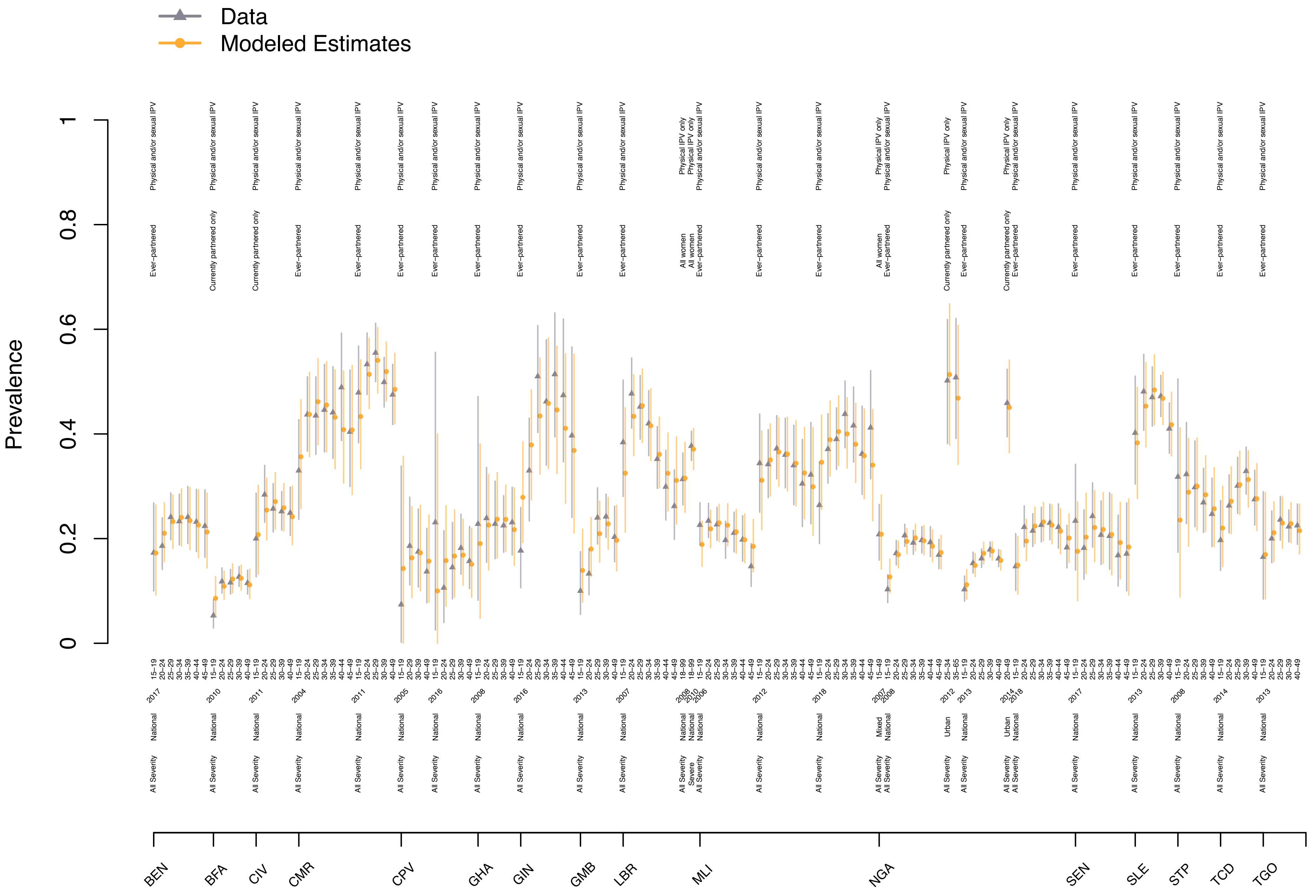

Figure S17. Posterior predictive checks for the past year intimate partner violence (IPV) model.

Past Year IPV – Asia Pacific, High Income

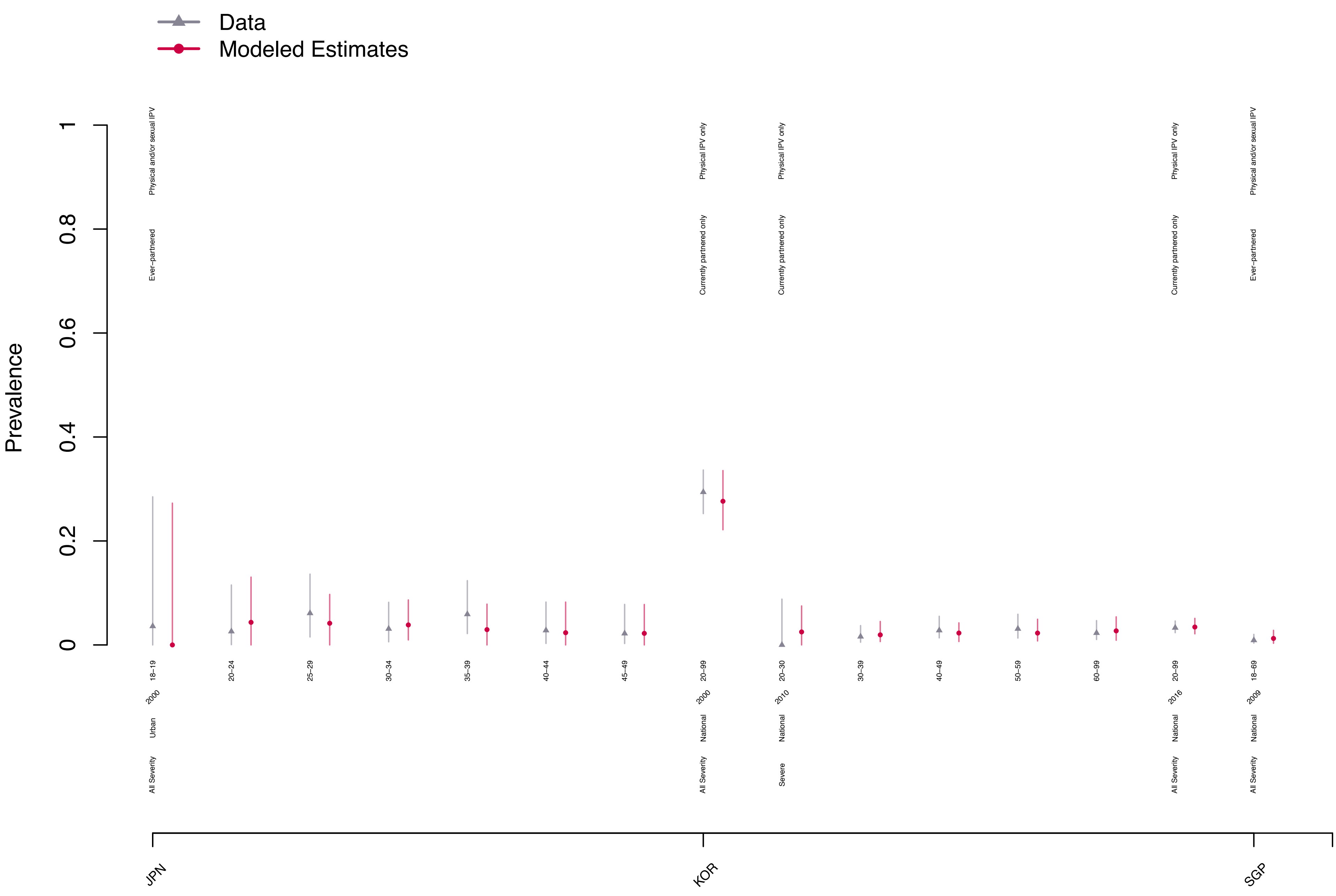

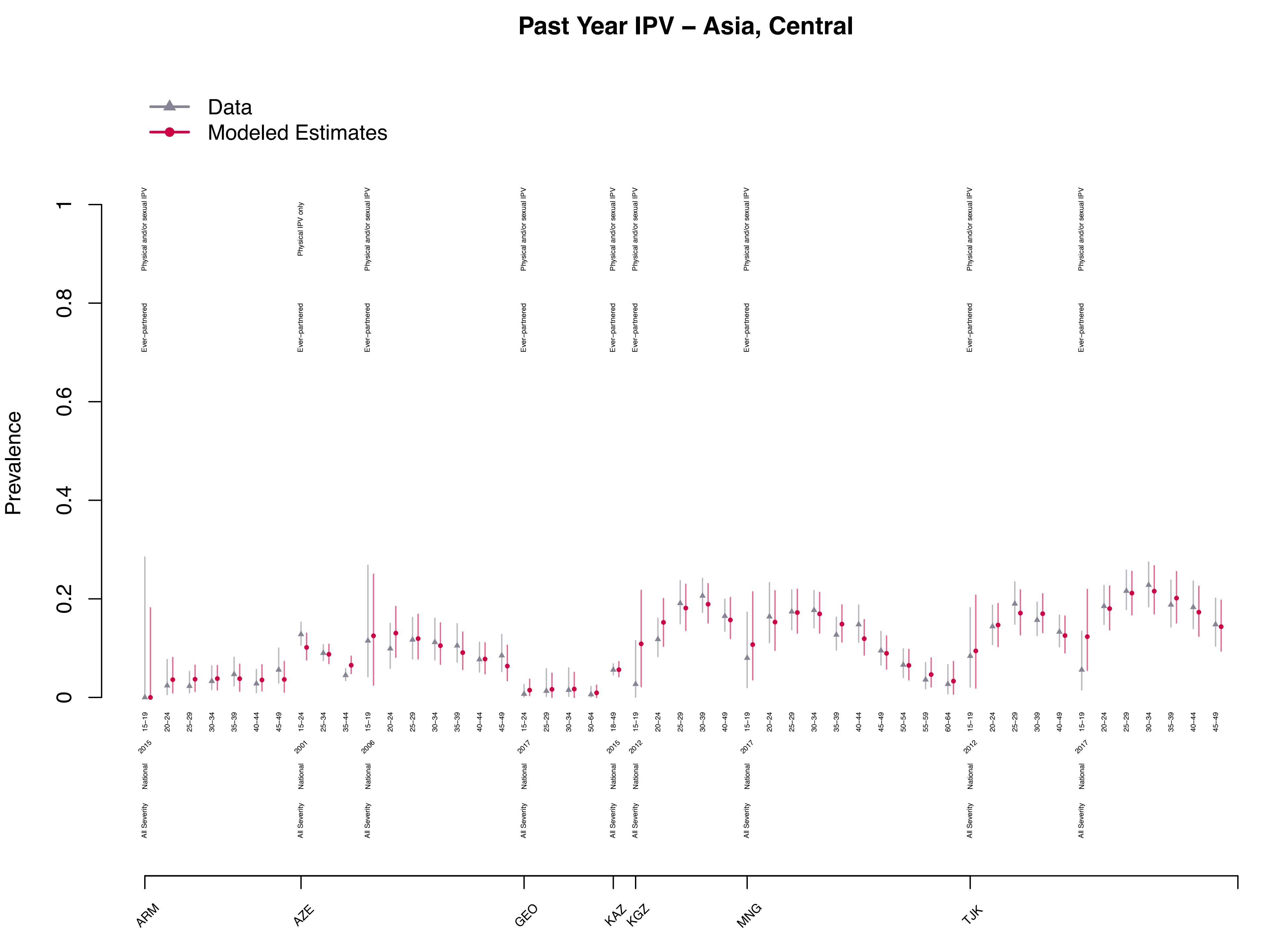

Past Year IPV – Asia, Central
Data
Modeled Estimates
Prevalence
0
0.2
0.4
0.6
0.8
1
ARM
AZE
GEO
KAZ
KGZ
MNG
TJK

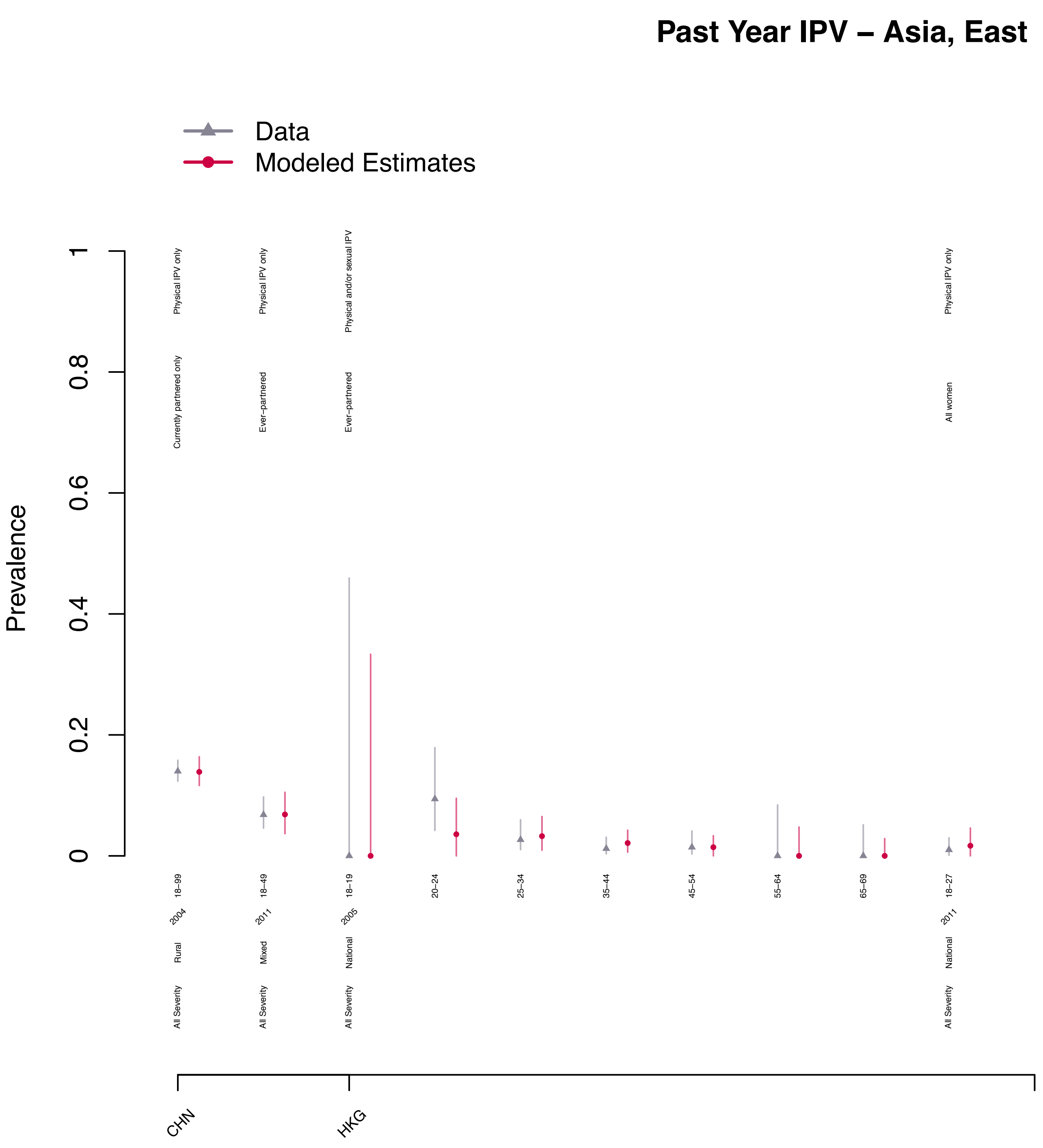

Past Year IPV – Asia, East
Data
Modeled Estimates
Prevalence
0
0.2
0.4
0.6
0.8
1
Physical IPV only
Currently partnered only
Physical IPV only
Ever–partnered
Physical and/or sexual IPV
Ever–partnered
Physical IPV only
All women
18–99
2004
Rural
All Severity
18–49
2011
Mixed
All Severity
18–19
2005
National
All Severity
20–24
25–34
35–44
45–54
55–64
65–69
18–27
2011
National
All Severity
CHN
HKG

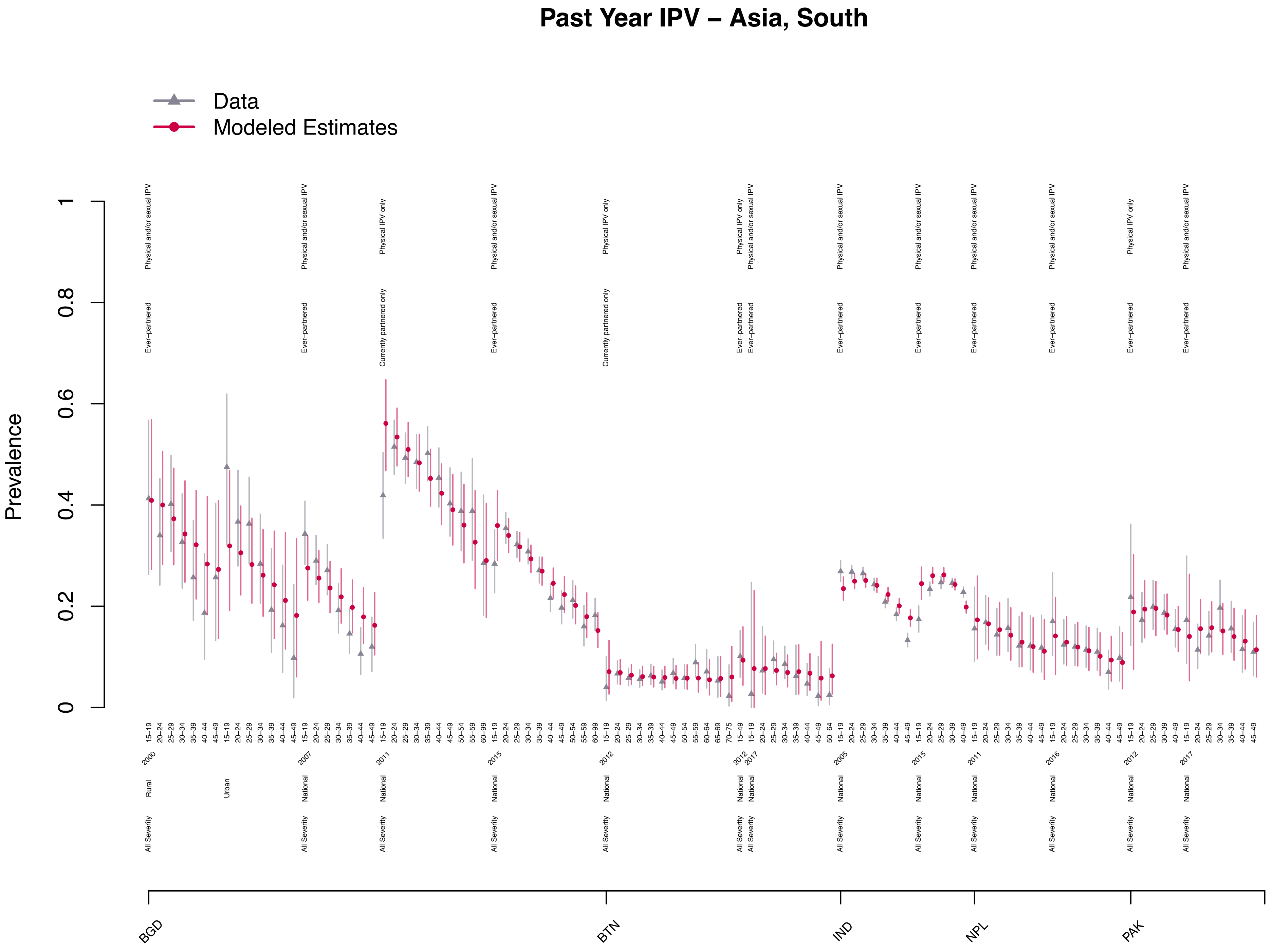

Past Year IPV – Asia, South
Data
Modeled Estimates
Prevalence
0
0.2
0.4
0.6
0.8
1
BGD
BTN
IND
NPL
PAK

Past Year IPV – Asia, Southeast

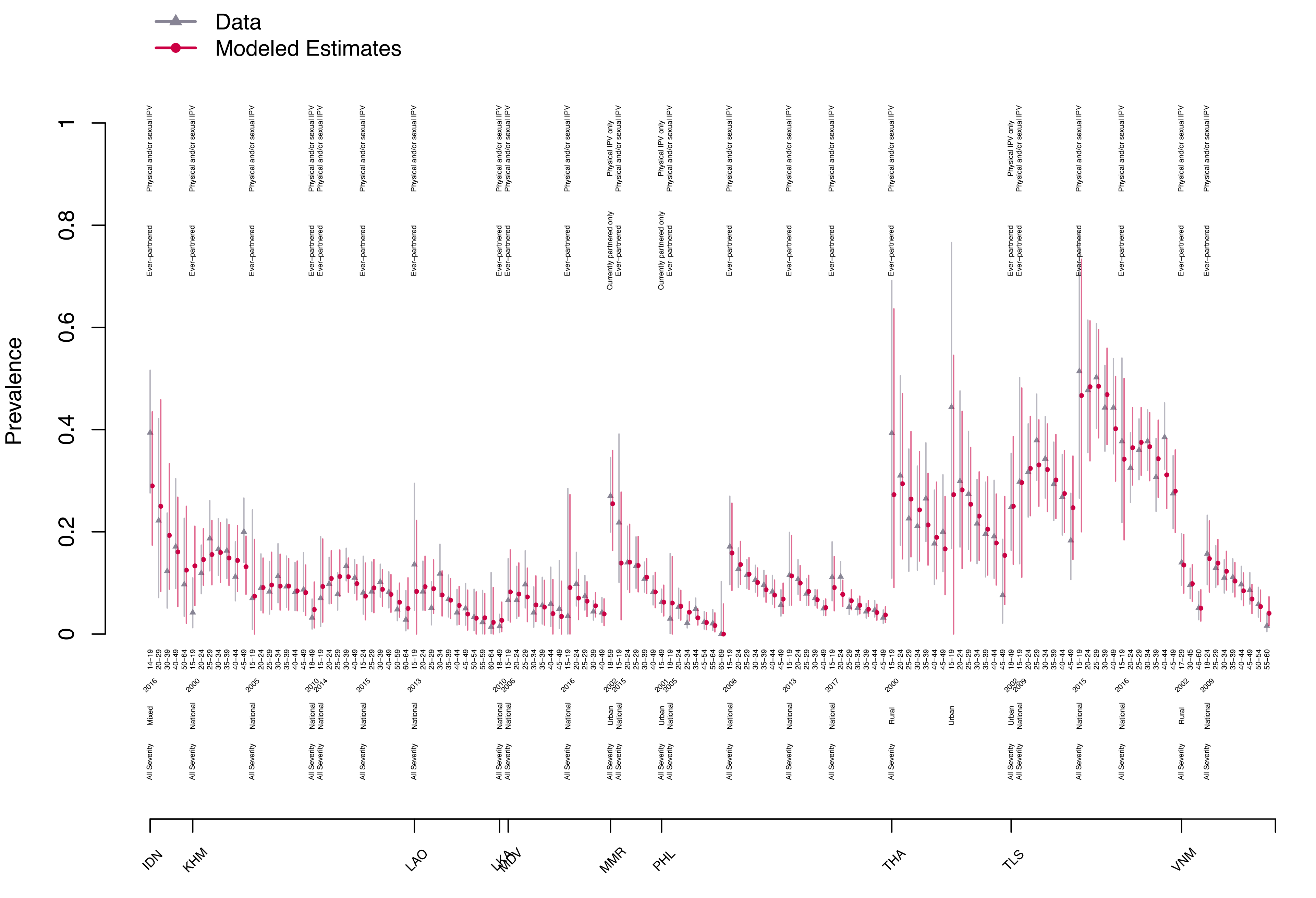

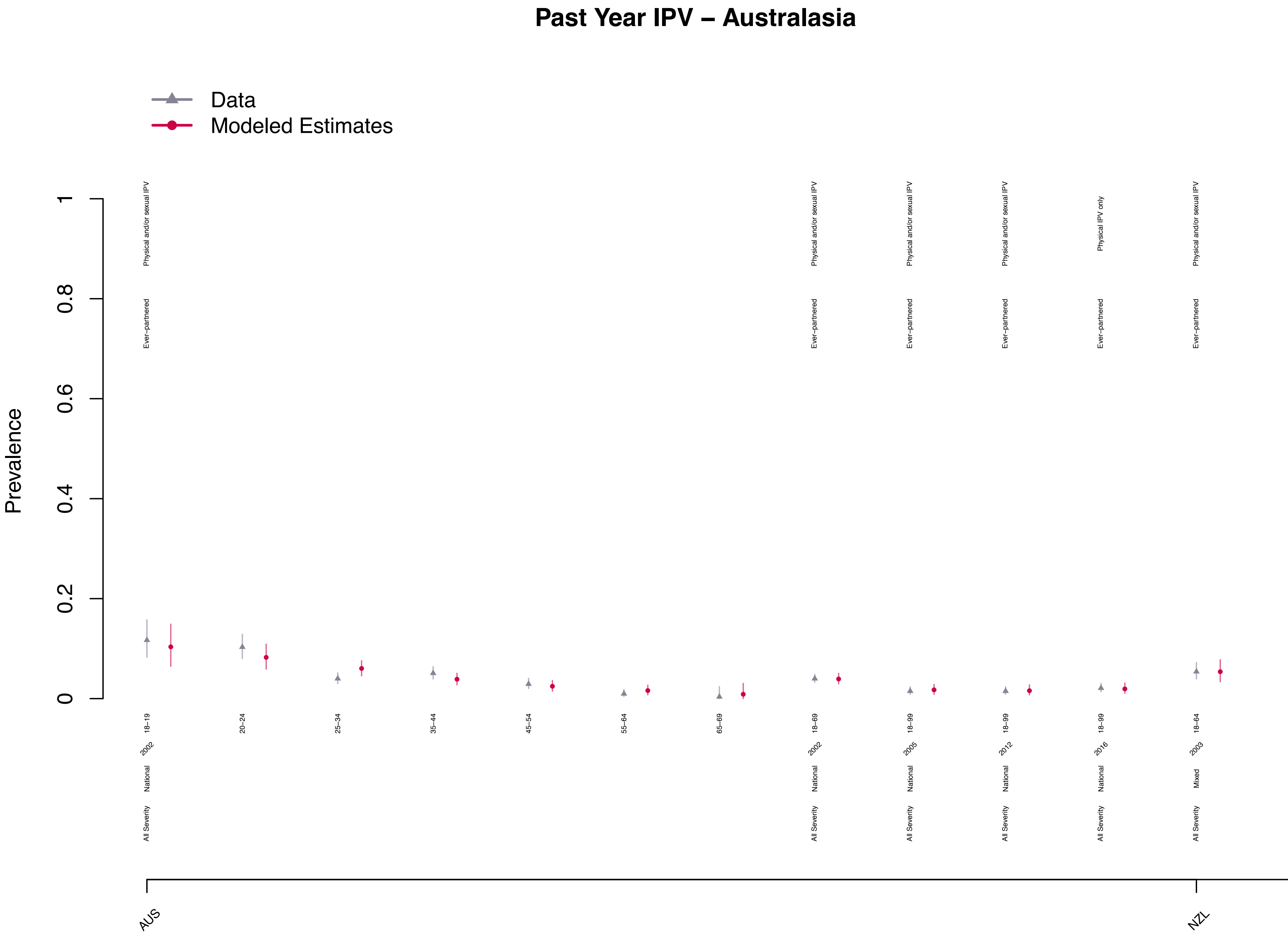
Past Year IPV – Australasia
Data
Modeled Estimates
Prevalence
0
0.2
0.4
0.6
0.8
1
Physical and/or sexual IPV
Physical IPV only
Ever−partnered
18−19
20−24
25−34
35−44
45−54
55−64
65−69
18−69
18−99
18−64
2002
2005
2012
2016
2003
National
Mixed
All Severity
AUS
NZL

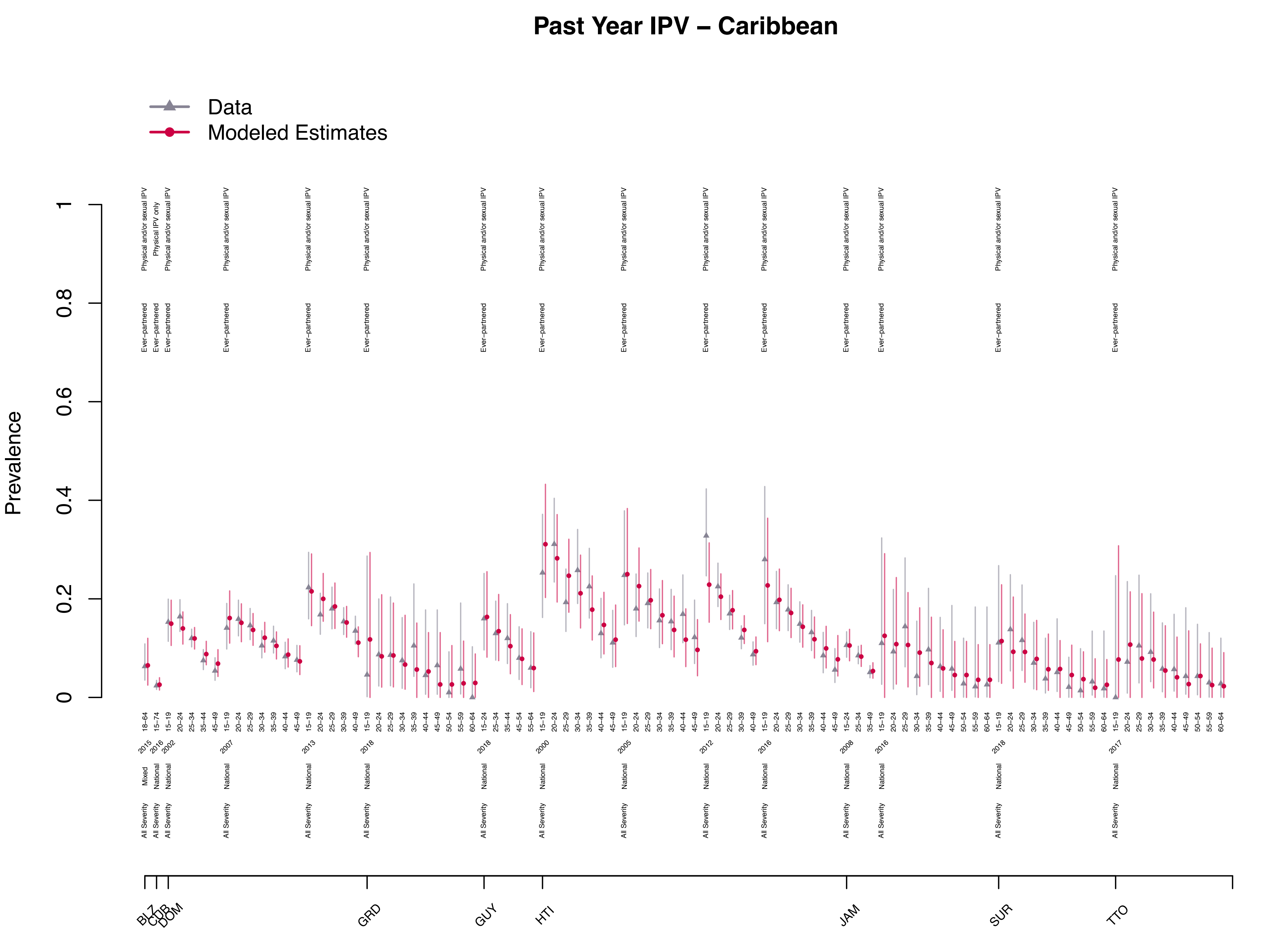

Past Year IPV – Caribbean
Data
Modeled Estimates
Prevalence
0
0.2
0.4
0.6
0.8
1
BLZ
CUB
DOM
GRD
GUY
HTI
JAM
SUR
TTO

Past Year IPV – Europe, Central

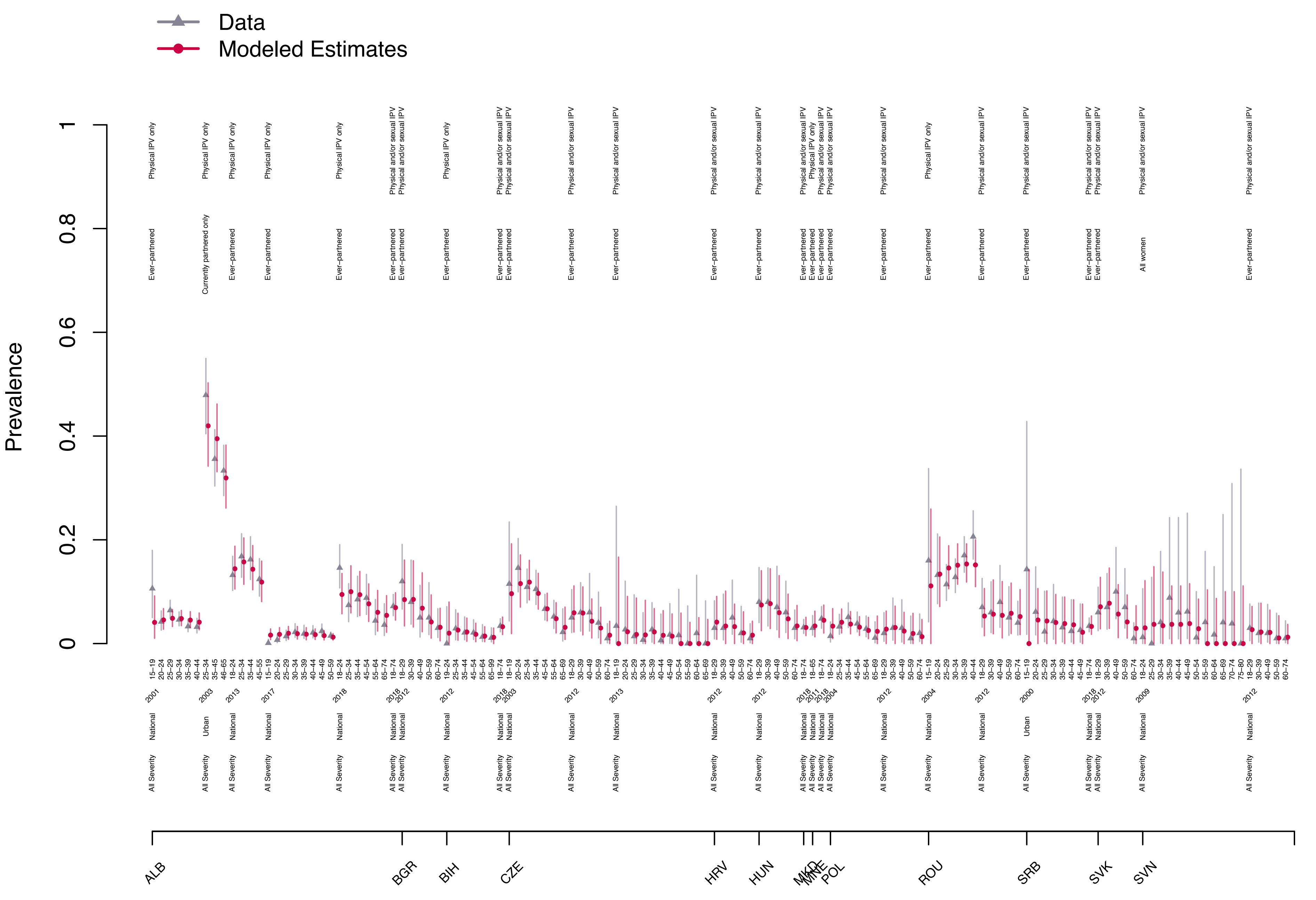

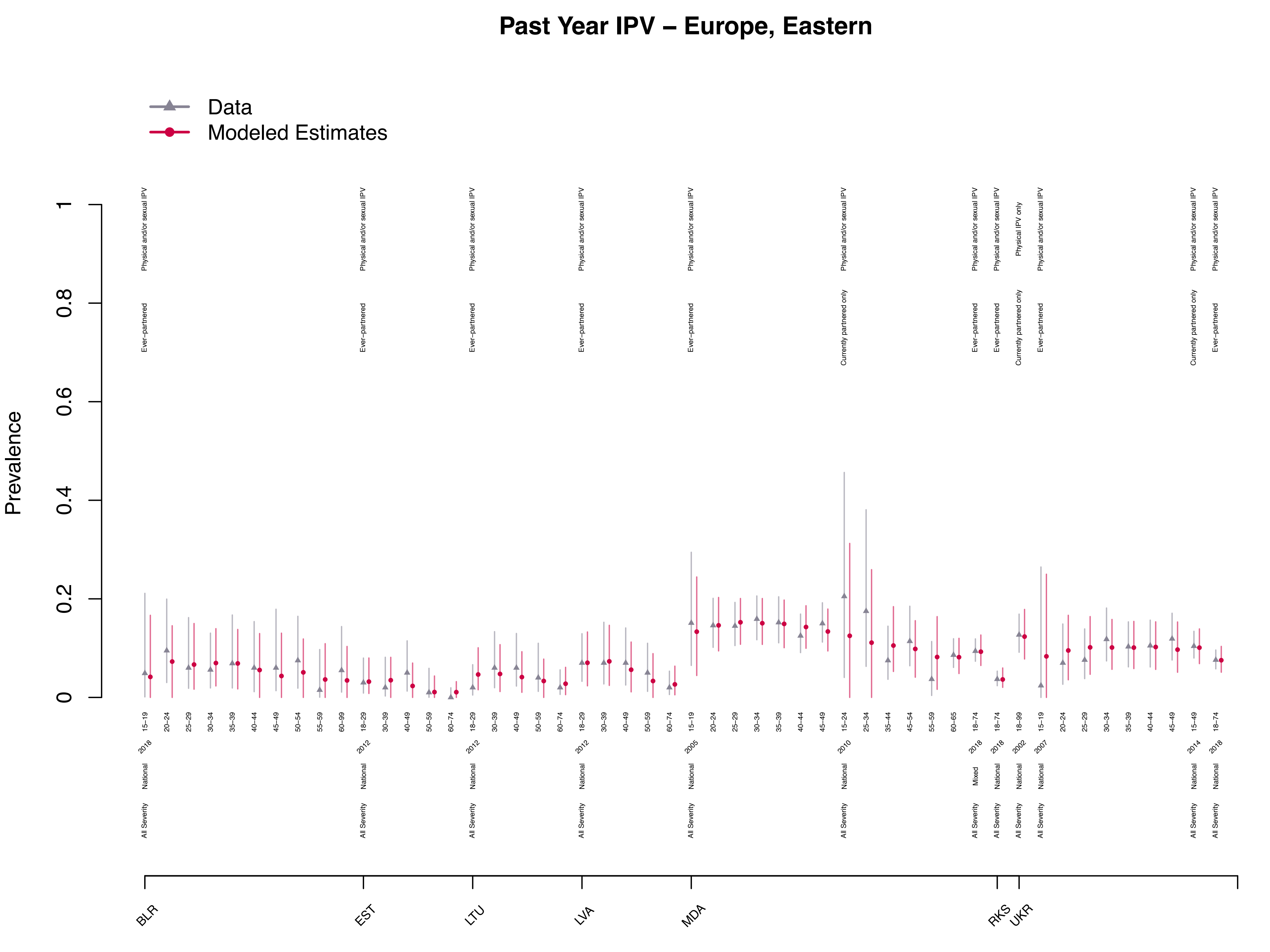

Past Year IPV – Europe, Eastern
Data
Modeled Estimates
Prevalence
0
0.2
0.4
0.6
0.8
1
BLR
EST
LTU
LVA
MDA
RKS
UKR

Past Year IPV – Europe, Western

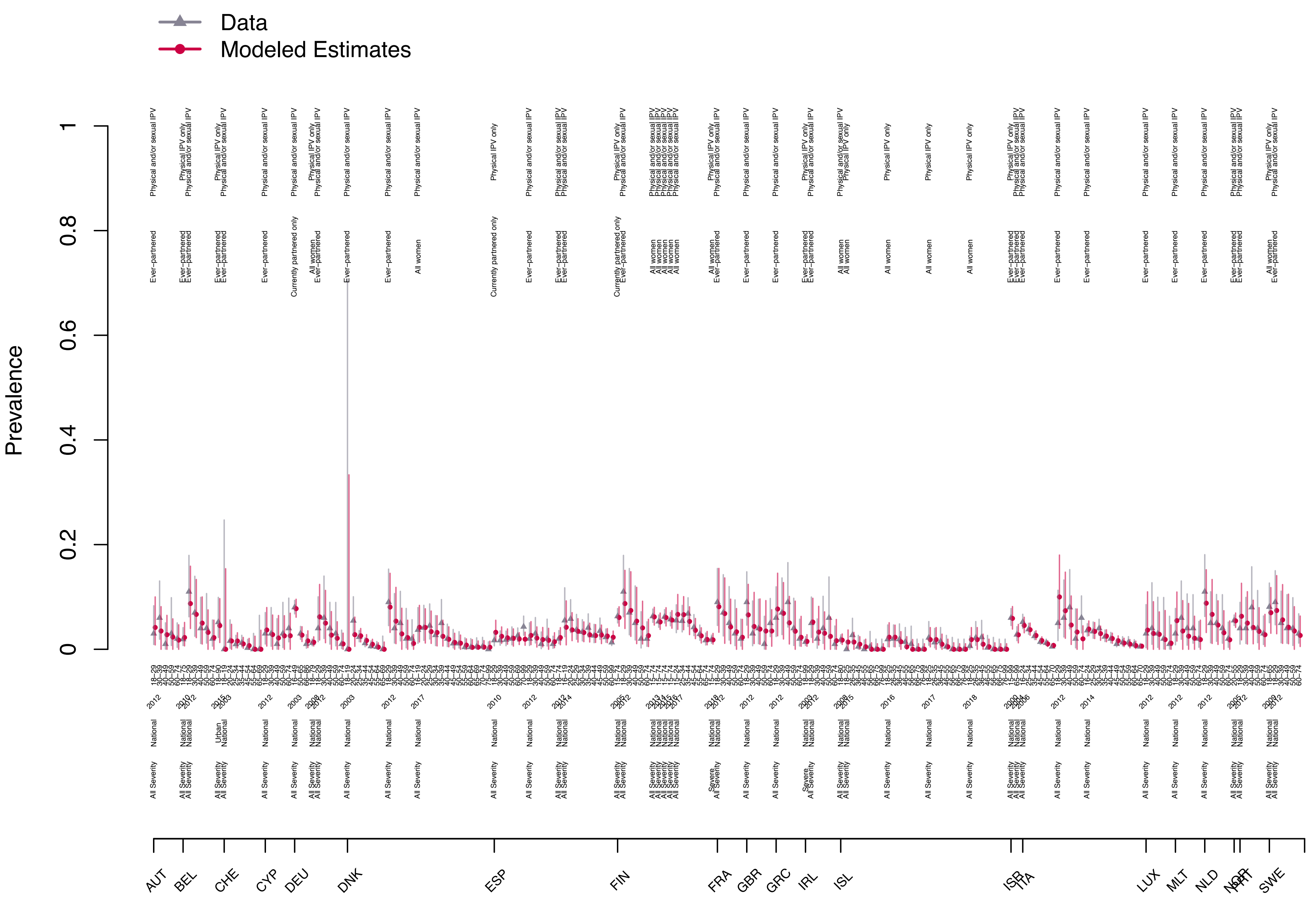

Past Year IPV – Latin America, Andean

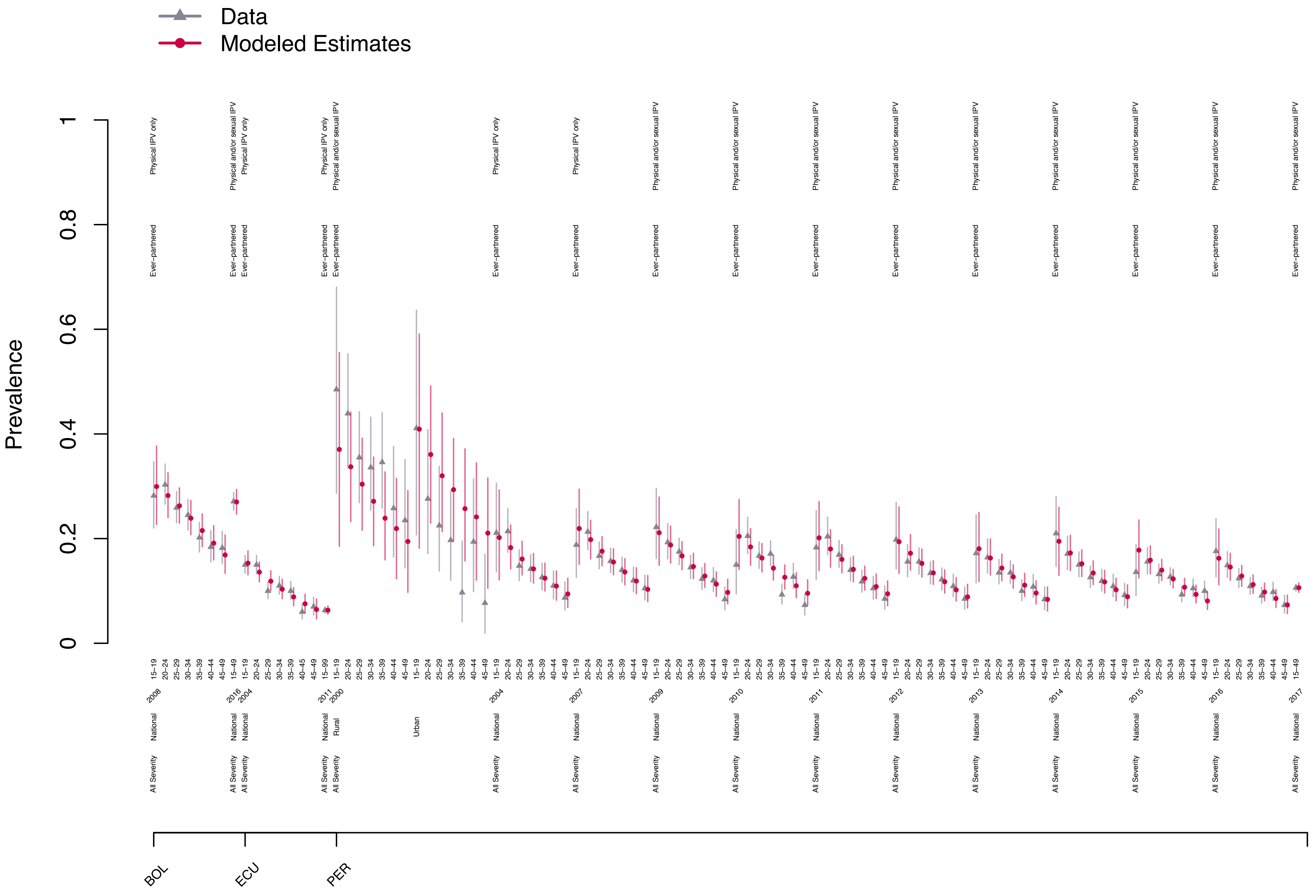

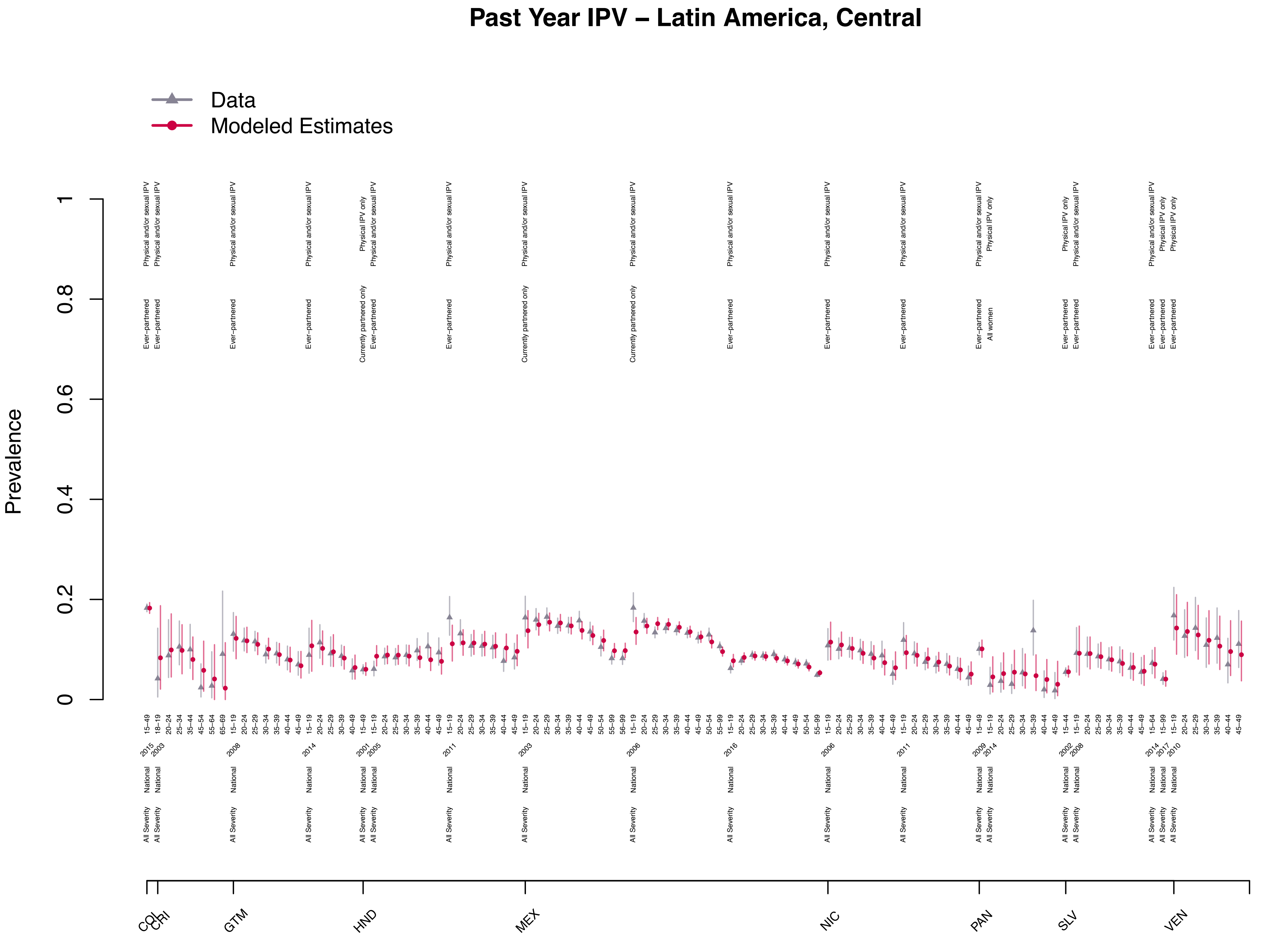

Past Year IPV – Latin America, Central
Data
Modeled Estimates
Prevalence
COL
CRI
GTM
HND
MEX
NIC
PAN
SLV
VEN

Past Year IPV – Latin America, Southern

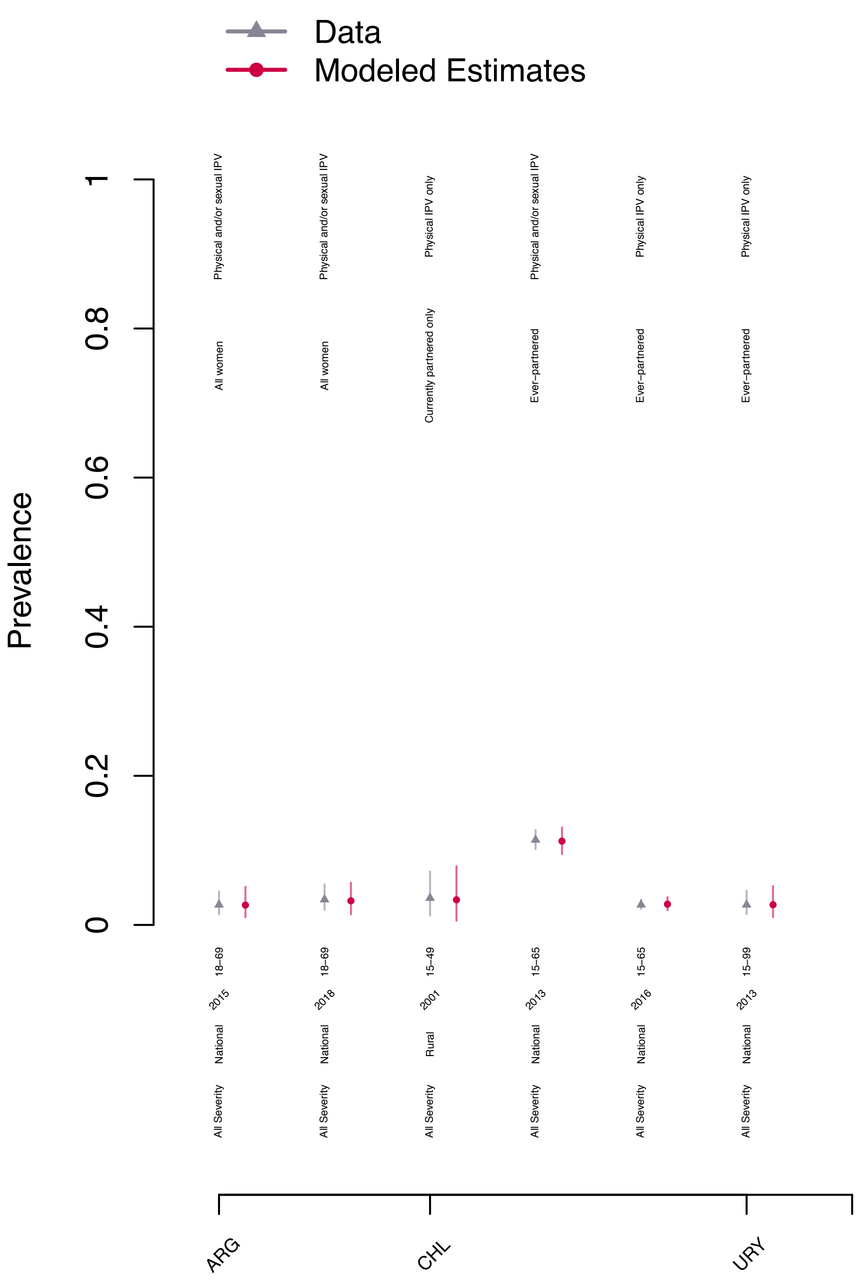

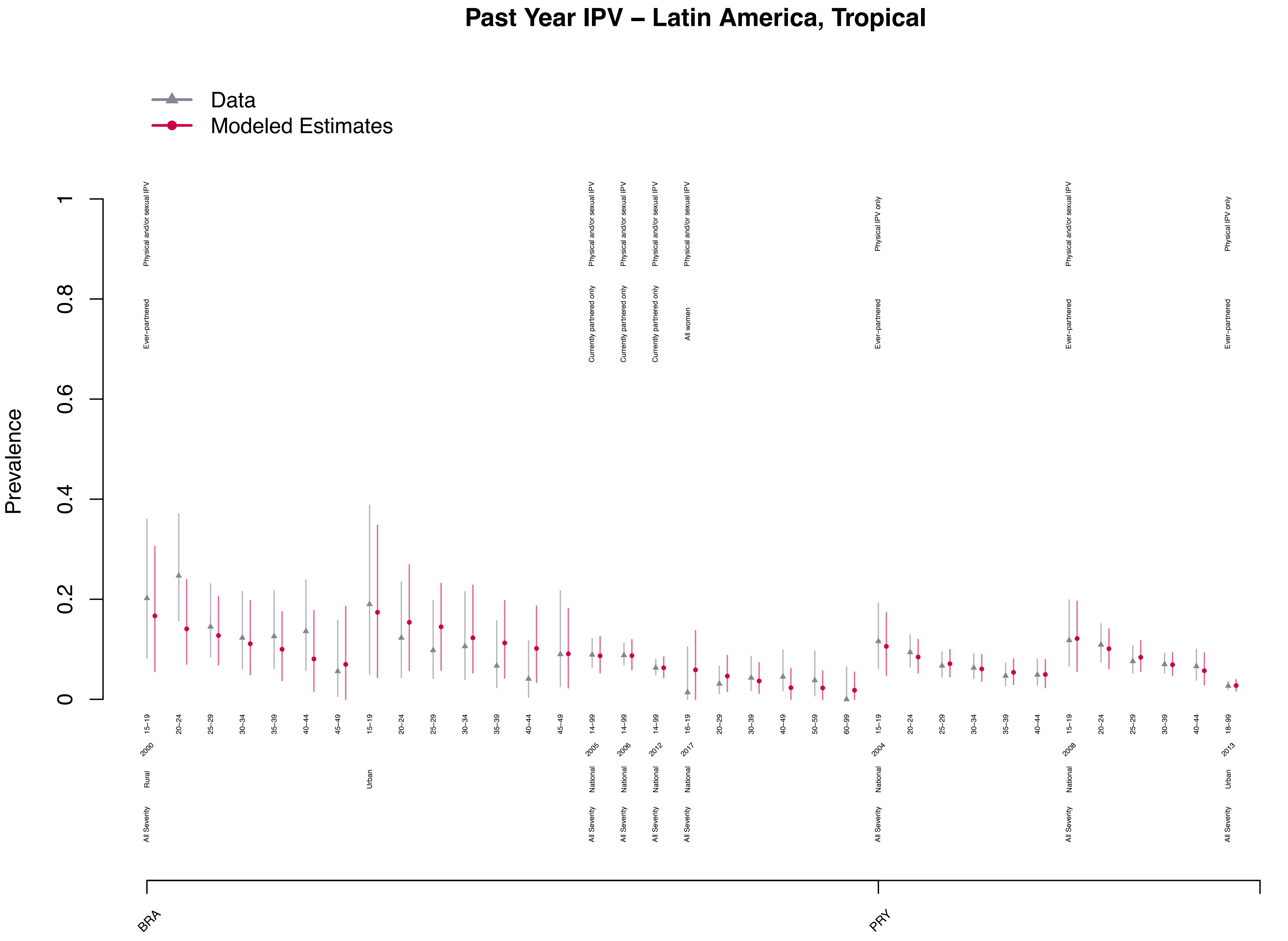

Past Year IPV – Latin America, Tropical
Data
Modeled Estimates
Prevalence
0
0.2
0.4
0.6
0.8
1
BRA
PRY

Past Year IPV – North Africa/Middle East

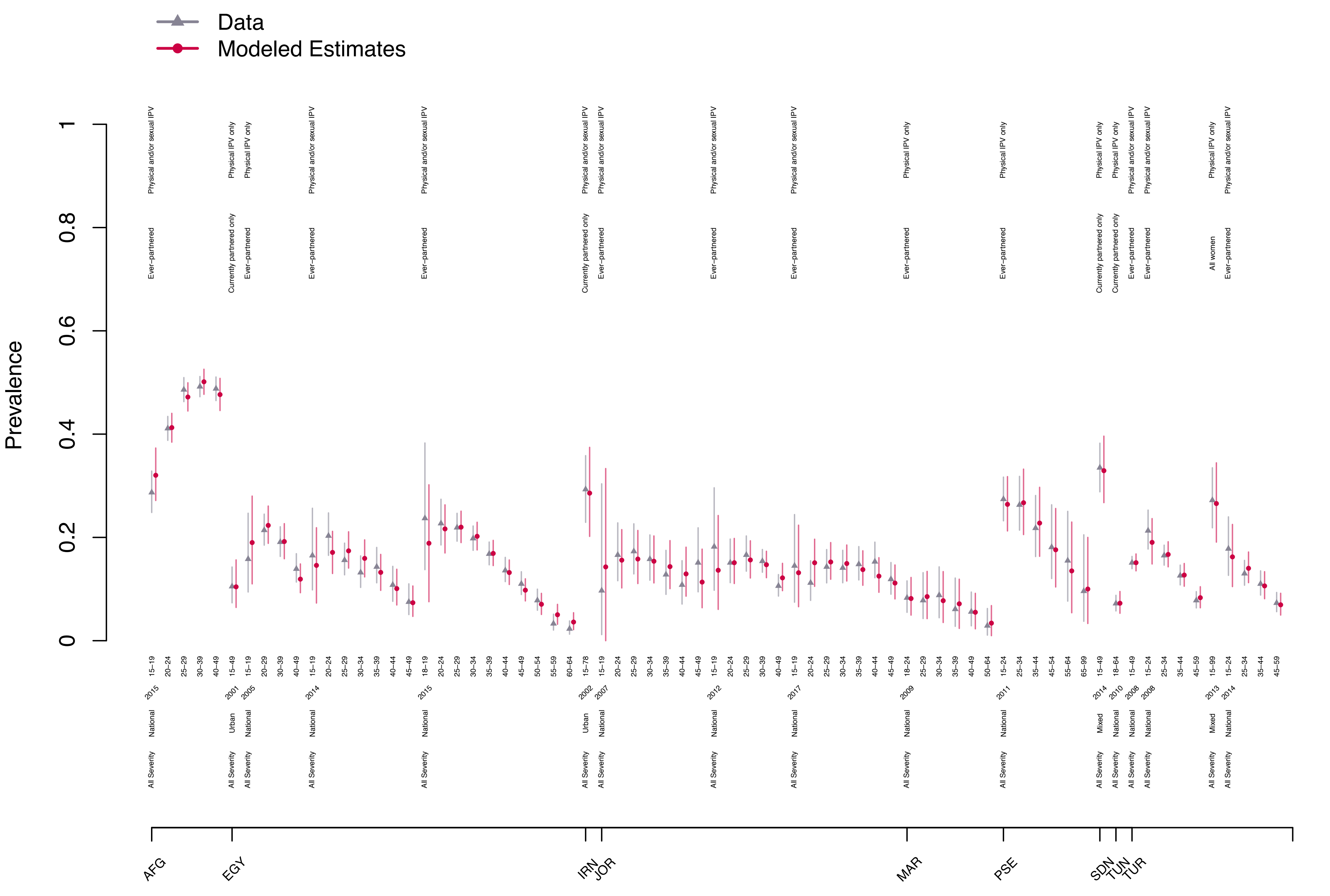

Past Year IPV – North America, High Income

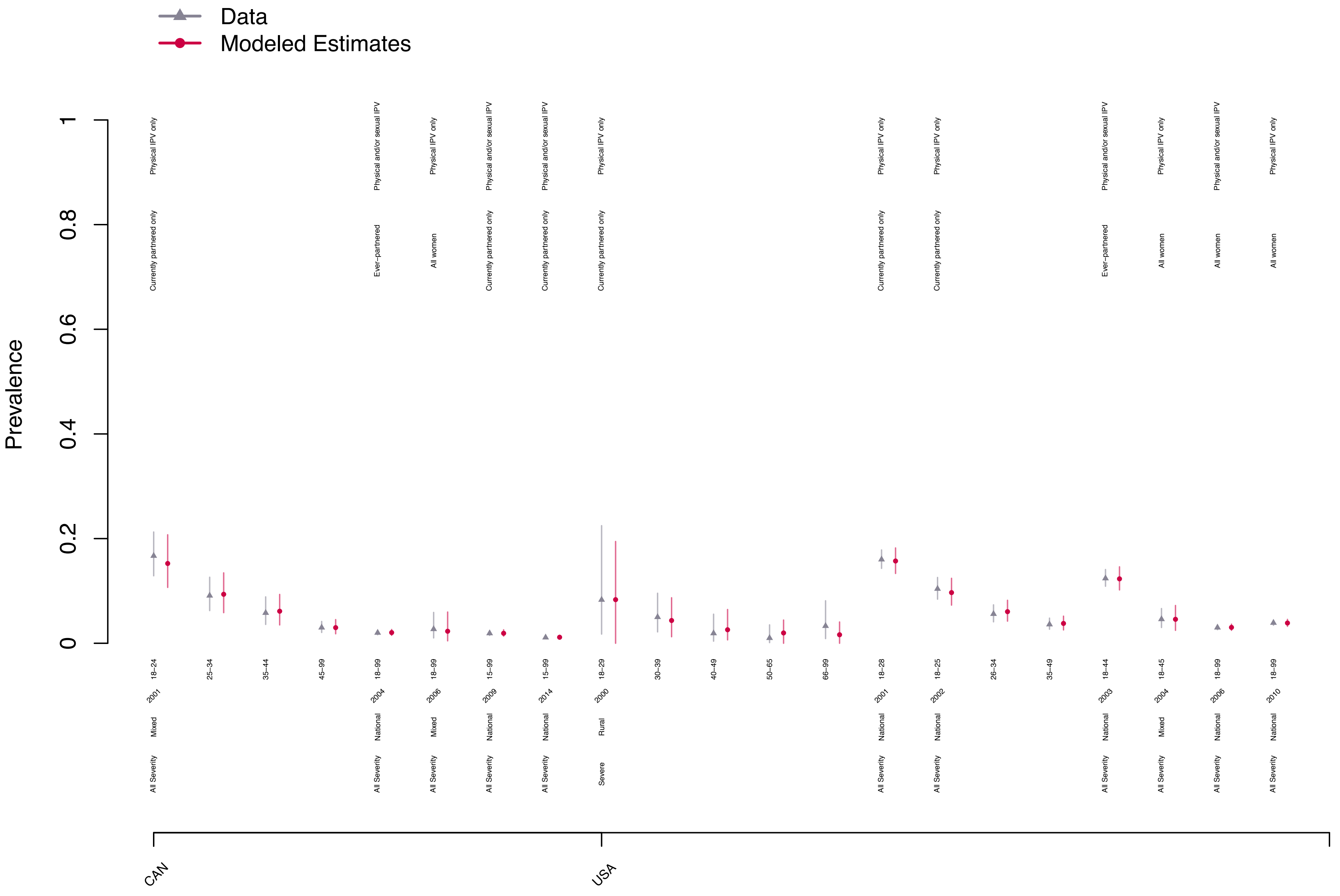

Past Year IPV – Oceania

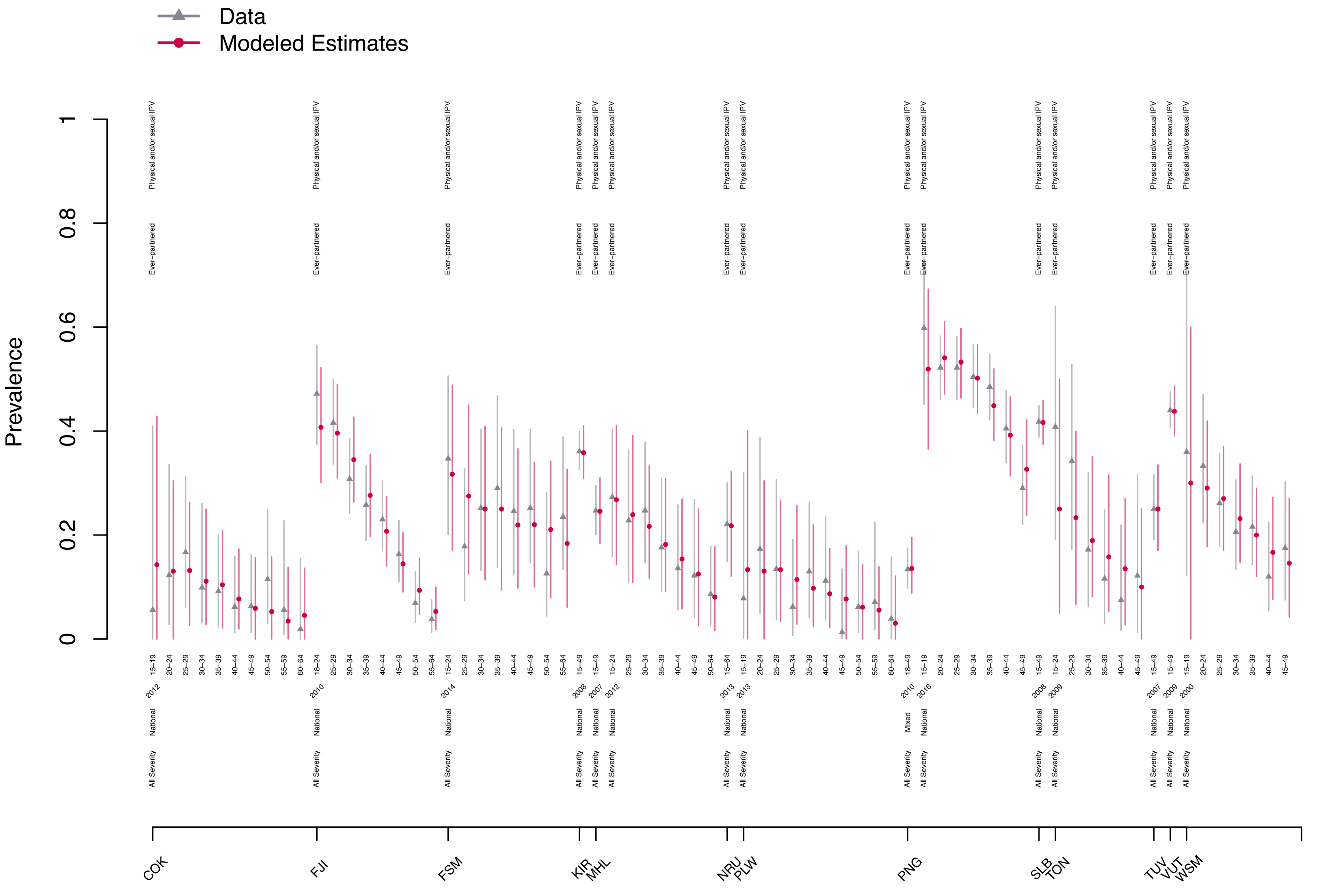

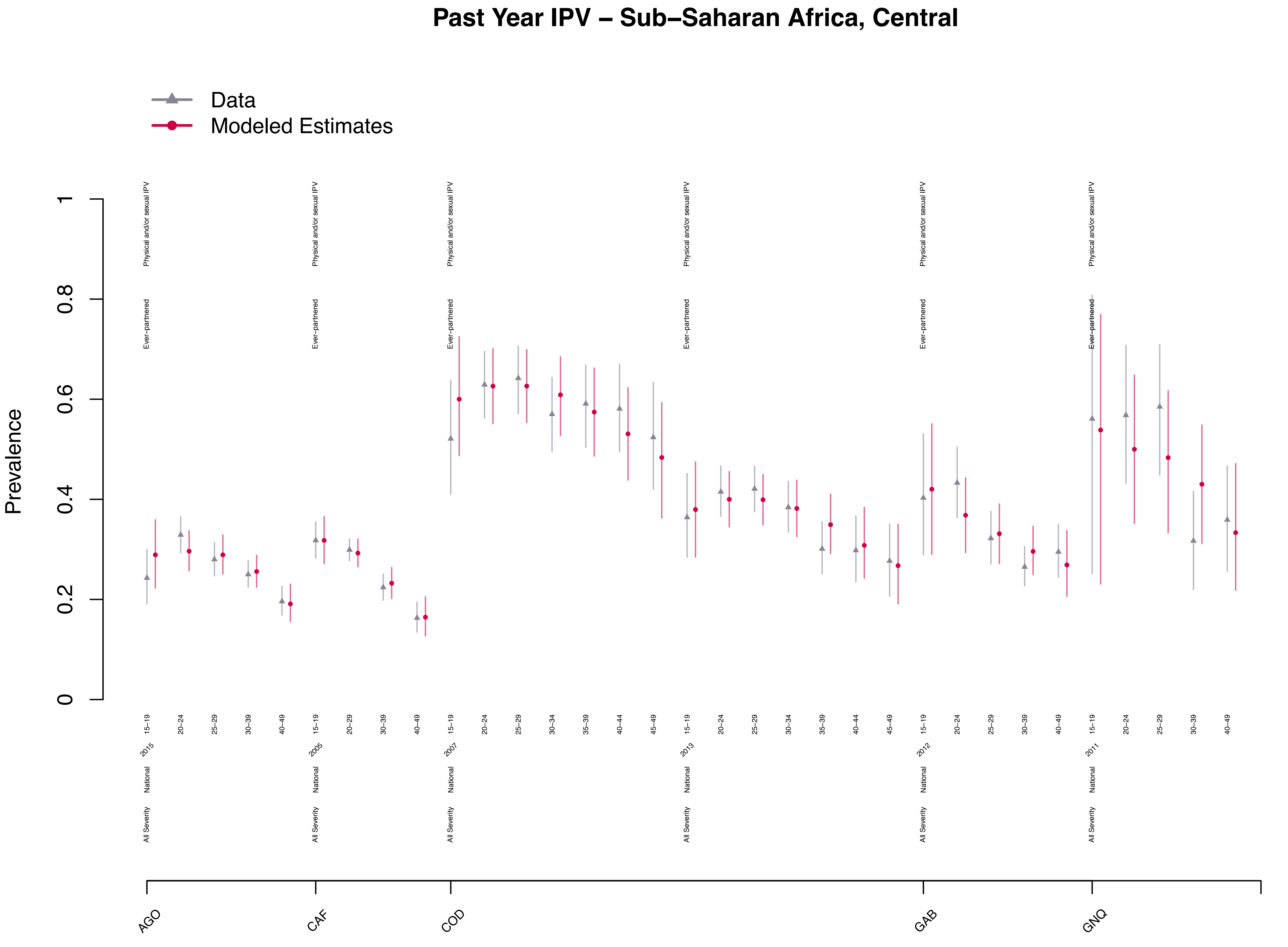

Past Year IPV – Sub–Saharan Africa, Central
Data
Modeled Estimates
Prevalence
0
0.2
0.4
0.6
0.8
1
Physical and/or sexual IPV
Ever–partnered
15–19
20–24
25–29
30–34
35–39
40–44
45–49
30–39
40–49
20–29
2015
2005
2007
2013
2012
2011
National
All Severity
AGO
CAF
COD
GAB
GNQ

Past Year IPV – Sub–Saharan Africa, East

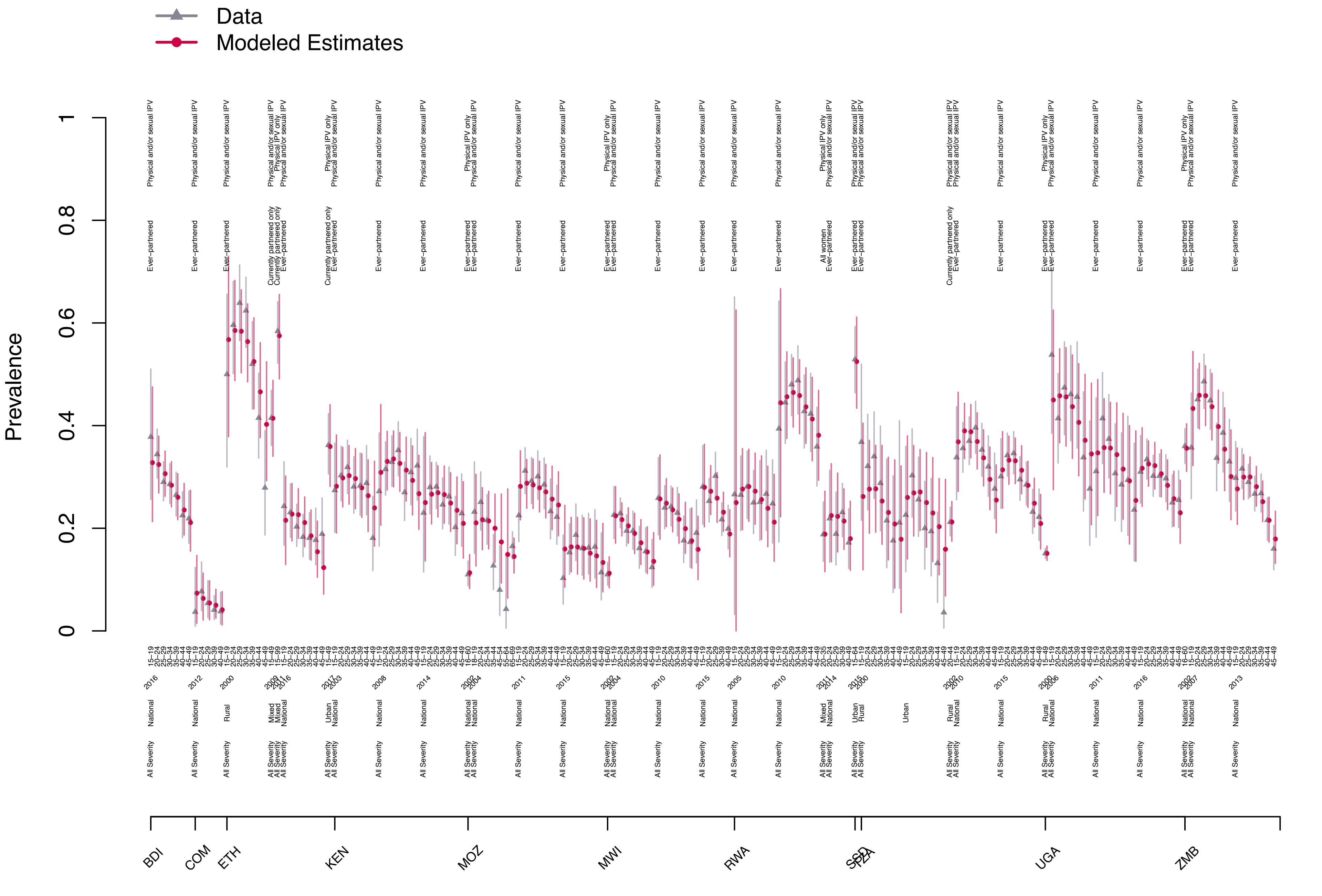

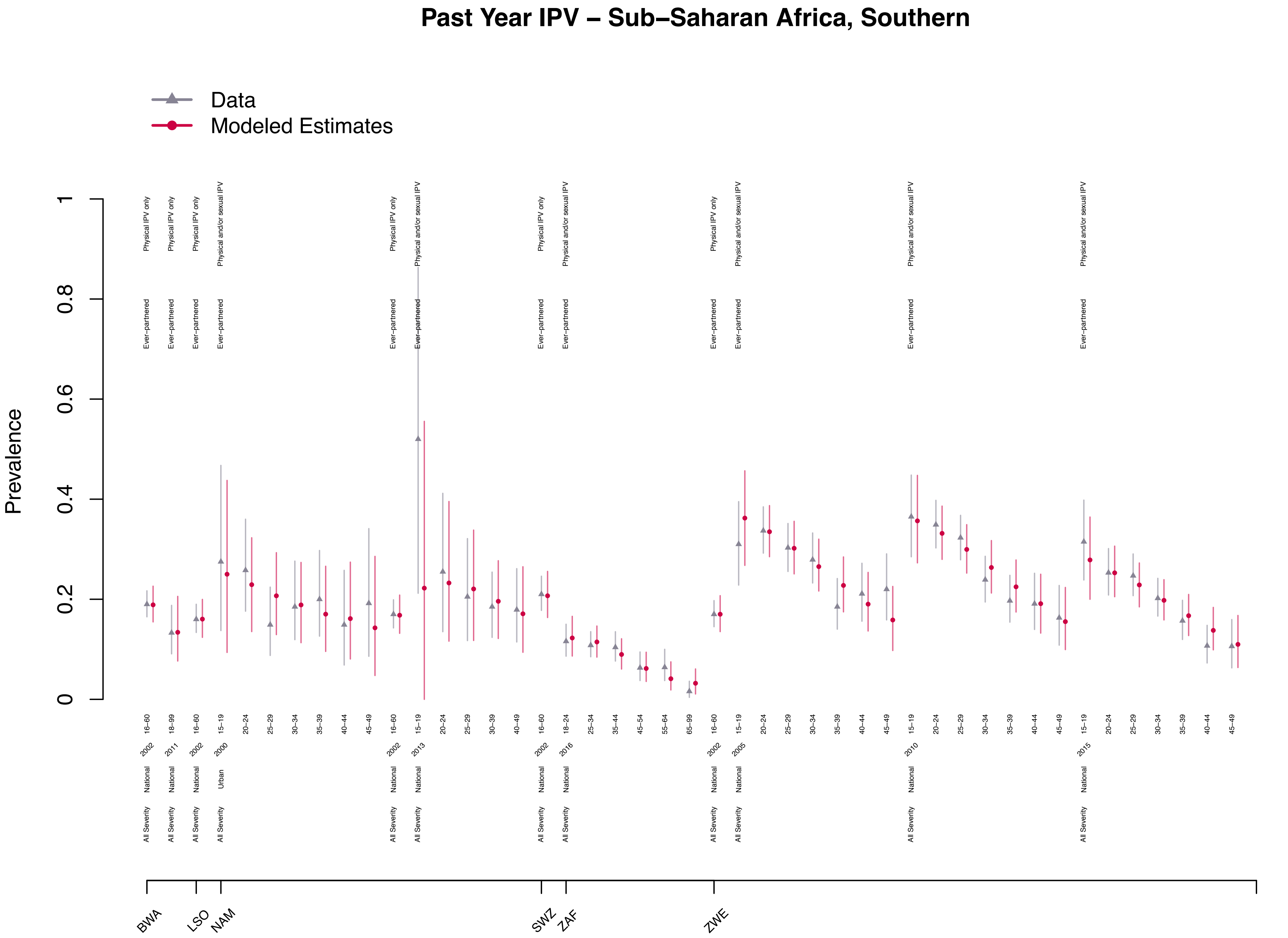

Past Year IPV – Sub–Saharan Africa, Southern
Data
Modeled Estimates
Prevalence
0
0.2
0.4
0.6
0.8
1
BWA
LSO
NAM
SWZ
ZAF
ZWE

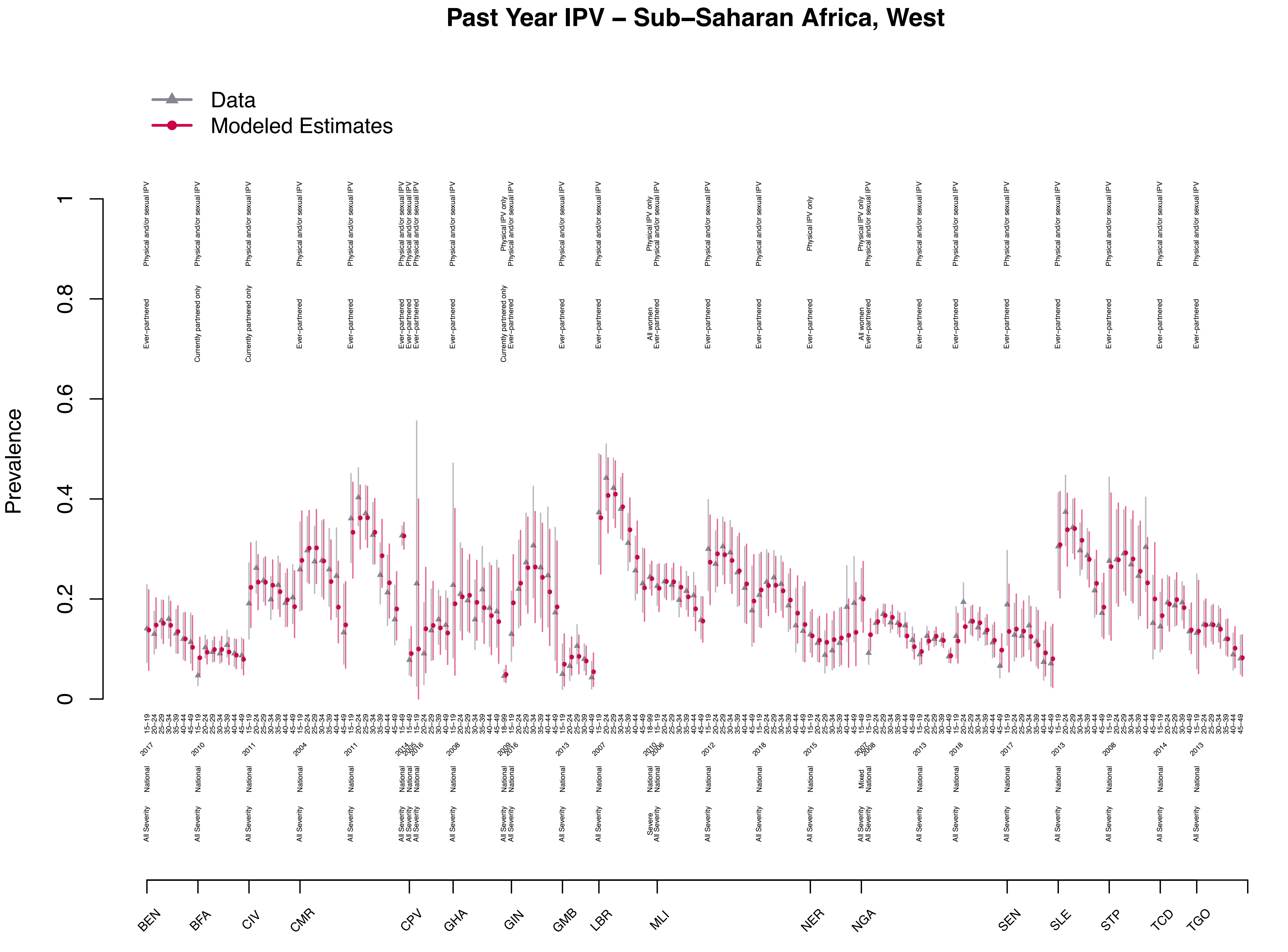

Past Year IPV – Sub–Saharan Africa, West
Data
Modeled Estimates
Prevalence
BEN
BFA
CIV
CMR
CPV
GHA
GIN
GMB
LBR
MLI
NER
NGA
SEN
SLE
STP
TCD
TGO

References

(2001). Ecuador Reproductive Health Survey 1999 (Ecuador Encuesta Demografica y de Salud Materna e Infantil 1999 (ENDEMAIN)). S. Ecuador Center for Population, D. Social, C. Division of Reproductive Health-Centers for Disease and Prevention.

Aarhus University, A. S. R. I., Alcohol Research Group, Public Health Institute, Centre for Addiction and Mental Health (Canada), Centre for Alcohol Policy Research, Turning Point Alcohol and Drug Centre (Australia), Kettil Bruun Society for Social and Epidemiological Research on Alcohol and University of North Dakota (2003). Argentina - Buenos Aires Gender, Alcohol and Culture: An International Study (GENACIS).

Achécar, M. M., N. Ramírez, J. J. Polanco, L. H. Ochoa and B. Lerebours (2003). República Dominicana Encuesta Demográfica y de Salud 2002. Santo Domingo, República Dominicana, Centro de Estudios Sociales y Demográficos - CESDEM/República Dominicana and ORC Macro.

Adegbite, O. and A. Ajuwon (2015). Intimate Partner Violence among Women of Child Bearing Age in Alimosho LGA of Lagos State, Nigeria. *African Journal of Biomedical Research* **18**: 135-146.

Aekplakorn, W. and R. Kongsakon (2007). Intimate partner violence among women in slum communities in Bangkok, Thailand. *Singapore Med J* **48**(8): 763-768.

Agence Nationale de la Statistique et de la Démographie, A. S. and Icf (2018). Senegal: Enquête Démoggraphique et de Santé Continue (EDS-Continue) 2017. Dakar, Sénégal, ANSD and ICF.

Ajdukovic, M., J. Ogresta and S. Rusac (2009). Family Violence and Health Among Elderly in Croatia. *Journal of Aggression, Maltreatment & Trauma* **18**(3): 261-279.

Ali, A. A., K. Yassin and R. Omer (2014). Domestic violence against women in Eastern Sudan. *BMC public health* **14**: 1136-1136.

Ally, E. Z., R. Laranjeira, M. C. Viana, I. Pinsky, R. Caetano, S. Mitsuhiro and C. S. Madruga (2016). Intimate partner violence trends in Brazil: data from two waves of the Brazilian National Alcohol and Drugs Survey. *Brazilian Journal of Psychiatry* **38**: 98-105.

Alvarez, J., J. Pavao, K. Mack, J. Chow, N. Baumrind and R. Kimerling (2009). Lifetime Interpersonal Violence and Self-Reported Chlamydia trachomatis Diagnosis among California Women. *Journal of women's health (2002)* **18**: 57-63.

Amowitz, L. L., G. Kim, C. Reis, J. L. Asher and V. Iacopino (2004). Human Rights Abuses and Concerns About Women's Health and Human Rights in Southern Iraq. *JAMA* **291**(12): 1471-1479.

Andarge, E. and Y. Shiferaw (2018). Disparities in Intimate Partner Violence among Currently Married Women from Food Secure and Insecure Urban Households in South Ethiopia: A Community Based Comparative Cross-Sectional Study. *BioMed Research International* **2018**: 4738527.

Andersson, N., A. Ho-Foster, S. Mitchell, E. Scheepers and S. Goldstein (2007). Risk factors for domestic physical violence: national cross-sectional household surveys in eight southern African countries. *BMC Women's Health* **7**(1): 11.

Andersson, T., G. Heimer and S. Lucas (2014). Violence and Health in Sweden: A National Prevalence Study on Exposure to Violence among Women and Men and its Association to Health, National Centre for Knowledge on Men’s Violence Against Women - NCK.

Asociación Demográfica Salvadoreña - ADS/El Salvador and Division of Reproductive Health-Centers for Disease Control and Prevention (CDC) (2004). El Salvador Reproductive Health Survey 2002-2003. San Salvador, El Salvador, ADS.

Asociación Demográfica Salvadoreña - ADS/El Salvador and Division of Reproductive Health-Centers for Disease Control and Prevention (CDC) (2009). El Salvador Reproductive Health Survey 2008. San Salvador, El Salvador, ADS.

Australian Bureau of Statistics (2013). Personal safety survey, Australia, 2012. Canberra, Australian Bureau of Statistics.

Australian Bureau of Statistics (2017). Personal Safety Survey, Australia, 2016. Canberra, Australian Bureau of Statistics.
Baker, C. K., F. H. Norris, D. M. V. Diaz, J. L. Perilla, A. D. Murphy and E. G. Hill (2005). Violence and PTSD in Mexico. *Social Psychiatry and Psychiatric Epidemiology* **40**(7): 519-528.
Balogun, M. O., E. T. Owoaje and O. I. Fawole (2012). Intimate Partner Violence in Southwestern Nigeria: Are There Rural-Urban Differences? *Women & Health* **52**(7): 627-645.
Bangladesh Bureau of Statistics (2013). Violence Against Women Survey 2011. Dhaka, Bangladesh, Bangladesh Bureau of Statistics.
Bangladesh Bureau of Statistics Statistics and Informatics Division - Ministry of Planning/Government of the People's Republic of Bangladesh (2016). Report on Violence Against Women Survey 2015. Dhaka, Bangladesh, Bangladesh Bureau of Statistics.
Barros, É. N. d., M. A. Silva, G. H. Falbo Neto, S. G. Lucena, L. Ponzo and A. P. Pimentel (2016). Prevalência e fatores associados à violência por parceiro íntimo em mulheres de uma comunidade em Recife/Pernambuco, Brasil [Prevalence and factors associated with intimate partner violence among women in Recife/Pernambuco, Brazil]. *Ciência & Saúde Coletiva* **21**: 591-598.
Basile, K. C., M. J. Breiding and S. G. Smith (2016). Disability and Risk of Recent Sexual Violence in the United States. *American journal of public health* **106**(5): 928-933.
Bergmark, K., P. W. Avall-Lundqvist E Fau - Dickman, G. Dickman Pw Fau - Steineck, L. Steineck G Fau - Henningsohn and L. Henningsohn Synergy between sexual abuse and cervical cancer in causing sexual dysfunction. (0092-623X (Print)).
Bott, S., A. Guedes, A. Ruiz-Celis and J. Mendoza (2019). Intimate partner violence in the Americas: a systematic review and reanalysis of national prevalence estimates. *Pan American health* **43**.
Bouchard, E. M., M. Tourigny, J. Joly, M. Hébert and M. Cyr (2008). Les conséquences à long terme de la violence sexuelle, physique et psychologique vécue pendant l'enfance. *Revue D Epidemiologie Et De Sante Publique - REV EPIDEMIOL SANTE PUBL* **56**: 333-344.
Bouhours, B. and R. Broadhurst (2015). Violence Against Women in Hong Kong: Results of the International Violence Against Women Survey. *Violence Against Women* **21**(11): 1311-1329.
Bouhours, B., W. C. Chan, B. Bong and S. Anderson (2013). International Violence Against Women Survey: Final Report on Singapore. *SSRN Electronic Journal*.
Boyda, D., D. McFeeters and M. Shevlin (2015). Intimate partner violence, sexual abuse, and the mediating role of loneliness on psychosis. *Psychosis* **7**(1): 1-13.
Brown, L., T. Thurman, J. Bloem and C. Kendall (2006). Sexual Violence in Lesotho. *Studies in Family Planning* **37**(4): 269-280.
Burazeri, G., E. Roshi, R. Jewkes, S. Jordan, V. Bjegovic and U. Laaser (2005). Factors associated with spousal physical violence in Albania: cross sectional study. *Bmj* **331**(7510): 197-201.
Burczycka, M. and S. Conroy (2016). Family violence in Canada: A statistical profile, 2016, Statistics Canada - Juristats.
Buriánek, J., S. Pikálková, Z. Podaná and V. Kolínská (2016). *Abused, Battered, or Stalked: Violence in Intimate Partner Relations Gendered*, Charles University.
Canadian Centre for Justice Statistics - Statistics Canada (2006). Family Violence in Canada: A Statistical Profile2006, Statistics Canada**:** 78.
Cayemittes, M., M. F. Busangu, J. d. D. Bizimana, B. Barrère and B. Sévère (2013). Haïti Enquête Mortalité, Morbidité et Utilisation des Services 2012. Calverton, Maryland, USA, Ministère de la Santé Publique et de la Population - MSPP/Haïti, l'Institut Haïtien de l'Enfance - IHE and ICF International.

Cayemittes, M., M. F. Placide, B. Barrère, S. Mariko and B. Sévère (2001). Haïti Enquête Mortalité, Morbidité et Utilisation des Services 2000. Calverton, Maryland, USA, Ministère de la Santé Publique et de la Population/Haïti, Institut Haïtien de l'Enfance and ORC Macro.
Cayemittes, M., M. F. Placide, S. Mariko, B. Barrère and B. Sévère (2007). Haiti Enquête Mortalité, Morbidité et Utilisation des Services 2005-2006. Calverton, Maryland, USA, Institut Haïtien de l'Enfance and Macro International.
CDV/Malta, C. o. D. V.-. (2011). A nationwide research study on the prevalence of domestic violence against women in Malta and its impact on their employment prospects.
Cellule de Planification et de Statistique - C. P. S. Ssdspf Mali, Institut National de la Statistique - Instat Mali, C. d. É. e. d. I. S.-I.-S. Mali and I. C. F. International (2014). Mali Enquête Démographique et de Santé (EDSM V) 2012-2013. Rockville, Maryland, USA, CPS, INSTAT, INFO-STAT and ICF International.
Centers for Disease Control and Prevention - CDC and ORC Macro (2003). Reproductive, Maternal and Child Health in Eastern Europe and Eurasia: A Comparative Report. Atlanta (GA) Calverton (MD).
Central Bureau of Statistics - CBS/Kenya, Ministry of Health - MOH/Kenya and O. R. C. Macro (2004). Kenya Demographic and Health Survey 2003. Calverton, Maryland, USA, CBS, MOH, and ORC Macro.
Central Statistical Agency - CSA/Ethiopia and ICF (2017). Ethiopia Demographic and Health Survey 2016. Addis Ababa, Ethiopia, CSA and ICF.
Central Statistical Office - CSO/Zambia, Ministry of Health - MOH/Zambia, Tropical Disease Research Centre - TDRC/Zambia and University of Zambia (2009). Zambia Demographic and Health Survey 2007. Calverton, Maryland, USA, Central Statistical Office/Zambia and Macro International.
Central Statistical Office - CSO/Zambia, Ministry of Health - MOH/Zambia, University of Zambia Teaching Hospital Virology Laboratory, University of Zambia Department of Population Studies, Tropical Diseases Research Centre Zambia and I. International (2015). Zambia Demographic and Health Survey 2013-14. Rockville, Maryland, USA, Central Statistical Office/Zambia, Ministry of Health/Zambia, and ICF International.
Central Statistical Office - CSO/Zimbabwe and Macro International (2007). Zimbabwe Demographic and Health Survey 2005-06. Calverton, Maryland, USA, Central Statistical Office/Zimbabwe and Macro International.
Central Statistical Office -CSO/Zambia, Central Board of Health - CBOH/Zambia and ORC Macro (2003). Zambia Demographic and Health Survey 2001-2002. Calverton, Maryland, USA, Central Statistical Office/Zambia, Central Board of Health/Zambia, and ORC Macro.
Central Statistics Organization/Afghanistan, Ministry of Public Health/Afghanistan and ICF (2017). Afghanistan Demographic and Health Survey 2015. Kabul, Afghanistan, Central Statistics Organization.
Centre for Disease Control and Prevention - CDC and ORC Macro (2003). Georgia Reproductive Health Survey 1999-2000.
Centro de Estudios de la Mujer and Centro de Estudios de Población y Desarrollo (2018). Encuesta Nacional sobre Igualdad de Género (ENIG-2016).
Centro de Estudios de Población - CEPEP/Paraguay (2005). Encuesta Nacional de Demografía y Salud • Sexual y Reproductiva 2004, ENDSSR 2004: informe final, CEPEP, USAID, UNICEF, CDC.
Centro de Estudios de Población y Desarrollo Social (2005). *Endemain 2004 : informe final : encuesta demográfica y de salud materna e infantil*. Quito, CEPAR.

Centro de Estudios Sociales y Demográficos - CESDEM/República Dominicana and ICF International (2014). República Dominicana Encuesta Demográfica y de Salud 2013. Santo Domingo, República Dominicana, CESDEM/República Dominicana and ICF International.
Centro de Estudios Sociales y Demográficos - CESDEM/República Dominicana and Macro International (2008). República Dominicana Encuesta Demográfica y de Salud 2007. Santo Domingo, República Dominicana, CESDEM/República Dominicana and Macro International.
Centro Paraguayo de Estudios de Población - CEPEP/Paraguay (2009). Encuesta Nacional de Demografía y Salud • Sexual y Reproductiva 2008, ENDSSR 2008: informe final, CEPEP, USAID, UNICEF, CDC.
CEPAR - Centro de Estudios de Población y Desarrollo Social - Ecuador (2005). Encuesta demográfica y de salud materna e infantil "ENDEMAIN". *CEPAR - Centro de Estudios de Población y Desarrollo Social*. Quito, CEPAR - Centro de Estudios de Población y Desarrollo Social**:** 146.
Chacham, A. S., A. B. S. Simão and A. J. Caetano (2016). Gender-based violence and sexual and reproductive health among low-income youth in three Brazilian cities. *Reproductive Health Matters* **24**(47): 141-152.
Chiu, G. R., K. E. Lutfey, H. J. Litman, C. L. Link, S. A. Hall and J. B. McKinlay (2013). Prevalence and overlap of childhood and adult physical, sexual, and emotional abuse: a descriptive analysis of results from the Boston Area Community Health (BACH) survey. *Violence and victims* **28**(3): 381-402.
Chuang, C. H., A. L. Cattoi, J. S. McCall-Hosenfeld, F. Camacho, A.-M. Dyer and C. S. Weisman (2012). Longitudinal association of intimate partner violence and depressive symptoms. *Mental health in family medicine* **9**(2): 107-114.
Coa, R. and L. H. Ochoa (2009). Bolivia Encuesta Nacional de Demografía y Salud 2008. Calverton, Maryland, USA, Ministerio de Salud y Deportes and Macro International.
Coker, A. L., C. P. Hopenhayn C Fau - DeSimone, H. M. DeSimone Cp Fau - Bush, L. Bush Hm Fau - Crofford and L. Crofford Violence against Women Raises Risk of Cervical Cancer. (1931-843X (Electronic)).
Consejo Nacional para la Igualdad de Género - CNIG/Ecuador (2014). La Violencia de Género contra las mujeres en el Ecuador: Análisis de los resultados sobre la Encuesta Nacional sobre Relaciones Familiares y Violencia de Género
Contreras-Urbina, M., A. Bourassa, R. Myers, J. Ovince, R. Rodney and S. Bobbili (2019). *Guyana Women's Health and Life Experiences Survey Report*.
Dalal, K., F. Rahman and B. Jansson (2009). Wife abuse in rural Bangladesh. *Journal of biosocial science* **41**: 561-573.
De León Richardson, R. and e. al. (2011). Encuesta Nacional de Salud Sexual y Reproductiva (ENASSER) Panama 2009: Informe Final. Panama, Instituto Conmemorativo Gorgas de Estudios de la Salud.
De León Richardson, R. and e. al. (2018). Encuesta nacional de salud sexual y reproductiva (ENASSER) Panama 2014-2015. Panama, Instituto Conmemorativo Gorgas de Estudios de la Salud,.
Department of Health and Social Affairs - DHSA/Micronesia (2014). Federated States of Micronesia Family Health and Safety Study: A prevalence study on violence against women.
Department of Statistics Jordan and ICF (2019). Jordan Population and Family Health Survey 2017-18. Amman, Department of Statistics/Jordan and ICF.
Department of Statistics Jordan and ICF International (2013). Jordan Population and Family Health Survey 2012. Calverton, Maryland, USA, Department of Statistics/Jordan and ICF International.

Department of Statistics Jordan and Macro International (2008). Jordan Population and Family Health Survey 2007. Calverton, Maryland, USA, Department of Statistics/Jordan and Macro International.
Deribe, K., B. K. Beyene, A. Tolla, P. Memiah, S. Biadgilign and A. Amberbir (2012). Magnitude and Correlates of Intimate Partner Violence against Women and Its Outcome in Southwest Ethiopia. *PLOS ONE* **7**(4): e36189.
Diop Sall, F., D. Saley, A. Modieli and UNFPA (2015). Ampleur et Déterminants des Violences Basées sur le Genre au Niger - Rapport Final.
Dirección General de Estadística y Censos (DIGESTYC) (2018). Encuesta Nacional de Violencia Contra la Mujer - El Salvador 2017, Dirección General de Estadística y Censos (DIGESTYC).
Direction Générale de la Statistique - DGS/Gabon and ICF International (2013). Gabon Enquête Démographique et de Santé 2012. Calverton, Maryland, USA, Direction Générale de la Statistique - DGS/Gabon and ICF International.
Direction Générale de la Statistique et de la Prospective - DGSP/Comores and ICF International (2014). Comores Enquête Démographique et de Santé et à Indicateurs Multiples (EDSC-MICS II) 2012. Rockville, Maryland, USA, DGSP and ICF International.
Dougé, N., E. B. Lehman and J. S. McCall-Hosenfeld (2014). Social support and employment status modify the effect of intimate partner violence on depression symptom severity in women: results from the 2006 Behavioral Risk Factor Surveillance System Survey. *Women's health issues : official publication of the Jacobs Institute of Women's Health* **24**(4): e425-e434.
Dunn, E. C., S. E. Gilman, J. B. Willett, N. B. Slopen and B. E. Molnar (2012). THE IMPACT OF EXPOSURE TO INTERPERSONAL VIOLENCE ON GENDER DIFFERENCES IN ADOLESCENT-ONSET MAJOR DEPRESSION: RESULTS FROM THE NATIONAL COMORBIDITY SURVEY REPLICATION (NCS-R). *Depression and Anxiety* **29**(5): 392-399.
Duvvury, N., M. Ozonas Marcos, M. Gadallah, S. Attia, N. El Adly, W. Maged and G. Haddad (2015). The Egypt Economic Cost of Gender-Based Violence Survey (ECGBVS) 2015, UNFPA, Central Agency for Public Mobilization and Statistics (CAPMAS),
National Council for Women.
Economic Policy Planning and Statistics Office - EPPSO/Marshall Islands, SPC and Macro International (2008). Republic of the Marshall Islands Demographic and Health Survey 2007. Majuro, Marshall Islands.
Eisikovits, Z., Z. Winstok and G. Fishman (2004). The First Israeli National Survey on Domestic Violence. *Violence Against Women* **10**(7): 729-748.
El-Zanaty, F. and A. Way (2006). Egypt Demographic and Health Survey 2005. Cairo, Egypt, Ministry of Health and Population/Egypt, National Population Council/Egypt, El-Zanaty and Associates/Egypt, and ORC Macro.
Elliott, D. M., D. S. Mok and J. Briere (2004). Adult Sexual Assault: Prevalence, Symptomatology, and Sex Differences in the General Population. *Journal of Traumatic Stress* **17**(3): 203-211.
Ellsberg, M., M. Contreras, M. Murphy and A. Blackwell (2017). *No Safe Place: A Lifetime of Violence for Conflict-affected Women and Girls in South Sudan*.
European Union Agency for Fundamental Rights (2014). Violence against women: an EU-wide survey. Vienna, European Union Agency for Fundamental Rights,**:** 200.
Fan, A. Z., J. Liu, H. Kress, S. Gupta, M. Shawa, N. Wadonda-Kabondo and J. Mercy (2017). Applying Structural Equation Modeling to Measure Violence Exposure and Its Impact on Mental Health: Malawi Violence Against Children and Young Women Survey, 2013. *Journal of Interpersonal Violence*: 0886260517741214.

FANSLOW, J. AND E. ROBINSON (2004). Violence against women in New Zealand: prevalence and health consequences. *N Z Med J* **117**(1206): U1173.
FIJI WOMEN'S CRISIS CENTRE (FWCC) (2013). National Research on Women's Health and Life Experiences in Fiji (2010/2011): A summary exploring the prevalence, incidence and attitudes to intimate partner violence in Fiji. Fiji, Fiji Women's Crisis Centre (FWCC)**:** 24.
FULU, E., R. JEWKES, T. ROSELLI AND C. GARCIA-MORENO (2013). Prevalence of and factors associated with male perpetration of intimate partner violence: findings from the UN Multi-country Cross-sectional Study on Men and Violence in Asia and the Pacific. *Lancet Glob Health* **1**(4): e187-207.
GARCIA-MORENO, C., H. A. F. M. JANSEN, M. ELLSBERG, L. HELSE AND C. WATTS (2005). *WHO multi-country study on women's health and domestic violence against women: initial results on prevalence, health outcomes and women's responses*. Geneva, WHO.
GENDER EQUALITY AGENCY OF BOSNIA AND HERZEGOVINA AND MINISTRY FOR HUMAN RIGHTS AND REFUGEES OF BIH (2013). Prevalence and Characteristics of Violence Against Women in Bosnia and Herzegovina, Gender Equality Agency of Bosnia and Herzegovina,
UNFPA**:** 62.
GENERAL DIRECTORATE OF STATISTICS, MINISTRY OF FINANCE/TIMOR LESTE AND ICF (2018). Timor-Leste Demographic and Health Survey 2016. Dili, Timor-Leste, GDS and ICF.
GENERAL STATISTICAL OFFICE - GSO/VIET NAM (2010). National Study on Domestic Violence against Women in Viet Nam 2010: Full Report**:** 210.
GFK (2013). Principales Resultados “Encuesta Nacional de Victimización por Violencia Intrafamiliar y Delitos Sexuales”, Ministerio del Interior y Seguridad Pública.
GFK UKRAINE (2014). The Prevalence of Violence Against Women and Girls.
GHANA STATISTICAL SERVICE - GSS/GHANA, GHANA HEALTH SERVICE - GHS/GHANA AND ICF MACRO (2009). Ghana Demographic and Health Survey 2008. Accra, Ghana, GSS, GHS, and ICF Macro.
GHAZIZADEH, A. Domestic violence: a cross-sectional study in an Iranian city. (1020-3397 (Print)).
GÍSLASON, I. V. (2008). Ofbeldi í nánum samböndum: orsakir, afleiðingar, úrræði [Violence in intimate relationships: causes, consequences, remedies]. Reykjavík, Iceland, Félagsmálaráðuneytið (Ministry of Social Affairs – now Ministry for Welfare)
GRUSZCZYŃSKA, B. (2007). Survey on violence against women in Poland. Key Findings.
GUCLU, Y. A. AND H. CAN (2018). Ethnic background and alcohol use of the spouse emerge as major risk factors for domestic violence: An observational study from Turkey. *JPMA. The Journal of the Pakistan Medical Association* **68**(12): 1782-1786.
GUO, S. F., J. L. WU, C. Y. QU AND R. Y. YAN (2004). Domestic abuse on women in China before, during, and after pregnancy. *Chin Med J (Engl)* **117**(3): 331-336.
HAARR, R. N. (2013). Domestic Violence in Albania: 2013 National Population-Based Survey. *United Nations Development Program (UNDP).* Tirana, Albania.**:** 80.
HABIB, S. R., E. K. ABDEL AZIM, I. A. FAWZY, N. N. KAMAL AND A. M. EL SHERBINI (2011). Prevalence and Effects of Violence Against Women in a Rural Community in Minia Governorate, Egypt*. *Journal of Forensic Sciences* **56**(6): 1521-1527.
HACETTEPE UNIVERSITY INSTITUTE OF POPULATION STUDIES, MINISTY OF HEALTH GENERAL DIRECTORATE OF MOTHER - MOHGDM/TURKEY, CHILD HEALTH/FAMILY PLANNING - CHFP/TURKEY, TR PRIME MINISTRY STATE PLANNING ORGANIZATION AND ICF MACRO (2009). Turkey Demographic and Health Survey 2008. Ankara, Turkey, Hacettepe Univeristy Institute of Population Studies/Turkey, Ministry of Health General Directorate.
HACETTEPE UNIVERSITY INSTITUTE OF POPULATION STUDIES, T.R. MINISTRY OF DEVELOPMENT, T.R. MINISTRY OF HEALTH AND ICF (2014). Turkey Demographic and Health Survey 2013. Ankara, Turkey, Hacettepe University Institute of Population Studies, Ministry of Health, Ministry of Development.

HASEGAWA, M., Y. BESSHO, T. HOSOYA AND Y. DEGUCHI (2005). Prevalence of intimate partner violence and related factors in a local city in Japan. *[Nihon kōshū eisei zasshi] Japanese journal of public health* **52**: 411-421.
HASSAN, F., L. S. SADOWSKI, S. I. BANGDIWALA, B. VIZCARRA, L. RAMIRO, C. S. DE PAULA, I. A. BORDIN AND M. K. MITRA (2004). Physical intimate partner violence in Chile, Egypt, India and the Philippines. *Inj Control Saf Promot* **11**(2): 111-116.
HAUT-COMMISSARIAT AU PLAN - HCP/MAROC (2009). Enquête nationale sur la prévalence de la violence à l'égard des femmes au Maroc 414.
HENSTRIDGE, J. (2007). Analysis of the 2005 Personal Safety Survey Nedland, WA, Data Analysis Australia.
HYNES, M., J. WARD, K. ROBERTSON AND C. CROUSE (2004). A Determination of the Prevalence of Gender-based Violence among Conflict-affected Populations in East Timor. *Disasters* **28**(3): 294-321.
IIPS/INDIA, I. I. F. P. S.-. AND ICF (2017). India National Family Health Survey NFHS-4 2015-16. Mumbai, India, IIPS and ICF.
INSTITUT HAÏTIEN DE L'ENFANCE, I. H. E. H. AND ICF (2018). Haiti Enquête Mortalité, Morbidité et Utilisation des Services 2016-2017 - EMMUS-VI. Pétion-Ville/Haïti, IHE/Haiti, ICF.
INSTITUT NATIONAL DE LA STATISTIQUE - INS/CAMEROUN (2015). Enquête par grappes à indicateurs multiples (MICS5), 2014, Rapport Final. Yaoundé, Cameroun, Institut National de la Statistique,.
INSTITUT NATIONAL DE LA STATISTIQUE - INS/CAMEROUN AND ICF INTERNATIONAL (2012). Cameroun Enquête Démographique et de Santé et à Indicateurs Multiples (EDS-MICS) 2011. Calverton, Maryland, USA, INS/Cameroun and ICF International.
INSTITUT NATIONAL DE LA STATISTIQUE - INS/CAMEROUN AND ORC MACRO (2005). Enquête Démographique et de Santé Cameroun 2004. Calverton, Maryland, USA, INS/Cameroun and ORC Macro.
INSTITUT NATIONAL DE LA STATISTIQUE - INS/CÔTE D'IVOIRE AND ICF INTERNATIONAL (2013). Côte d'Ivoire Enquête Démographique et de Santé et à Indicateurs Multiples 2011-2012. Calverton, Maryland, USA, INS/Côte d'Ivoire and ICF International.
INSTITUT NATIONAL DE LA STATISTIQUE - INSTAT/MALI, CELLULE DE PLANIFICATION ET DE STATISTIQUE SECTEUR SANTÉ-DÉVELOPPEMENT AND ICF (2019). Mali Demographic and Health Survey 2018. Bamako, Mali, INSTAT/CPS/SS-DS-PF and ICF.
INSTITUT NATIONAL DE LA STATISTIQUE DES ÉTUDES ÉCONOMIQUES ET DÉMOGRAPHIQUES - INSEED/TCHAD, MINISTÈRE DE LA SANTÉ PUBLIQUE - MSP/TCHAD AND ICF INTERNATIONAL (2016). Enquête Démographique et de Santé et à Indicateurs Multiples au Tchad (EDS-MICS) 2014-2015.
INSTITUT NATIONAL DE LA STATISTIQUE DU RWANDA - INSR AND ORC MACRO (2006). Rwanda Enquête Démographique et de Santé 2005. Calverton, Maryland, USA, INSR and ORC Macro.
INSTITUT NATIONAL DE LA STATISTIQUE ET DE L'ANALYSE ÉCONOMIQUE - INSAE/BÉNIN AND ICF (2019). République Du Bénin Ciquième Enquête Démographique et de Santé au Bénin (EDSB-V) 2017-2018. Cotonou, Bénin, INSAE/Benin and ICF.
INSTITUT NATIONAL DE LA STATISTIQUE ET DE LA DÉMOGRAPHIE - INSD/BURKINA FASO AND ICF INTERNATIONAL (2012). Burkina Faso Enquête Démographique et de Santé et à Indicateurs Multiples (EDSBF-MICS IV) 2010. Calverton, Maryland, USA, Institut National de la Statistique et de la Démographie - INSD/Burkina Faso and ICF International.
INSTITUTE FOR LABOR AND FAMILY RESEARCH - ILFR/SLOVAKIA (2010). Súhrnná správa o stave rodovej rovnosti na Slovensku za ro k 2010.
INSTITUTE OF STATISTICS - INSTAT/ALBANIA, INSTITUTE OF PUBLIC HEALTH - IPH/ALBANIA AND ICF (2018). Albania Demographic and Health Survey 2017-18. Triana, Albania, INSTAT, IPH, and ICF.

INSTITUTIO DE PESQUISA DATASENADO - IPDS/BRASIL, OBSERVATÓRIO DA MULHER CONTRA VIOLÊNCIA AND SECRETARIA DE TRANSPARÊNCIA (2017). Violência doméstica e familiar contra a mulher.

INSTITUTO CABOVERDIANO PARA A IGUALDADE E EQUIDADE DE GÉNERO - ICIEG/CABO VERDE AND INSTITUTIO NACIONAL DE ESTATÍSTICA - INE/CABO VERDE (2017). Mulheres e Homens em Cabo Verde, Factos e Números, 2017. Cidade da Praia - Santiago, Cabo Verde, ICIEG, INE: 82.

INSTITUTO NACIONAL DE ESTADÍSTICA - INE/URUGUAY (2013). Encuesta de Violencia Basade en Género y Generaciones (EVBGG) 2013.

INSTITUTO NACIONAL DE ESTADÍSTICA - INE/VENEZUELA (2013). Estadística de Violencia Doméstica y Violencia de Género: Año 2011.

INSTITUTO NACIONAL DE ESTADÍSTICA (INE)/ESTADO PLURINACIONAL DE BOLIVIA (2017). Encuesta de prevalencia y características de la violencia contra las mujeres 2016. Resultados. La Paz, Bolivia Instituto Nacional de Estadística (INE) 104.

INSTITUTO NACIONAL DE ESTADÍSTICA E INFORMÁTICA - INEI/PERÚ (2012). Perú Encuesta Demográfica y de Salud Familiar - ENDES 2011. Lima, Perú, INEI/Perú.

INSTITUTO NACIONAL DE ESTADÍSTICA E INFORMÁTICA - INEI/PERÚ (2013). Perú Encuesta Demográfica y de Salud Familiar - ENDES 2012. Lima, Perú, INEI/Perú.

INSTITUTO NACIONAL DE ESTADÍSTICA E INFORMÁTICA - INEI/PERÚ (2014). Perú Encuesta Demográfica y de Salud Familiar - ENDES 2013. Lima, Perú, INEI/Perú.

INSTITUTO NACIONAL DE ESTADÍSTICA E INFORMÁTICA - INEI/PERÚ (2015). Perú Encuesta Demográfica y de Salud Familiar - ENDES 2014. Lima, Perú, INEI/Perú.

INSTITUTO NACIONAL DE ESTADISTICA E INFORMÁTICA - INEI/PERÚ AND MACRO INTERNATIONAL (2001). Perú Encuesta Demográfica y de Salud Familiar 2000. Lima, Perú, INEI/Perú and Macro International.

INSTITUTO NACIONAL DE ESTADÍSTICA E INFORMÁTICA - INEI/PERÚ AND ORC MACRO (2007). Peru Encuesta Demográfica y de Salud Familiar - ENDES Continua 2004-2006. Lima, Perú, INEI/Perú and ORC Macro.

INSTITUTO NACIONAL DE ESTADÍSTICA E INFORMÁTICA - INEI/PERÚ AND ORC MACRO (2009). Peru Encuesta Demográfica y de Salud Familiar 2007-2008. Lima, Perú, INEI/Peru and ORC Macro.

INSTITUTO NACIONAL DE ESTADÍSTICA E INFORMÁTICA - INEI/PERÚ AND ORC MACRO (2010). Perú Encuesta Demográfica y de Salud Familiar - ENDES Continua 2009. Lima, Perú, INEI/Perú and ORC Macro.

INSTITUTO NACIONAL DE ESTADÍSTICA E INFORMÁTICA - INEI/PERÚ AND ORC MACRO (2011). Perú Encuesta Demográfica y de Salud Familiar - ENDES Continua 2010. Lima, Perú, INEI/Perú and ORC Macro.

INSTITUTO NACIONAL DE ESTADÍSTICA Y GEOGRAFÍA - INEGI/MEXICO (2004). Encuesta Nacional sobre la Dinámica de las Relaciones en los Hogares 2003. ENDIREH. Estados Unidos Mexicanos.

INSTITUTO NACIONAL DE ESTADÍSTICA Y GEOGRAFÍA - INEGI/MEXICO (2007). Panorama de violencia contra las mujeres. ENDIREH 2006. Estados Unidos Mexicanos.

INSTITUTO NACIONAL DE ESTADÍSTICA Y GEOGRAFÍA - INEGI/MEXICO (2013). Panorama de violencia contra las mujeres en los Estados Unidos Mexicanos. ENDIREH 2011.

INSTITUTO NACIONAL DE ESTADÍSTICA Y GEOGRAFÍA - INEGI/MEXICO (2016). Encuesta Nacional sobre la Dinámica de las Relaciones en los Hogares (ENDIREH) 2016.

INSTITUTO NACIONAL DE ESTATÍSTICA - INE/ANGOLA, MINSTÉRIO DA SAÚDE - MINSA/ANGOLA AND ICF (2017). Angola Inquérito de Indicadores Múltiplos e de Saúde (IIMS) 2015-2016. Luanda, Angola, INE, MINSA, and ICF.

INSTITUTO NACIONAL DE ESTATÍSTICA - INE/CABO VERDE (2008). Mulheres e Homens em Cabo Verde Factos e Números 2008. Cidade da Praia - Santiago, Cabo Verde, Instituto Nacional de Estatística - INE/Cabo Verde,: 61.

Instituto Nacional de Estatística - INE/São Tomé and ICF Macro (2010). São Tomé e Príncipe Inquérito Demográfico e Sanitário 2008-2009. Calverton, Maryland, USA, INE/São Tomé.

Instituto Nacional de Información de Desarrollo - INIDE/Nicaragua (2008). Encuesta Nicaragüense de Demografía y Salud - ENDESA (2006/07).

Instituto Nacional de Información de Desarrollo - INIDE/Nicaragua and Ministerio de Salud - MINSA/Nicaragua (2014). Encuesta Nicaragüense de Demografía y Salud (2011/12).

International Institute for Population Sciences - IIPS/India and Macro International (2007). India National Family Health Survey (NFHS-3) 2005-06. *Volume 1*. Mumbai, India, IIPS and Macro International.

Istituto Nazionale di Statistica - Istat/Italy (2007). La violenza e i maltrattamenti contro le donne dentro e fuori la famiglia. Rome, Italy, Istituto Nazionale di Statistica - Istat/Italy.

Istituto Nazionale di Statistica - Istat/Italy (2007). La violenza contro le donne dentre e fuori la famiglia Anno 2014.

Jain, D., S. Sanon, L. Sadowski and W. Hunter (2004). Violence against women in India: evidence from rural Maharashtra, India. *Rural Remote Health* **4**(4): 304.

Jaspard, M. and L'équipe Enveff (2001). Nommer et compter les violences envers les femmes : une première enquête nationale en France. *Populations et Sociétés*(364).

Johnson, H., N. Ollus and S. Nevala (2008). *Violence against women: An international perspective*.

Johnson, K., J. Scott, B. Rughita, M. Kisielewski, J. Asher, R. Ong and L. Lawry (2010). Association of Sexual Violence and Human Rights Violations With Physical and Mental Health in Territories of the Eastern Democratic Republic of the Congo. *JAMA* **304**(5): 553-562.

Joseph, J., C. Pemberton and U. Phillip (2019). National Women's Health Survey for Surina,e. D. M. King, Inter-American Development Bank.

Kenya National Bureau of Statistics, Ministry of Health - MOH/Kenya, National Aids Control Council - NACC/Kenya, Kenya Medical Research Institute and National Council for Population Development - NCPD/Kenya (2015). Kenya Demographic and Health Survey 2014. Rockville, MD, USA.

Kenya National Bureau of Statistics - KNBS, National Aids Control Council Kenya, National Aids STD Control Programme Kenya, Ministry of Public Health - MOPH/Kenya, Sanitation/Kenya and Kenya Medical Research Institute (2010). Kenya Demographic and Health Survey 2008-09. Calverton, Maryland, USA, KNBS and ICF Macro.

Kim, J. Y., S. Oh and S. I. Nam (2015). Prevalence and Trends in Domestic Violence in South Korea: Findings From National Surveys. *Journal of Interpersonal Violence* **31**(8): 1554-1576.

Koenig, M. A., T. Lutalo, F. Zhao, F. Nalugoda, N. Kiwanuka, F. Wabwire-Mangen, G. Kigozi, N. Sewankambo, J. Wagman, D. Serwadda, M. Wawer and R. Gray (2004). Coercive sex in rural Uganda: prevalence and associated risk factors. *Soc Sci Med* **58**(4): 787-798.

Koenig, M. A., T. Lutalo, F. Zhao, F. Nalugoda, F. Wabwire-Mangen, N. Kiwanuka, J. Wagman, D. Serwadda, M. Wawer and R. Gray (2003). Domestic violence in rural Uganda : evidence from a community-based study / Michael A. Koenig ... [et al.]. *Bulletin of the World Health Organization : the International Journal of Public Health 2003 ; 81(1) : 53-60*.

Kyu, N. and A. Kanai (2005). Prevalence, antecedent causes and consequences of domestic violence in Myanmar. *Asian Journal of Social Psychology* **8**(3): 244-271.

Lee, I., M. Chang, J. Hwang, M. Lee, J. Joo and S.-Y. Jung (2016). 2016 년 가정폭력 실태조사 연구 [The Domestic Violence Survey in 2016]. Seoul, Ministry of Gender Equality and Family - MOGEF/Korea.

Liberia Institute of Statistics Geo-Information Services - LISGIS/Liberia, Ministry of Health & Social Welfare - MOHSW/Liberia, National Aids Control Program - NACP/Liberia and Macro

INTERNATIONAL (2008). Liberia Demographic and Health Survey 2007. Monrovia, Liberia, LISGIS and Macro International.
LINDNER, S. R., E. B. S. COELHO, C. C. BOLSONI, P. F. ROJAS AND A. F. BOING (2015). [Prevalence of intimate partner physical violence in men and women from Florianópolis, Santa Catarina State, Brazil: a population-based study]. *Cadernos de saude publica* **31**(4): 815-826.
LOWENSTEIN, A., Z. EISIKOVITS, T. BAND-WINTERSTEIN AND G. ENOSH (2009). Is elder abuse and neglect a social phenomenon? Data from the First National Prevalence Survey in Israel. *J Elder Abuse Negl* **21**(3): 253-277.
MA'A FAFINE MO E FAMILI (2012). National Study on Domestic Violence against Women in Tonga 2009.
MACDOWALL, W., L. J. GIBSON, C. TANTON, C. H. MERCER, R. LEWIS, S. CLIFTON, N. FIELD, J. DATTA, K. R. MITCHELL, P. SONNENBERG, B. ERENS, A. J. COPAS, A. PHELPS, P. PRAH, A. M. JOHNSON AND K. WELLINGS (2013). Lifetime prevalence, associated factors, and circumstances of non-volitional sex in women and men in Britain: findings from the third National Survey of Sexual Attitudes and Lifestyles (Natsal-3). *The Lancet* **382**(9907): 1845-1855.
MACHISA, M., L. C, R. K AND J. R (2011). *The War at Home: The Gauteng GBV Indicators Research Study.: Gender Links and the South African Medical Research Council*.
MACHISA, M. AND R. DORP (2012). *The Gender Based Violence Indicators Project: Botswana*.
MAVRIKIOU, P. M., M. APOSTOLIDOU AND S. K. PARLALIS (2014). Risk factors for the prevalence of domestic violence against women in Cyprus. *The Social Science Journal* **51**(2): 295-301.
MCCLOSKEY, L. A., C. WILLIAMS AND U. LARSEN (2005). Gender Inequality and Intimate Partner Violence among Women in Moshi, Tanzania. *International Family Planning Perspectives* **31**(3): 124-130.
MEDINA-MORA ICAZA, M. E., C. BORGES-GUIMARAES G FAU - LARA, L. LARA C FAU - RAMOS-LIRA, J. RAMOS-LIRA L FAU - ZAMBRANO, C. ZAMBRANO J FAU - FLEIZ-BAUTISTA AND C. FLEIZ-BAUTISTA [Prevalence of violent events and post-traumatic stress disorder in the Mexican population]. (0036-3634 (Print)).
MINISTÈRE À LA PRÉSIDENCE CHARGÉ DE LA BONNE GOUVERNANCE ET DU PLAN - MPBGP/BURUNDI, MINISTÈRE DE LA SANTÉ PUBLIQUE ET DE LA LUTTE CONTRE LE SIDA - MSPLS/BURUNDI, INSTITUT DE STATISTIQUES ET D'ÉTUDES ÉCONOMIQUES DU BURUNDI - ISTEEBU AND ICF (2017). Burundi Troisième Enquête Démographique et de Santé 2016-2017. Bujumbura, Burundi, MPBGP, MSPLS, ISTEEBU, and ICF.
MINISTÈRE DE LA PLANIFICATION DU DÉVELOPPEMENT ET DE L'AMÉNAGEMENT DU TERRITOIRE - MPDAT/TOGO, MINISTÈRE DE LA SANTÉ - MS/TOGO AND ICF INTERNATIONAL (2015). Togo Enquête Démographique et de Santé 2013-2014. Rockville,Maryland, USA, MPDAT/Togo, MS/Togo and ICF International.
MINISTÈRE DU PLAN - MPC/CONGO AND MACRO INTERNATIONAL (2008). République Démocratique du Congo Enquête Démographique et de Santé 2007. Calverton, Maryland, USA, Ministère du Plan/Congo and Macro International.
MINISTÈRE DU PLAN ET SUIVI DE LA MISE EN ŒUVRE DE LA RÉVOLUTION DE LA MODERNITÉ - MPSMRM/CONGO, MINISTÈRE DE LA SANTÉ PUBLIQUE - MSP/CONGO AND ICF INTERNATIONAL (2014). République Démocratique du Congo Enquête Démographique et de Santé (EDS-RDC) 2013-2014. Rockville, Maryland, USA, MPSMRM, MSP, and ICF International.
MINISTÉRIO DA SAÚDE - MISAU/MOÇAMBIQUE, INSTITUTO NACIONAL DE ESTATÍSTICA - INE/MOÇAMBIQUE AND ICF (2018). Inquérito de Indicadores de Imunização, Malária e HIV/SIDA em Moçambique (IMASIDA) 2015. Maputo/Moçambique, MISAU/Moçambique, INE, and ICF.

MINISTERIO DA SAUDE - MISAU/MOÇAMBIQUE, INSTITUTO NACIONAL DE ESTATÍSTICA - INE/MOÇAMBIQUE AND ICF INTERNATIONAL (2013). Moçambique Inquérito Demográfico e de Saúde 2011. Calverton, Maryland, USA, MISA/Moçambique, INE/Moçambique and ICF International.
MINISTERIO DE JUSTICIA Y DERECHOS HUMANOS DE LA NACIÓN (2017). Primer estudio nacional sobre violencias contra la mujer, Basado en la International Violence Against Women Survey (IVAWS). Ciudad Autónoma de Buenos Aires, Ministerio de Justicia y Derechos Humanos de la Nación.
MINISTERIO DE LA MUJER - MM/PARAGUAY (2014). Primera Encuesta sobre Violencia Intrafamiliar basada en Género : Área Urbana, Informe Final, Ministerio de la Mujer, Centro Superior de Estudios de Administración y Finanzas Públicas para el Desarrollo: 144.
MINISTERIO DE SALUD PÚBLICA Y ASISTENCIA SOCIAL - MSPAS/GUATEMALA AND INSTITUTO NACIONAL DE ESTADÍSTICA - INE/GUATEMALA (2010). Encuesta Nacional de Salud Materno Infantil (2008-2009). Guatemala, Ministerio de Salud Pública y Asistencia Social, Mspas Guatemala: 670.
MINISTERIO DE SALUD PÚBLICA Y ASISTENCIA SOCIAL - MSPAS/GUATEMALA, INSTITUTO NACIONAL DE ESTADÍSTICA - INE/GUATEMALA AND SECRETARÍA DE PLANIFICACIÓN Y PROGRAMACIÓN DEL LA PRESIDENCIA - SEGEPLÁN/GUATEMALA (2017). Encuesta Nacional de Salud Materno Infantil 2014-2015, Ministerio de Salud Pública y Asistencia Social - MSPAS/Guatemala,
Instituto Nacional de Estadística - INE/Guatemala,
Secretaría de Planificación y Programación del la Presidencia, Segeplán Guatemala,.
MINISTERIO DE SALUD Y PROTECCIÓN SOCIAL Y PROFAMILIA (2017). Colombia Encuesta Nacional de Demografía y Salud 2015. Bogotá,Colombia, Profamilia/Colombia.
MINISTERIO DE SANIDAD Y BIENESTAR SOCIAL - MSBS/GUINEA ECUATORIAL, P. E. I. P.-M. G. E. MINISTERIO DE ECONOMÍA AND ICF INTERNATIONAL (2012). Guinea Ecuatorial Encuesta Demográfica y de Salud (EDSGE-I) 2011. Calverton, Maryland, USA, Ministerio de Sanidad y Bienestar Social, Ministerio de Economía, Planificación e Inversiones Públicas/Guinea Ecuatorial and ICF International.
MINISTERIO DEL INTERIOR Y SEGURIDAD PÚBLICA (2009). Encuesta Nacional de Victimización por Violencia Intrafamiliar y Delitos Sexuales 2008, Ministerio del Interior y Seguridad Pública.
MINISTERIO DEL INTERIOR Y SEGURIDAD PÚBLICA (2017). Tercera encuesta nacional de violencia intrafamiliar contra la mujer y delitos sexuales, Ministerio del Interior y Seguridad Pública.
MINISTRY OF HEALTH - MOH /BHUTAN (2014). 2012 National Health Survey (NHS). Thimphu, Bhutan.
MINISTRY OF HEALTH - MOH/IRAQ, MINISTRY OF PLANNING AND DEVELOPMENT COOPERATION - MPDC/IRAQ AND WORLD HEALTH ORGANIZATION - WHO (2007). Iraq Family Health Survey 2006/7.
MINISTRY OF HEALTH - MOH/MALDIVES AND ICF (2018). Maldives Demographic and Health Survey 2016-17. MalÈ, Maldives, MOH and ICF.
MINISTRY OF HEALTH - MOH/NEPAL, E. N. NEW AND ICF (2017). Nepal Demographic and Health Survey 2016. Kathmandu, Nepal, MOH/Nepal, New ERA, and ICF.
MINISTRY OF HEALTH - MOH/PALAU (2014). Belau Family Health and Safety Study: National Research Project on Violence Against Women in Palau.
MINISTRY OF HEALTH - MOH/ROMANIA (2005). Reproductive Health Survey Romania 2004.
MINISTRY OF HEALTH AND FAMILY - MOHF/MALDIVES AND ICF MACRO (2010). Maldives Demographic and Health Survey 2009. Calverton, Maryland, USA, MOHF and ICF Macro.
MINISTRY OF HEALTH AND SOCIAL SERVICES - MOHSS/NAMIBIA AND ICF INTERNATIONAL (2014). Namibia Demographic and Health Survey 2013. Windhoek, Namibia, MoHSS/Namibia and ICF International.
MINISTRY OF HEALTH AND SPORTS - MOHS/MYANMAR AND ICF (2017). Myanmar Demographic and Health Survey 2015-16. Nay Pyi Taw, Myanmar, MoHS and ICF.
MINISTRY OF HEALTH COMMUNITY DEVELOPMENT GENDER ELDERLY CHILDREN - MOHCDGEC/TANZANIA MAINLAND, MINISTRY OF HEALTH - MOH/ZANZIBAR, NATIONAL BUREAU OF STATISTICS - NBS/TANZANIA, OFFICE OF CHIEF GOVERNMENT STATISTICIAN - OCGS/ZANZIBAR AND ICF (2016). Tanzania Demographic

and Health Survey and Malaria Indicator Survey 2015-2016. Dar es Salaam, Tanzania, MoHCDGEC, MoH, NBS, OCGS, and ICF.
Ministry of Home Affairs - MHA/Nauru (2014). Nauru Family Health and Support Study: An exploratory study on violence against women.
Ministry of Internal Affairs (2014). Republic of the Marshall Islands National Study on Family Health and Safety**:** 292.
Ministry of Justice and Human Rights - MJHR/Montegegro and UNDP (2012). Study on Family Violence and Violence Against Women in Montenegro.
Ministry of National Economy of the Republic of Kazakhstan - MNERK/Kazakhstan (2017). Sample Survey on Violence Against Women in Kazakhstan. Astana**:** 246.
Ministry of Population Health - MOPH/Egypt, El-Zanaty Associates/Egypt and ICF International (2015). Egypt Demographic and Health Survey 2014. Cairo, Egypt, Ministry of Health and Population and ICF International.
Ministry of Women Youth & Children's Affairs - MWYCA/Solomon Islands (2009). Solomon Islands Family Health and Safety Study: A study on violence against women and children**:** 238.
Ministry of Women's Affairs - MoWA/Cambodia (2015). National Survey on Women's Health and Life Experiences in Cambodia, Ministry of Women's Affairs - MoWA/Cambodia,**:** 91.
MOHP/Nepal, M. o. H. P.-., New ERA/Nepal and ICF International (2012). Nepal Demographic and Health Survey 2011. Kathmandu, Nepal, MOHP/Nepal, New ERA, and ICF International.
Moore, A. M., K. Awusabo-Asare, N. Madise, J. John-Langba and A. Kumi-Kyereme (2007). Coerced first sex among adolescent girls in sub-Saharan Africa: prevalence and context. *African journal of reproductive health* **11**(3): 62-82.
Moracco, K. E., C. W. Runyan, J. M. Bowling and J. A. L. Earp (2007). Women's experiences with violence: A national study. *Women's Health Issues* **17**(1): 3-12.
Morris, L., J. Herold, S. Brino, A. Yili and D. Jackson (2005). Albania Reproductive Health Survey 2002.
Mousavi, S. M. and A. Eshagian Wife abuse in Esfahan, Islamic Republic of Iran, 2002. (1020-3397 (Print)).
Muganyizi, P. S., C. Kilewo and C. Moshiro (2004). Rape against Women: The Magnitude, Perpetrators and Patterns of Disclosure of Events in Dar es Salaam, Tanzania. *African Journal of Reproductive Health / La Revue Africaine de la Santé Reproductive* **8**(3): 137-146.
Müller, U., M. Schröttle and S. Glammeier (2004). Health, well-being and personal safety of women in Germany: A representative study of violence against women in Germany. *Baden-Baden: Federal Ministry for Family Affairs Senior Citizens Women and Youth*.
Munné, M. I. (2005). Alcohol and the economic crisis in Argentina: recent findings*. *Addiction* **100**(12): 1790-1799.
Murty, S. A., C. Peek-Asa, C. Zwerling, A. M. Stromquist, L. F. Burmeister and J. A. Merchant (2003). Physical and emotional partner abuse reported by men and women in a rural community. *American journal of public health* **93**(7): 1073-1075.
National Bureau of Statistics - N. B. S. Tanzania and I. C. F. Macro (2011). Tanzania Demographic and Health Survey 2010. Dar es Salaam, Tanzania, NBS/Tanzania and ICF Macro.
National Bureau of Statistics - NBS/Moldova (2011). Violence against Women in the Family in the Republic of Moldova. Chisinau, Moldova.
National Commission for the Advancement of Women - NCAW/Lao (2015). Lao National Survey on Women's Health and Life Experiences 2014 - A Study on Violence against Women, Lao PDR.
National Commission for Women and Children (2012). Situation of Violence Against Women in Bhutan. Thimphu, Bhutan, National Commission for Women and Children.

NATIONAL COMMISSION FOR WOMEN AND CHILDREN (2019). National Survey on Women's Health and Life Experiences 2017: A Study on Violence Against Women and Girls in Bhutan. Thimphu, Bhutan, National Commission for Women and Children.
NATIONAL DEPARTMENT OF HEALTH AND ICF (2019). South Africa Demographic and Health Survey 2016. Pretoria, National Department of Health - NDoH - ICF.
NATIONAL INSTITUTE OF POPULATION RESEARCH TRAINING - NIPORT/BANGLADESH, MITRA, ASSOCIATES/BANGLADESH AND MACRO INTERNATIONAL (2009). Bangladesh Demographic and Health Survey 2007. Dhaka, Bangladesh, NIPORT, Mitra and Associates, and Macro International.
NATIONAL INSTITUTE OF POPULATION RESEARCH TRAINING - NIPORT/BANGLADESH, MITRA ASSOCIATES AND ICF INTERNATIONAL (2016). Bangladesh Demographic and Health Survey 2014. Dhaka, Bangladesh, NIPORT, Mitra and Associates, and ICF International.
NATIONAL INSTITUTE OF POPULATION RESEARCH TRAINING - NIPORT/BANGLADESH, MITRA ASSOCIATES/BANGLADESH AND ICF INTERNATIONAL (2013). Bangladesh Demographic and Health Survey 2011. Dhaka, Bangladesh, NIPORT, Mitra and Associates, and ICF International.
NATIONAL INSTITUTE OF POPULATION STUDIES - NIPS/PAKISTAN AND ICF (2019). Pakistan Demographic and Health Survey 2017-18. Islamabad, Pakistan, NIPS/Pakistan and ICF.
NATIONAL INSTITUTE OF POPULATION STUDIES - NIPS/PAKISTAN AND ICF INTERNATIONAL (2013). Pakistan Demographic and Health Survey 2012-13. Islamabad, Pakistan, NIPS/Pakistan and ICF International.
NATIONAL INSTITUTE OF PUBLIC HEALTH - NIPH/CAMBODIA, NATIONAL INSTITUTE OF STATISTICS - NIS/CAMBODIA AND ORC MACRO (2006). Cambodia Demographic and Health Survey 2005. Phnom Penh, Cambodia, National Institute of Public Health/Cambodia, National Institute of Statistics/Cambodia, and ORC Macro.
NATIONAL INSTITUTE OF STATISTICS - NIS/CAMBODIA, DIRECTORATE GENERAL FOR HEALTH - DGH/CAMBODIA AND ICF INTERNATIONAL (2015). Cambodia Demographic and Health Survey 2014. Phnom Penh, Cambodia, National Institute of Statistics/Cambodia, Directorate General for Health/Cambodia, and ICF International.
NATIONAL INSTITUTE OF STATISTICS - NIS/CAMBODIA, DIRECTORATE GENERAL FOR HEALTH - DGH/CAMBODIA AND ORC MACRO (2001). Cambodia Demographic and Health Survey 2000. Phnom Penh, Cambodia, National Institute of Statistics/Cambodia, Directorate General for Health/Cambodia, and ORC Macro.
NATIONAL INSTITUTE OF STATISTICS - NIS/CAMBODIA, DIRECTORATE GENERAL FOR HEALTH - DGH/CAMBODIA AND ORC MACRO (2001). Cambodia Demographic and Health Survey 2000. Phnom Penh, Cambodia, National Institute of Statistics/Cambodia, Directorate General for Health/Cambodia, and ORC Macro.
NATIONAL INSTITUTE OF STATISTICS OF RWANDA, MINISTRY OF FINANCE AND ECONOMIC PLANNING - MFEP/RWANDA, MINISTRY OF HEALTH - MOH/RWANDA AND ICF INTERNATIONAL (2016). Rwanda Demographic and Health Survey 2014-15. Kigali, Rwanda, National Institute of Statistics of Rwanda, Ministry of Finance and Economic Planning/Rwanda, Ministry of Health/Rwanda, and ICF International.
NATIONAL INSTITUTE OF STATISTICS OF RWANDA - NISR, MINISTRY OF HEALTH - MOH/RWANDA AND ICF INTERNATIONAL (2012). Rwanda Demographic and Health Survey 2010. Calverton, Maryland, USA, NISR/Rwanda, MOH/Rwanda, and ICF International.
NATIONAL POPULATION COMMISSION - NPC/NIGERIA AND ICF (2019). Nigeria Demographic and Health Survey 2018 - Final Report. Abuja, Nigeria, NPC and ICF.
NATIONAL POPULATION COMMISSION - NPC/NIGERIA AND ICF INTERNATIONAL (2014). Nigeria Demographic and Health Survey 2013. Abuja, Nigeria, NPC/Nigeria and ICF International.

National Population Commission - NPC/Nigeria and ICF Macro (2009). Nigeria Demographic and Health Survey 2008. Abuja, Nigeria, NPC/Nigeria and ICF Macro.
National Scientific Applied Center for Preventive Medicine - NCPM/Moldova and ORC Macro (2006). Moldova Demographic and Health Survey 2005. Calverton, Maryland, USA, National Scientific and Applied Center for Preventive Medicine of the Ministry of Health and Social Protection and ORC Macro.
National Statistical Committee of the Kyrgyz Republic -NSCKR/Kyrgys Republic, Ministry of Health - MOH/Kyrgyz Republic and ICF International (2013). Kyrgyz Republic Demographic and Health Survey 2012. Bishkek, Kyrgyz Republic, NSC, MOH, and ICF International.
National Statistical Office - NSO/Malawi and ICF Macro (2011). Malawi Demographic and Health Survey 2010. Zomba, Malawi, NSO/Malawi and ICF Macro.
National Statistical Office - NSO/Malawi and ORC Macro (2005). Malawi Demographic and Health Survey 2004. Calverton, Maryland, USA, NSO/Malawi and ORC Macro.
National Statistical Office - NSO/Papua New Guinea and ICF (2019). Papua New Guinea Demographic and Health Survey 2016-18. Port Moresby, Papua New Guinea, NSO and ICF.
National Statistical Office Malawi and ICF (2017). Malawi Demographic and Health Survey 2015-16. Zomba, Malawi, National Statistical Office and ICF.
National Statistical Service - NSS/Armenia, Ministry of Health - MOH/Armenia and ICF (2017). Armenia Demographic and Health Survey 2015-16. Yerevan, Armenia, NSS, MOH, and ICF.
National Statistics Directorate - NSD/Timor-Leste, Ministry of Finance - MOF/Timor-Leste and ICF Macro (2010). Timor-Leste Demographic and Health Survey 2009-10. Dili, Timor-Leste, NSD/Timor-Leste and ICF Macro.
National Statistics Office - NSO/Mongolia (2018). Breaking the silence for equality: 2017 National Study on Gender-based Violence in Mongolia. Ulaanbaatar, Mongolia**:** 224.
National Statistics Office - NSO/Philippines and ICF Macro (2009). Philippines National Demographic and Health Survey 2008. Calverton, Maryland, USA, NSO/Philippines and ICF Macro.
Navarro-Mantas, L., M. Velásquez and J. Megías (2015). *Violencia contra las mujeres El Salvador Estudio poblacional*.
Nerøien, A. I. and B. Schei (2008). Partner violence and health: Results from the first national study on violence against women in Norway. *Scandinavian Journal of Public Health* **36**(2): 161-168.
Nguyen, D. V., G. Ostergren Po Fau - Krantz and G. Krantz (2008). Intimate partner violence against women in rural Vietnam--different socio-demographic factors are associated with different forms of violence: need for new intervention guidelines? (1471-2458 (Electronic)).
Nguyen, P. H., S. V. Nguyen, M. Q. Nguyen, N. T. Nguyen, S. C. Keithly, L. T. Mai, L. T. T. Luong and H. Q. Pham (2012). The association and a potential pathway between gender-based violence and induced abortion in Thai Nguyen province, Vietnam. *Global health action* **5**: 1-11.
Nicholson, C. and H. Deshong (2020). *Grenada Women's Health and Life Experiences Study*.
Nybergh, L., C. Taft, V. Enander and G. Krantz (2013). Self-reported exposure to intimate partner violence among women and men in Sweden: results from a population-based survey. *BMC Public Health* **13**(1): 845.
O'Leary, K. D., E. J. Tintle N Fau - Bromet, S. F. Bromet Ej Fau - Gluzman and S. F. Gluzman Descriptive epidemiology of intimate partner aggression in Ukraine. (1433-9285 (Electronic)).
Office of National Statistics - ONS/United Kingdom (2015). Crime Survey for England and Wales (CSEW).
Ojeda, G., M. Ordóñez and L. H. Ochoa (2005). Colombia Salud Sexual y Reproductiva. *Resultados Encuesta Nacional de Demografía y Salud 2005*. Bogotá, Colombia, Profamilia/Colombia.

OJEDA, G., M. ORDÓÑEZ AND L. H. OCHOA (2011). Colombia Encuesta Nacional de Demografía y Salud 2010. Bogotá, Colombia, Profamilia/Colombia.

ONIGBOGI, M. O., K. A. ODEYEMI AND O. O. ONIGBOGI (2015). Prevalence and Factors Associated with Intimate Partner Violence among Married Women in an Urban Community in Lagos State, Nigeria. *Afr J Reprod Health* **19**(1): 91-100.

ORGANIZATION FOR SECURITY AND CO-OPERATION IN EUROPE - OSCE (2019). Well-Being and Safety of Women. Vienna Organization for Security and Co-operation in Europe - OSCE**:** 163.

ÖSTERREICHISCHES INSTITUT FÜR FAMILIENFORSCHUNG (2011). Gewalt in der Familie und im sozialen Nahraum, Österreichische Prävalenzstudie zur Gewalt an Frauen und Männern (Violence in the family and social environment, Austrian prevalence study on violence against women and men). Vienna, Gefördert aus Mitteln des Bundesministeriums für Wirtschaft, Familie und Jugend über die Familie & Beruf Management GmbH**:** 303.

OUAGADJIO, B., K. NODJIMADJI, T. BAGAMLA, R. MADNODJI AND J. S. TOKINDANG (2005). Enquête Démographique et de Santé Tchad 2004. Calverton, Maryland, USA, Institut National de la Statistique, des Études Économiques et Démographiques - INSEED/Tchad and ORC Macro.

OWOAJE, E. T. AND F. M. OLAOLORUN (2006). Intimate Partner Violence among Women in a Migrant Community in Southwest Nigeria. *International Quarterly of Community Health Education* **25**(4): 337-349.

PALESTINIAN CENTRAL BUREAU OF STATISTICS - PCBS/PALESTINIAN NATIONAL AUTHORITY (2011). Palestine - Violence Survey in the Palestinian Society, 2011.

PANDEY, G. K., D. DUTT AND B. BANERJEE (2008). Partner and Relationship Factors in Domestic Violence: Perspectives of Women From a Slum in Calcutta, India. *Journal of Interpersonal Violence* **24**(7): 1175-1191.

PEMBERTON, C. AND J. JOSEPH (2018). National Women's Health Survey for Trinidad and Tobago: Final Report, Inter-American Development Bank.

PESHEVSKA, D. J., M. MARKOVIK, D. SETHI, E. SERAFIMOVSKA AND T. JORDANOVA (2014). Prevalence of Elder Abuse and Neglect: Findings from First Macedonian Study. *Macedonian Journal of Medical Sciences* **7**(2): 355.

PHILIPPINES STATISTICS AUTHORITY - PSA/PHILIPPINES AND ICF (2018). Philippines National Demographic and Health Survey 2017. Quezon City, Philippines, PSA and ICF.

PIETERS, J., P. ITALIANO, A.-M. OFFERMANS AND S. HELLEMANS (2010). Emotional, physical and sexual abuse – the experiences of women and men. Brussels, Institute for the Equality of Women and Men.

PROJET DE COOPéRATION ONFP/AECID « PROMOTION DE L'EQUITé DE GENRE ET PRéVENTION DE LA VIOLENCE à L'EGARD DES FEMMES » (2010). Enquête nationale sur la violence à l'égard des femmes en Tunisie: rapport de l'enquête. Tunis.

PSA/PHILIPPINES, P. S. A.-. AND ICF INTERNATIONAL (2014). Philippines National Demographic and Health Survey 2013. Manila, Philippines, PSA and ICF International.

PURVANECKIENE, G. (2000). Violence Against Women: Victim Survey Report.

RAHMATIAN, A. A. AND S. A. A. HOSSEINI (2015). Domestic Abuse in Behshahr, Iran. *Iranian journal of psychiatry and behavioral sciences* **9**(4): e1790-e1790.

RASANATHAN, J. AND A. BHUSHAN (2011). Measuring and responding to gender-based violence in the Pacific: Action on gender inequality as a social determinant of Health Republic of Kiribati, Ministry of Internal and Social Affairs, World Health Organization Regional Office for the Western Pacific.

RASOULIAN, M., S. HABIB, J. BOLHARI, M. HAKIM SHOOSHTARI, M. NOJOMI AND S. ABEDI (2014). Risk factors of domestic violence in Iran. *Journal of environmental and public health* **2014**: 352346-352346.

REINGARDIENE, J. (2003). Dilemmas in private/public discourse: Contexts for gender-based violence against women in Lithuania. *Journal of Baltic Studies* **34**(3): 354-368.
RINFRET-RAYNOR, M., A. RIOU, S. CANTIN, C. DROUIN AND M. DUBÉ (2004). A Survey on Violence Against Female Partners in Québec, Canada. *Violence Against Women* **10**(7): 709-728.
RUIKAR, M. AND A. PRATINIDHI (2008). Physical wife abuse in an urban slum of Pune, Maharastra. *Indian journal of public health* **52**: 215-217.
SALAM, A., A. ALIM AND T. NOGUCHI (2006). Spousal abuse against women and its consequences on reproductive health: a study in the urban slums in Bangladesh. *Matern Child Health J* **10**(1): 83-94.
SAMAKÉ, S., S. M. TRAORÉ, S. BA, É. DEMBÉLÉ AND M. DIOP (2007). Enquête Démographique et de Santé du Mali 2006. Calverton, Maryland, USA, Cellule de Planification et de Statistique du Ministère de la Santé - CPS/MS/Mali, Direction Nationale de la Statistique et de l'Informatique du Ministère de l'Économie, de l'Industrie et du Commerce - DNSI/MEIC/Mali and Macro International.
SANTAULARIA, J., M. JOHNSON, L. HART, L. HASKETT, E. WELSH AND B. FASERU (2014). Relationships between sexual violence and chronic disease: a cross-sectional study. *BMC Public Health* **14**(1): 1286.
SANZ-BARBERO, B., C. VIVES-CASES, L. OTERO-GARCÍA, C. MUNTANER, J. TORRUBIANO-DOMÍNGUEZ AND Y. P. O'CAMPO (2015). Intimate partner violence among women in Spain: the impact of regional-level male unemployment and income inequality. *European Journal of Public Health* **25**(6): 1105-1111.
SARDÁN, M. G., L. H. OCHOA AND W. CASTILLO GUERRA (2004). Bolivia Encuesta Nacional de Demografía y Salud 2003. Calverton, Maryland, USA, Instituto Nacional de Estadística - INE/Bolivia and ORC Macro.
SCHLACK, R., J. RÜDEL, A. KARGER AND H. HÖLLING (2013). [Physical and psychological violence perpetration and violent victimisation in the German adult population: results of the German Health Interview and Examination Survey for Adults (DEGS1)]. *Bundesgesundheitsblatt Gesundheitsforschung Gesundheitsschutz* **56**(5-6): 755-764.
SEBRANESCU, F., A. RUIZ AND D. SUCHDEV (2010). Reproductive Health Survey Jamaica 2008 Final Report. Atlanta, GA (USA) and Kingston, Jamaica, National Family Planning Board, United States Agency for International Development (USAID), Division of Reproductive Health-Centers for Disease Control and Prevention (CDC).
SECRETARÍA DE SALUD - SS/HONDURAS, INSTITUTO NACIONAL DE ESTADÍSTICA - INE/HONDURAS AND ICF INTERNATIONAL (2013). Honduras Encuesta Nacional de Salud y Demografía 2011-2012. Tegucigalpa, Honduras, SS, INE and ICF International.
SECRETARÍA DE SALUD - SS/HONDURAS, INSTITUTO NACIONAL DE ESTADÍSTICA - INE/HONDURAS AND MACRO INTERNATIONAL (2006). Honduras Encuesta Nacional de Demografía y Salud 2005-2006. Tegucigalpa, Honduras, SS, INE and Macro.
SEEDHOM, A. E. (2012). Sociodemographic associations of intimate partner violence against women in a rural area, El-Minia governorate, Egypt, 2010. *Journal of Public Health* **20**(1): 81-88.
SEMAHEGN, A., T. BELACHEW AND M. ABDULAHI (2013). Domestic violence and its predictors among married women in reproductive age in Fagitalekoma Woreda, Awi zone, Amhara regional state, North Western Ethiopia. *Reproductive health* **10**: 63-63.
SERBANESCU, F., L. MORRIS, S. RAHIMOVA AND P. W. STUPP (2003). Reproductive health survey Azerbaijan, 2001 : final report.
SHERRY LIPSKY , P. D., M.P.H. , AND M. D. RAUL CAETANO , PH.D. , (2007). Impact of Intimate Partner Violence on Unmet Need for Mental Health Care: Results From the NSDUH. *Psychiatric Services* **58**(6): 822-829.
SHUIB, R., N. ENDUT, S. H. ALI, I. OSMAN, S. ABDULLAH, S. W. OON, P. A. GHANI, G. PRABAKARAN, N. S. HUSSIN AND S. S. H. SHAHRUDIN (2013). Domestic Violence and Women's Well-being in Malaysia:

Issues and Challenges Conducting a National Study Using the WHO Multi-country Questionnaire on Women's Health and Domestic Violence Against Women. *Procedia - Social and Behavioral Sciences* **91**: 475-488.
SINHA, M. (2011). Family violence in Canada: A statistical profile, 2011. *Juristat*(85-002-X).
SIPSMA, H., A. OFORI-ATTA, M. CANAVAN, I. OSEI-AKOTO, C. UDRY AND E. H. BRADLEY (2013). Poor mental health in Ghana: who is at risk? *BMC Public Health* **13**(1): 288.
SIZIYA, S., A. MUULA, A. MANSOUR, E. RUDATSIKIRA, S. NZALA, C. ZYAMBO, P. SONGOLO AND O. BABANIYI (2014). Zambia: Forced sex among females: 117-127.
SMITH, A. M., J. RISSEL CE FAU - RICHTERS, A. E. RICHTERS J FAU - GRULICH, R. O. GRULICH AE FAU - DE VISSER AND R. O. DE VISSER Sex in Australia: a guide for readers. (1326-0200 (Print)).
SMITH, S. G., X. ZHANG, K. C. BASILE, M. T. MERRICK, J. WANG, M.-J. KRESNOW AND J. CHEN (2018). The National Intimate Partner and Sexual Violence Survey (NISVS): 2015 Data Brief – Updated Release. Atlanta, GA, National Center for Injury Prevention and Control, Centers for Disease Control and Prevention.
STATE STATISTICAL COMMITTEE - SSC/AZERBAIJAN AND MACRO INTERNATIONAL (2008). Azerbaijan Demographic and Health Survey 2006. Calverton, Maryland, USA, State Statistical Committee/Azerbaijan and Macro International.
STATISTICAL AGENCY UNDER THE PRESIDENT OF THE REPUBLIC OF TAJIKISTAN - SAPRT/TAJIKISTAN, MINISTRY OF HEALTH - MOH/TAJIKISTAN AND ICF (2018). Tajikistan Demographic and Health Survey 2017. Dushanbe, Tajikistan, SA/Tajikistan, MOH/Tajikistan, and ICF.
STATISTICAL AGENCY UNDER THE PRESIDENT OF THE REPUBLIC OF TAJIKISTAN - SAPRT/TAJIKISTAN, MINISTRY OF HEALTH - MOH/TAJIKISTAN AND ICF INTERNATIONAL (2013). Tajikistan Demographic and Health Survey 2012. SA/Tajikistan, MOH/Tajikistan, and ICF International, Dushanbe, Tajikistan.
STATISTICS CANADA (2004). General Social Survey - Victimization (GSS).
STATISTICS SIERRA LEONE - SSL AND ICF INTERNATIONAL (2014). Sierra Leone Demographic and Health Survey 2013. SSL and ICF International, Freetown, Sierra Leone.
STEEL, J. L. AND C. A. HERLITZ (2005). The association between childhood and adolescent sexual abuse and proxies for sexual risk behavior: A random sample of the general population of Sweden. *Child Abuse & Neglect* **29**(10): 1141-1153.
STENE, L. E., G. DYB, G. W. JACOBSEN AND B. SCHEI (2010). Psychotropic drug use among women exposed to intimate partner violence: A population-based study. *Scandinavian Journal of Public Health* **38**(5_suppl): 88-95.
STÖCKL, H. AND B. PENHALE (2014). Intimate Partner Violence and Its Association With Physical and Mental Health Symptoms Among Older Women in Germany. *Journal of Interpersonal Violence* **30**(17): 3089-3111.
SVAVARSDOTTIR, E. K. AND B. ORLYGSDOTTIR (2009). Intimate partner abuse factors associated with women's health: a general population study. *J Adv Nurs* **65**(7): 1452-1462.
TAYLOR, B., J. REHM, J. TRINIDAD, C. ABURTO, J. BEJARANO, C. CAYETANO, F. KERR-CORREA, M. PIAZZA-FERRAND, G. GMEL, K. GRAHAM, T. GREENFIELD, R. LARANJEIRA, M. LIMA, R. MAGRI, M. MONTEIRO, M. MEDINA-MORA, M. MUNNE, M. P. MENDOZA, A. TUCCI AND S. WILSNACK (2007). Alcohol, gender, culture and harms in the Americas: PAHO Multicentric Study final report.
THE ASIA FOUNDATION (2016). Understanding Violence against Women and Children in Timor-Leste: Findings from the Nabilan Baseline Study – Main Report. Dili, The Asia Foundation.
THE GAMBIA BUREAU OF STATISTICS - GBOS/GAMBIA AND ICF INTERNATIONAL (2014). The Gambia Demographic and Health Survey 2013. Banjul, The Gambia, GBOS and ICF International.

TUMWESIGYE, N. M., G. B. KYOMUHENDO, T. K. GREENFIELD AND R. K. WANYENZE (2012). Problem drinking and physical intimate partner violence against women: evidence from a national survey in Uganda. *BMC Public Health* **12**(1): 399.
UBOS/UGANDA, U. B. O. S.-. AND ICF (2018). Uganda Demographic and Health Survey 2016. Kampala, Uganda, UBOS and ICF.
UGANDA BUREAU OF STATISTICS - UBOS/UGANDA AND ICF INTERNATIONAL (2012). Uganda Demographic and Health Survey 2011. Kampala, Uganda, UBOS and ICF International.
UGANDA BUREAU OF STATISTICS - UBOS/UGANDA AND MACRO INTERNATIONAL (2007). Uganda Demographic and Health Survey 2006. Calverton, Maryland, USA, UBOS and Macro International.
UKRAINIAN CENTER FOR SOCIAL REFORMS - UCSR/UKRAINE, STATE STATISTICAL COMMITTEE - SSC/UKRAINE, MINISTRY OF HEALTH - MOH/UKRAINE AND MACRO INTERNATIONAL (2008). Ukraine Demographic and Health Survey 2007. Calverton, Maryland, USA, UCSR and Macro International.
ULLOA, E. C. AND J. F. HAMMETT (2014). The Effect of Gender and Perpetrator–Victim Role on Mental Health Outcomes and Risk Behaviors Associated With Intimate Partner Violence. *Journal of Interpersonal Violence* **31**(7): 1184-1207.
UMUBYEYI, A., I. MOGREN, J. NTAGANIRA AND G. KRANTZ (2014). Women are considerably more exposed to intimate partner violence than men in Rwanda: results from a population-based, cross-sectional study. *BMC Women's Health* **14**(1): 99.
UN WOMEN AND GEOSTAT (2018). National study on Violence against Women in Georgia 2017, UN Women, GEOSTAT.
UNDP (2016). Study on women's and men's health and life experiences in Papua, Indonesia, UNDP.
VAN PARYS, A.-S., E. DESCHEPPER, K. MICHIELSEN, A. GALLE, K. ROELENS, M. TEMMERMAN AND H. VERSTRAELEN (2015). Intimate partner violence and psychosocial health, a cross-sectional study in a pregnant population. *BMC Pregnancy and Childbirth* **15**(1): 278.
VANUATU WOMEN'S CENTRE (2011). The Vanuatu National Survey on Women's lives and Family Relationships. Port Vila, Vanuatu.
VINCK, P. AND P. N. PHAM (2013). Association of exposure to intimate-partner physical violence and potentially traumatic war-related events with mental health in Liberia. *Soc Sci Med* **77**: 41-49.
WALI, R., A. KHALIL, R. ALATTAS, R. FOUDAH, I. MEFTAH AND S. SARHAN (2020). Prevalence and risk factors of domestic violence in women attending the National Guard Primary Health Care Centers in the Western Region, Saudi Arabia, 2018. *BMC Public Health* **20**(1): 239.
WALSH, K., H. S. RESNICK, C. K. DANIELSON, J. L. MCCAULEY, B. E. SAUNDERS AND D. G. KILPATRICK (2014). Patterns of drug and alcohol use associated with lifetime sexual revictimization and current posttraumatic stress disorder among three national samples of adolescent, college, and household-residing women. *Addict Behav* **39**(3): 684-689.
WASZAK GEARY, C., M. WEDDERBURN, D. MCCARRAHER, C. CUTHBERTSON AND A. POTTINGER (2006). Sexual Violence and Reproductive Health Among Young People in Three Communities in Jamaica. *Journal of Interpersonal Violence* **21**(11): 1512-1533.
WATSON, D. AND S. PARSONS (2005). Domestic abuse of women and men in Ireland: report on the national study of domestic abuse, Government Publications Office.
WIJMA, K., L. SAMELIUS, G. WINGREN AND B. WIJMA (2007). The association between ill-health and abuse: A cross-sectional population based study. *Scandinavian Journal of Psychology* **48**(6): 567-575.
WILLIAMS, C. W. (2018). *Women's Health Survey 2016: Jamaica: Final Report*, Inter-American Development Bank.

YOUNG R, M. V. B. B. I. F. L. D. (2016). Family and community safety with emphasis on the situation of gender-based violence in Belize: Belize Public Health Survey 2015, Belize Institute For Local Development.

YÜKSEL-KAPTANOĞLU, İ., A. S. TÜRKYILMAZ AND L. HEISE (2012). What Puts Women at Risk of Violence From Their Husbands? Findings From a Large, Nationally Representative Survey in Turkey. *Journal of Interpersonal Violence* **27**(14): 2743-2769.

ZALESKI, M., I. PINSKY, R. LARANJEIRA, S. RAMISETTY-MIKLER AND R. CAETANO (2009). Intimate Partner Violence and Contribution of Drinking and Sociodemographics: The Brazilian National Alcohol Survey. *Journal of Interpersonal Violence* **25**(4): 648-665.

ZALESKI, M., I. PINSKY, R. LARANJEIRA, S. RAMISETTY-MIKLER AND R. CAETANO (2010). Intimate partner violence and alcohol consumption. *Rev Saude Publica* **44**(1): 53-59.

ZHANG, H., W. C. W. WONG, P. IP, S. FAN AND P. S. F. YIP (2014). Intimate Partner Violence Among Hong Kong Young Adults: Prevalence, Risk Factors, and Associated Health Problems. *Journal of Interpersonal Violence* **30**(13): 2258-2277.

ZIMBABWE NATIONAL STATISTICS AGENCY - ZIMSTAT/ZIMBABWE AND ICF INTERNATIONAL (2012). Zimbabwe Demographic and Health Survey 2010-11. Calverton, Maryland, USA, ZIMSTAT and ICF International.

ZIMBABWE NATIONAL STATISTICS AGENCY - ZIMSTAT/ZIMBABWE AND ICF INTERNATIONAL (2016). Zimbabwe Demographic and Health Survey 2015: Final Report. Rockville, Maryland, USA, Zimbabwe National Statistics Agency (ZIMSTAT) and ICF International.

ZINZOW, H. M., H. S. RESNICK, J. L. MCCAULEY, A. B. AMSTADTER, K. J. RUGGIERO AND D. G. KILPATRICK (2012). Prevalence and risk of psychiatric disorders as a function of variant rape histories: results from a national survey of women. *Soc Psychiatry Psychiatr Epidemiol* **47**(6): 893-902.
